# NRXN1 is associated with enlargement of the temporal horns of the lateral ventricles in psychosis

**DOI:** 10.1038/s41398-019-0564-9

**Published:** 2019-09-17

**Authors:** Ney Alliey-Rodriguez, Tamar A. Grey, Rebecca Shafee, Huma Asif, Olivia Lutz, Nicolas R. Bolo, Jaya Padmanabhan, Neeraj Tandon, Madeline Klinger, Katherine Reis, Jonathan Spring, Lucas Coppes, Victor Zeng, Rachal R. Hegde, Dung T. Hoang, Deepthi Bannai, Uzma Nawaz, Philip Henson, Siyuan Liu, Diane Gage, Steven McCarroll, Jeffrey R. Bishop, Scot Hill, James L. Reilly, Rebekka Lencer, Brett A. Clementz, Peter Buckley, David C. Glahn, Shashwath A. Meda, Balaji Narayanan, Godfrey Pearlson, Matcheri S. Keshavan, Elena I. Ivleva, Carol Tamminga, John A. Sweeney, David Curtis, Judith A. Badner, Sarah Keedy, Judith Rapoport, Chunyu Liu, Elliot S. Gershon

**Affiliations:** 1 University of Chicago, Department of Psychiatry and Behavioral Neurosciences, Chicago, USA; 2 Massachusetts Institute of Technology, Cambridge, USA; 3 Harvard Medical School, Department of Genetics, Boston, USA; 4 Stanley Center, Broad Institute of MIT and Harvard, Cambridge, USA; 5 Harvard Medical School, Department of Psychiatry, Boston, USA; 6 University of Chicago Laboratory for Advanced Computing, Chicago, USA; 7 Harvard University, Cambridge, USA; 8 Boston University, Boston, USA; 9 Child Psychiatry Branch, National Institutes of Mental Health, National Institutes of Health, Bethesda, MD USA; 10 Broad Institute of MIT and Harvard, Cambridge, USA; 11 University of Minnesota, Department of Experimental and Clinical Pharmacology and Department of Psychiatry, Minneapolis, USA; 12 Rosalind Franklin University, North Chicago, USA; 13 Northwestern University, Evanston, USA; 14 University of Muenster, Munster, Germany; 15 Department of Psychology, University of Georgia, Athens, Georgia; 16 Virginia Commonwealth University, Richmond, USA; 17 Yale University Departments of Psychiatry & Neuroscience, New Haven, USA; 18 University of Texas Southwestern Medical Center, Department of Psychiatry, Dallas, USA; 19 University College London and Centre for Psychiatry, Barts and the London School of Medicine and Dentistry, London, UK; 20 Rush University Medical Center, Chicago, USA; 21 SUNY Upstate Medical University, Binghamton, USA; 22 University of Chicago, Department of Human Genetics, Chicago, USA

**Keywords:** Molecular neuroscience, Clinical genetics

## Abstract

Schizophrenia, Schizoaffective, and Bipolar disorders share behavioral and phenomenological traits, intermediate phenotypes, and some associated genetic loci with pleiotropic effects. Volumetric abnormalities in brain structures are among the intermediate phenotypes consistently reported associated with these disorders. In order to examine the genetic underpinnings of these structural brain modifications, we performed genome-wide association analyses (GWAS) on 60 quantitative structural brain MRI phenotypes in a sample of 777 subjects (483 cases and 294 controls pooled together). Genotyping was performed with the Illumina PsychChip microarray, followed by imputation to the 1000 genomes multiethnic reference panel. Enlargement of the Temporal Horns of Lateral Ventricles (THLV) is associated with an intronic SNP of the gene NRXN1 (rs12467877, *P* = 6.76E–10), which accounts for 4.5% of the variance in size. Enlarged THLV is associated with psychosis in this sample, and with reduction of the hippocampus and enlargement of the choroid plexus and caudate. Eight other suggestively significant associations (*P* < 5.5E–8) were identified with THLV and 5 other brain structures. Although rare deletions of NRXN1 have been previously associated with psychosis, this is the first report of a common SNP variant of NRXN1 associated with enlargement of the THLV in psychosis.

## Introduction

There is ample evidence of partially overlapping brain morphology abnormalities in schizophrenia (SZ), schizoaffective disorder (SAD), and bipolar disorder (BD)^1–3^. These disorders also have a shared set of genetic loci associated with them which can generate diverse disease phenotypes (pleiotropism), and a partially shared polygenic diathesis^4,5^, as well as shared behavioral features, including psychosis in some BD patients. Although large samples of patients with psychotic mental disorders and controls have undergone GWAS, the phenotypes in these studies have been mostly restricted to diagnostic categories^4,6^, and most studies of intermediate phenotypes associated with psychosis reported are based on smaller samples^7–10^. Gottesman proposed the term endophenotype for sub-phenotypes observable in patients that might prove more tractable to genetic analysis^11,12^, and independently Gershon et al.^13,14^ proposed a similar idea. Following these concepts, the Bipolar and Schizophrenia Network for Intermediate Phenotypes study (B-SNIP) collected multiple phenotypes on each studied individual, aimed to identify biomarkers of psychosis and their genetic correlations, which can help elucidate mechanisms of the disorders, and lead to improved classifications and personalized treatment^15^. Previous neuroimaging studies found enlargement of the ventricular system in bipolar disorder and schizophrenia^16,17^, and the temporal (inferior) horns of the lateral ventricles (THLV) were also reported enlarged in patients with these disorders^18^. Here we present genome-wide association analyses of brain imaging in 777 B-SNIP patients with psychosis and healthy controls. Although this sample size is smaller than current large case-control studies, the power to detect associations using quantitative phenotypes is higher than with dichotomous traits, and pooling ill subjects with normal controls to analyze quantitative traits has the advantage of a wider range in the observed phenotypes, which translates into higher power to detect genetic associations^19^. As noted by Dahl et al.^20^, deep phenotyping with simultaneous genome-wide analyses can serve as a discovery tool for previously unsuspected relationships of phenotypic traits with each other, and with shared molecular events. In this study we found a significant association between the THLV volume and a common intronic SNP of the gene Neurexin 1 (NRXN1). NRXN1 is known to be involved in brain development and function. These facts support the potential role of this common variant of NRXN1 in psychosis.

## Methods

The Bipolar and Schizophrenia Network for Intermediate Phenotypes (B-SNIP) studies individuals with schizophrenia (SZ), schizoaffective disorder (SAD), bipolar disorder with psychosis (BD), and healthy controls. Data were collected between 2008 and 2012 at six study sites in the USA, with IRB approval at each participating institution. Details of phenotype collection are described in Tamminga el al.^21,22^, including standardization of phenotyping methods across all the collaborating sites. Here we present genome-wide association analyses of structural brain MRI data from 59 brain volumes plus the whole brain gray matter density, studied in 483 cases (169 BD, 127 SAD, and 187 SZ) and 294 healthy controls. Sample demographics are shown in Table 1. A total of 60 quantitative traits (listed in Supplementary Material) from these 777 cases and controls pooled together are analyzed here as phenotypes in genome-wide association studies (GWAS). Diagnostic categories were not used as phenotypes or as covariates in GWAS, except for post-hoc analyses to check on phenotypic associations with case-control status in this dataset, and whether our top SNP was associated with disease. The rationale to pool cases and controls is that in the study of quantitative traits when the phenotype is associated with disease there will be greater phenotypic variation and thus more power to detect association. These associations could help to explain part of the shared pathophysiology of these disorders. Including each diagnostic category as a covariate would ignore the genetic intercorrelation of the diagnoses, and detract from estimates of genetic associations with the tested phenotypes.

**Table 1:** Sample Demographics

		Age	Sex	Self-reported Ethnicity		
	*N*	Mean ± SD	Female *%*	Caucasian *%*	African-American *%*	Others *%*
Controls	294	38.19 ± 12.48	51.36	67.69	26.87	5.44
Cases						
SZ	187	34.99 ± 12.43	35.29	46.52	45.45	8.02
SAD	127	35.94 ± 11.87	55.12	52.76	41.73	5.51
BD	169	36.46 ± 13.13	69.23	76.92	19.53	3.55
All Cases	483	35.75 ± 12.53	52.38	58.80	35.40	5.80
Total Sample	777	36.67 ± 12.56	51.99	62.16	32.18	5.66

### Phenotypes: Image processing

Structural T1-weighted 3D magnetization-prepared rapid gradient echo (MPRAGE) scans were acquired at six sites: Boston (3.0 T, GE Signa), Detroit (3.0 T, Siemens Allegra), Baltimore (3.0 T, Siemens Trio trim), Hartford (3.0 T, Siemens Allegra), Dallas (3.0 T, Philips) and Chicago (3.0 T, GE Signa). The scans (TR = 6.7 msec, TE = 3.1 msec, 8° flip angle, 256 × 240 matrix size, total scan duration = 10:52.6 min, 170 sagittal slices, 1 mm slice thickness, 1 × 1 × 1.2 mm^3^ voxel resolution) were acquired following the Alzheimer’s Disease Neuroimaging Initiative (ADNI) protocol (http://adni.loni.usc.edu/methods/documents/mri-protocols/). Scans were visually assessed for artifacts and processed using Freesurfer 6.0 (https://surfer.nmr.mgh.harvard.edu/)^23^ using Scientific Linux 7.5. The scans went through first-level auto-reconstruction to undergo registration in standard space, motion correction and skull striping. Trained raters edited the images to remove dura, sinuses and blood vessels that could interfere with segmentation. Scans were run through second and third level auto-reconstruction to segment gray-white matter, reviewed by two independent raters and removed if motion interfered with segmentation. A total of 777 scans were retained for analysis and registered to the Desikan/Killiany atlas^24^. For this study we used only FreeSurfer volumetric variables, joining left and right hemisphere volumes into single bilateral variables. The whole brain gray matter density was calculated using Voxelbased Morphometry Toolbox for SPM8 as in Ivleva et al.^25^.

#### Genotypes

Genotypes were assessed from blood DNA at the Broad Institute using the Illumina Infinium Psycharray (PsychChip) (Illumina Inc., San Diego, CA, USA), which contains a total of 588,454 SNP markers, including 50,000 specific genetic markers for neuropsychiatric disorders. PsychChip genotype calls were processed through a custom pipeline at the Broad Institute designed to merge calls from 3 different algorithms (GenCall, Birdseed and zCall), in order to maximize reliability and usability of rare markers (https://sites.google.com/a/broadinstitute.org/psych-chip-resources/genotype-calling). We used programs PREST-plus^26^ and KING^27^ in order to remove individuals with 3rd degree or closer kinship. Pre-Imputation QC applied to PsychChip genotypes included filtering by Call Rate > 98% by SNP and > 98% by sample, HWE *P*-value > 1E–06 in controls, Inbreeding Coefficient (−0.2 > F_Het > 0.2), exclusion of monomorphic markers, Sex Check using X-chromosome heterozygosity and Y-chromosome call rate (all samples with sex check mismatch were dropped), and Minor Allele Frequency (MAF) > 0.01.

#### Genotype Imputation

PsychChip genotypes were imputed to the 1000 Genomes phase 1 multiethnic reference panel^28^ using IMPUTE2 and HAPI-UR for pre-phasing^29–31^. Chromosomes were phased separately, and then divided into 5 megabase pair chunks for imputation. Poorly imputed SNPs (those with information score < 0.5) were filtered out post-imputation. Imputed genotype probabilities with an uncertainty <0.1 were transformed to hard calls using PLINK 1.9. The final imputed genotype set contained 30 million autosomal markers, reduced to 4,322,238 variants after filtering for missingness by marker < 0.05, missingness by individual sample < 0.02, and MAF > 0.05, in order to work with common variants only, given the limited power to detect rare events with our sample size (see power analysis in Suppl. Material).

#### GWAS analyses

GWAS analyses were performed using PLINK 1.9 linear regression model^32,33^ on the Bionimbus protected cloud of the Open Science Data Cloud servers at the University of Chicago (http://www.opensciencedatacloud.org)^34^. Genetic locations refer to the human genome GRCh37/hg19 build, gene mapping was done using the UCSC genome browser^35^, and graphics generated with Manhattan Plotter, FUMA^36^ and LocusZoom^37^. Q–Q plots and calculated lambdas were used to examine possible inflation in GWAS results. We corrected for population stratification and admixture using the method of Price et al.^38^; we used the first two eigenvectors from principal component analysis on the genotypes as covariates, which captured the majority of the ethnic-related variance (see Supplementary Material). Sex and age were used as covariates in all our analyses, and the total intracranial volume was also used as a covariate except for the association with itself as phenotype. Covariates used for gray matter density GWAS were age, sex, handedness and two PCA eigenvectors from genotypes.

Post-GWAS eQTL and functional genomic analyses were performed on FUMA version 1.3.4 (K. Watanabe, http://fuma.ctglab.nl/)^36^, using data from The Genotype-Tissue Expression (GTEx) project version 7 (https://gtexportal.org)^39^ and the BrainSpan Atlas of the Developing Human Brain (http://www.brainspan.org/)^40^. The UK Brain Expression Consortium database (http:// http://www.braineac.org/)^41^ was also consulted for eQTLs of our top SNP markers.

#### Significance Thresholds

We calculated the GWAS statistical significance threshold for a single phenotype as *P* < 5.5E–08 using the Li et al. method based on independent LD blocks from our genotypes^42^. In the context of multiple phenotype analyses reported, this is our threshold for suggestive significance. Many of the studied phenotypes are correlated, and as Bonferroni correction assumes independence of the tests, using it would increase the type-II error. We followed the Li and Ji method based on spectral decomposition of the phenotype correlation matrix to calculate the effective number of independent phenotype tests^43^. Thus, our significance threshold after multiple test correction was 5.5E–08/27 independent effective tests = *P* < 2.04E–09.

#### Additional phenotypic associations

Phenotypic associations were tested using analysis of variance (ANOVA) and partial correlations, calculated with IBM SPSS Statistics version 24.

## Results

**Fig. 1:**
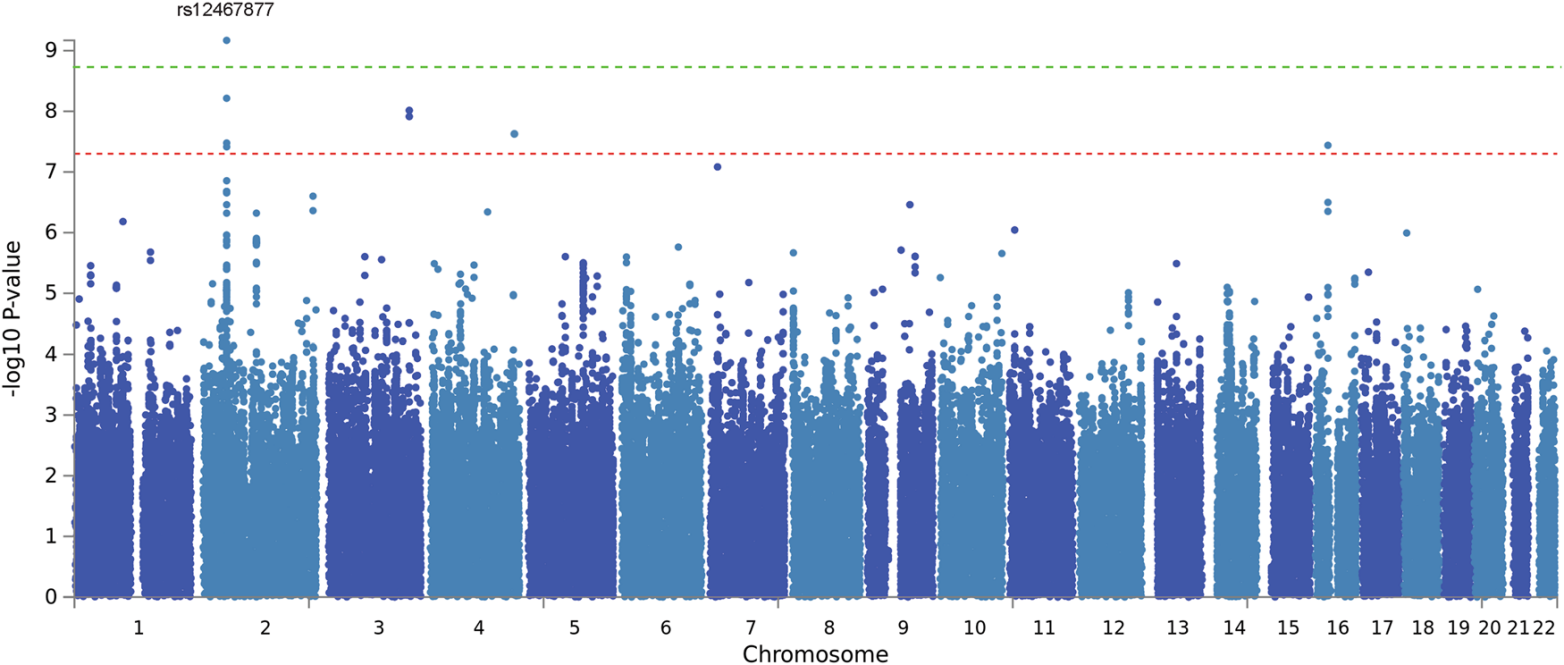
Genome-wide association of the volume of the temporal horn of the lateral ventricle in the B-SNIP sample. Green dotted line: significance threshold after multiple test correction (*P* < 2.04E–09). Red dotted line: suggestive significance threshold (*P* < 5.56E–8)

**Fig. 2:**
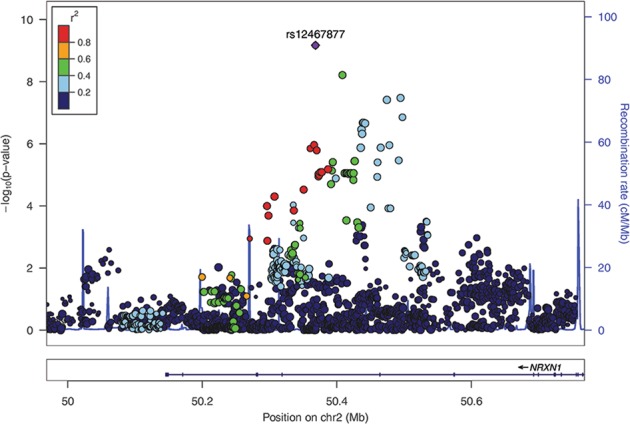
Regional association plot of lead variant: Regional association plot of 2p16.3 with the temporal horn of the lateral ventricle. Most significant associated SNP in violet

We identified a significant genome-wide association after multiple test correction between the combined volume of the temporal (inferior) horns of the lateral ventricles (THLV) and the T allele of rs12467877 (*P* = 6.76E–10), an intronic SNP of the gene Neurexin1 (*NRXN1*), see Table 2 and Figs. 1 and 2. The association between THLV and the NRXN1 allele was present in both of the two major ethnic groups of this sample (*P* = 8.195E–07 in Caucasians and *P* = 9.97E–05 in African-Americans) with no significant allelic frequency differences between them for rs12467877 (MAF 0.13 and 0.09 respectively, ANOVA *P* = 0.12, see Suppl. Material), which is consistent with reference data^44^. The T allele was associated with enlargement of THLV and its effect size on this phenotype was 0.045 (R^2^_G_), that is, 4.5% of the phenotype variance was attributable to this SNP. However, the identified NRXN1 SNP was not associated with case-control status (*P* = 0.21, using PLINK logistic regression model, controlling for sex and ethnicity).

**Table 2:** Genome-Wide Significant and Suggestive Association results

Phenotype	Chr	Band	Base Pair	SNP marker	Allele	Beta	*P*	Gene
Temporal Horn of Lateral Ventricle	**2**	**p16.3**	**50,368,229**	**rs12467877**	**T**	**184.9**	**6.76E–10**	**NRXN1**
Temporal Horn of Lateral Ventricle	3	q26.31	172,659,246	rs10440041	G	246.6	9.65E–09	SPATA16
Temporal Horn of Lateral Ventricle	4	q32.1	156,265,336	rs75174989	G	258.1	3.20E–08	MAP9
Temporal Horn of Lateral Ventricle	16	p12.1	27,890,361	chr16:27890361:I	TG	232.2	3.63E–08	GSG1L
Precentral	1	p36.32	5,337,271	rs61759358	A	−718.5	2.91E–08	intergenic
Pallidum	16	q23.2	80,215,561	rs9935652	G	109.6	4.08E–08	intergenic
Lateral Ventricle	3	p12.3	77,856,623	rs3852018	C	6836	4.22E–08	intergenic
Cortex	3	q21.3	128,650,296	chr3:128650296:I	GTA	11710	4.43E–08	KIAA1257
Isthmus Cingulate	22	q13.2	42,398,849	rs133310	C	−179.6	5.17E–08	WBP2NL

GWAS results that passed the suggestive significance threshold (*P* < 5.5E–08). **Bold font:** significant after multiple test correction (*P* < 2.04E–09).

### Other suggestive GWAS findings

Eight more genomic regions had P values lower than 5.5E−08 with 6 phenotypes; the top 3 were intronic SNPs of genes SPATA16, MAP9 and GSG1L associated with THLV (Table 2).

Precentral, Pallidum and Lateral Ventricle volumes had suggestive associations with intergenic SNPs. A variant in an uncharacterized protein, KIAA1257, was found to be suggestively associated with total cortical volume, and the gene WBP2NL, a sperm domain-binding protein previously associated with intelligence, was associated in our study with the volume of Isthmus Cingulate.

GWAS summary statistics for SNP associations with *P* < 1E–5 from the 60 brain morphology phenotypes studied are available in Supplementary Material.

### Phenotypic associations

In our sample we observed larger volumes of the temporal (inferior) horns of the lateral ventricles in cases vs. controls (ANOVA *P* = 0.015). Hippocampal volumes were smaller in cases than in controls (ANOVA *P* = 4E–6), and amygdala volumes were also significantly smaller in cases (*P* = 0.01), as reported in previous publications of our group^25,45,46^. Enlargement of THLV was correlated in our sample with volume reduction of the hippocampus and with enlargement of the choroid plexus and caudate (Table 3).

**Table 3:** Partial correlations of volumes of the combined temporal horns of lateral ventricles and surrounding structures

	Correlation	P
Caudate	**0.101**	**0.005**
Putamen	−0.068	0.059
Hippocampus	**−0.152**	**2.00E–05**
Amygdala	−0.052	0.15
Choroid plexus	**0.356**	**1.62E–24**

Partial correlations controlling for intracranial volume, age and sex. 2-tailed, 774 df. **Bold font**: *P* < 0.05.

## Discussion

Enlarged ventricular volumes have been among the most consistent anatomical alterations found in SZ and BD, first reported by Johnstone in schizophrenia more than forty years ago^47^. This has been confirmed in SZ and BD patients compared with healthy controls in large studies by the ENIGMA consortium, and the heritability of the volume of lateral ventricles was calculated to be 0.54 by Kremen et al.^48^. The temporal horn of the lateral ventricle traverses the temporal lobe in a lateral-anterior direction, bordering the caudate, putamen, amygdala, choroid plexus, and hippocampus. Increased ventricular volumes observed in schizophrenia have been attributed to volume reduction of surrounding gray matter structures^49,50^, which is confirmed in this study. Enlargement of choroid plexus in this sample was also associated with cognitive and structural connectivity problems in cases, published by Lizano et al.^51^.

The UK Biobank has published structural MRI GWAS results that contain SNP associations with the temporal horn^52^, which did not replicate our significant finding (UK biobank result for rs12467877 *P* = 0.095). Non-replication of our finding in the UK Biobank sample may be due to a negligible proportion of psychotic patients in their dataset. GWAS analysis of THLV in our controls (only) also gave a non-significant association for rs12467877 (*P* = 0.07). The Psychiatric Genomic Consortium (PGC) did not find association of case status with rs12467877 (*P* = 0.076) in their large case-control schizophrenia GWASs^4,53^, which indicates that the NRXN1 association is pertinent to the intermediate phenotype (THLV volume) and not directly with diagnosis.

NRXN1 encodes a large presynaptic transmembrane protein that binds neuroligins to calcium-dependent synaptic complexes in the central nervous system, and is thus involved in the formation of synaptic contacts^54^. This gene is highly intolerant to loss of function mutations, as described by the Exome Aggregation Consortium (ExAC), with a probability of Loss of function Intolerance, pLI = 1 (https://decipher.sanger.ac.uk/genes). Structural copy number variants (CNVs) disrupting NRXN1 are associated with a wide spectrum of brain disorders including schizophrenia, autism, and developmental disorders^55–57^. The UK Brain Expression Consortium (http://www.braineac.org/)^41^ has reported expression data for NRXN1 related to rs12467877, with no significant evidence for affecting expression (see Suppl. Material, page 10). The Genotype-Tissue Expression (GTEx) project (http://gtexportal.org) also does not report functional associations for rs12467877. Functional analyses using FUMA identified this and other suggestively associated SNPs to be related to the expression of other genes, such as PPP1R21 and GSG1L (Suppl. Material), which are highly expressed in brain during development and adulthood.

Three other genes suggestively associated with THLV are SPATA16, MAP9 and GSG1L. SPATA16 is involved in spermatogenesis and structural variants of this gene have been associated with globozoospermia^58^. MAP9 (also known as ASAP) is a gene encoding a microtubule associated protein highly expressed in brain and thyroid (https://gtexportal.org)^59^. The germ cell-specific gene 1-like protein (GSG1L) is a component of the AMPA receptor complex, a glutamate transmembrane receptor in the CNS, highly expressed in nucleus accumbens and basal ganglia, that has been associated with synaptic plasticity, mathematical ability, and creative activities in music^60–62^. Functional analysis of the GSG1L suggestively associated SNPs identified additional eQTLs (See Supplementary Material).

This is the first report of a common SNP variant of NRXN1 associated with enlargement of THLV volume in psychosis, and this volume is correlated with reduction of the hippocampus and enlargement of the caudate and choroid plexus. The association *p*-value 6.76E–10 is significant after multiple test correction, but further research with a larger sample of cases and controls will be needed to confirm these results. Although the role of this common NRXN1 SNP in psychosis is indirect, this study adds evidence to the role of NRXN1 in psychosis, which was originally implied by rare CNVs of this gene.

## Supplementary information

Supplementary Material

## Data Availability

Manhattan plots and GWAS summary statistics for SNP associations with *P* < 1E–5 from the 60 brain morphology phenotypes studied here are available in Supplementary Material. Complete GWAS summary statistics can be requested from the authors.
