# Supplementary material for: NRXN1 is associated with enlargement of the temporal horns of the lateral ventricles in psychosis
Source: Transl Psychiatry. 2019 Sep 17;9:230. doi: 10.1038/s41398-019-0564-9 (PMC6748921; doi:10.1038/s41398-019-0564-9)
Supplement: Supplementary file 1 — Supplementary Material [file 41398_2019_564_MOESM1_ESM.pdf]

SUPPLEMENTARY MATERIAL

Supplementary Material Index

1. Complete List of 60 Phenotypes used for GWAS (Page 3)

2. Supplementary Power Analysis (Page 4)

3. Principal Component Analysis of genotypes (Page 5)

4. Selected genotype and phenotype comparisons by ethnic groups (Page 6)

5. Functional genetic analysis (Page 7)

6. Manhattan plots and GWAS summary statistics for SNP associations with P<1E-5 from the 60 brain morphology phenotypes studied (Page 11)

7. References (Page 139)

1. Complete List of 60 Phenotypes used for GWAS

Volumes calculated with FreeSurfer 6.0. Parcellations and segmentations are based on Desikan et al. 2006(1), Destrieux et al. 2010(2) and Fischl et al. 2002(3). Gray matter density calculated with voxelbased morphometry toolbox for SPM8 (4) as in Ivleva et al. 2017 (5).

<u>CORTICAL PARCELLATIONS:</u>

Volume of the Banks of the Superior Temporal Sulcus
Volume of the Caudal anterior-cingulate cortex
Volume of the Caudal middle frontal gyrus
Volume of the Cuneus cortex
Volume of the Entorhinal cortex
Volume of the Fusiform gyrus
Volume of the Inferior parietal cortex
Volume of the Inferior temporal gyrus
Volume of the Isthmus – cingulate cortex
Volume of the Lateral occipital cortex
Volume of the Lateral orbital frontal cortex
Volume of the Lingual gyrus
Volume of the Medial orbital frontal cortex
Volume of the Middle temporal gyrus
Volume of the Parahippocampal gyrus
Volume of the Paracentral lobule
Volume of the Pars opercularis
Volume of the Pars orbitalis
Volume of the Pars triangularis
Volume of the Pericalcarine cortex
Volume of the Postcentral gyrus
Volume of the Posterior-cingulate cortex
Volume of the Precentral gyrus
Volume of the Precuneus cortex
Volume of the Rostral anterior cingulate cortex
Volume of the Rostral middle frontal gyrus
Volume of the Superior frontal gyrus
Volume of the Superior parietal cortex
Volume of the Superior temporal gyrus
Volume of the Supramarginal gyrus
Volume of the Frontal pole
Volume of the Temporal pole
Volume of the Transverse temporal cortex
Volume of the Insular cortex

<u>SUB-CORTICAL SEGMENTATIONS:</u>

Volume of the Thalamus
Volume of the Caudate
Volume of the Putamen
Volume of the Pallidum
Volume of the Hippocampus
Volume of the Amygdala
Volume of the Accumbens
Volume of the Ventral Diencephalon
Volume of the Choroid plexus
Volume of the Lateral Ventricles
Volume of the Temporal Horn of Lateral Ventricle
Volume of the Third Ventricle
Volume of the Fourth Ventricle
Volume of the Corpus Callosum Posterior
Volume of the Corpus Callosum Mid-Posterior
Volume of the Corpus Callosum Central
Volume of the Corpus Callosum Mid-Anterior
Volume of the Corpus Callosum Anterior

<u>GLOBAL BRAIN METRICS:</u>

Volume of the Cortex
Volume of the Cerebral White Matter
Volume of the Sub-Cortical Gray Matter
Total Gray Matter Volume
Whole Brain Gray Matter Density
Total Intracranial Volume

<u>CEREBELLUM:</u>

Volume of the Cerebellum White Matter
Volume of the Cerebellum cortex

2. Supplementary Power Analysis

We used QUANTO version 1.2 (Gauderman & Morrison 2007) (6, 7) in order to calculate the sample sizes required to achieve 80% power in genome-wide association for genetic markers with effect sizes (R^2_G) from 0.01 to 0.20, given our calculated significance thresholds (suggestive: P<5.5E-08 in blue; and significant: P<2.04E-09 in orange). We used real data from our results as parameters to plot the curves (phenotype mean=740; SD=397). Our significant NRXN1 SNP had an effect size R^2_G=0.045 (plotted red dot), calculated from its GWAS Beta linear regression coefficient.

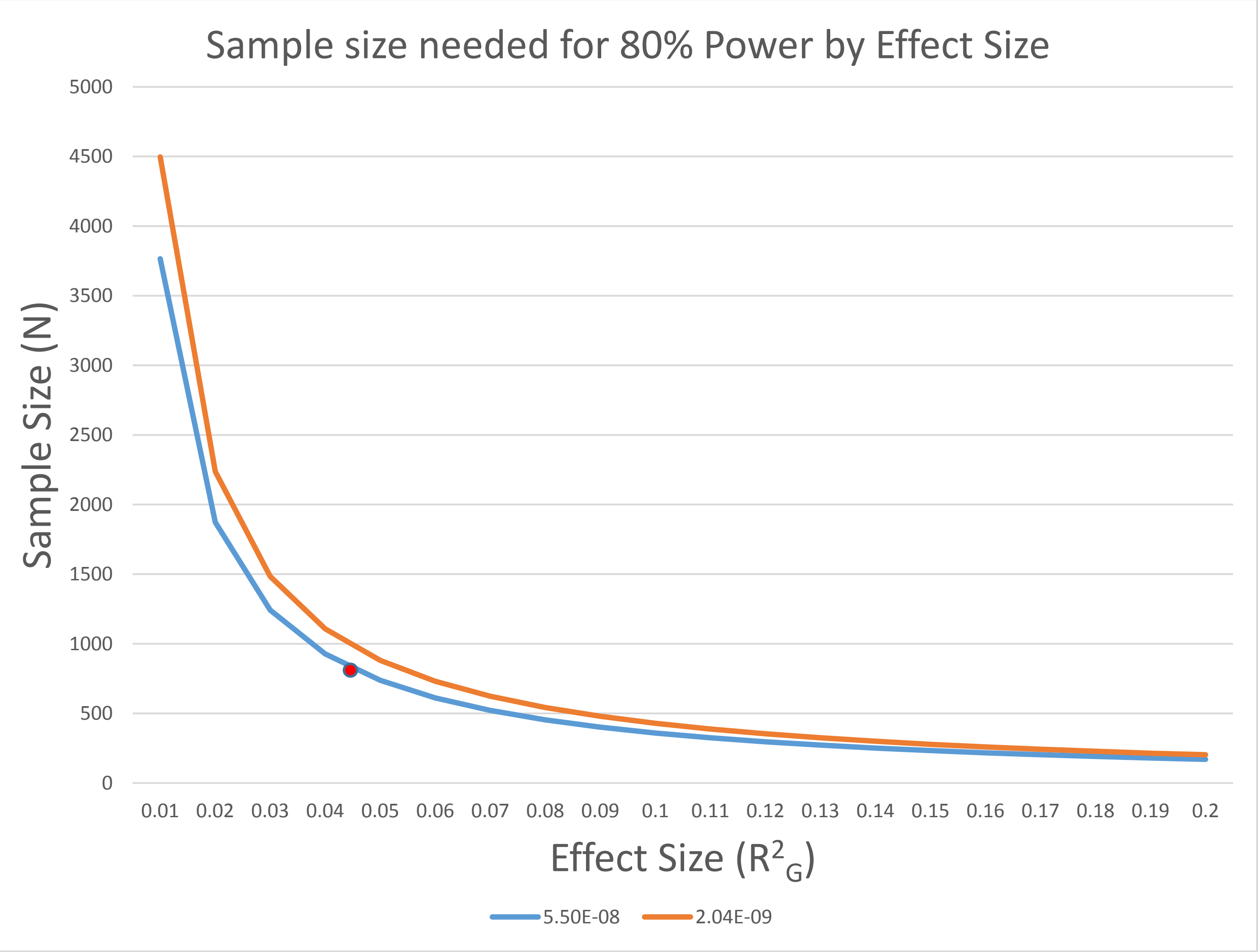

3.- Principal Component Analysis of genotypes:

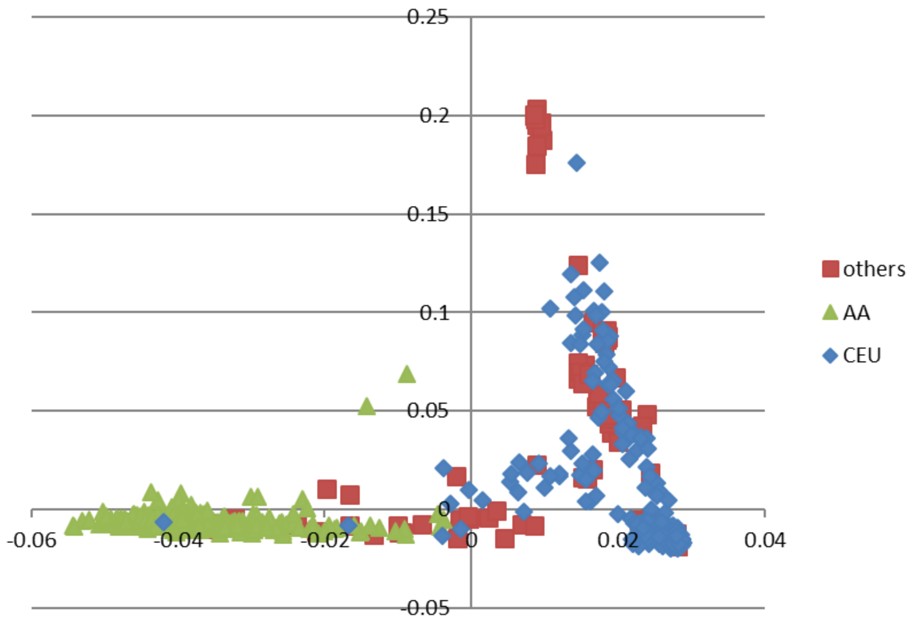

Genotype's PCA vs. Self-Reported ethnicity: Plot of the 2 first Eigenvectors from Principal Component Analysis (PCA) of genotypes: lower left corner cluster corresponds to African ancestry (AA), lower right corner cluster to Caucasian ancestry (CEU), and the upper middle to Asian ancestry component. Color code is self-reported ethnicity.

Genetic Variance Captured by PCA Eigenvectors:

Eigenvalues	Variance	Accumul.Variance
70.3865	0.6398	0.6398
8.0488	0.0732	0.7129
3.0345	0.0276	0.7405
2.2481	0.0204	0.7609
2.1078	0.0192	0.7801
1.9298	0.0175	0.7976
1.8580	0.0169	0.8145
1.7422	0.0158	0.8304
1.7104	0.0155	0.8459
1.6704	0.0152	0.8611
1.6234	0.0148	0.8759
1.5887	0.0144	0.8903
1.5692	0.0143	0.9046
1.5514	0.0141	0.9187
1.5363	0.0140	0.9326
1.5208	0.0138	0.9464
1.5054	0.0137	0.9601
1.4792	0.0134	0.9736
1.4608	0.0133	0.9869
1.4462	0.0131	1.0000

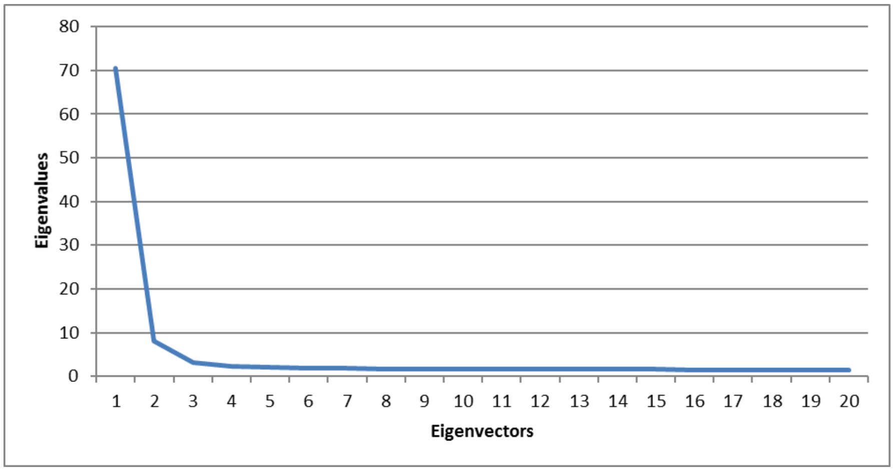

Plot of Eigenvalues for each Eigenvector from Genotypes' PCA. The two first Eigenvectors which captured the majority of variance were used as covariates for GWAS (as in Price 2006).

4. Selected genotype and phenotype comparisons by ethnic groups

No significant differences were observed in allelic frequencies of the NRXN1 top SNP allele among ethnic groups:

Risk allele frequencies vs. ethnic group (ANOVA)					
	Sum of Squares	df	Mean Square	F	P
rs12467877_T * Ethnic Group	0.89	2	0.45	2.12	0.12

However, there were significant differences in THLV volumes (reported in voxels) by ethnic groups.

**Ethnic Group**	**Mean**	**N**	**Std. Deviation**
AA	709.907	250	462.2173
CEU	766.486	483	364.6241
OTHER	616.711	44	299.4789
Total	739.800	777	397.1068

**ANOVA Table**						**Measures of Association**	
	Sum of Squares	df	Mean Square	F	P	Eta	Eta Squared
THLV volume * Ethnic Group	1233982.790	2	616991.395	3.942	.020	.100	.010

5. Functional genetic analysis:

Functional analyses using FUMA (K. Watanabe, http://fuma.ctglab.nl) (8) to our THLV GWAS results identified additional genes via eQTLs and chromatin interactions.

<u>Circos plot of Chromosome 2</u>: (THLV significant association region with NRXN1 is highlighted in blue)

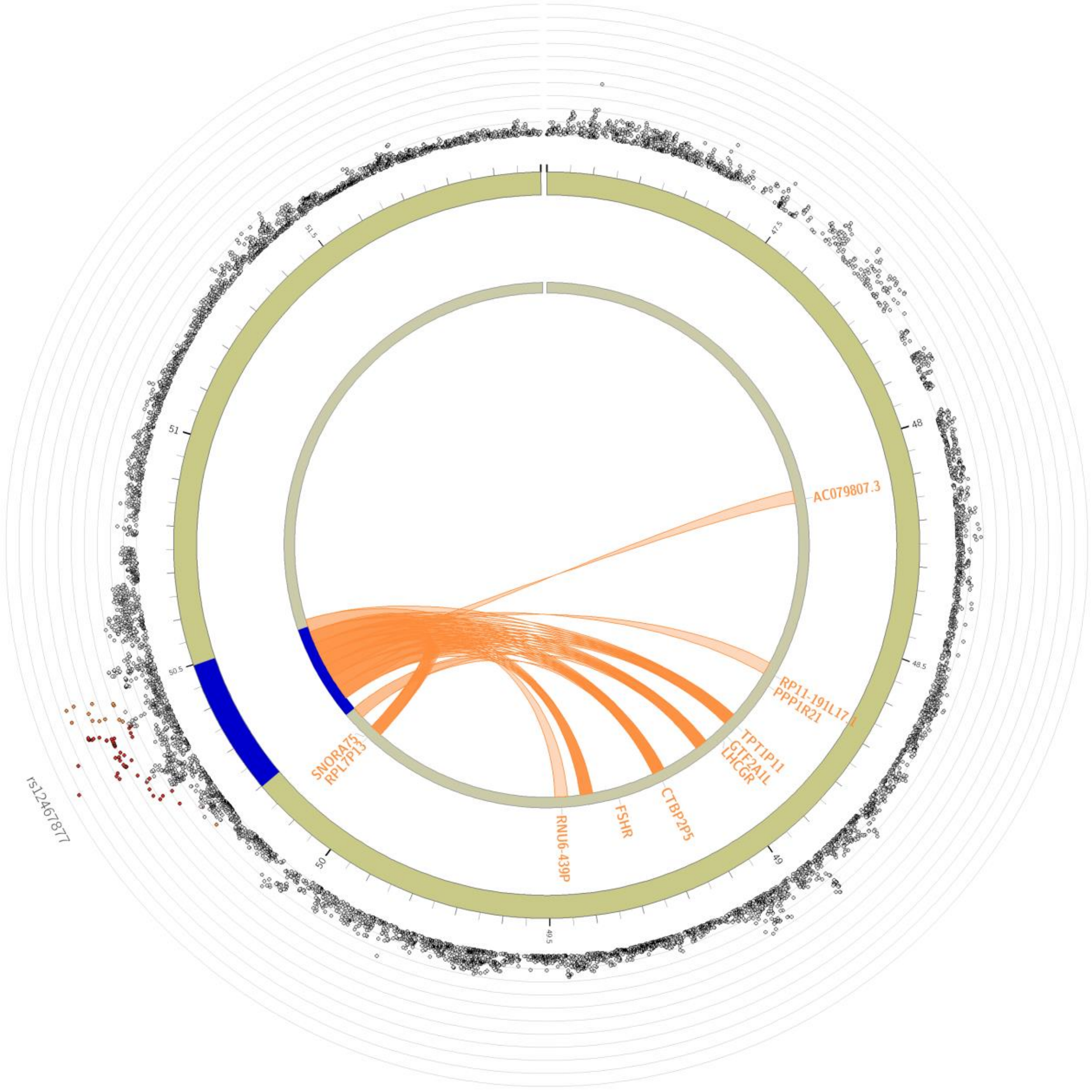

Circos plot of chromosome 16: (suggestive-significant association of THLV with SNPs of gene GSGIL)

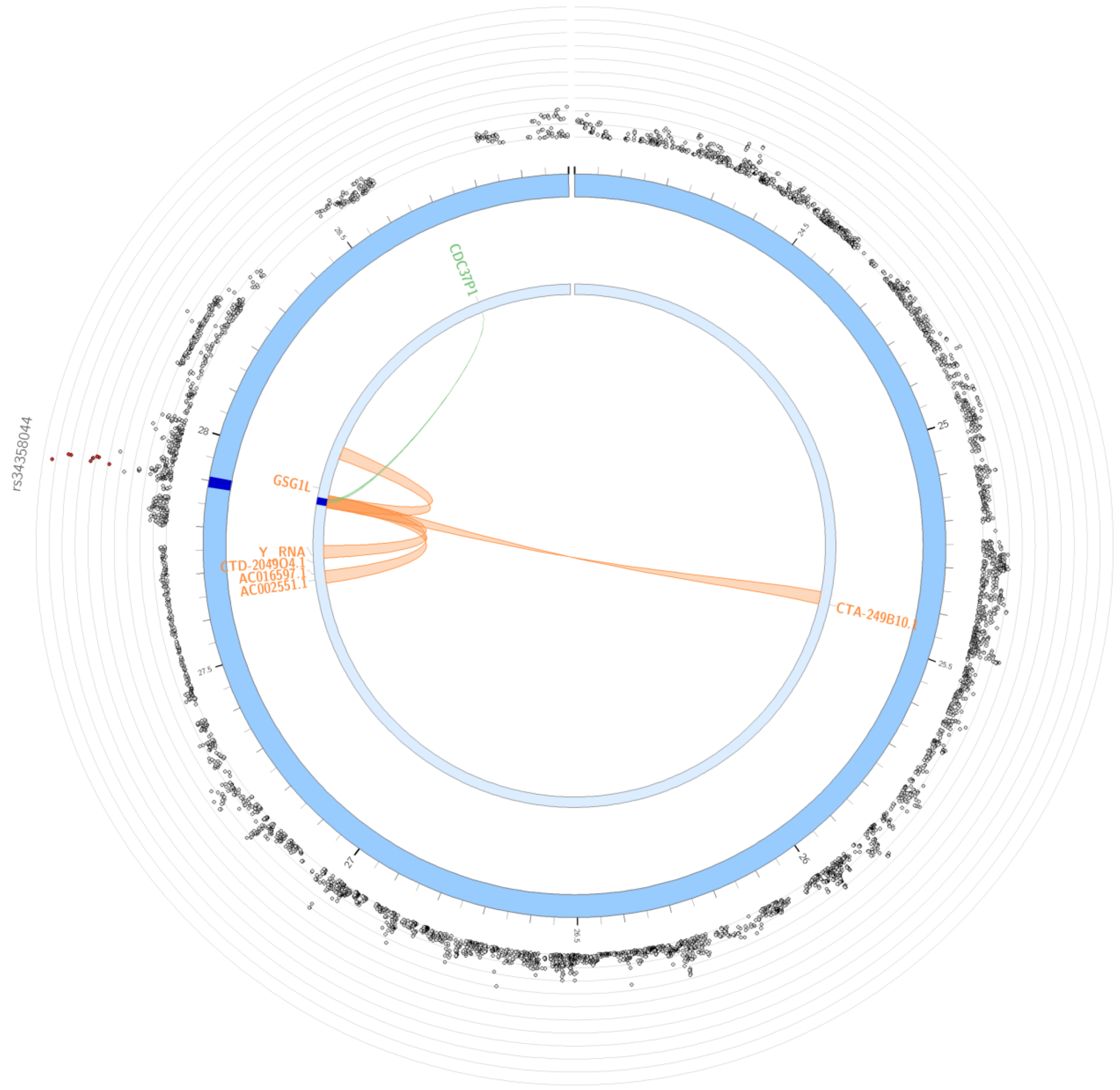

Circos Plots: the outer ring is a Manhattan plot, with SNPs in genomic risk loci color-coded as a function of their maximum r^2 to the one of the independent significant SNPs in the locus, as follows: red ($r^2 > 0.8$), orange ($r^2 > 0.6$), green ($r^2 > 0.4$) and blue ($r^2 > 0.2$). SNPs that are not in LD with any of the independent significat SNPs (with $r^2 \le 0.2$) are grey. The rsID of the top SNPs in each risk locus are displayed in the most outer layer. Y-axis are raned between 0 to the maximum -log10(P-value) of the SNPs. Chromosome ring: The second layer. Genomic risk loci are highlighted in blue. Mapped genes by chromatin interactions are colored orange in the inner ring, and eQTLs are green.

Brain Expression Heatmap:

The BrainSpan 11 (9) heatmap shows that NRXN1, GSGIL, and other genes with functional relationship to SNPS of these two genes, as shown in the Circos plots above, are expressed in brain during development and adulthood:

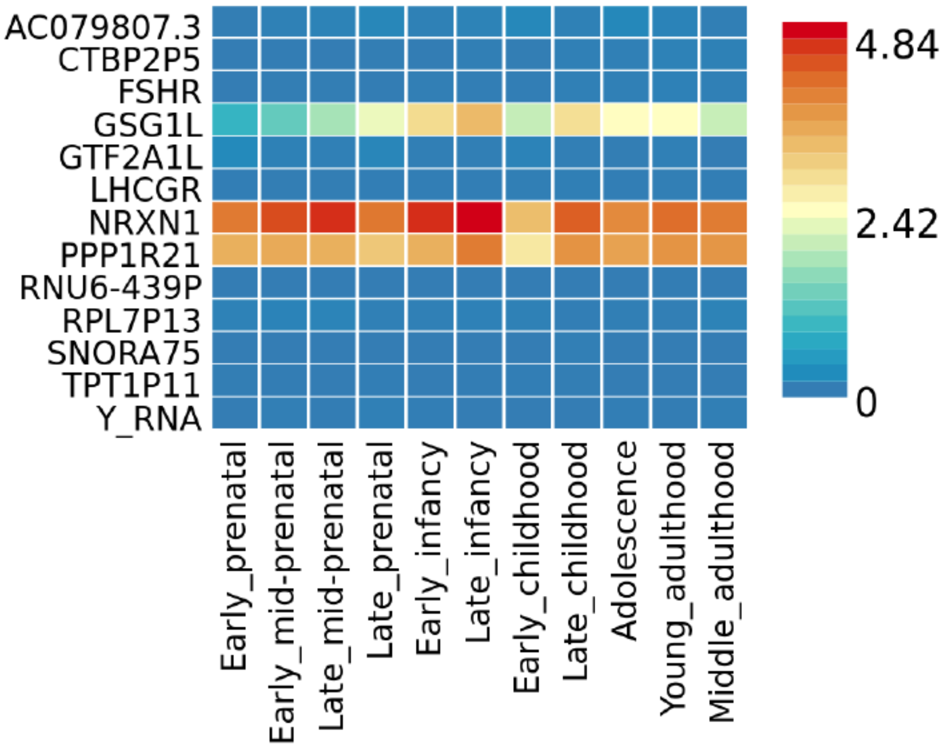

UK Brain Expression Consortium (www.braineac.org) eQTL data for rs12467877:

gene	marker	rsid	exprID	chr	start	stop	Average Probe	CRBL	FCTX	HIPP	MEDU	OCTX	PUTM	SNIG	TCTX	THAL	WHMT
**NRXN1**	chr2:50368229	rs12467877	2552834	chr2	50145654	51271272	5.80E-02	7.60E-01	6.90E-01	6.10E-01	3.20E-01	7.40E-01	6.80E-01	5.80E-01	3.50E-02	5.10E-02	9.30E-01
**NRXN1**	chr2:50368229	rs12467877	2552665	chr2	50145654	51271272	1.50E-01	3.30E-01	2.60E-02	5.40E-01	9.00E-01	4.70E-01	1.40E-01	4.00E-01	1.30E-02	3.30E-01	8.90E-01
**NRXN1**	chr2:50368229	rs12467877	2552735	chr2	50145654	51271272	1.60E-01	5.30E-01	7.80E-02	9.80E-01	8.00E-01	4.40E-01	3.60E-02	2.00E-01	5.30E-02	6.60E-01	7.20E-01
**NRXN1**	chr2:50368229	rs12467877	2552644	chr2	50145654	51271272	1.80E-01	1.30E-01	4.90E-01	4.10E-01	2.20E-01	1.80E-01	6.30E-01	5.90E-01	3.50E-01	5.30E-02	6.70E-01
**NRXN1**	chr2:50368229	rs12467877	2552686	chr2	50145654	51271272	1.80E-01	6.50E-01	4.10E-01	1.30E-01	7.00E-01	4.50E-01	1.50E-01	7.10E-01	2.20E-01	2.50E-01	8.10E-01
**NRXN1**	chr2:50368229	rs12467877	2552835	chr2	50145654	51271272	1.90E-01	8.60E-01	2.50E-01	1.70E-01	6.10E-01	7.00E-01	5.30E-01	4.40E-01	1.40E-01	5.70E-01	6.20E-01
**NRXN1**	chr2:50368229	rs12467877	2552873	chr2	50145654	51271272	2.20E-01	1.60E-01	9.60E-01	1.50E-01	7.10E-01	4.20E-01	1.60E-01	9.50E-01	3.50E-01	1.10E-02	1.90E-01
**NRXN1**	chr2:50368229	rs12467877	2552838	chr2	50145654	51271272	2.30E-01	6.30E-01	3.10E-01	9.60E-02	7.80E-01	5.60E-01	4.50E-01	3.00E-01	4.30E-01	4.50E-01	6.00E-01
**NRXN1**	chr2:50368229	rs12467877	t2552643	chr2	50145654	51271272	2.60E-01	8.40E-01	2.00E-01	1.80E-01	2.80E-01	6.20E-01	1.00E-01	4.80E-01	2.50E-01	2.60E-01	8.70E-01
**NRXN1**	chr2:50368229	rs12467877	2552804	chr2	50145654	51271272	2.60E-01	4.30E-01	8.20E-01	2.80E-01	4.30E-01	4.00E-01	1.80E-01	3.90E-01	8.00E-01	1.90E-01	7.10E-01
**NRXN1**	chr2:50368229	rs12467877	2552647	chr2	50145654	51271272	2.70E-01	7.10E-01	3.80E-02	7.10E-01	5.40E-01	7.50E-01	4.20E-02	5.30E-01	2.90E-01	1.60E-01	8.40E-01
**NRXN1**	chr2:50368229	rs12467877	2552806	chr2	50145654	51271272	2.70E-01	1.00E+00	3.40E-01	1.50E-01	5.60E-01	5.00E-01	1.50E-01	7.30E-01	1.40E-01	3.60E-01	8.40E-01
**NRXN1**	chr2:50368229	rs12467877	2552718	chr2	50145654	51271272	2.80E-01	5.90E-01	1.90E-01	2.10E-01	5.70E-01	6.00E-01	2.30E-01	7.20E-01	2.80E-01	1.20E-01	8.80E-01
**NRXN1**	chr2:50368229	rs12467877	2552656	chr2	50145654	51271272	3.20E-01	9.90E-01	2.60E-01	6.10E-01	7.60E-01	3.90E-01	1.90E-02	2.40E-02	1.10E-01	6.80E-01	2.10E-01
**NRXN1**	chr2:50368229	rs12467877	2552788	chr2	50145654	51271272	3.20E-01	5.90E-01	3.70E-01	7.20E-02	8.70E-01	8.40E-01	1.20E-01	6.20E-01	3.60E-01	4.00E-01	7.90E-01
**NRXN1**	chr2:50368229	rs12467877	2552866	chr2	50145654	51271272	3.20E-01	8.50E-01	5.20E-01	1.60E-01	7.00E-01	4.90E-01	8.60E-01	7.30E-01	1.60E-01	5.30E-01	1.80E-01
**NRXN1**	chr2:50368229	rs12467877	2552785	chr2	50145654	51271272	3.30E-01	8.40E-01	5.10E-01	3.00E-02	3.90E-01	3.80E-01	7.60E-02	6.00E-01	7.30E-01	4.60E-01	8.90E-01
**NRXN1**	chr2:50368229	rs12467877	2552709	chr2	50145654	51271272	3.40E-01	8.30E-01	9.50E-01	4.50E-01	7.50E-02	2.90E-01	3.90E-01	6.20E-01	2.20E-01	4.20E-01	3.20E-01
**NRXN1**	chr2:50368229	rs12467877	2552864	chr2	50145654	51271272	3.40E-01	9.60E-01	6.80E-01	2.50E-01	3.80E-01	7.90E-01	1.60E-01	9.10E-02	8.90E-01	2.20E-01	2.70E-01
**NRXN1**	chr2:50368229	rs12467877	2552645	chr2	50145654	51271272	3.40E-01	4.40E-01	4.40E-01	7.40E-01	7.10E-01	7.90E-01	4.90E-01	9.40E-01	5.10E-01	2.40E-01	9.40E-01
**NRXN1**	chr2:50368229	rs12467877	2552789	chr2	50145654	51271272	3.60E-01	3.30E-01	1.20E-01	3.00E-01	1.50E-01	7.10E-01	2.30E-01	9.00E-01	2.40E-02	3.40E-01	3.60E-01
**NRXN1**	chr2:50368229	rs12467877	2552695	chr2	50145654	51271272	3.70E-01	9.00E-01	2.50E-01	3.60E-01	3.50E-01	2.70E-01	3.90E-01	5.80E-01	1.90E-01	4.20E-01	7.80E-01
**NRXN1**	chr2:50368229	rs12467877	2552649	chr2	50145654	51271272	4.10E-01	8.20E-01	1.70E-01	8.20E-01	4.10E-01	4.40E-01	9.00E-02	5.40E-01	3.70E-01	3.50E-01	8.90E-01
**NRXN1**	chr2:50368229	rs12467877	2552807	chr2	50145654	51271272	4.10E-01	6.70E-01	1.90E-01	1.70E-01	3.30E-01	4.80E-01	1.60E-01	9.10E-01	2.20E-01	4.80E-01	7.00E-01
**NRXN1**	chr2:50368229	rs12467877	2552868	chr2	50145654	51271272	4.20E-01	7.30E-01	1.30E-01	3.70E-01	2.80E-01	8.40E-01	3.50E-01	4.30E-01	4.30E-01	1.90E-01	8.40E-01
**NRXN1**	chr2:50368229	rs12467877	2552802	chr2	50145654	51271272	4.30E-01	4.10E-01	6.10E-01	2.30E-03	1.70E-01	9.80E-01	5.20E-01	8.70E-01	6.60E-01	9.20E-01	5.20E-01
**NRXN1**	chr2:50368229	rs12467877	2552870	chr2	50145654	51271272	4.30E-01	8.20E-01	4.60E-01	9.70E-01	7.50E-02	3.30E-01	3.10E-01	2.50E-01	7.70E-01	1.40E-01	3.20E-01
**NRXN1**	chr2:50368229	rs12467877	2552754	chr2	50145654	51271272	4.30E-01	9.00E-01	1.80E-01	1.10E-01	3.90E-01	7.40E-01	4.60E-01	7.60E-01	5.00E-01	3.50E-01	8.20E-01
**NRXN1**	chr2:50368229	rs12467877	2552811	chr2	50145654	51271272	4.30E-01	9.30E-01	2.80E-01	4.70E-01	3.20E-01	4.30E-01	1.40E-01	4.60E-01	6.90E-01	4.50E-01	9.30E-01
**NRXN1**	chr2:50368229	rs12467877	2552865	chr2	50145654	51271272	4.50E-01	6.80E-01	9.20E-01	7.80E-01	2.90E-01	7.70E-01	2.90E-01	3.90E-02	3.20E-01	3.10E-01	3.20E-01
**NRXN1**	chr2:50368229	rs12467877	2552741	chr2	50145654	51271272	4.50E-01	5.60E-01	1.80E-01	9.10E-01	4.60E-01	3.00E-01	7.80E-01	6.70E-01	9.90E-01	6.50E-01	1.80E-01
**NRXN1**	chr2:50368229	rs12467877	2552740	chr2	50145654	51271272	4.60E-01	5.10E-01	4.10E-01	7.30E-01	8.20E-01	2.90E-01	9.20E-01	5.90E-01	7.80E-02	6.80E-01	6.80E-01
**NRXN1**	chr2:50368229	rs12467877	2552648	chr2	50145654	51271272	4.80E-01	1.80E-01	3.80E-01	6.80E-01	8.00E-01	3.20E-01	9.20E-01	3.60E-01	3.40E-02	4.60E-01	3.70E-01
**NRXN1**	chr2:50368229	rs12467877	2552646	chr2	50145654	51271272	4.90E-01	8.80E-01	1.30E-01	6.00E-01	3.20E-01	4.60E-01	3.00E-01	4.30E-01	3.60E-01	3.90E-01	8.80E-01
**NRXN1**	chr2:50368229	rs12467877	2552782	chr2	50145654	51271272	5.10E-01	9.40E-01	1.10E-01	9.00E-02	2.10E-01	5.30E-01	3.00E-01	9.00E-01	5.60E-01	4.90E-01	6.50E-01
**NRXN1**	chr2:50368229	rs12467877	2552708	chr2	50145654	51271272	5.60E-01	3.20E-02	5.50E-01	2.10E-01	6.80E-01	4.60E-01	5.70E-01	6.50E-01	9.10E-01	5.60E-01	5.00E-01
**NRXN1**	chr2:50368229	rs12467877	2552833	chr2	50145654	51271272	5.60E-01	9.10E-01	4.90E-02	7.80E-01	1.00E-01	6.60E-01	8.10E-01	9.40E-01	4.20E-01	2.50E-01	9.30E-01
**NRXN1**	chr2:50368229	rs12467877	2552781	chr2	50145654	51271272	5.60E-01	8.40E-01	3.40E-01	1.40E-01	1.80E-01	7.00E-01	2.10E-01	6.70E-01	5.40E-01	4.30E-01	6.30E-01
**NRXN1**	chr2:50368229	rs12467877	2552758	chr2	50145654	51271272	5.80E-01	5.90E-01	5.90E-01	1.60E-01	2.70E-01	8.80E-01	1.00E-01	9.20E-01	6.80E-01	3.90E-01	9.10E-01
**NRXN1**	chr2:50368229	rs12467877	2552739	chr2	50145654	51271272	6.30E-01	7.50E-01	5.60E-01	5.80E-01	3.10E-01	8.20E-01	3.80E-01	2.80E-01	2.30E-01	4.20E-01	9.50E-01
**NRXN1**	chr2:50368229	rs12467877	2552816	chr2	50145654	51271272	6.40E-01	9.90E-02	7.40E-01	9.00E-02	9.90E-01	1.20E-01	8.80E-01	5.70E-02	1.70E-01	9.00E-01	9.00E-01
**NRXN1**	chr2:50368229	rs12467877	2552769	chr2	50145654	51271272	6.40E-01	8.20E-01	7.60E-01	1.10E-01	2.20E-01	6.90E-01	1.10E-01	8.30E-01	9.00E-01	4.20E-01	9.40E-01
**NRXN1**	chr2:50368229	rs12467877	2552765	chr2	50145654	51271272	6.90E-01	8.20E-01	3.40E-01	3.00E-01	2.60E-01	9.20E-01	1.20E-01	7.30E-01	9.40E-01	3.90E-01	9.90E-01
**NRXN1**	chr2:50368229	rs12467877	2552863	chr2	50145654	51271272	8.30E-01	5.20E-01	8.60E-01	7.60E-01	7.80E-03	9.30E-01	7.10E-01	3.60E-01	3.80E-01	2.60E-01	6.10E-01
**NRXN1**	chr2:50368229	rs12467877	2552687	chr2	50145654	51271272	8.60E-01	5.10E-01	5.00E-01	3.50E-01	1.50E-01	9.10E-01	7.00E-01	4.80E-01	8.20E-01	4.50E-01	3.80E-01
**NRXN1**	chr2:50368229	rs12467877	2552872	chr2	50145654	51271272	8.80E-01	5.50E-01	1.60E-01	1.30E-01	7.60E-01	2.80E-01	3.80E-01	7.40E-01	3.60E-01	6.50E-01	5.00E-01
**NRXN1**	chr2:50368229	rs12467877	2552871	chr2	50145654	51271272	9.30E-01	5.20E-01	2.50E-01	9.20E-01	4.90E-01	1.90E-01	1.10E-02	9.20E-01	2.90E-01	3.70E-01	4.20E-01
**NRXN1**	chr2:50368229	rs12467877	2552766	chr2	50145654	51271272	9.90E-01	4.40E-01	3.40E-01	2.00E-01	3.10E-01	8.80E-01	4.10E-01	7.70E-01	9.70E-01	4.10E-01	7.00E-01
						**Average by Tissue:**	**4.35E-01**	**6.41E-01**	**3.98E-01**	**3.96E-01**	**4.46E-01**	**5.65E-01**	**3.57E-01**	**5.77E-01**	**4.20E-01**	**3.94E-01**	**6.62E-01**
**FSHR**	chr2:50368229	rs12467877	2552510	chr2	49189652	49389969	2.80E-02	9.00E-02	8.70E-04	9.50E-01	3.30E-01	3.10E-01	9.30E-01	4.50E-01	1.10E-02	3.20E-01	8.80E-01
**FSHR**	chr2:50368229	rs12467877	2552477	chr2	49189652	49389969	1.40E-01	4.20E-01	2.10E-01	8.30E-01	1.30E-01	2.30E-01	1.20E-01	8.70E-01	3.10E-01	4.60E-01	4.20E-01
**FSHR**	chr2:50368229	rs12467877	2552482	chr2	49189652	49389969	2.90E-01	6.40E-01	1.80E-02	6.50E-01	4.50E-01	6.50E-01	8.30E-01	9.00E-03	6.20E-02	4.70E-01	5.90E-01
**FSHR**	chr2:50368229	rs12467877	2552471	chr2	49189652	49389969	3.10E-01	6.20E-01	6.80E-01	4.00E-01	5.00E-01	5.90E-01	3.20E-01	6.70E-02	6.60E-01	3.00E-01	8.90E-01
**FSHR**	chr2:50368229	rs12467877	2552497	chr2	49189652	49389969	3.30E-01	5.70E-01	4.10E-01	2.50E-01	6.50E-01	5.40E-01	8.30E-03	7.10E-01	8.50E-03	4.90E-01	5.20E-02
**FSHR**	chr2:50368229	rs12467877	2552483	chr2	49189652	49389969	3.90E-01	9.70E-01	7.40E-01	3.20E-01	6.50E-01	5.40E-01	1.00E+00	8.70E-01	5.50E-01	8.40E-01	8.70E-01
**FSHR**	chr2:50368229	rs12467877	2552488	chr2	49189652	49389969	4.80E-01	6.70E-01	8.50E-01	1.30E-01	2.10E-01	7.00E-01	3.70E-01	6.20E-02	4.50E-02	6.90E-01	9.00E-01
**FSHR**	chr2:50368229	rs12467877	t2552470	chr2	49189652	49389969	5.10E-01	3.90E-01	5.50E-01	6.70E-01	6.80E-01	6.90E-01	4.70E-01	3.50E-02	7.30E-02	3.30E-01	6.30E-01
**FSHR**	chr2:50368229	rs12467877	2552474	chr2	49189652	49389969	5.40E-01	4.00E-01	1.10E-01	1.80E-01	5.30E-01	2.90E-01	8.40E-01	1.50E-01	9.70E-01	1.40E-01	8.60E-01
**FSHR**	chr2:50368229	rs12467877	2552472	chr2	49189652	49389969	5.40E-01	1.20E-01	6.70E-01	5.10E-01	6.60E-01	7.60E-01	9.60E-01	2.30E-01	7.60E-01	9.70E-01	9.20E-01
**FSHR**	chr2:50368229	rs12467877	2552478	chr2	49189652	49389969	5.50E-01	3.40E-01	4.50E-01	2.50E-01	3.60E-01	3.70E-01	4.50E-01	1.40E-01	3.50E-01	3.50E-01	1.40E-02
**FSHR**	chr2:50368229	rs12467877	2552489	chr2	49189652	49389969	7.40E-01	8.70E-01	4.20E-01	7.70E-01	8.20E-01	4.90E-01	8.80E-01	7.30E-02	1.50E-01	2.50E-01	2.40E-01
**FSHR**	chr2:50368229	rs12467877	2552511	chr2	49189652	49389969	8.60E-01	7.80E-01	3.70E-01	2.60E-01	9.40E-02	3.00E-02	7.80E-01	8.30E-01	3.70E-01	8.10E-01	6.30E-01
**FSHR**	chr2:50368229	rs12467877	2552512	chr2	49189652	49389969	1.00E+00	1.30E-01	4.00E-01	7.10E-01	8.20E-01	1.50E-01	8.30E-01	1.70E-01	5.90E-01	1.00E-01	7.80E-01
						**Average by Tissue:**	**4.79E-01**	**5.01E-01**	**4.20E-01**	**4.91E-01**	**4.92E-01**	**4.53E-01**	**6.28E-01**	**3.33E-01**	**3.51E-01**	**4.66E-01**	**6.20E-01**
**LOC730100**	chr2:50368229	rs12467877	2481963	chr2	51259749	52635045	1.40E-01	2.40E-01	7.80E-02	1.70E-01	9.10E-01	2.10E-01	6.40E-01	2.50E-01	9.40E-01	6.10E-01	2.40E-01
**LOC730100**	chr2:50368229	rs12467877	2481984	chr2	51259749	52635045	2.20E-01	7.10E-02	4.00E-01	4.10E-01	6.40E-01	8.80E-01	1.00E+00	8.40E-01	9.40E-01	9.90E-01	7.60E-01
**LOC730100**	chr2:50368229	rs12467877	2481983	chr2	51259749	52635045	3.40E-01	3.80E-01	6.40E-01	6.00E-01	1.40E-01	2.60E-01	7.80E-01	3.80E-01	1.50E-01	8.70E-01	9.80E-01
**LOC730100**	chr2:50368229	rs12467877	2481934	chr2	51259749	52635045	4.30E-01	9.70E-01	4.20E-01	7.90E-01	6.30E-01	9.10E-01	3.10E-01	5.50E-01	9.40E-01	1.80E-01	6.70E-01
**LOC730100**	chr2:50368229	rs12467877	2481975	chr2	51259749	52635045	5.20E-01	5.30E-01	5.80E-01	6.80E-01	4.50E-01	7.30E-02	5.90E-01	6.60E-01	5.90E-01	6.60E-02	7.60E-01
**LOC730100**	chr2:50368229	rs12467877	t2481929	chr2	51259749	52635045	5.70E-01	3.90E-01	6.10E-01	8.70E-01	1.20E-01	1.30E-01	5.50E-01	8.20E-01	1.00E-01	8.60E-01	1.70E-01
**LOC730100**	chr2:50368229	rs12467877	2481959	chr2	51259749	52635045	6.70E-01	7.60E-01	1.40E-01	1.00E+00	1.40E-01	4.00E-01	4.50E-01	9.40E-01	1.30E-01	8.60E-01	8.20E-01
**LOC730100**	chr2:50368229	rs12467877	2481989	chr2	51259749	52635045	9.80E-01	7.80E-02	8.00E-01	1.50E-01	1.50E-01	8.00E-03	1.70E-01	7.20E-01	1.10E-01	1.90E-01	1.20E-01
**LOC730100**	chr2:50368229	rs12467877	2481930	chr2	51259749	52635045	9.80E-01	3.00E-01	7.20E-01	7.10E-01	9.80E-01	1.20E-01	4.80E-01	1.60E-02	8.80E-02	9.50E-02	4.70E-01
						**Average by Tissue:**	**5.39E-01**	**4.13E-01**	**4.88E-01**	**5.98E-01**	**4.62E-01**	**3.32E-01**	**5.52E-01**	**5.75E-01**	**4.43E-01**	**5.25E-01**	**5.54E-01**

Brain regions: cerebellar cortex (CRBL), frontal cortex (FCTX), hippocampus (HIPP), medulla (specifically inferior olivary nucleus, MEDU), occipital cortex (specifically primary visual cortex, OCTX), putamen (PUTM), substantia nigra (SNIG), thalamus (THAL), temporal cortex (TCTX) and intralobular white matter (WHMT). Highlighted in green P < 0.05.

6.- Manhattan plots and GWAS summary statistics for SNP associations with P<1E-5 from the 60 brain morphology phenotypes studied

Use CLICK on this index to go directly to the desired result page

CORTICAL PARCELLATIONS:

Volume of the Banks of the Superior Temporal Sulcus
Volume of the Caudal anterior-cingulate cortex
Volume of the Caudal middle frontal gyrus
Volume of the Cuneus cortex
Volume of the Entorhinal cortex
Volume of the Fusiform gyrus
Volume of the Inferior parietal cortex
Volume of the Inferior temporal gyrus
Volume of the Isthmus – cingulate cortex
Volume of the Lateral occipital cortex
Volume of the Lateral orbital frontal cortex
Volume of the Lingual gyrus
Volume of the Medial orbital frontal cortex
Volume of the Middle temporal gyrus
Volume of the Parahippocampal gyrus
Volume of the Paracentral lobule
Volume of the Pars opercularis
Volume of the Pars orbitalis
Volume of the Pars triangularis
Volume of the Pericalcarine cortex
Volume of the Postcentral gyrus
Volume of the Posterior-cingulate cortex
Volume of the Precentral gyrus
Volume of the Precuneus cortex
Volume of the Rostral anterior cingulate cortex
Volume of the Rostral middle frontal gyrus
Volume of the Superior frontal gyrus
Volume of the Superior parietal cortex
Volume of the Superior temporal gyrus
Volume of the Supramarginal gyrus
Volume of the Frontal pole
Volume of the Temporal pole
Volume of the Transverse temporal cortex
Volume of the Insular cortex

SUB-CORTICAL SEGMENTATIONS:
Volume of the Thalamus
Volume of the Caudate
Volume of the Putamen
Volume of the Pallidum
Volume of the Hippocampus
Volume of the Amygdala
Volume of the Accumbens
Volume of the Ventral Diencephalon
Volume of the Choroid plexus
Volume of the Lateral Ventricles
Volume of the Temporal Horn of Lateral Ventricle
Volume of the Third Ventricle
Volume of the Fourth Ventricle
Volume of the Corpus Callosum Posterior
Volume of the Corpus Callosum Mid-Posterior
Volume of the Corpus Callosum Central
Volume of the Corpus Callosum Mid-Anterior
Volume of the Corpus Callosum Anterior

GLOBAL BRAIN METRICS:
Volume of the Cortex
Volume of the Cerebral White Matter
Volume of the Sub-Cortical Gray Matter
Total Gray Matter Volume
Whole Brain Gray Matter Density
Total Intracranial Volume

CEREBELLUM:
Volume of the Cerebellum White Matter
Volume of the Cerebellum cortex

Manhattan Plot:

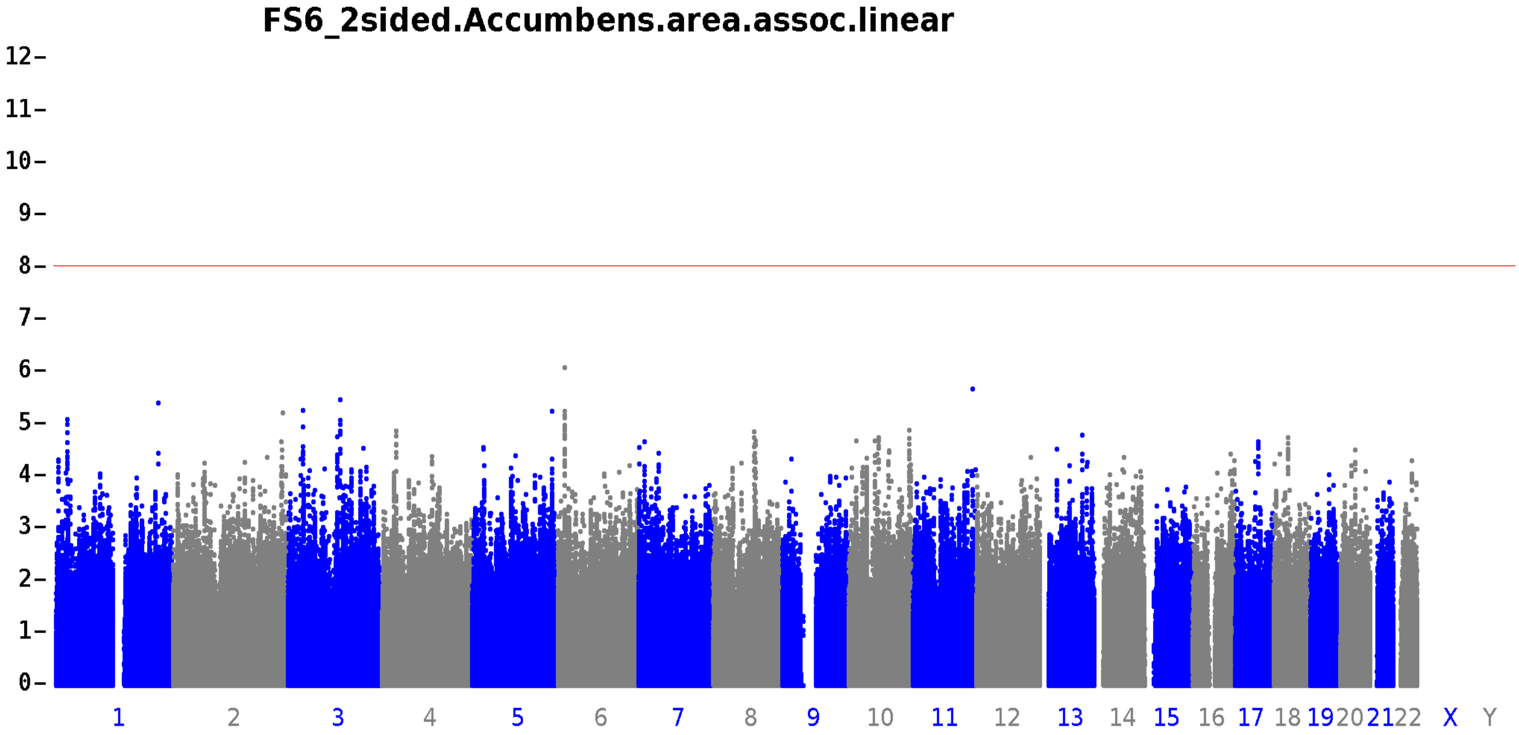

SNP mapped to gene:

CHR	BP	SNP	P	GENE
6	15195686	rs112702637	7.804e-07	missing
11	127445359	rs356256	1.997e-06	missing
3	110081920	rs35968545	3.319e-06	missing
1	216630651	rs4240919	3.788e-06	missing
3	31541300	rs12485487	5.14e-06	missing
6	15253398	rs11751041	5.492e-06	JARID2
5	169610644	chr5:169610644:I	5.501e-06	missing
2	231410520	rs6729378	5.855e-06	missing
6	15237062	rs6934978	6.552e-06	missing
6	15251181	rs9476810	7.222e-06	JARID2
1	24058449	rs2492599	7.871e-06	missing
3	110080138	rs4682575	8.191e-06	missing
1	24049595	rs561733	9.636e-06	missing
3	110081245	rs4352392	9.647e-06	missing

Manhattan Plot:

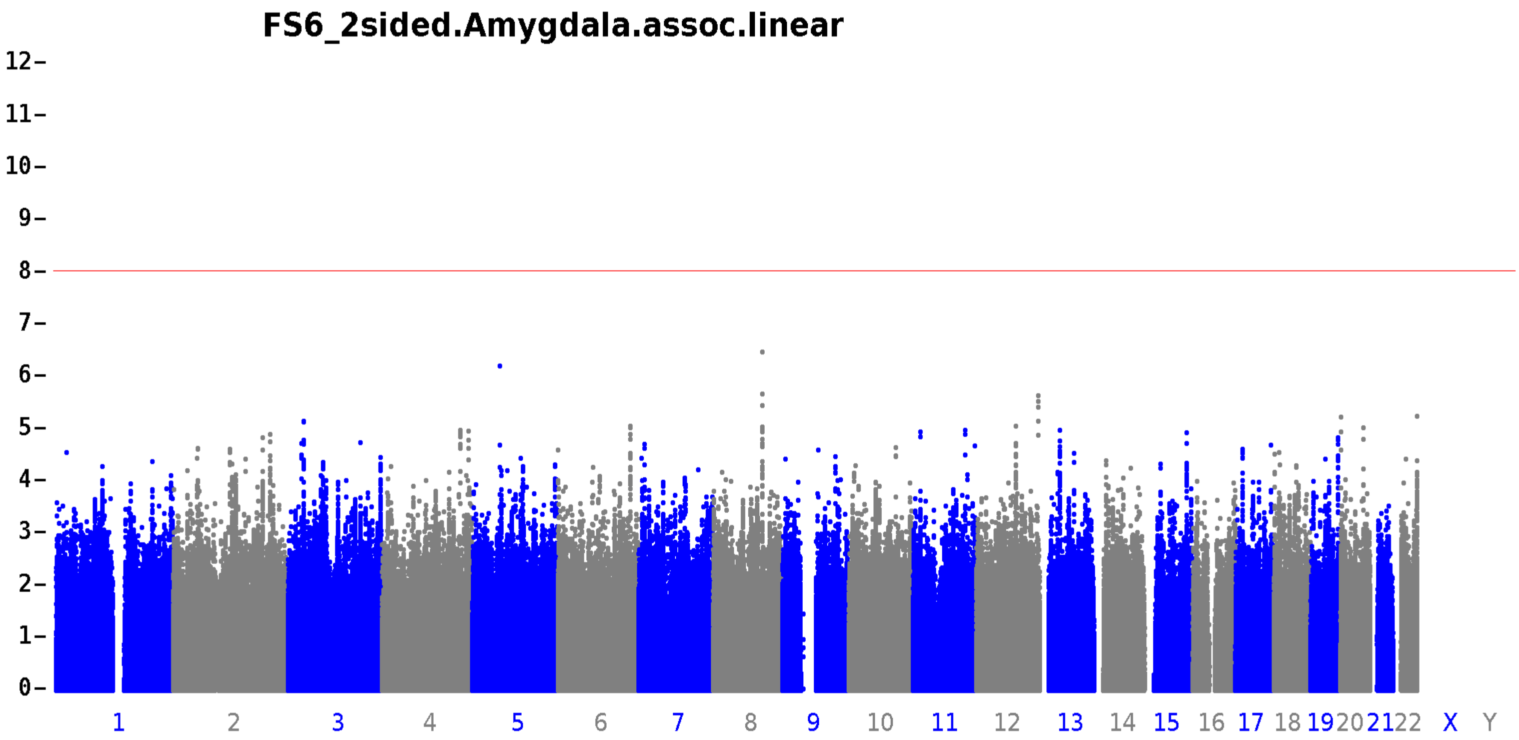

SNP mapped to gene:

CHR	BP	SNP	P	GENE
8	103940088	rs529802	3.213e-07	missing
5	58798099	rs35987304	5.86e-07	PDE4D
8	103934176	rs677406	2.018e-06	missing
12	131574485	rs1569020	2.2e-06	GPR133
12	131581429	rs11613786	2.788e-06	GPR133
8	103950997	rs571241	3.374e-06	LOC100996457
12	131573810	rs10848278	3.606e-06	GPR133
22	50555825	rs4838804	5.335e-06	MOV10L1
20	1707841	rs991958	5.614e-06	LOC100652885
3	34078751	chr3:34078751:I	6.698e-06	missing
12	131576445	rs1976930	6.781e-06	GPR133
3	34074670	rs35609149	6.986e-06	missing
3	34074424	rs35784002	6.986e-06	missing
12	83106277	rs17009882	8.287e-06	TMTC2
6	154716789	rs73009448	8.423e-06	missing
6	154716715	rs55800521	8.423e-06	missing
8	103898631	rs1806300	8.83e-06	missing
20	48985289	rs6512628	8.954e-06	missing
20	48985293	rs8124444	8.954e-06	missing
6	153311970	rs9479479	9.05e-06	MTRF1L
6	154714500	rs56341633	9.084e-06	missing
8	103944191	rs622072	9.593e-06	LOC100996457
13	42504822	rs75659673	9.888e-06	VWA8
4	166559145	rs1246905	9.91e-06	missing

4	166557848	rs1246908	9.91e-06	missing
4	166552698	rs736675	9.91e-06	missing
8	103896967	rs35656046	9.922e-06	missing

Manhattan Plot:

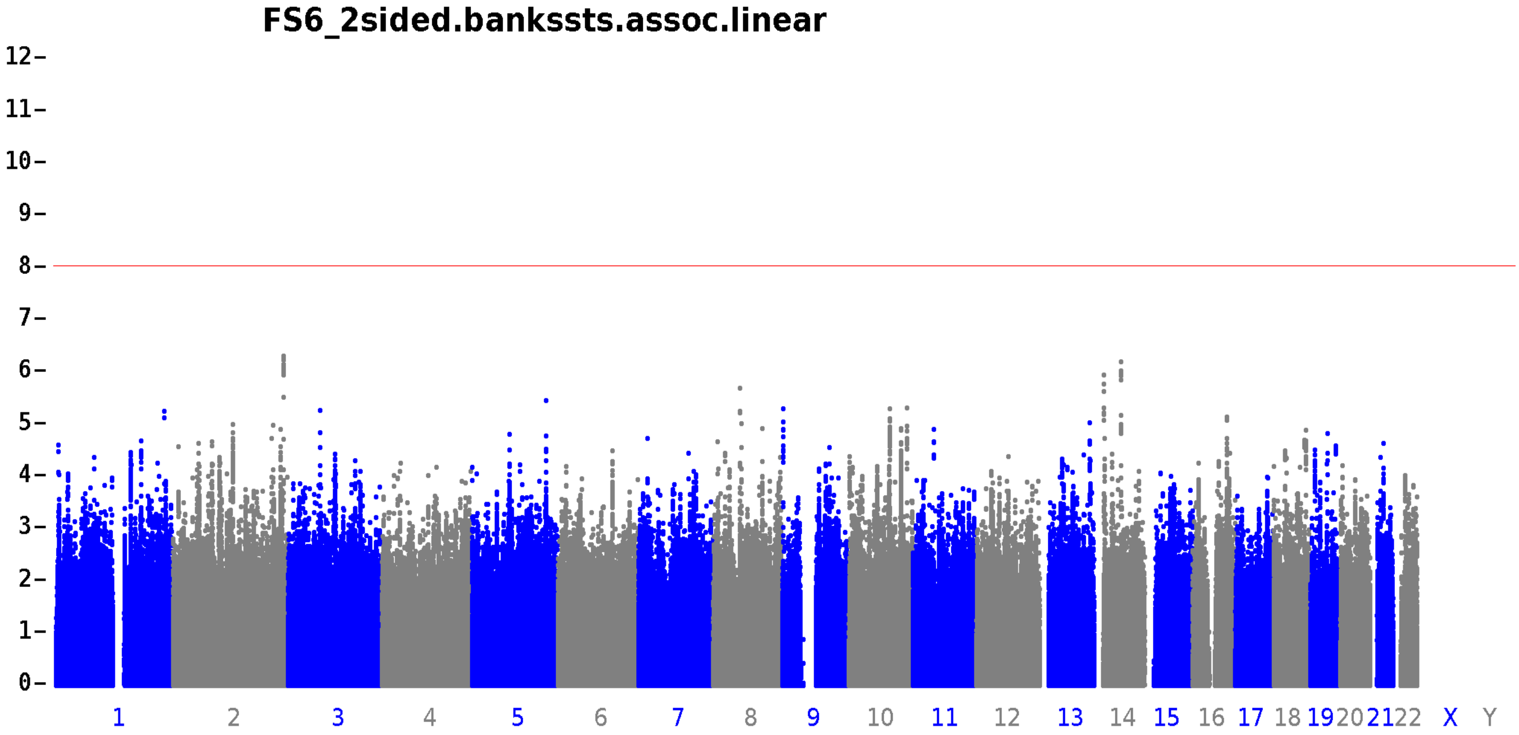

SNP mapped to gene:

CHR	BP	SNP	P	GENE
2	233866300	rs79943674	4.797e-07	NGEF
2	233852942	rs79782936	5.405e-07	NGEF
2	233858915	rs111430312	5.608e-07	NGEF
14	57546029	rs1189835	6.125e-07	missing
2	233856308	rs74845478	6.858e-07	NGEF
2	233867002	rs76980274	7.867e-07	NGEF
2	233863107	rs4076139	8.394e-07	NGEF
14	57555771	rs6573115	9.076e-07	missing
14	57543132	rs1189830	9.624e-07	missing
14	57545068	rs1189834	9.751e-07	missing
2	233866023	rs77327711	9.936e-07	NGEF
14	21819142	chr14:21819142:I	1.102e-06	RPGRIP1
2	233851715	rs78012699	1.107e-06	NGEF
14	57545017	rs1189832	1.13e-06	missing
14	57548152	rs1189837	1.15e-06	missing
14	57548024	rs1211818	1.347e-06	missing
14	21857916	rs61973165	1.614e-06	CHD8
8	56615711	rs73599169	1.97e-06	missing
14	21823117	rs17104027	2.261e-06	SUPT16H
2	233843598	rs78680200	2.938e-06	NGEF
5	155867428	rs4705016	3.362e-06	SGCD
10	123460639	rs11200084	4.671e-06	missing
14	21822787	rs61977523	4.689e-06	SUPT16H
14	21842814	rs61971524	4.689e-06	SUPT16H

14	21836612	rs61971521	4.689e-06	SUPT16H
14	21855439	rs138691852	4.689e-06	CHD8
14	21835948	rs8021793	4.689e-06	SUPT16H
14	21856356	rs61973164	4.689e-06	CHD8
9	1680072	rs1323237	4.846e-06	missing
10	87480439	rs4456206	4.882e-06	GRID1
3	68476074	rs6549126	5.242e-06	FAM19A1
1	230710196	rs73108011	5.34e-06	missing
8	56637333	rs55917188	5.354e-06	missing
1	230708964	rs73108008	5.439e-06	missing
14	21856642	chr14:21856642:D	5.792e-06	CHD8
8	56620446	rs113681805	5.8e-06	missing
14	21821149	rs61977522	6.184e-06	SUPT16H
14	57555846	rs6573116	6.586e-06	missing
16	71827792	rs11861285	7.113e-06	AP1G1
1	230710928	rs11804443	7.322e-06	missing
10	87481159	rs11201752	7.644e-06	GRID1
10	87507764	rs1917145	7.716e-06	GRID1
10	87483146	rs3740178	7.816e-06	GRID1
10	87481149	rs11201751	7.816e-06	GRID1
10	87479541	rs11201750	7.816e-06	GRID1
10	87480824	rs12217698	7.816e-06	GRID1
10	87479058	rs146934290	7.816e-06	GRID1
14	21817842	rs61977518	7.966e-06	RPGRIP1
16	71800540	rs115647000	8.181e-06	AP1G1
16	71800536	chr16:71800536:I	8.181e-06	AP1G1
10	87482345	rs17105793	8.192e-06	GRID1
10	87501953	rs74148750	8.492e-06	GRID1
9	1676734	rs7039858	8.751e-06	missing
13	106703620	rs17460862	9.119e-06	missing
8	59898060	rs7833731	9.332e-06	TOX
14	57556493	rs8015829	9.527e-06	missing
2	126180906	rs55900854	9.859e-06	missing

Manhattan Plot:

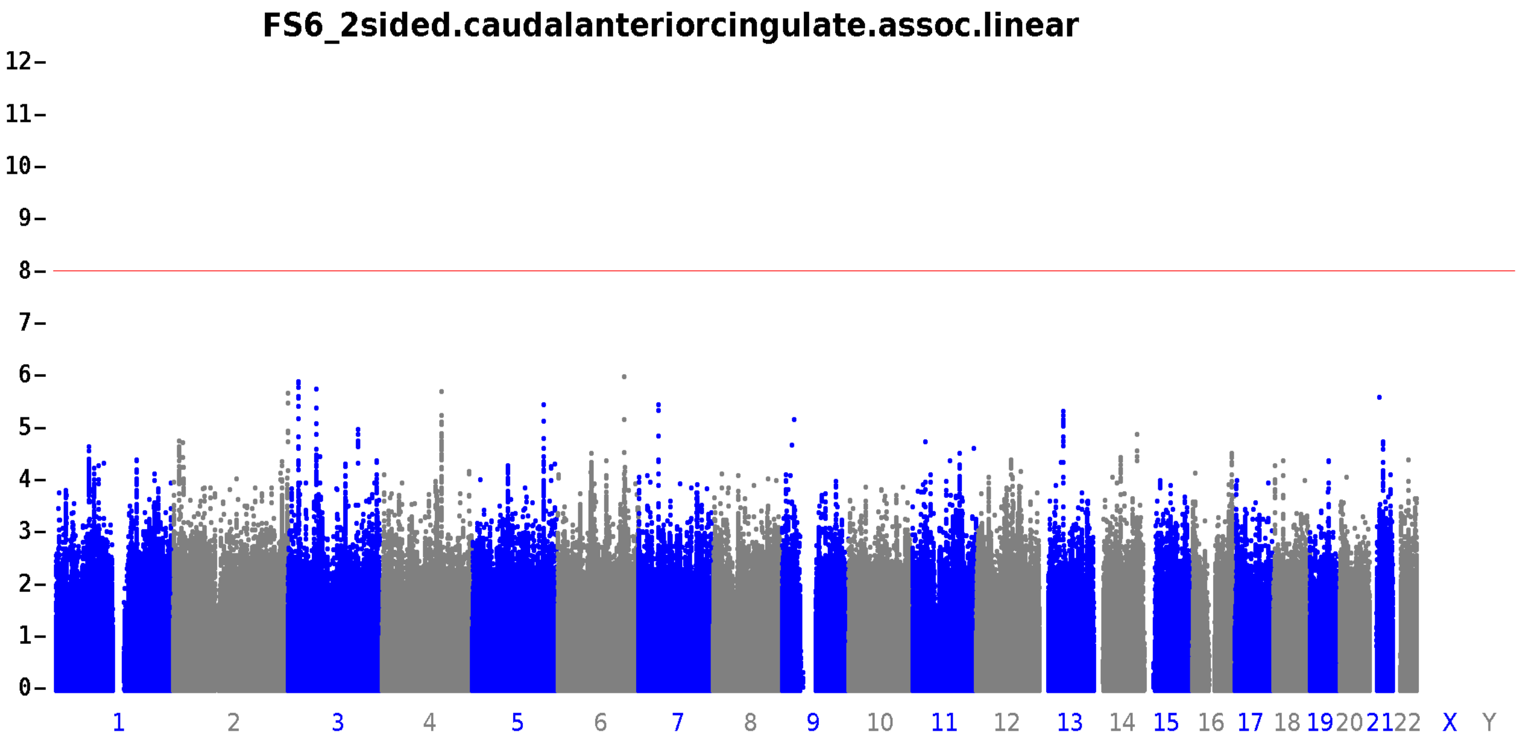

SNP mapped to gene:

CHR	BP	SNP	P	GENE
6	141813048	rs12190183	9.511e-07	missing
3	21636878	rs58466369	1.161e-06	ZNF385D
3	21634856	rs62236138	1.239e-06	ZNF385D
3	21637220	chr3:21637220:D	1.259e-06	ZNF385D
3	21638095	rs11705760	1.514e-06	ZNF385D
3	60802580	rs56290432	1.608e-06	FHIT
4	126629207	rs35356449	1.817e-06	missing
2	242843504	rs76494538	1.964e-06	LOC101927289
2	242842090	rs80145046	1.964e-06	LOC101927289
3	21606748	rs62236133	2.271e-06	ZNF385D
21	19789884	rs2268438	2.338e-06	missing
3	21639595	rs62237368	2.407e-06	ZNF385D
3	21638564	rs3901771	2.42e-06	ZNF385D
2	242842141	rs76854365	2.987e-06	LOC101927289
7	43584521	rs10245574	3.231e-06	HECW1
5	150977057	rs4958275	3.314e-06	missing
3	21634204	rs17009100	3.488e-06	ZNF385D
3	60800965	rs2364296	3.768e-06	FHIT
7	43581322	rs2117270	4.186e-06	HECW1
13	51101697	rs73184429	4.386e-06	LOC101929481
13	51104134	rs74078321	4.386e-06	LOC101929481
13	51102242	rs41284832	4.386e-06	LOC101929481
13	51063698	rs73201339	5.141e-06	LOC101929481
4	126627661	rs56735184	5.225e-06	missing

3	21608015	rs73033316	5.995e-06	ZNF385D
9	26038530	rs62543707	6.327e-06	missing
13	51109484	chr13:51109484:D	6.374e-06	LOC101929481
6	141817111	rs12662563	6.384e-06	missing
5	151395980	rs255490	6.77e-06	LOC101927115
13	51077323	rs55948456	6.886e-06	LOC101929481
4	126640280	chr4:126640280:D	7.043e-06	missing
13	51065040	chr13:51065040:D	7.095e-06	LOC101929481
13	51100953	rs73184428	7.373e-06	LOC101929481
3	60797868	rs56227671	7.614e-06	FHIT
4	126627180	rs34097882	7.798e-06	missing
4	126683528	chr4:126683528:D	7.903e-06	missing
13	51078879	rs145496814	8.379e-06	LOC101929481
3	148370025	rs13066735	9.571e-06	missing

Manhattan Plot:

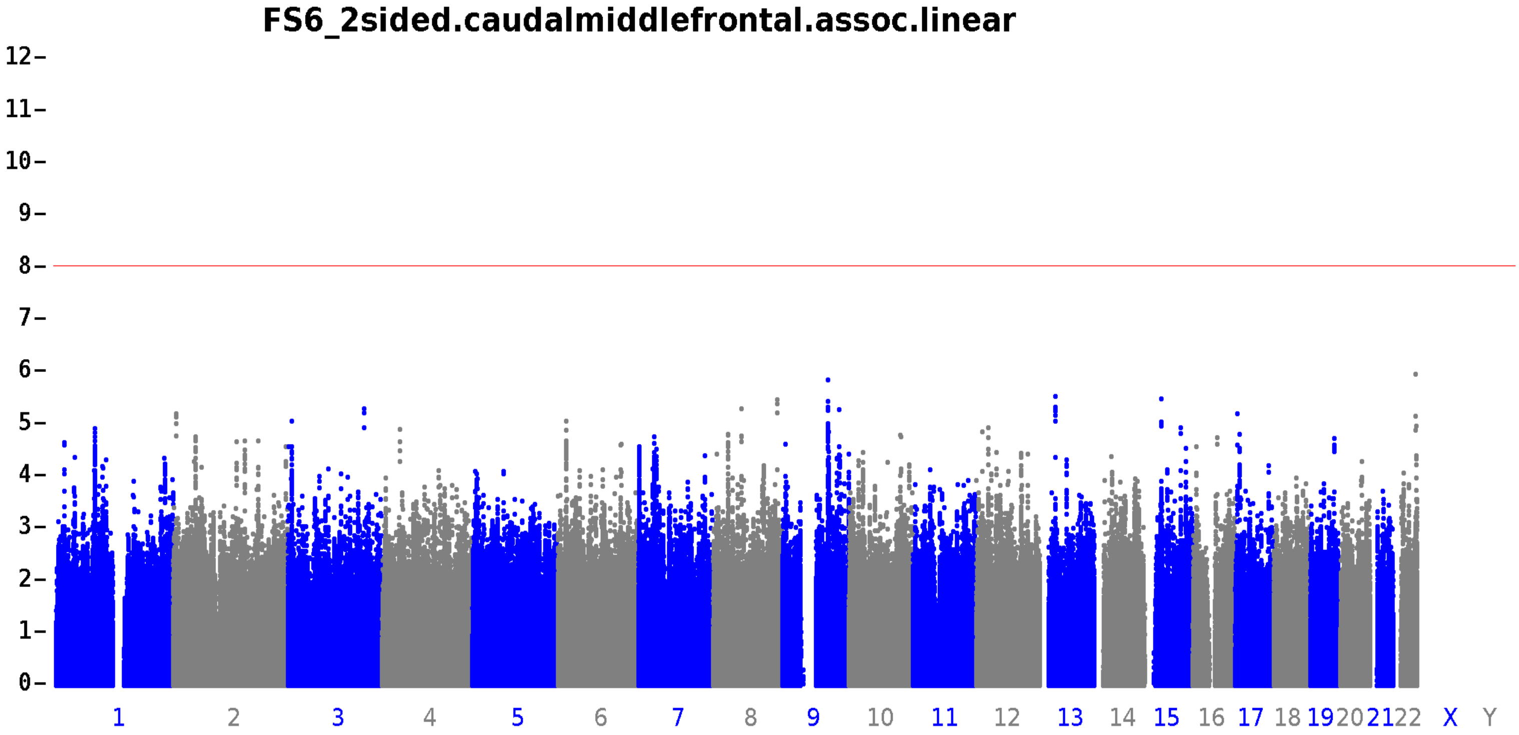

SNP mapped to gene:

CHR	BP	SNP	P	GENE
22	48819677	rs5768604	1.072e-06	missing
9	97328313	rs10761339	1.378e-06	LOC101928099,FBP2
13	32673042	rs433669	2.781e-06	FRY
15	35849903	rs59006481	3.181e-06	DPH6-AS1
8	134974922	rs17745716	3.297e-06	missing
9	97341169	rs13299030	3.527e-06	FBP2
8	134969538	rs6994575	3.96e-06	missing
13	32673837	rs438998	4.501e-06	FRY
13	32681518	rs434856	4.518e-06	FRY
9	97323816	rs11789810	4.576e-06	LOC101928099,FBP2
3	161948694	chr3:161948694:D	4.822e-06	missing
8	59659124	rs72649409	4.825e-06	missing
13	32673350	rs453774	4.947e-06	FRY
9	119707772	rs10116120	5.007e-06	ASTN2
13	32675472	rs464935	5.168e-06	FRY
9	97321127	rs1048510	5.196e-06	LOC101928099,FBP2
13	32677274	rs457793	5.364e-06	FRY
8	134969926	rs7837524	5.827e-06	missing
3	161942663	chr3:161942663:D	5.841e-06	missing
3	161942478	rs76373707	5.841e-06	missing
3	161942162	rs77753355	5.841e-06	missing
17	3403794	chr17:3403794:I	6.033e-06	SPATA22
2	5366923	rs73149679	6.033e-06	missing
13	32674508	rs465549	6.511e-06	FRY

22	48821349	rs762971	6.852e-06	missing
2	5373002	rs11893671	6.917e-06	missing
2	5385916	rs73151615	7.001e-06	missing
13	32678754	rs461373	8.418e-06	FRY
3	8186686	rs404753	8.465e-06	missing
6	18342260	rs13208674	8.498e-06	RNF144B
15	35862926	rs55884934	8.656e-06	DPH6-AS1
9	97337533	rs4744357	9.237e-06	FBP2
2	5381206	rs17274192	9.271e-06	missing
2	5386199	rs73151617	9.365e-06	missing
15	35850483	rs57187297	9.972e-06	DPH6-AS1

Manhattan Plot:

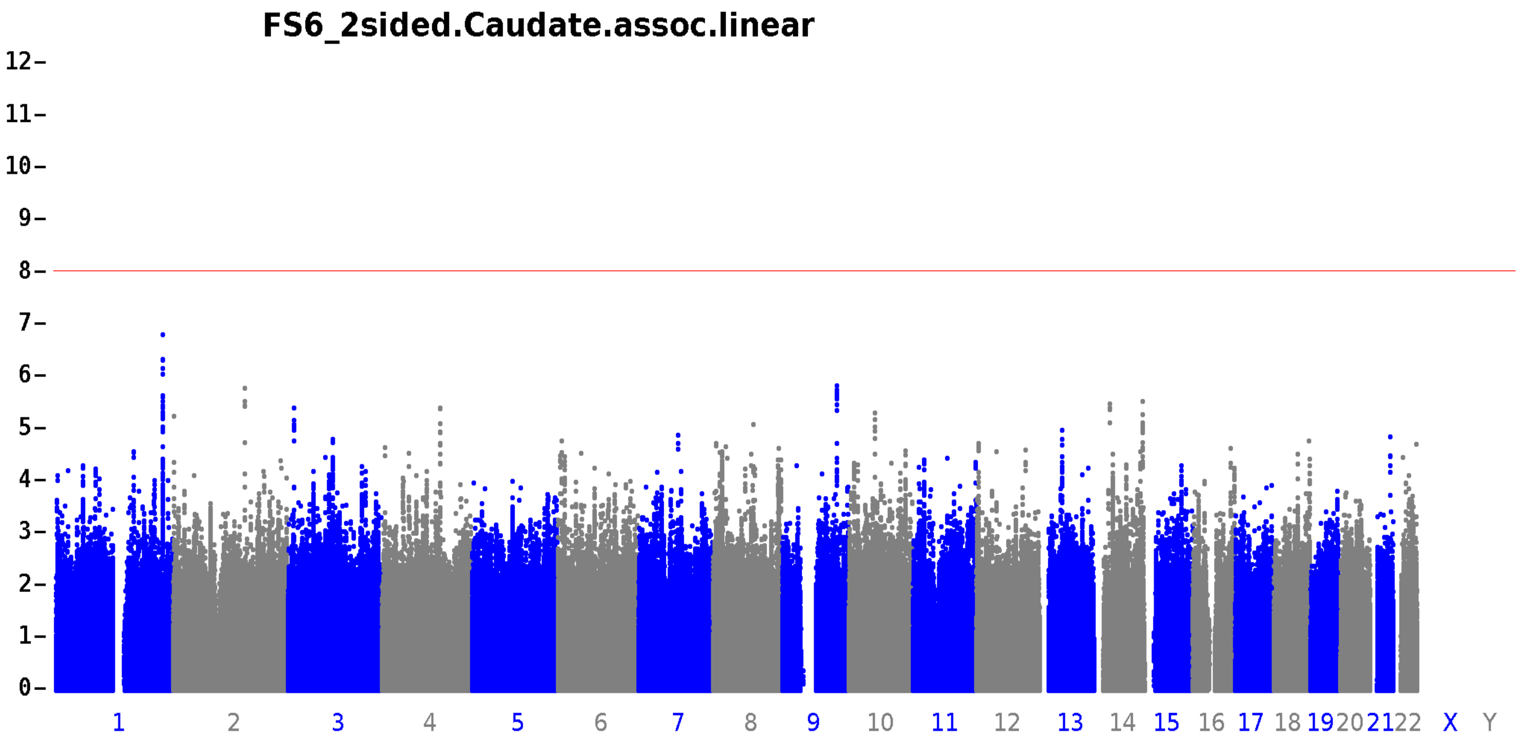

SNP mapped to gene:

CHR	BP	SNP	P	GENE
1	226325701	chr1:226325701:D	1.473e-07	missing
1	226319520	rs1219671	4.488e-07	missing
1	226312431	rs3892709	4.568e-07	missing
1	226405747	rs9728665	6.558e-07	missing
1	226405743	rs9728663	8.446e-07	missing
9	115414067	rs62575205	1.433e-06	KIAA1958
2	151029978	rs4667375	1.574e-06	missing
9	115401368	rs11788361	1.682e-06	KIAA1958
9	115413428	rs76498016	1.856e-06	KIAA1958
9	115413965	rs62575204	1.856e-06	KIAA1958
9	115413873	rs62575203	2.021e-06	KIAA1958
1	226407594	rs3008190	2.209e-06	missing
9	115409846	rs62575202	2.3e-06	KIAA1958
1	226473296	rs10753418	2.324e-06	LIN9
9	115403209	rs74307737	2.566e-06	KIAA1958
14	102703047	rs55780831	2.776e-06	MOK
1	226420722	rs2306119	2.782e-06	LIN9
1	226348005	rs9728014	2.8e-06	ACBD3
2	151029280	rs4417736	2.801e-06	missing
14	34283803	rs58842363	3.198e-06	missing
9	115404641	rs11792549	3.307e-06	KIAA1958
2	151025302	rs1374159	3.356e-06	missing
1	226494515	rs9286999	3.429e-06	LIN9
1	226445528	rs7367845	3.429e-06	LIN9

2	151026549	rs10195216	3.567e-06	missing
1	226407636	rs3008189	3.571e-06	missing
1	226423184	rs6703375	3.722e-06	LIN9
4	123656408	rs7658801	3.772e-06	BBS12
14	34282974	chr14:34282974:I	3.833e-06	missing
3	12493077	rs13091092	3.835e-06	missing
4	123662339	rs6833709	3.886e-06	BBS12
14	34284189	rs4146554	4.034e-06	missing
9	115407650	rs11794251	4.237e-06	KIAA1958
1	226408271	rs1219659	4.53e-06	missing
1	226357381	rs9286997	4.566e-06	ACBD3
10	54497540	chr10:54497540:D	4.73e-06	missing
1	226489455	rs1361559	4.967e-06	LIN9
14	102695693	rs2236493	5.003e-06	MOK
1	226427313	rs9629826	5.194e-06	LIN9
2	1877218	rs4519558	5.464e-06	MYT1L
1	226435429	rs6668540	5.957e-06	LIN9
1	226476665	rs7368170	5.99e-06	LIN9
10	54489680	rs1992526	6.335e-06	missing
3	12496887	rs13067911	6.595e-06	missing
14	34285469	rs10498323	7.141e-06	missing
14	102718419	rs28607975	7.326e-06	MOK
4	123639347	rs12054579	7.526e-06	missing
8	84928567	rs187743572	7.756e-06	missing
3	12487806	rs13088205	7.93e-06	missing
14	102720896	rs10145478	8.277e-06	MOK
1	226404531	rs3008191	8.677e-06	missing
10	54496641	rs55979213	8.689e-06	missing
14	102719620	rs3783372	8.746e-06	MOK
3	12487774	rs13065455	8.829e-06	missing
3	12487742	rs13065745	8.829e-06	missing
14	102698599	rs7153005	8.857e-06	MOK
1	226405710	rs2927339	9.618e-06	missing
1	226403187	rs1219652	9.662e-06	missing
14	102713581	rs12896377	9.918e-06	MOK

Manhattan Plot:

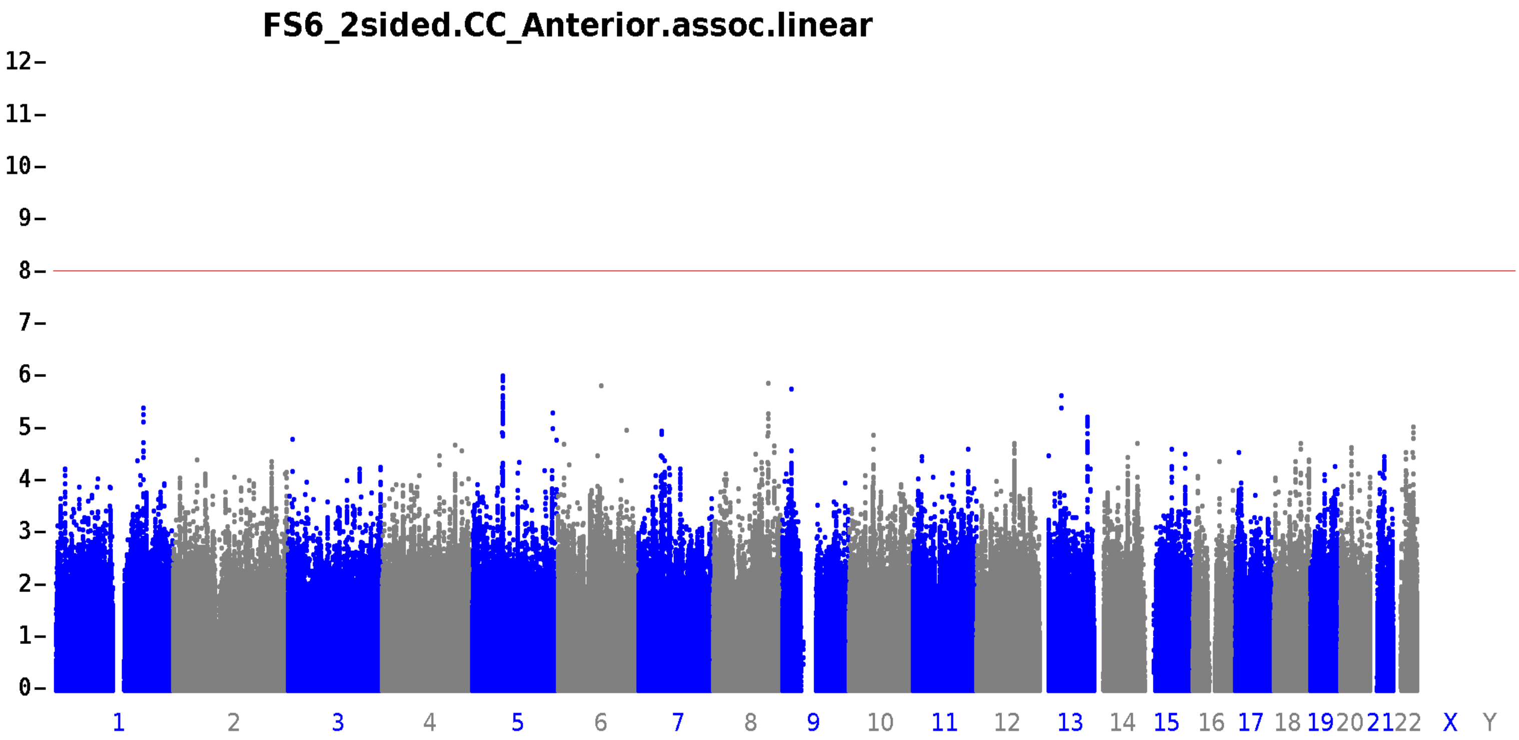

SNP mapped to gene:

CHR	BP	SNP	P	GENE
5	64570534	chr5:64570534:D	8.982e-07	ADAMTS6
5	64582490	rs2287905	1.009e-06	ADAMTS6
5	64580411	rs60793438	1.088e-06	ADAMTS6
5	64583785	rs67235604	1.147e-06	ADAMTS6
8	116227081	rs150767412	1.257e-06	missing
6	93121818	chr6:93121818:I	1.393e-06	missing
5	64567608	rs10461383	1.515e-06	ADAMTS6
5	64563237	rs10071190	1.533e-06	ADAMTS6
5	64562952	chr5:64562952:D	1.533e-06	ADAMTS6
5	64569420	rs1423425	1.589e-06	ADAMTS6
9	18829569	rs10963787	1.622e-06	ADAMTSL1
13	46295439	rs77418508	2.162e-06	missing
5	64574685	rs10077096	2.209e-06	ADAMTS6
5	64573931	rs10060077	2.209e-06	ADAMTS6
5	64586377	chr5:64586377:I	2.269e-06	ADAMTS6
5	64592775	rs10063048	2.426e-06	ADAMTS6
5	64565332	rs10057760	2.891e-06	ADAMTS6
5	64597580	rs10070189	3.006e-06	ADAMTS6
5	64597538	chr5:64597538:D	3.183e-06	ADAMTS6
5	64536143	rs77484235	3.581e-06	ADAMTS6
13	46291921	rs79130773	3.758e-06	missing
13	46291990	rs78260235	3.758e-06	missing
1	185368936	chr1:185368936:I	3.778e-06	missing
5	64567361	rs10461502	3.808e-06	ADAMTS6

5	64573981	rs10053096	4.467e-06	ADAMTS6
5	64597775	rs28412757	4.648e-06	ADAMTS6
5	171103318	rs931117	4.735e-06	missing
5	64556640	rs10940023	4.778e-06	ADAMTS6
8	116213116	rs6992538	4.878e-06	missing
1	185365267	rs1474837	4.987e-06	missing
1	185365240	rs1474836	4.987e-06	missing
5	64530082	rs1593039	5.276e-06	ADAMTS6
5	64535877	rs10058195	5.34e-06	ADAMTS6
5	64525521	rs11747988	5.555e-06	ADAMTS6
13	100895427	rs629509	5.695e-06	PCCA
5	64523854	rs13360673	5.887e-06	ADAMTS6
8	116226324	rs71528501	5.947e-06	missing
5	64525440	rs11747963	6.014e-06	ADAMTS6
5	64525434	rs11747962	6.014e-06	ADAMTS6
13	100898972	rs7333362	6.585e-06	PCCA
5	64522470	rs11747110	6.647e-06	ADAMTS6
1	185371940	rs2144291	7.017e-06	missing
13	100878820	rs7318781	7.349e-06	PCCA
5	64548961	rs10471310	7.55e-06	ADAMTS6
8	116221056	rs73703186	8.285e-06	missing
13	100873945	rs684215	8.352e-06	PCCA
22	42549164	rs5758613	8.797e-06	missing
5	171103434	rs931115	9.235e-06	missing

Manhattan Plot:

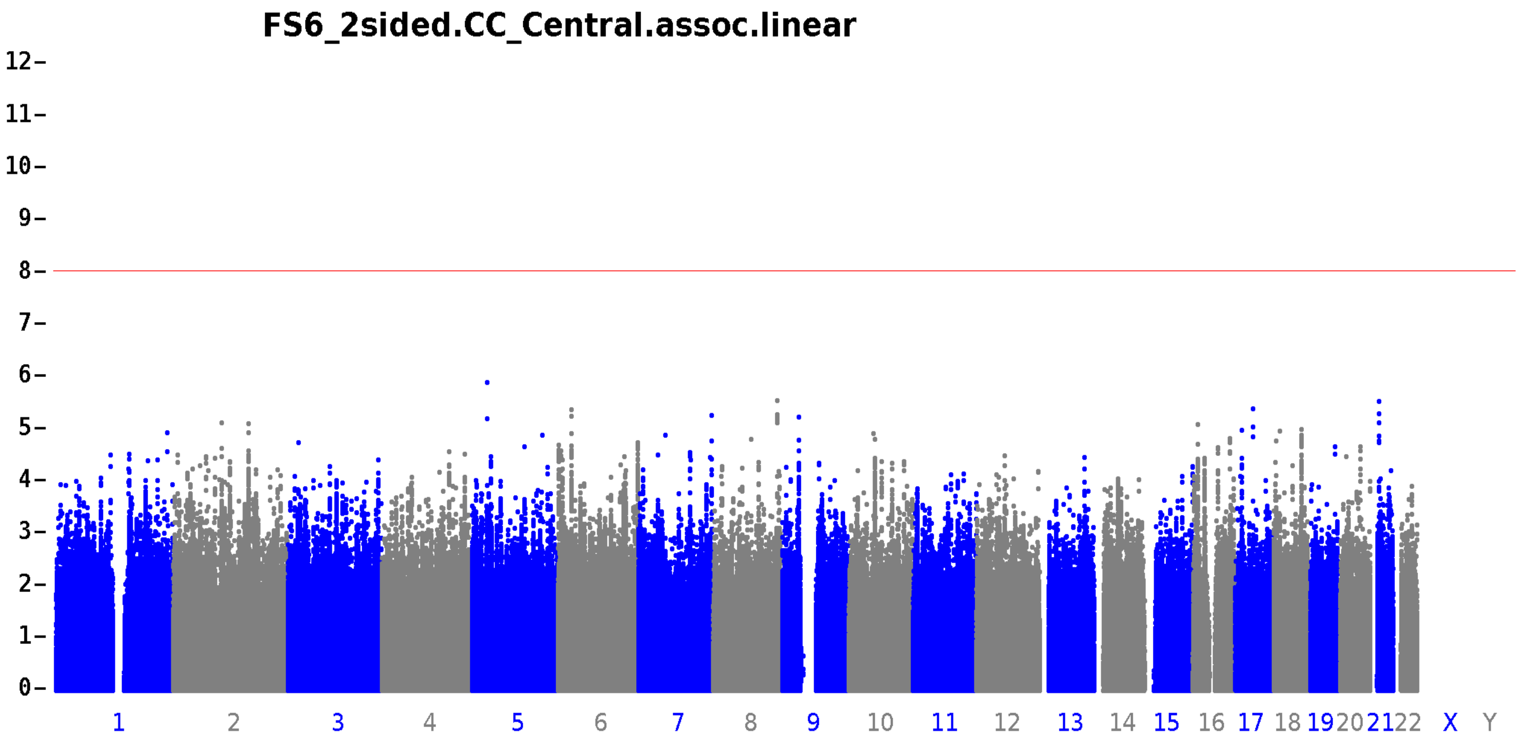

SNP mapped to gene:

CHR	BP	SNP	P	GENE
5	32093659	rs157499	1.226e-06	PDZD2
8	134621664	rs10956712	2.731e-06	missing
21	18971877	rs6517780	2.798e-06	BTG3
17	37214804	rs72821570	3.947e-06	LOC100131347
6	29477783	rs114459546	3.984e-06	missing
21	18959089	rs8133097	4.914e-06	CXADR
8	134617284	rs10087104	5.067e-06	missing
7	155641708	rs1225381	5.2e-06	missing
6	29477273	rs115652027	5.484e-06	missing
9	35086028	rs576376	5.602e-06	missing
8	134638258	rs3958181	5.644e-06	missing
5	32092150	rs157498	6.043e-06	PDZD2
8	134628594	rs34661725	6.506e-06	missing
8	134620332	rs7816514	7.132e-06	missing
2	103197839	rs11695255	7.166e-06	missing
21	18980268	rs2824397	7.204e-06	BTG3
8	134621178	rs10956711	7.305e-06	missing
2	160098266	rs58061225	7.607e-06	WDSUB1
16	11250413	rs7189563	7.781e-06	CLEC16A
17	37229707	rs77616360	8.578e-06	LOC100131347,PLXDC1
18	59287208	rs12185327	9.542e-06	missing
17	13386530	rs72637305	9.984e-06	missing

Manhattan Plot:

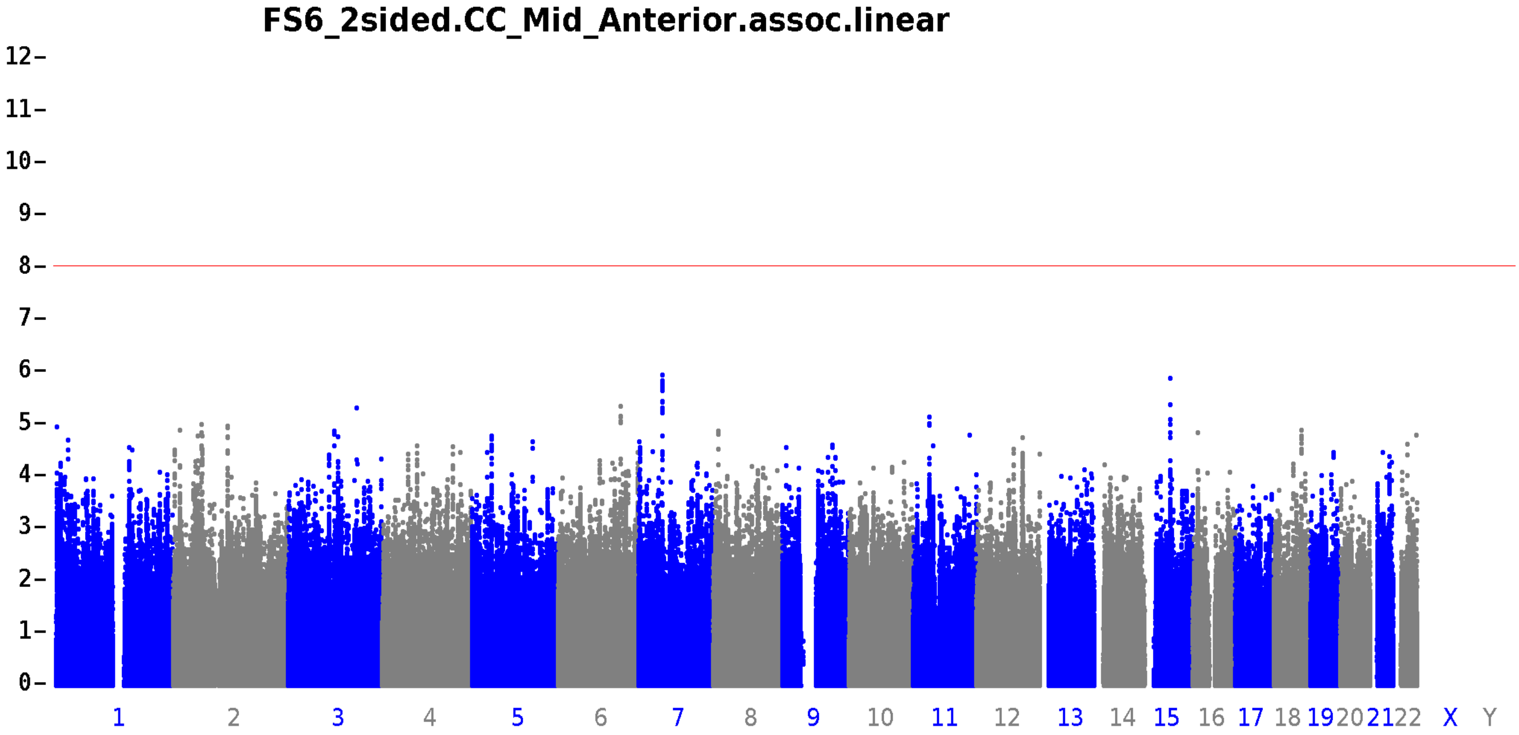

SNP mapped to gene:

CHR	BP	SNP	P	GENE
7	50723256	rs3807546	1.081e-06	GRB10
15	53847230	rs58156072	1.261e-06	WDR72
7	50714865	chr7:50714865:I	1.394e-06	GRB10
7	50722460	rs4504586	1.652e-06	GRB10
7	50731802	rs2237465	1.706e-06	GRB10
7	50726144	rs2237457	1.804e-06	GRB10
7	50712820	rs3807547	1.947e-06	GRB10
7	50712272	rs17133918	1.968e-06	GRB10
7	50717977	rs886823	2.044e-06	GRB10
7	50715083	rs17133922	2.044e-06	GRB10
7	50718654	rs737292	2.044e-06	GRB10
7	50729944	rs2237462	2.161e-06	GRB10
7	50701814	rs12530592	3.569e-06	GRB10
7	50700936	rs3901371	3.632e-06	GRB10
15	53808363	rs7182198	4.091e-06	WDR72
6	132898363	rs6930765	4.422e-06	missing
7	50705576	chr7:50705576:D	4.62e-06	GRB10
3	145532325	rs11706895	4.652e-06	missing
7	50698549	rs2237442	5.044e-06	GRB10
7	50704124	rs76969172	5.744e-06	GRB10
6	132899399	rs6937506	6.668e-06	missing
6	132898786	rs6935920	6.668e-06	missing
6	132897029	rs6924201	7.061e-06	missing
11	35316846	rs7946149	7.088e-06	SLC1A2

15	53799293	rs3941046	7.881e-06	missing
6	132896447	rs6941797	8.441e-06	missing
6	132899665	rs6921461	8.875e-06	missing
6	132899761	rs6941913	8.905e-06	missing
6	132896910	rs6903874	8.927e-06	missing
6	132895403	rs17061465	8.927e-06	missing
11	35317712	rs10742340	9.323e-06	SLC1A2
11	35317717	rs10742341	9.323e-06	SLC1A2
11	35317353	rs3794091	9.341e-06	SLC1A2
11	35317415	rs6484778	9.545e-06	SLC1A2
15	53811187	rs7180554	9.739e-06	WDR72
2	60463594	rs141820716	9.843e-06	missing

Manhattan Plot:

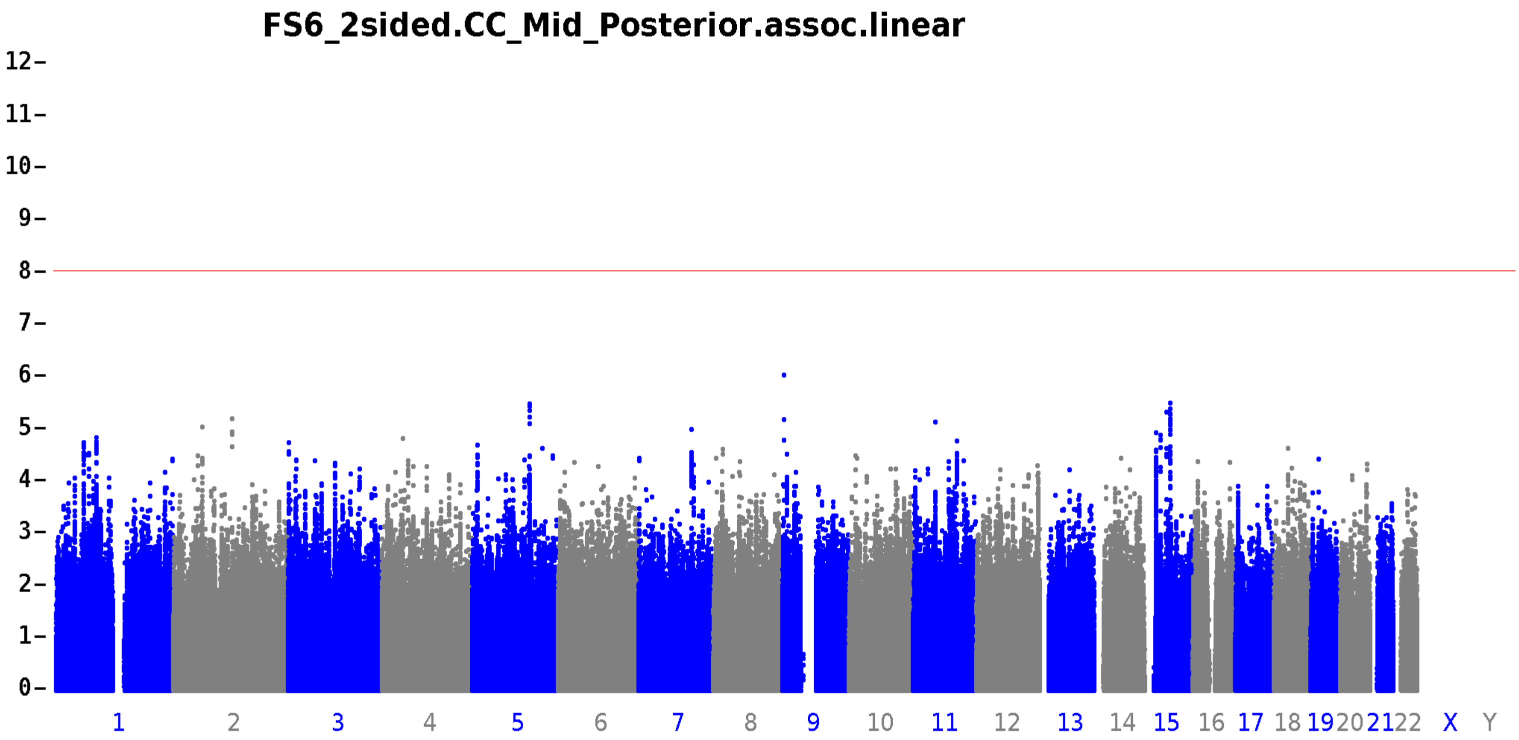

SNP mapped to gene:

CHR	BP	SNP	P	GENE
9	2690710	rs77260291	8.755e-07	missing
15	55196089	rs72738235	3.027e-06	missing
5	120765218	rs10061417	3.172e-06	missing
5	120764582	rs2406514	3.172e-06	missing
5	120770749	rs13188343	3.541e-06	missing
15	55176276	rs55714641	3.92e-06	missing
5	120769879	rs10077184	4.246e-06	missing
15	45974869	rs115372813	4.483e-06	SQRDL
15	55165033	rs28763276	4.951e-06	missing
15	55150802	rs12440505	5.08e-06	missing
15	55189354	rs55721632	5.115e-06	missing
5	120763492	rs10434736	5.598e-06	missing
15	55151641	rs17818855	5.958e-06	missing
2	125183450	rs17011501	6.148e-06	CNTNAP5
9	2687539	rs55992105	6.253e-06	missing
15	55146622	rs145879997	6.825e-06	missing
11	47703311	rs79408863	6.95e-06	AGBL2
5	120763754	rs75147963	7.489e-06	missing
15	55175350	rs17818867	7.73e-06	missing
15	55139343	rs34121292	7.874e-06	missing
15	55165143	rs28804377	8.063e-06	missing
2	61970732	rs7596428	8.624e-06	missing
2	61974383	rs113299517	8.805e-06	missing
2	61974335	rs113697620	8.805e-06	missing

15	55178773	rs11634410	9.616e-06	missing
7	113068345	rs6466447	9.875e-06	missing

Manhattan Plot:

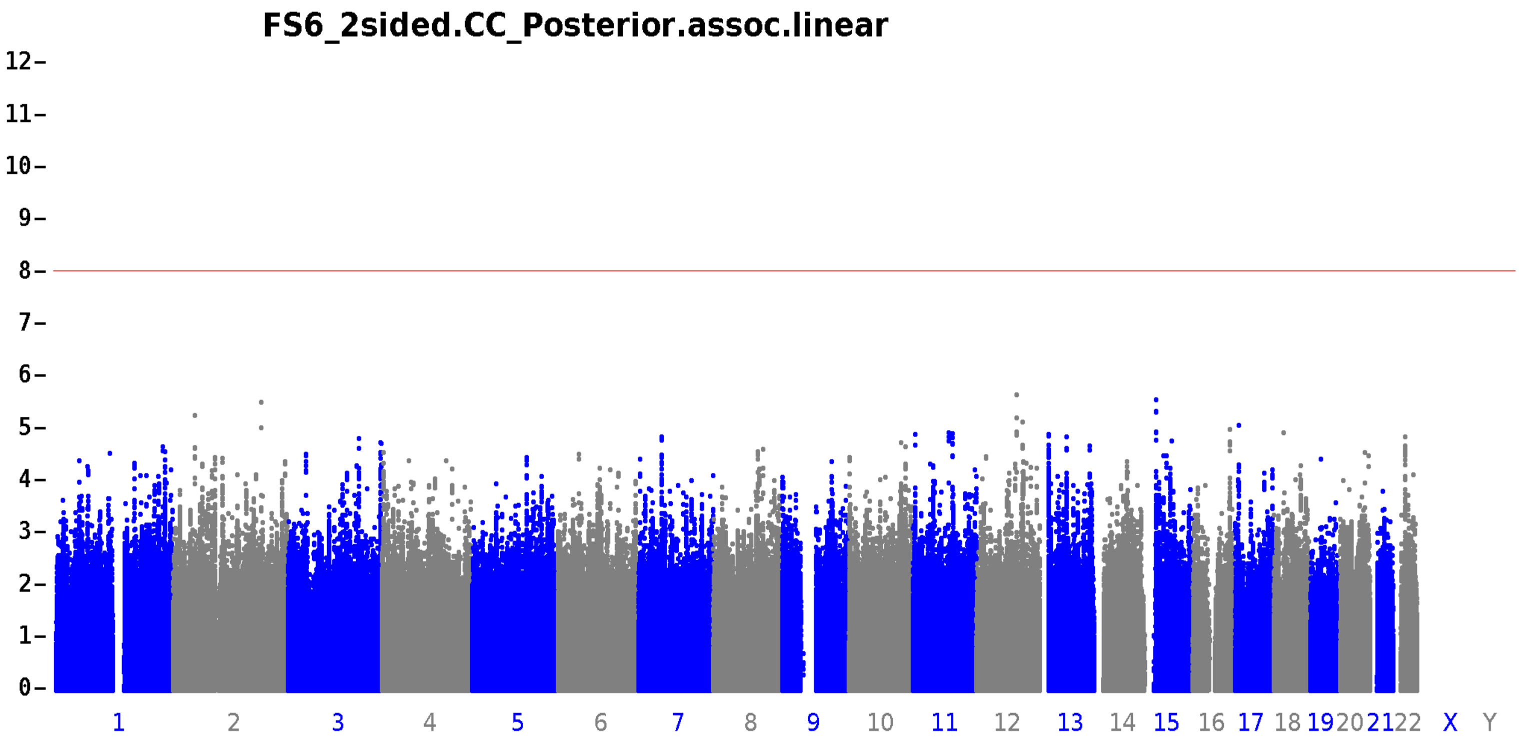

SNP mapped to gene:

CHR	BP	SNP	P	GENE
12	85237617	rs10746336	2.121e-06	missing
15	24315868	rs1850477	2.608e-06	missing
15	24316069	rs1850479	2.608e-06	missing
15	24315398	rs2883506	2.608e-06	missing
15	24317142	rs6420964	2.608e-06	missing
15	24317987	rs58060368	2.608e-06	missing
2	186076220	rs71430179	2.878e-06	missing
15	24310440	rs75846095	4.417e-06	missing
15	24313918	rs6576663	4.531e-06	missing
15	24313681	rs6576662	4.531e-06	missing
2	46278720	rs12712961	5.139e-06	PRKCE
12	85238411	rs1074325	5.753e-06	missing
12	97063357	rs11108631	6.966e-06	C12orf55
17	7371932	rs55749333	8.193e-06	ZBTB4,SLC35G6
2	185984976	rs144161862	8.864e-06	missing
16	78264680	rs10221047	9.823e-06	WWOX

Manhattan Plot:

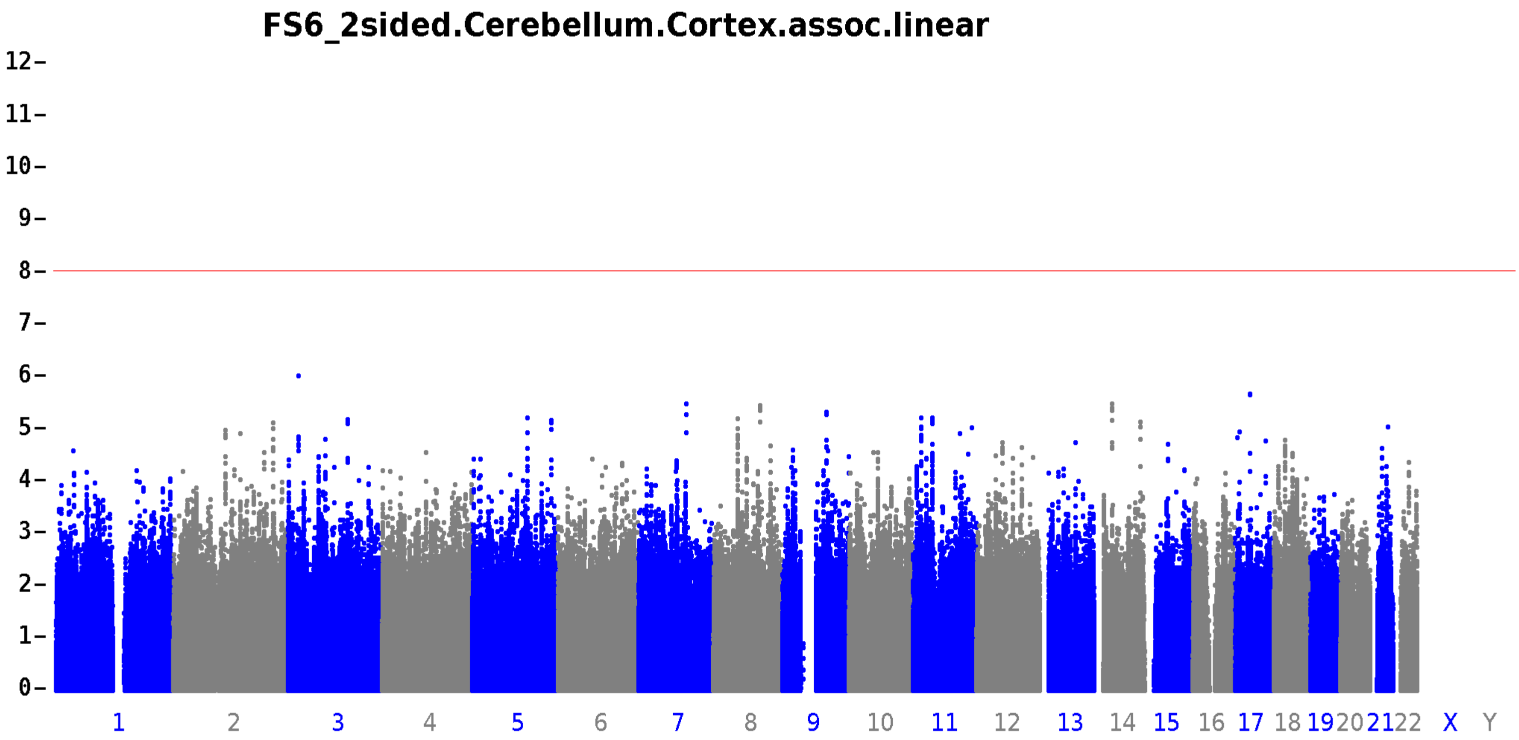

SNP mapped to gene:

CHR	BP	SNP	P	GENE
3	22457663	rs1548137	9.092e-07	missing
17	31702422	rs7215745	2.041e-06	ASIC2
17	31702256	rs10438803	2.077e-06	ASIC2
14	38301988	rs7142469	3.091e-06	LOC101059957
7	101909005	rs1725600	3.163e-06	CUX1
8	99089484	rs2513831	3.426e-06	C8orf47
14	38310537	rs11629163	3.721e-06	LOC101059957
8	99088463	rs2513828	3.971e-06	C8orf47
8	99089452	rs2447513	4.14e-06	C8orf47
14	38305047	rs1952782	4.245e-06	LOC101059957
9	93331998	rs73503347	4.461e-06	LOC340515
9	93331573	rs73503343	4.81e-06	LOC340515
7	101907679	rs1725596	4.961e-06	CUX1
9	93328448	rs77877888	4.967e-06	LOC340515
9	93328011	rs57643896	4.967e-06	LOC340515
5	116143516	rs17373407	5.729e-06	missing
11	17063304	chr11:17063304:D	5.765e-06	missing
11	41033103	rs1462231	5.888e-06	LRRC4C
11	41043860	chr11:41043860:I	6.109e-06	LRRC4C
8	52111217	rs35027875	6.155e-06	missing
3	126207222	rs777483	6.186e-06	UROC1
14	38311840	rs3825636	6.398e-06	missing
11	41064950	rs1462222	6.493e-06	LRRC4C
5	167853433	rs11738811	6.622e-06	WWC1

14	98668618	rs34440630	7.055e-06	missing
8	99088913	rs2513829	7.107e-06	C8orf47
2	211676025	rs72941874	7.161e-06	missing
3	126206800	rs812366	7.203e-06	UROC1
5	167858004	rs729149	7.35e-06	WWC1
11	41026972	rs355243	7.422e-06	LRRC4C
3	126206669	rs777485	7.65e-06	UROC1
11	17062164	rs56254605	8.55e-06	missing
14	98649816	rs9323992	8.785e-06	missing
21	38117505	rs2073602	8.842e-06	SIM2
11	125824737	rs948113	9.017e-06	LOC338667
11	17063716	rs12417522	9.248e-06	missing
8	52102487	rs10958248	9.278e-06	missing
2	211809236	rs4410224	9.423e-06	missing
5	167849669	rs11740649	9.839e-06	WWC1

Manhattan Plot:

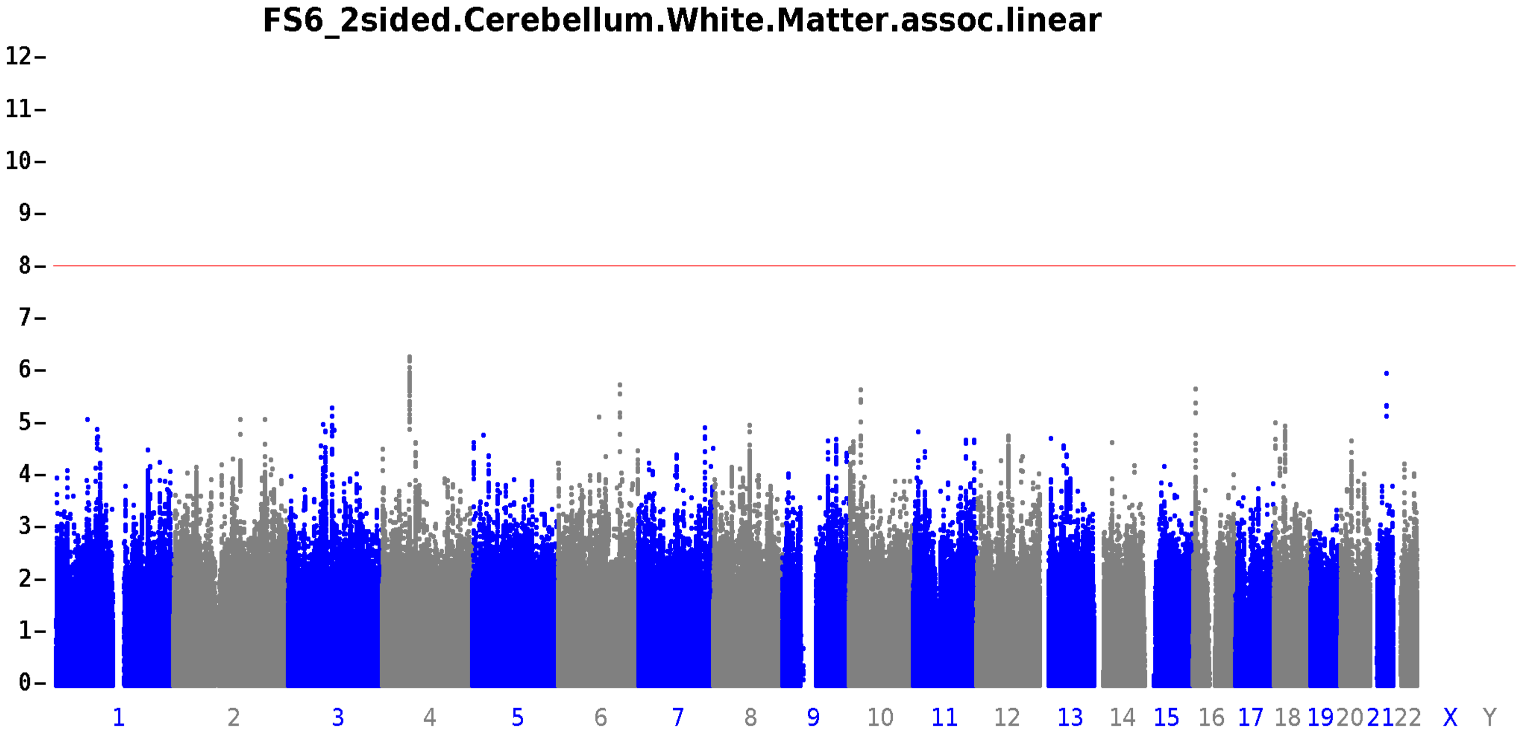

SNP mapped to gene:

CHR	BP	SNP	P	GENE
4	58311743	rs12644813	4.869e-07	LOC101928851
4	58311457	rs12642652	4.869e-07	LOC101928851
4	58327800	rs12641903	5.64e-07	LOC101928851
4	58318669	rs13146405	5.917e-07	LOC101928851
4	58319435	rs71601691	7.905e-07	LOC101928851
4	58327372	rs17831783	9.851e-07	LOC101928851
4	58327040	rs17769765	9.851e-07	LOC101928851
4	58345932	rs35898241	9.947e-07	missing
21	34625590	rs13047715	1.028e-06	IFNAR2
4	58339009	rs7664064	1.117e-06	missing
4	58310349	rs36038005	1.165e-06	LOC101928851
4	58317675	rs71601690	1.186e-06	LOC101928851
4	58341448	chr4:58341448:D	1.209e-06	missing
4	58336532	rs13129428	1.401e-06	missing
4	58341893	rs35158236	1.438e-06	missing
4	58326711	rs78766201	1.542e-06	LOC101928851
4	58320684	rs6554466	1.578e-06	LOC101928851
4	58311974	rs34787896	1.654e-06	LOC101928851
6	131317527	chr6:131317527:D	1.701e-06	EPB41L2
4	58313146	rs17769432	1.734e-06	LOC101928851
4	58314049	rs34836578	1.942e-06	LOC101928851
16	5620740	rs4341747	2.052e-06	RBFOX1
10	25529753	rs11014456	2.133e-06	GPR158
4	58334803	rs78361796	2.164e-06	missing

4	58334611	rs143285660	2.228e-06	missing
6	131317876	rs7752292	2.511e-06	EPB41L2
4	58332294	rs7669775	2.696e-06	missing
10	25550669	rs16925515	3.306e-06	GPR158
4	58315048	rs12647474	3.49e-06	LOC101928851
10	25533010	rs12358959	3.655e-06	GPR158
16	5623455	rs12930250	3.707e-06	RBFOX1
4	58326155	rs17088150	3.888e-06	LOC101928851
4	58327895	rs12641925	4.177e-06	LOC101928851
21	34620409	rs2834162	4.177e-06	IFNAR2
21	34620668	rs17860211	4.177e-06	IFNAR2
4	58326979	rs72606413	4.177e-06	LOC101928851
21	34620113	rs2834160	4.336e-06	IFNAR2
3	93869952	rs73159602	4.67e-06	missing
3	93869430	rs73159600	4.67e-06	missing
4	58330329	chr4:58330329:I	4.971e-06	LOC101928851
16	5613170	rs4786042	5.777e-06	RBFOX1
6	131352447	rs7774648	5.921e-06	EPB41L2
4	58322150	rs35570786	6.302e-06	LOC101928851
3	93838432	chr3:93838432:I	6.647e-06	NSUN3
21	34618089	rs2834159	6.744e-06	IFNAR2
3	93843580	rs73159590	6.823e-06	NSUN3
6	87897365	rs9450629	6.873e-06	ZNF292
6	131394554	rs7752642	6.909e-06	missing
4	58330503	rs36117298	7.432e-06	LOC101928851
4	58329946	rs12641533	7.432e-06	LOC101928851
4	58330622	rs34314852	7.432e-06	LOC101928851
4	58330679	rs34991989	7.432e-06	LOC101928851
2	142567471	rs10496900	7.751e-06	LRP1B
2	194276934	rs1837491	7.833e-06	missing
1	67195776	rs115745439	7.928e-06	SGIP1
4	58322796	rs34247570	8.308e-06	LOC101928851
4	58324183	chr4:58324183:D	8.587e-06	LOC101928851
10	25529571	chr10:25529571:D	8.611e-06	GPR158
18	3288708	chr18:3288708:I	9.173e-06	missing
3	73733092	rs56703930	9.776e-06	missing

Manhattan Plot:

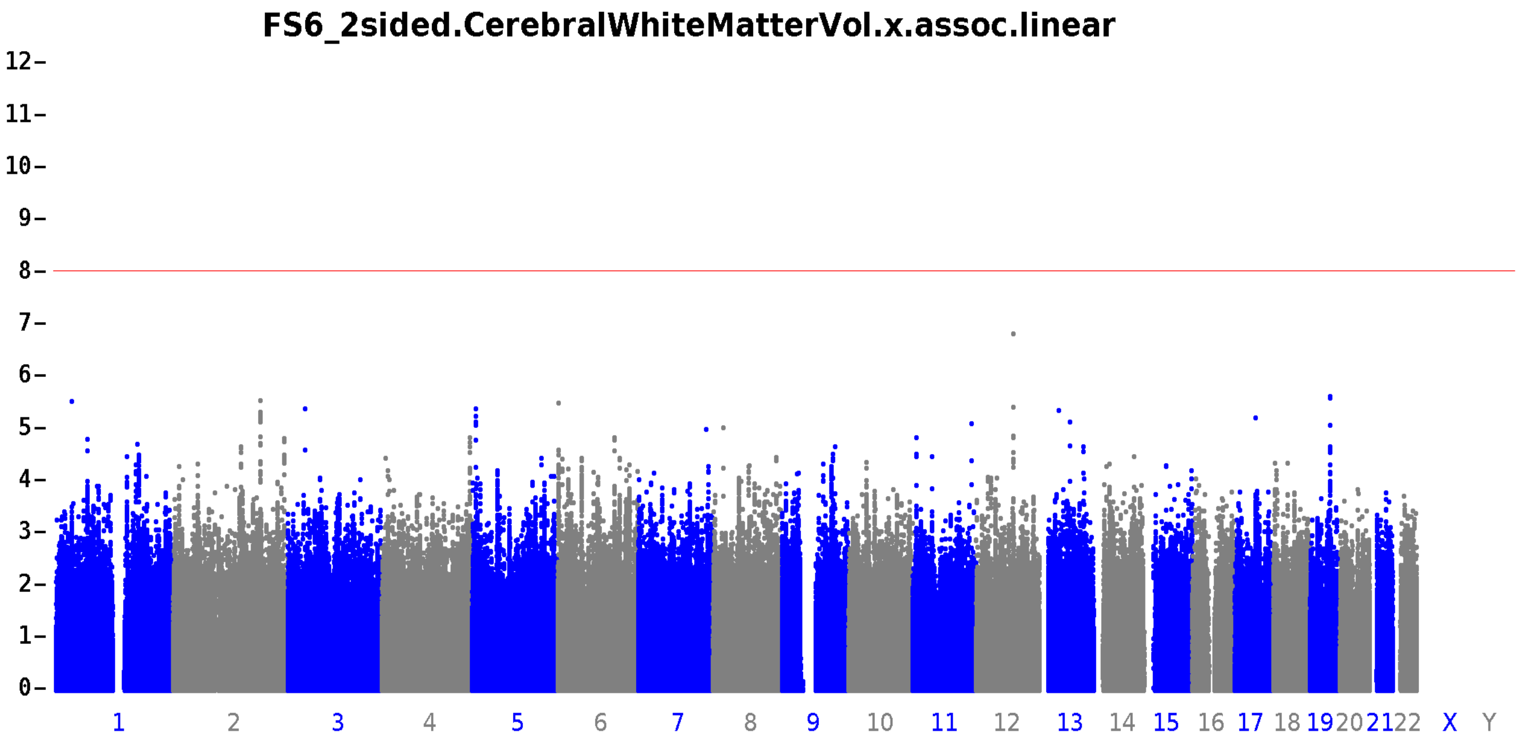

SNP mapped to gene:

CHR	BP	SNP	P	GENE
12	78761426	rs7295312	1.443e-07	missing
19	42767593	rs113028115	2.271e-06	missing
19	42747229	rs11878620	2.444e-06	missing
2	185353473	rs1014024	2.753e-06	missing
1	33788884	rs7542777	2.803e-06	missing
6	3075553	rs2326173	3.068e-06	RIPK1
12	78765879	rs10860350	3.654e-06	missing
3	36077962	rs2359962	3.841e-06	missing
5	7306148	rs116604819	3.877e-06	LOC442132
13	40853346	rs7331658	4.17e-06	missing
2	185360281	rs114318379	4.532e-06	missing
2	185361579	rs1596267	4.839e-06	missing
2	185362483	rs17429906	4.905e-06	missing
5	7314085	chr5:7314085:D	5.345e-06	missing
2	185357532	rs2162455	5.542e-06	missing
17	43804772	chr17:43804772:D	5.791e-06	CRHR1
2	185355695	rs3902438	5.88e-06	missing
2	185369649	rs1867187	6.145e-06	missing
5	7327172	rs10512915	6.872e-06	missing
13	64881954	rs9540044	7.021e-06	missing
2	185368838	rs1348339	7.085e-06	missing
11	125838912	rs603564	7.465e-06	CDON
5	7301424	rs114043041	8.075e-06	LOC442132
19	42764352	rs57938324	8.136e-06	missing

8	21298535	rs734364	9.019e-06	missing
7	143814570	rs73728210	9.61e-06	missing

Manhattan Plot:

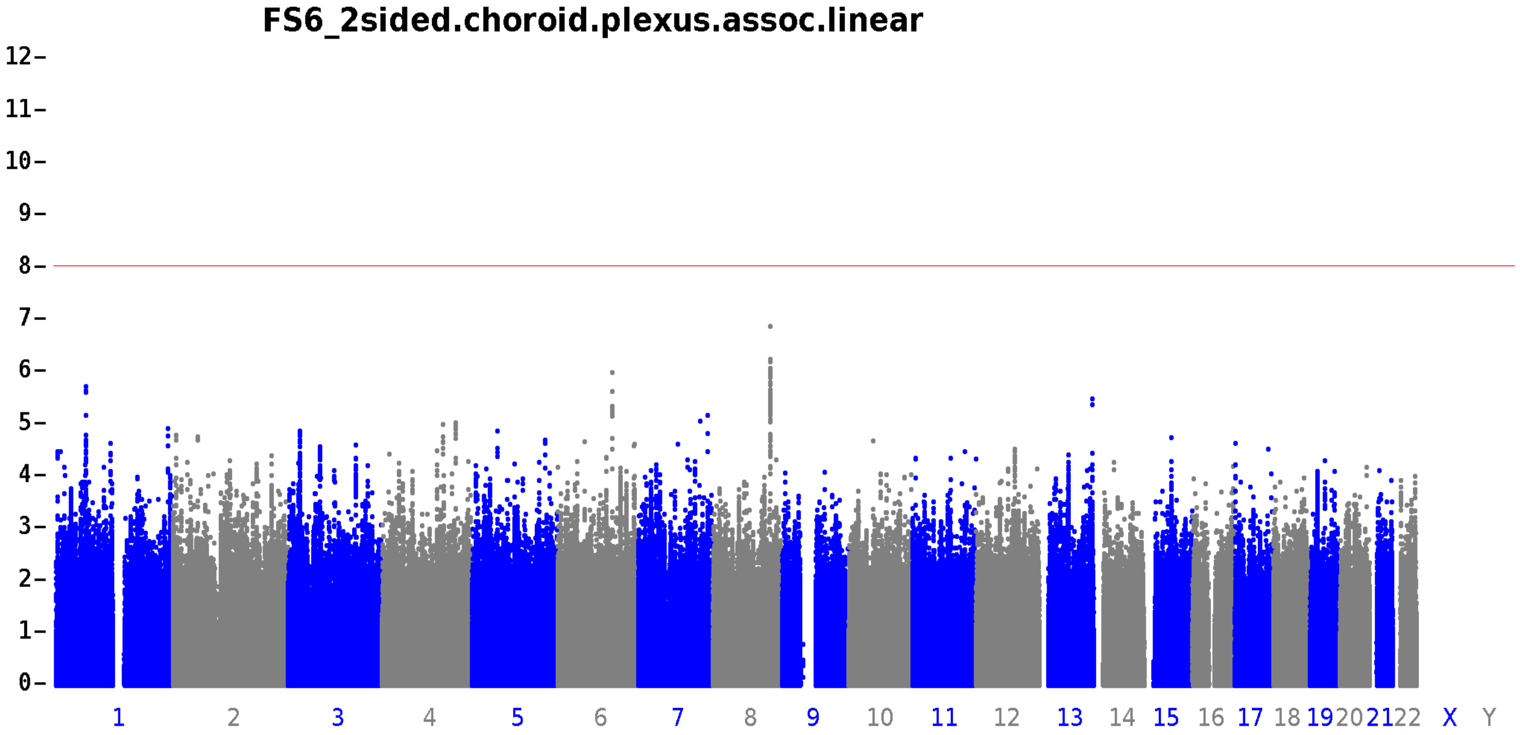

SNP mapped to gene:

CHR	BP	SNP	P	GENE
8	120913885	rs7465612	1.259e-07	DEPTOR
8	120910787	rs10103660	5.584e-07	DEPTOR
8	120913860	rs7465609	6.017e-07	DEPTOR
8	120910783	rs35272074	8.24e-07	DEPTOR
8	120916651	rs9721042	9.375e-07	DEPTOR
8	120908308	chr8:120908308:D	9.837e-07	DEPTOR
8	120900180	chr8:120900180:I	9.86e-07	DEPTOR
6	116389977	rs12529273	9.944e-07	FRK
8	120916394	rs7461290	1.085e-06	DEPTOR
8	120916555	rs9721029	1.219e-06	DEPTOR
8	120910235	rs6469865	1.543e-06	DEPTOR
8	120909628	rs7814496	1.593e-06	DEPTOR
8	120917902	rs12545854	1.71e-06	DEPTOR
1	63716841	rs79038692	1.84e-06	LINC00466
8	120914432	rs13277947	2.089e-06	DEPTOR
8	120914527	rs13277992	2.089e-06	DEPTOR
8	120914908	rs13253140	2.089e-06	DEPTOR
8	120914270	rs13279398	2.089e-06	DEPTOR
8	120915862	rs12681402	2.089e-06	DEPTOR
8	120913357	rs9649947	2.089e-06	DEPTOR
8	120912846	rs10093230	2.089e-06	DEPTOR
8	120925621	rs7824545	2.102e-06	DEPTOR
8	120918923	rs7006905	2.107e-06	DEPTOR
8	120917771	rs7462302	2.107e-06	DEPTOR

8	120917388	rs4073560	2.107e-06	DEPTOR
8	120918748	rs7002839	2.107e-06	DEPTOR
8	120916840	rs9720591	2.107e-06	DEPTOR
8	120921841	rs6469868	2.122e-06	DEPTOR
8	120922079	rs6469871	2.122e-06	DEPTOR
8	120922247	rs6469872	2.122e-06	DEPTOR
8	120922013	rs6469870	2.122e-06	DEPTOR
8	120922397	rs6995139	2.122e-06	DEPTOR
8	120921412	rs6469867	2.122e-06	DEPTOR
8	120923183	rs10217077	2.122e-06	DEPTOR
8	120920654	rs13267896	2.122e-06	DEPTOR
8	120924270	rs939242	2.122e-06	DEPTOR
8	120925083	rs10216503	2.122e-06	DEPTOR
1	63712850	rs75342058	2.166e-06	LINC00466
8	120919975	rs13265546	2.196e-06	DEPTOR
8	120922341	rs7015470	2.198e-06	DEPTOR
6	116316676	rs58604737	2.283e-06	FRK
1	63662830	rs75198730	2.325e-06	LINC00466
8	120924537	rs939241	2.467e-06	DEPTOR
8	120915232	rs11777705	2.539e-06	DEPTOR
8	120898765	rs13260088	2.599e-06	DEPTOR
8	120897501	rs7387694	2.599e-06	DEPTOR
8	120923285	rs10217083	2.642e-06	DEPTOR
8	120909157	chr8:120909157:D	2.662e-06	DEPTOR
8	120907420	rs13261304	2.687e-06	DEPTOR
8	120908921	rs10098306	2.847e-06	DEPTOR
8	120890586	rs13267652	2.969e-06	DEPTOR
8	120909780	rs7832923	3.028e-06	DEPTOR
8	120909913	rs6469861	3.058e-06	DEPTOR
8	120910150	rs6469863	3.058e-06	DEPTOR
8	120910225	rs6469864	3.058e-06	DEPTOR
8	120910036	rs6469862	3.058e-06	DEPTOR
8	120910380	rs6987580	3.058e-06	DEPTOR
8	120909493	rs13281299	3.058e-06	DEPTOR
8	120910538	rs6988011	3.058e-06	DEPTOR
8	120908886	rs7459671	3.058e-06	DEPTOR
8	120911630	rs6993375	3.058e-06	DEPTOR
8	120908607	rs7462250	3.058e-06	DEPTOR
8	120911645	rs6993797	3.058e-06	DEPTOR
8	120908589	rs7465181	3.058e-06	DEPTOR
8	120912154	rs6989741	3.058e-06	DEPTOR
8	120912233	rs13257252	3.058e-06	DEPTOR
8	120907990	rs4871013	3.093e-06	DEPTOR
8	120907974	rs4871012	3.093e-06	DEPTOR
8	120907895	rs4871772	3.093e-06	DEPTOR
13	111933998	rs7983485	3.175e-06	ARHGEF7
8	120907250	rs13260933	3.216e-06	DEPTOR

8	120907911	rs4871773	3.227e-06	DEPTOR
8	120939436	rs7814294	3.277e-06	DEPTOR
8	120940206	rs10808505	3.277e-06	DEPTOR
8	120909836	rs7818471	3.408e-06	DEPTOR
8	120925998	rs7387264	3.468e-06	DEPTOR
8	120934569	rs10107579	3.638e-06	DEPTOR
8	120887566	rs10110223	4.022e-06	DEPTOR
13	111937652	rs7985658	4.045e-06	ARHGEF7
8	120906391	rs13250594	4.09e-06	DEPTOR
8	120936418	rs4871787	4.147e-06	DEPTOR
8	120937041	rs1464276	4.147e-06	DEPTOR
8	120938448	rs6469878	4.208e-06	DEPTOR
8	120935452	rs2037346	4.213e-06	DEPTOR
8	120907183	rs13259990	4.24e-06	DEPTOR
8	120926153	rs7388508	4.247e-06	DEPTOR
8	120929289	rs1607624	4.247e-06	DEPTOR
6	116406419	rs17077529	4.307e-06	missing
8	120909665	rs7814520	4.428e-06	DEPTOR
6	116403785	rs1115813	4.665e-06	missing
8	120889242	rs11781657	4.701e-06	DEPTOR
8	120902486	rs10955940	4.703e-06	DEPTOR
8	120903856	rs12681623	4.703e-06	DEPTOR
8	120933963	rs9987332	4.704e-06	DEPTOR
8	120888752	rs7003126	4.754e-06	DEPTOR
8	120886545	rs13263296	4.984e-06	DEPTOR
6	116414389	rs140117512	5.011e-06	missing
8	120887041	rs1467044	5.248e-06	DEPTOR
8	120905919	rs13249122	5.31e-06	DEPTOR
8	120904009	rs13265367	5.455e-06	DEPTOR
6	116398199	rs17077512	5.528e-06	missing
6	116397233	chr6:116397233:D	5.533e-06	FRK
8	120929834	rs7829901	5.839e-06	DEPTOR
8	120903511	rs10955941	5.986e-06	DEPTOR
6	116318706	rs74987153	6.158e-06	FRK
8	120918111	rs12545863	6.175e-06	DEPTOR
8	120904800	rs10094587	6.374e-06	DEPTOR
8	120904688	rs10094458	6.374e-06	DEPTOR
8	120903734	rs13257316	6.395e-06	DEPTOR
8	120903070	rs10103058	6.395e-06	DEPTOR
1	63667885	rs79376841	6.474e-06	LINC00466
6	116387168	rs7453982	6.533e-06	FRK
6	116382997	rs2095795	6.533e-06	FRK,LOC101927818
6	116369110	rs12524293	6.533e-06	FRK,LOC101927818
7	147428505	rs76984521	6.583e-06	CNTNAP2
7	147428506	rs77834497	6.583e-06	CNTNAP2
6	116374207	rs79604852	6.745e-06	FRK,LOC101927818
6	116345430	rs117302099	6.833e-06	FRK

6	116344360	rs75482103	6.833e-06	FRK
8	120912429	rs13258592	7.789e-06	DEPTOR
7	131796548	rs7793511	8.343e-06	missing
8	120887547	rs10110216	8.573e-06	DEPTOR
4	156171545	rs11934271	8.972e-06	missing
4	156177738	rs2341896	9.384e-06	missing
4	130030199	rs28643269	9.658e-06	C4orf33

Manhattan Plot:

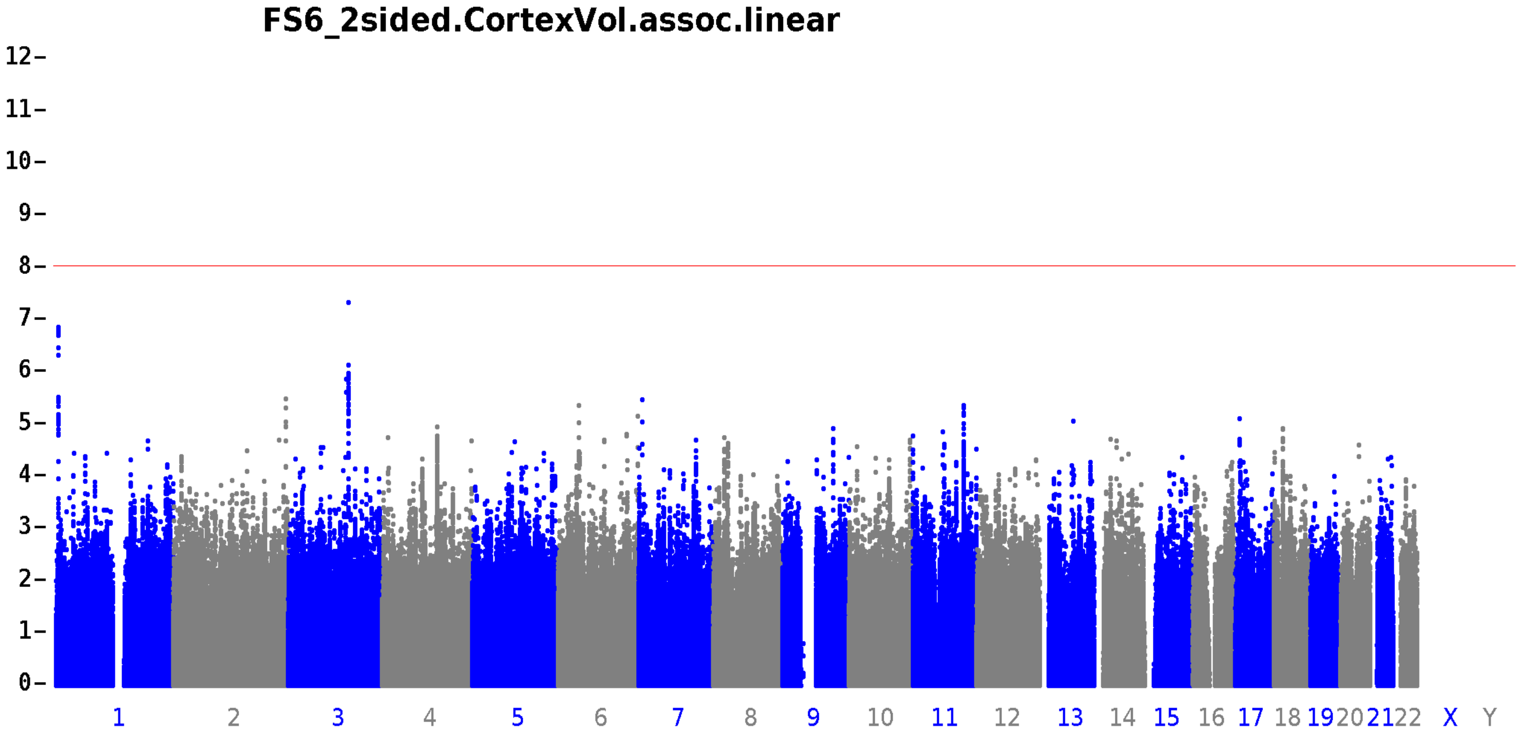

SNP mapped to gene:

CHR	BP	SNP	P	GENE
3	128650296	chr3:128650296:I	4.428e-08	LOC100132731
1	5337271	rs61759358	1.33e-07	missing
1	5341689	rs57925978	1.525e-07	missing
1	5341784	rs17423575	1.525e-07	missing
1	5339531	rs66495077	1.642e-07	missing
1	5341289	rs58812660	1.676e-07	missing
1	5341248	rs17457420	1.676e-07	missing
1	5341296	rs61558245	1.676e-07	missing
1	5340869	rs56278800	1.676e-07	missing
1	5339744	rs56056508	1.821e-07	missing
1	5341819	chr1:5341819:D	1.879e-07	missing
1	5341464	rs17423547	3.24e-07	missing
1	5340385	rs12143953	4.527e-07	missing
3	128585715	rs56063711	6.994e-07	LOC653712
3	128595795	rs9825854	1.034e-06	missing
3	128592457	rs6768014	1.039e-06	missing
3	128570263	rs16851607	1.169e-06	missing
3	128595514	rs9825119	1.295e-06	missing
3	128596734	chr3:128596734:I	1.295e-06	missing
3	128594373	rs9847759	1.295e-06	missing
3	128592669	rs6795293	1.295e-06	missing
3	128593259	rs6796031	1.295e-06	missing
3	122683207	rs12635724	1.33e-06	SEMA5B
3	128652553	rs9810890	1.584e-06	LOC100132731

3	128591267	rs6789094	1.856e-06	missing
3	128609255	rs13434201	1.934e-06	ACAD9
3	128647823	rs6778057	2.039e-06	LOC100132731
3	128641995	chr3:128641995:D	2.039e-06	LOC100132731
3	128602135	rs13323787	2.259e-06	ACAD9
3	128585471	rs60876670	2.326e-06	LOC653712
3	128587818	rs6796590	2.326e-06	LOC653712
3	128587629	rs6808675	2.326e-06	LOC653712
3	128590405	rs9826700	2.326e-06	missing
3	122683171	rs12630339	2.386e-06	SEMA5B
3	128581001	rs11921456	2.565e-06	LOC653712
3	128575365	rs28694035	2.565e-06	missing
3	128583073	rs11923686	2.565e-06	LOC653712
3	128575353	rs28675843	2.565e-06	missing
3	128583226	rs78115835	2.565e-06	LOC653712
3	128572314	rs9862461	2.565e-06	missing
3	128583514	rs68127468	2.565e-06	LOC653712
3	128571735	rs13322981	2.565e-06	MARK2P6
3	128588632	rs4927923	2.587e-06	LOC653712,LOC101927197
3	128571974	rs13323780	2.813e-06	missing
1	5341840	rs12738436	2.919e-06	missing
1	5338212	rs17457350	2.934e-06	missing
2	238236921	rs6720773	3.118e-06	COL6A3
3	128623785	rs61096019	3.16e-06	ACAD9
7	8796378	rs10486254	3.271e-06	missing
1	5341078	rs12733390	3.368e-06	missing
1	5340818	rs35263239	3.368e-06	missing
1	5340730	rs35556848	3.621e-06	missing
3	128595566	chr3:128595566:I	3.87e-06	missing
6	45631530	rs74697776	4.141e-06	missing
6	45625396	rs76428039	4.157e-06	missing
6	45623095	rs74460375	4.157e-06	missing
3	128649081	rs9869254	4.165e-06	LOC100132731
3	128659244	rs7632015	4.245e-06	LOC100132731
11	107262510	rs11212197	4.253e-06	CWF19L2
11	107275251	rs11212206	4.254e-06	CWF19L2,SMARCE1P1
3	128662213	chr3:128662213:I	4.275e-06	LOC100132731
1	5341622	rs35522643	4.37e-06	missing
11	107155911	rs7929345	4.728e-06	missing
2	238238480	rs6722463	4.751e-06	COL6A3
3	128572219	rs9862012	5.189e-06	missing
11	107155909	rs7929343	5.755e-06	missing
11	107276281	chr11:107276281:D	5.823e-06	CWF19L2
3	128661260	rs72977135	6.017e-06	LOC100132731
1	5331673	rs4290073	6.341e-06	missing
11	107278683	rs17106875	6.42e-06	CWF19L2
6	170339227	rs73790307	6.86e-06	missing

1	5342240	rs12142741	7.327e-06	missing
1	5342295	rs12141954	7.373e-06	missing
17	8968500	rs9910696	7.477e-06	NTN1
1	5342310	rs12141957	7.671e-06	missing
3	128647141	rs56000731	8.46e-06	LOC100132731
13	71646254	rs1467627	8.498e-06	missing
1	5337936	rs67958015	8.619e-06	missing
7	8794010	rs10238890	8.657e-06	missing
2	238237917	rs13018217	8.75e-06	COL6A3
6	45633623	rs61433014	9e-06	missing
11	107252208	rs10502087	9.245e-06	CWF19L2
3	128584270	rs9842450	9.552e-06	LOC653712
1	5335170	rs55729623	9.603e-06	missing
1	5333919	rs56260836	9.603e-06	missing

Manhattan Plot:

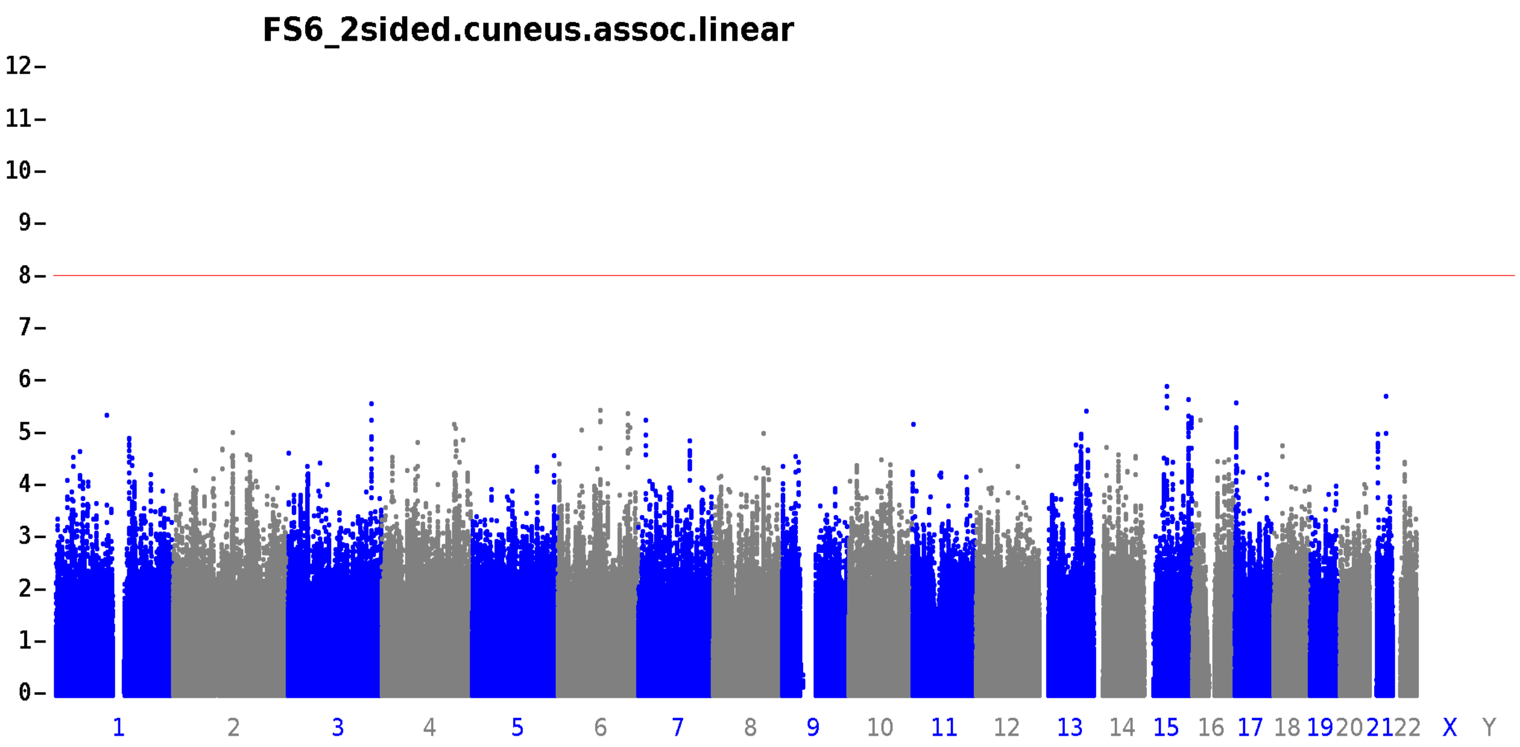

SNP mapped to gene:

CHR	BP	SNP	P	GENE
15	47901737	rs10152500	1.165e-06	SEMA6D
15	47903174	rs74014029	1.811e-06	SEMA6D
21	34354062	rs8132517	1.818e-06	missing
15	94225516	rs6497102	2.079e-06	missing
17	3295368	rs162253	2.453e-06	missing
3	176560943	rs75444093	2.542e-06	missing
15	47902502	rs28431305	3.084e-06	SEMA6D
6	90609191	rs62416030	3.369e-06	missing
13	99894406	chr13:99894406:I	3.532e-06	UBAC2
6	148999454	rs9498113	3.948e-06	missing
1	108662072	rs17021001	4.171e-06	missing
15	94224823	rs6497099	4.302e-06	missing
15	100735352	rs8025049	4.65e-06	ADAMTS17
7	16943785	rs77016575	5.166e-06	missing
16	16985541	rs74009651	5.271e-06	missing
3	176562244	rs80032394	5.284e-06	missing
15	100735967	rs1038235	5.441e-06	ADAMTS17
6	90611858	rs62416032	5.512e-06	missing
6	90611116	rs149830477	5.664e-06	missing
15	100735066	rs8041050	6.121e-06	ADAMTS17
15	94227636	rs4497634	6.152e-06	missing
15	94227446	rs4617803	6.152e-06	missing
15	94226879	rs8031833	6.279e-06	missing
15	94226725	rs4315296	6.279e-06	missing

4	152854258	rs360938	6.335e-06	missing
11	2324347	rs55648810	6.371e-06	TSPAN32
6	149003411	rs4897035	6.523e-06	missing
15	94227065	rs8033314	7.041e-06	missing
15	100738145	rs7179412	7.158e-06	ADAMTS17
17	3288545	rs2170686	7.207e-06	missing
6	153384196	rs1933262	7.374e-06	RGS17
15	94228165	rs8039749	7.389e-06	missing
17	3285471	rs1067094	7.518e-06	missing
4	156145116	rs79892985	7.61e-06	missing
6	52090505	rs34554839	8.002e-06	missing
6	149018925	rs4897038	8.695e-06	missing
17	3311365	rs162255	8.878e-06	missing
15	94233410	rs4598857	8.879e-06	missing
2	126612987	rs72847150	9.11e-06	missing
21	34353993	rs12106324	9.293e-06	missing
8	107089603	rs7833595	9.478e-06	missing
21	16362856	rs2822997	9.705e-06	NRIP1
13	89225353	rs9521352	9.769e-06	missing

Manhattan Plot:

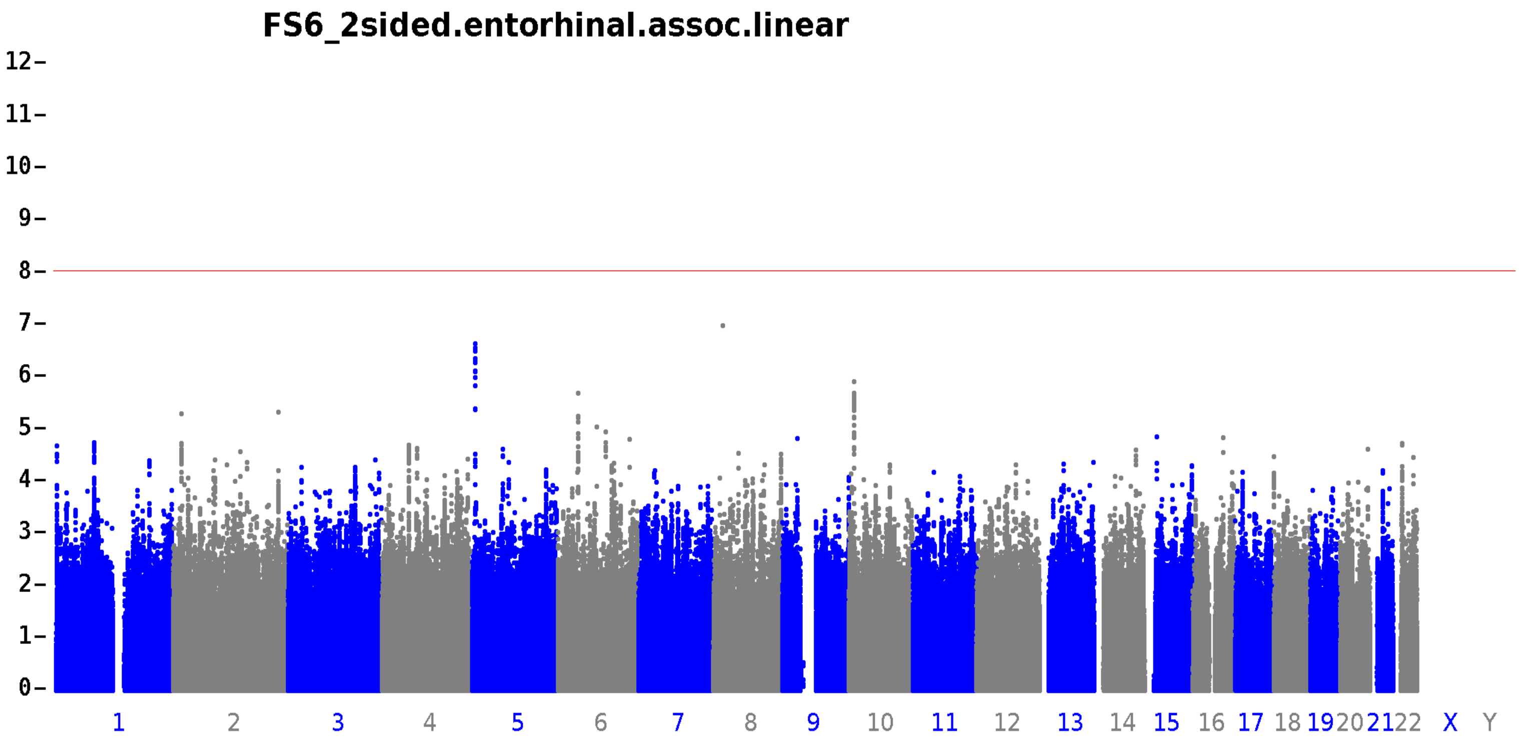

SNP mapped to gene:

CHR	BP	SNP	P	GENE
8	20720454	rs2616222	1.008e-07	missing
5	5603321	chr5:5603321:D	2.189e-07	missing
5	5608380	rs385588	2.612e-07	missing
5	5604193	rs2448442	3.115e-07	missing
5	5603889	rs2438478	3.115e-07	missing
5	5604270	chr5:5604270:D	3.115e-07	missing
5	5603110	rs2918255	3.115e-07	missing
5	5604316	rs2455479	3.115e-07	missing
5	5602989	rs2918256	3.115e-07	missing
5	5604668	rs1438674	3.115e-07	missing
5	5602964	rs2918257	3.115e-07	missing
5	5604892	rs2438475	4.299e-07	missing
5	5604788	rs2438476	4.299e-07	missing
5	5602530	rs2438479	4.681e-07	missing
5	5605004	rs2438474	5.068e-07	missing
5	5605078	rs2448437	5.068e-07	missing
5	5605393	rs2448439	5.121e-07	missing
5	5605220	rs2438473	5.121e-07	missing
5	5605983	rs2438471	7.334e-07	missing
5	5606066	rs2455476	7.334e-07	missing
5	5605705	rs2455477	7.533e-07	missing
5	5605830	rs2438472	9.722e-07	missing
10	10152460	chr10:10152460:I	1.164e-06	missing
5	5602308	rs1318543	1.418e-06	missing

6	42901120	rs4714634	1.948e-06	CNPY3
10	10155884	rs1012131	1.968e-06	missing
10	10108251	rs2657518	2.241e-06	missing
10	10148821	rs2657508	2.388e-06	missing
10	10145565	rs2147298	2.388e-06	missing
10	10148233	rs2209299	2.402e-06	missing
10	10098628	rs2804074	2.52e-06	missing
10	10105587	rs2804069	2.705e-06	missing
10	10097817	rs2181855	2.806e-06	missing
10	10102240	rs2804071	2.819e-06	LOC101928298
10	10137893	rs2023582	2.956e-06	missing
10	10105358	rs2657519	3.006e-06	LOC101928298
10	10095395	rs2804078	3.167e-06	missing
10	10099425	rs2025469	3.317e-06	missing
10	10107590	rs2021854	3.353e-06	missing
5	5604163	rs2578542	3.919e-06	missing
10	10103089	rs2657522	3.972e-06	LOC101928298
5	5607865	rs252601	4.04e-06	missing
10	10106965	chr10:10106965:I	4.173e-06	missing
10	10107267	rs2804068	4.173e-06	missing
2	222384480	rs2710511	4.532e-06	EPHA4
2	16952522	chr2:16952522:I	4.808e-06	missing
6	42903766	rs1053539	5.357e-06	CNPY3
10	10098650	rs2657525	5.587e-06	missing
10	10142076	rs2762652	5.831e-06	missing
6	42903763	rs1053538	5.872e-06	CNPY3
6	42915920	rs6941212	7.091e-06	missing
10	10129279	rs2804061	8.078e-06	missing
6	83615698	rs12195720	8.553e-06	UBE3D

Manhattan Plot:

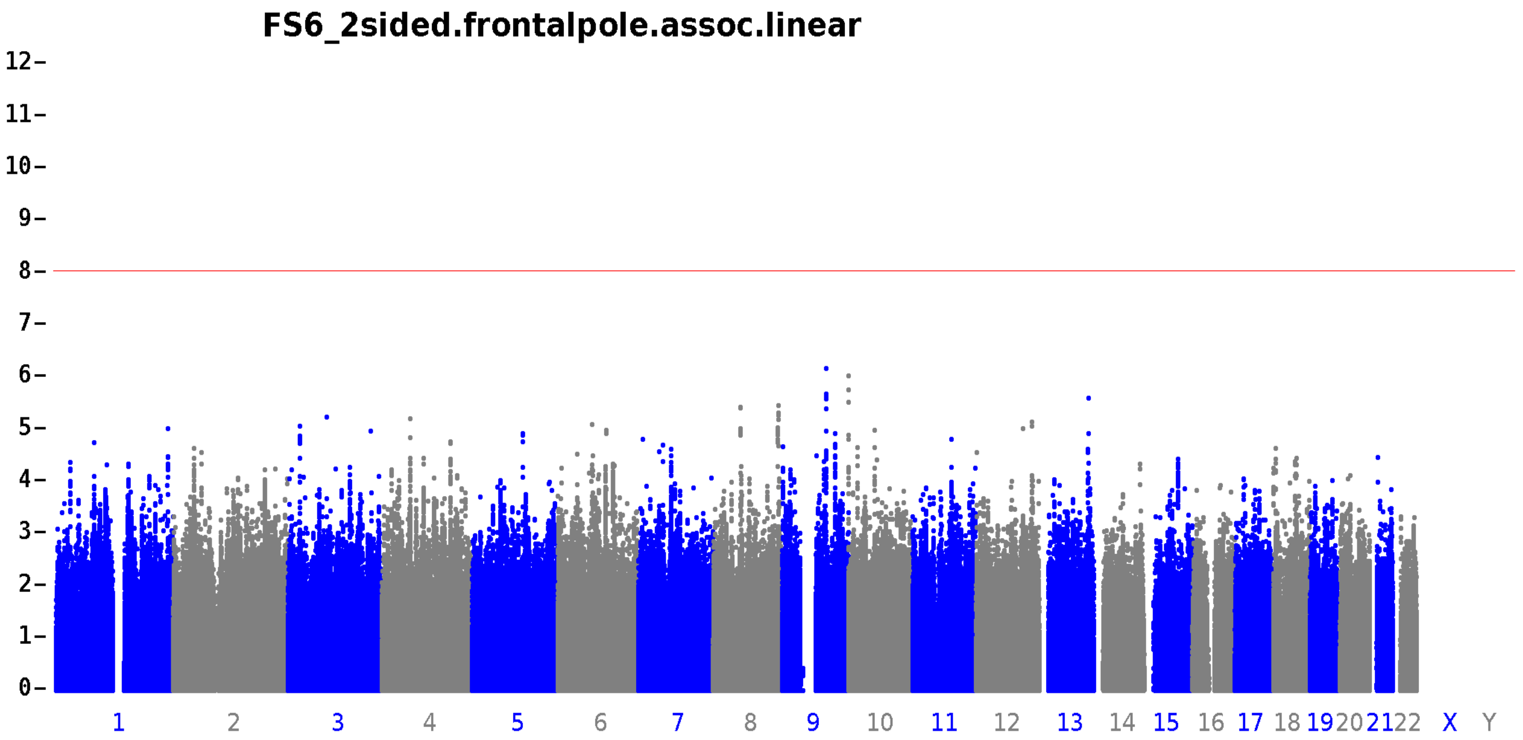

SNP mapped to gene:

CHR	BP	SNP	P	GENE
9	93607472	rs2387339	6.484e-07	SYK
10	468183	rs12254690	9.155e-07	DIP2C
10	470628	rs7070162	1.673e-06	DIP2C
9	93608702	rs2259147	2.035e-06	SYK
9	93612776	rs12380445	2.082e-06	SYK
9	93611737	rs10993711	2.203e-06	SYK
9	93611186	rs3789898	2.203e-06	SYK
9	93610132	rs1993704	2.203e-06	SYK
9	93609773	rs2387337	2.203e-06	SYK
13	104525390	rs2184795	2.418e-06	missing
9	93613551	rs17489429	2.492e-06	SYK
10	471881	rs71489246	2.908e-06	DIP2C
8	137832397	rs3907169	3.334e-06	missing
8	57542912	rs16920884	3.638e-06	missing
8	57541988	rs16920883	3.798e-06	missing
9	93611900	rs10993712	3.886e-06	SYK
8	137790982	rs12677075	4.761e-06	missing
8	137802892	rs3850501	5.2e-06	missing
3	82495988	rs7644123	5.538e-06	missing
4	59513357	rs28551064	6.113e-06	missing
4	59512470	rs17089520	6.113e-06	missing
8	137812496	rs73379436	6.186e-06	missing
12	117813981	rs492623	7.113e-06	missing
6	73681783	rs9360623	7.779e-06	KCNQ5

12	117828081	rs10850817	8.296e-06	missing
3	24793811	rs4241533	8.422e-06	missing
8	137715422	rs57533972	8.911e-06	missing
1	238512657	rs7527000	9.262e-06	missing
8	57557056	rs116377309	9.335e-06	missing
12	98808144	rs112650478	9.475e-06	missing
8	137713790	rs10108144	9.743e-06	missing
6	104243992	rs68024227	9.921e-06	missing
8	137734494	rs3911696	9.987e-06	missing

Manhattan Plot:

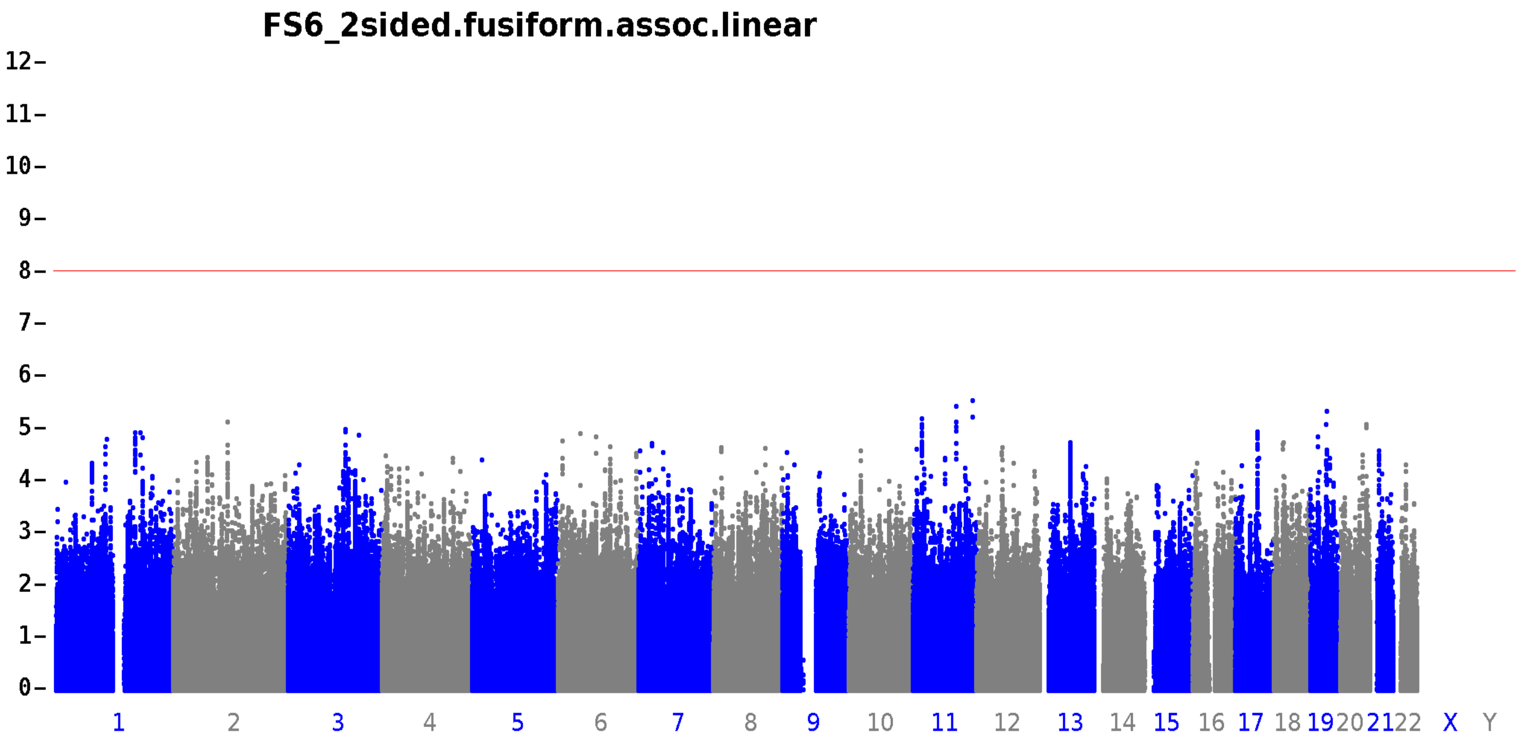

SNP mapped to gene:

CHR	BP	SNP	P	GENE
11	127058783	rs7104858	2.739e-06	missing
11	91260529	rs72963340	3.479e-06	missing
11	91266136	rs12416830	3.555e-06	missing
11	91265942	rs16916172	3.555e-06	missing
19	34497648	rs75234213	4.399e-06	missing
11	127058947	rs7121129	5.677e-06	missing
11	18677811	rs35024874	6.073e-06	missing
11	91259703	rs12575271	7.039e-06	missing
2	114528761	rs62170102	7.076e-06	missing
11	18689168	rs11607934	7.728e-06	missing
19	33915521	rs73588216	7.894e-06	PEPD
20	55508541	rs62208758	7.916e-06	missing
11	18684645	rs145476855	8.07e-06	missing
11	91270930	rs72963350	8.574e-06	missing
11	91276225	rs10830751	8.589e-06	missing
11	18686916	rs9705036	8.965e-06	LOC100506569
20	55505912	rs62208757	9.157e-06	missing
3	122580259	rs75250529	9.568e-06	DIRC2
3	122577959	rs4377440	9.568e-06	DIRC2
3	122581330	rs75436330	9.568e-06	DIRC2
3	122577456	chr3:122577456:D	9.568e-06	DIRC2

Manhattan Plot:

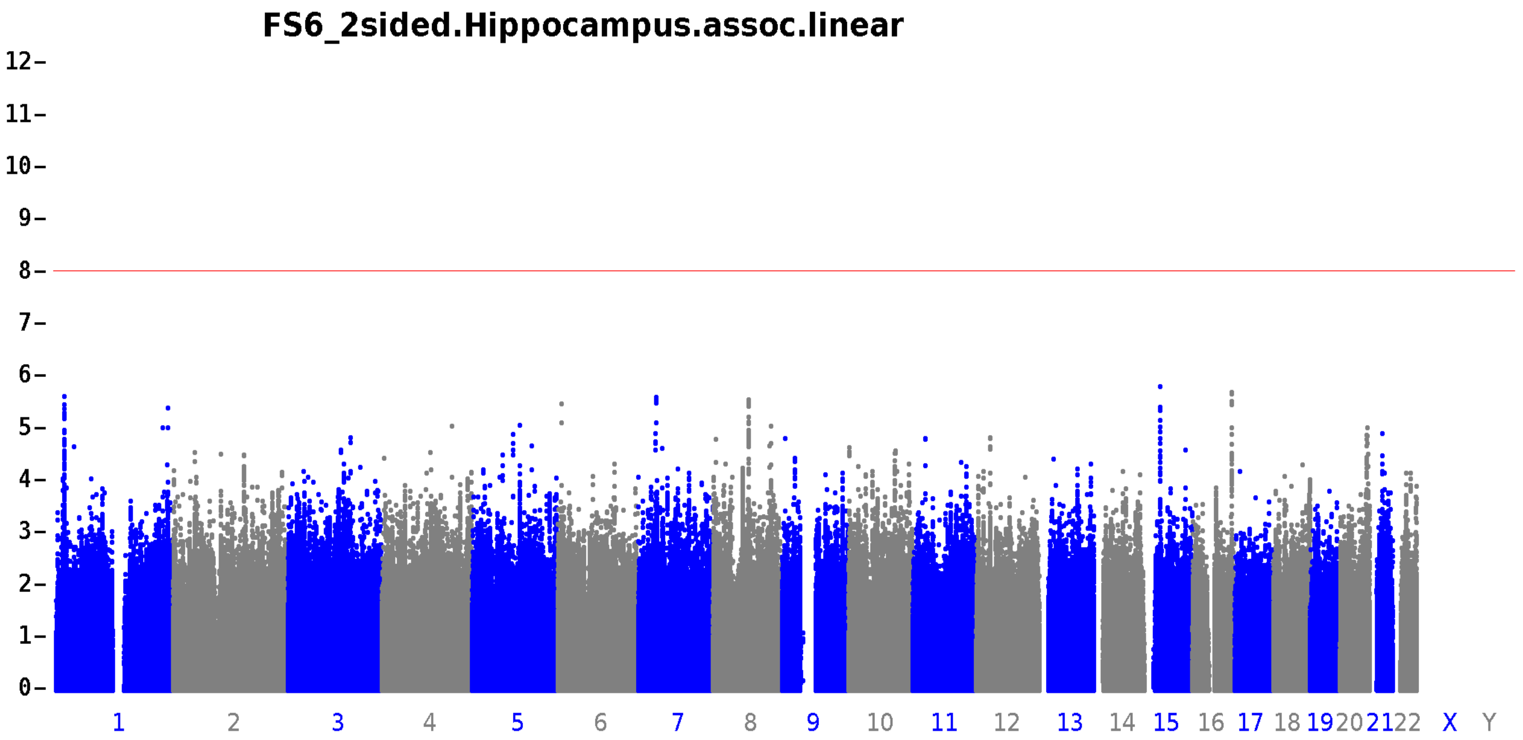

SNP mapped to gene:

CHR	BP	SNP	P	GENE
15	34132388	rs72716818	1.458e-06	RYR3
16	83796216	rs1345850	1.867e-06	CDH13
16	83794234	rs877920	2.026e-06	CDH13
1	18374725	rs4920415	2.298e-06	missing
7	38176373	rs6969314	2.38e-06	missing
8	75295333	rs12546775	2.64e-06	missing
7	38174182	rs56356532	2.662e-06	missing
16	83795483	rs1833972	2.793e-06	CDH13
7	38168016	rs1568755	3.027e-06	missing
8	75272727	rs4310196	3.071e-06	GDAP1
8	75270494	rs4410896	3.086e-06	GDAP1
6	9393712	rs214438	3.162e-06	missing
1	18359117	chr1:18359117:I	3.251e-06	missing
16	83795059	rs880639	3.267e-06	CDH13
8	75269548	rs13254381	3.457e-06	GDAP1
8	75269564	rs13256983	3.457e-06	GDAP1
15	34149609	rs61506923	3.593e-06	RYR3
1	237974720	rs813173	3.735e-06	RYR2
1	18357983	rs11203263	3.882e-06	missing
15	34149034	rs111585987	4.178e-06	RYR3
1	18369434	rs6586493	4.63e-06	missing
1	18359901	rs7553424	5.413e-06	missing
8	75292492	rs6996971	5.551e-06	missing
1	18369645	rs7535081	6.099e-06	missing

15	34137079	chr15:34137079:D	6.424e-06	RYR3
8	75312672	rs141149852	6.982e-06	missing
6	9397714	rs214442	7.163e-06	missing
7	38175133	chr7:38175133:D	7.253e-06	missing
8	75301253	rs10108295	7.278e-06	missing
8	75297342	rs7813767	7.503e-06	missing
8	75297062	rs10098296	7.503e-06	missing
8	75298201	rs4305896	7.503e-06	missing
5	101392184	rs28535886	8.175e-06	missing
4	149035449	rs1879829	8.32e-06	NR3C2
8	122217004	rs6469996	8.541e-06	missing
15	34146886	rs713201	8.791e-06	RYR3
1	226523560	rs11800726	8.9e-06	missing
20	58681414	rs56738889	8.963e-06	LOC729296
16	83794986	rs889406	8.988e-06	CDH13
16	83794991	rs880640	8.988e-06	CDH13
1	237974849	rs790886	9.123e-06	RYR2
8	75293712	rs4567029	9.889e-06	missing
1	18360070	rs7530554	9.952e-06	missing

Manhattan Plot:

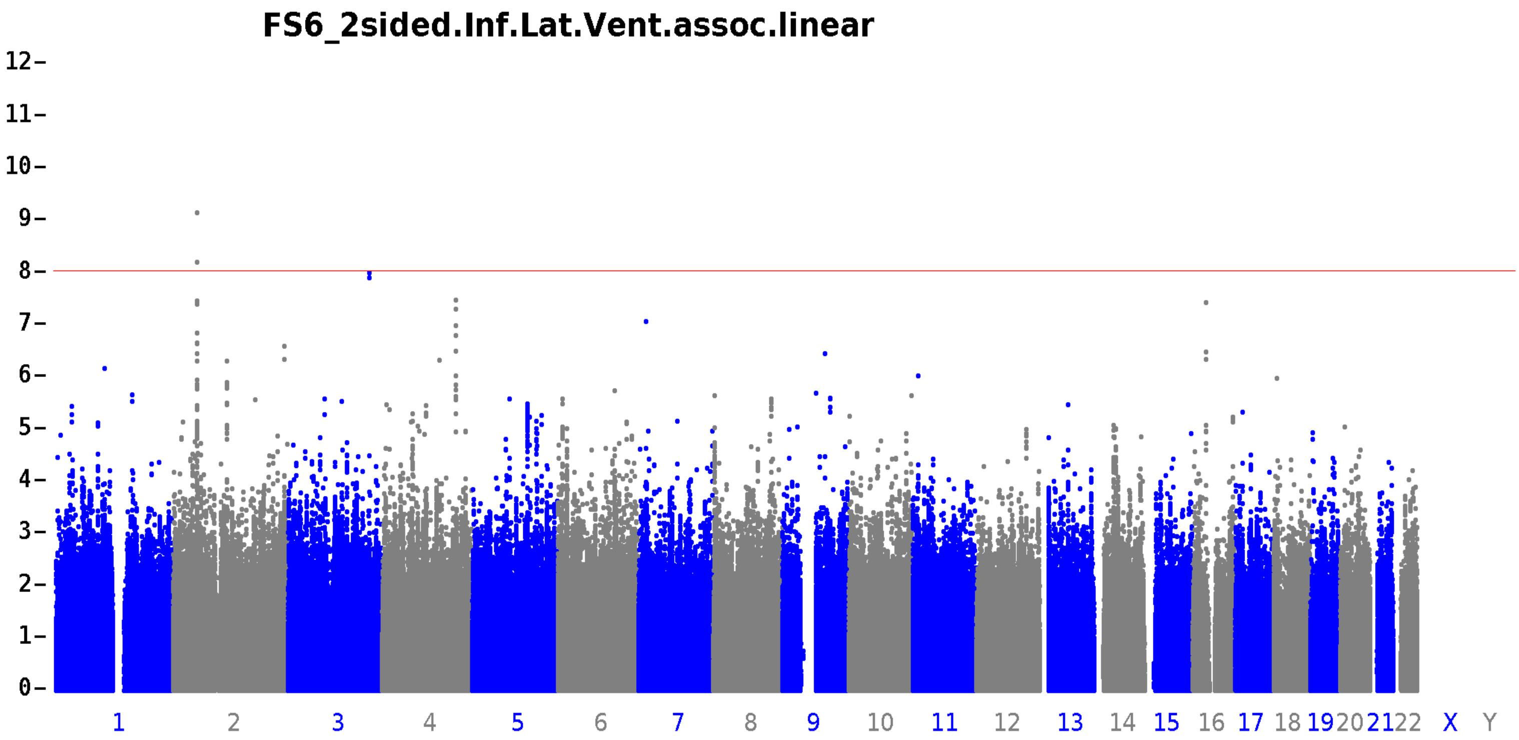

SNP mapped to gene:

CHR	BP	SNP	P	GENE
2	50368229	rs12467877	6.761e-10	NRXN1
2	50408397	rs76189321	6.097e-09	NRXN1
3	172659246	rs10440041	9.654e-09	SPATA16
3	172660306	chr3:172660306:I	1.218e-08	SPATA16
4	156265336	rs75174989	3.196e-08	MAP9
2	50494711	rs80262609	3.325e-08	NRXN1
16	27890361	chr16:27890361:I	3.633e-08	GSG1L
2	50474429	rs17463187	3.847e-08	NRXN1
4	156263122	rs17032846	4.835e-08	missing
7	16509086	rs1981596	8.243e-08	missing
4	156298461	rs76910738	9.834e-08	missing
4	156295899	rs116705331	9.834e-08	MAP9
2	50497563	rs12476988	1.394e-07	NRXN1
4	156302215	rs79397405	1.529e-07	missing
2	50439966	rs17462099	2.097e-07	NRXN1
2	50439473	rs17508400	2.097e-07	NRXN1
2	50438729	rs17461897	2.097e-07	NRXN1
2	50442052	rs17508684	2.205e-07	NRXN1
2	50438398	chr2:50438398:D	2.207e-07	NRXN1
2	234551413	rs7569014	2.506e-07	UGT1A,UGT1A8,
4	156198980	chr4:156198980:I	3.077e-07	missing
16	27902356	rs80121937	3.171e-07	GSG1L
2	50436716	rs17461653	3.462e-07	NRXN1
9	90671189	rs79516263	3.463e-07	LOC101927680

2	234538232	rs28948385	4.342e-07	UGT1A,UGT1A8
16	27902011	rs57728459	4.469e-07	GSG1L
4	122218977	rs56272075	4.561e-07	missing
2	113853350	rs1665189	4.773e-07	missing
2	50437687	rs116262844	4.778e-07	NRXN1
1	103507114	rs58975794	6.565e-07	COL11A1
11	10722738	rs73415773	9.036e-07	missing
4	156259032	rs996602	9.061e-07	missing
18	7585354	rs4798575	1.013e-06	PTPRM
2	50366332	rs113635677	1.092e-06	NRXN1
2	50478139	rs77771770	1.106e-06	NRXN1
2	113857606	rs62158848	1.24e-06	missing
2	113841968	rs55932259	1.326e-06	missing
2	113845505	rs66613854	1.326e-06	missing
2	50465217	rs111626088	1.329e-06	NRXN1
2	50435575	rs78276107	1.336e-06	NRXN1
4	156173251	rs17032726	1.362e-06	missing
2	50360398	rs17445771	1.416e-06	NRXN1
2	113849986	rs12475887	1.447e-06	missing
2	113848123	rs57130564	1.447e-06	missing
2	113847237	rs66615594	1.447e-06	missing
2	113858495	rs1688076	1.471e-06	missing
2	113855774	rs1618084	1.471e-06	missing
2	113858196	rs1688075	1.471e-06	missing
2	113839286	rs4848314	1.6e-06	missing
2	50370168	rs79934676	1.627e-06	NRXN1
4	156291460	rs719688	1.71e-06	MAP9
6	120950372	rs9490015	1.729e-06	missing
9	71931388	rs11138118	1.94e-06	missing
1	162109787	rs12116968	2.093e-06	NOS1AP
8	3334338	rs10110203	2.148e-06	CSMD1
10	131988165	rs11017125	2.205e-06	missing
4	156277631	rs17032869	2.266e-06	MAP9
4	156163302	rs1444800	2.38e-06	missing
4	156164155	rs80155656	2.38e-06	missing
9	101807295	rs77060722	2.453e-06	COL15A1
9	101807404	rs60891605	2.484e-06	COL15A1
5	78787249	rs57027572	2.485e-06	HOMER1
3	77856623	rs3852018	2.486e-06	missing
6	10270149	rs6918084	2.515e-06	missing
8	122217004	rs6469996	2.515e-06	missing
2	173764392	rs2553014	2.618e-06	RAPGEF4
4	156147798	rs77324586	2.64e-06	missing
8	122224091	chr8:122224091:I	2.761e-06	missing
3	113614236	rs73230239	2.783e-06	GRAMD1C
1	162107205	chr1:162107205:I	2.87e-06	NOS1AP
8	122219251	rs10100336	2.993e-06	missing

8	122220095	rs6988785	2.993e-06	missing
8	122220866	rs61451379	2.993e-06	missing
8	122219336	rs10086962	2.993e-06	missing
8	122220900	rs57568346	2.993e-06	missing
8	122220943	rs58566656	2.993e-06	missing
8	122217196	rs7839878	2.993e-06	missing
8	122217575	rs73324943	3.025e-06	missing
8	122220771	rs6989823	3.037e-06	missing
2	113860196	rs1794071	3.076e-06	missing
6	10265001	rs150548007	3.137e-06	missing
5	116965615	rs419359	3.141e-06	missing
13	60153623	rs862728	3.237e-06	missing
4	8603795	rs10019092	3.241e-06	CPZ
5	116975794	chr5:116975794:I	3.249e-06	missing
2	113865367	rs315925	3.273e-06	missing
5	116965325	rs11489152	3.406e-06	missing
2	50491989	rs17463545	3.423e-06	NRXN1
4	93214774	chr4:93214774:D	3.424e-06	missing
1	34665647	rs1542555	3.507e-06	C1orf94
2	50426319	rs10490235	3.608e-06	NRXN1
2	50426615	rs76164269	3.608e-06	NRXN1
8	122224067	rs28516600	3.61e-06	missing
8	122225540	rs28712038	3.61e-06	missing
8	122224359	rs7814406	3.61e-06	missing
9	101806058	rs949802	3.658e-06	COL15A1
5	116976257	rs367474	3.852e-06	missing
2	50393986	rs12479013	3.876e-06	NRXN1
2	50460480	rs7602425	3.964e-06	NRXN1
4	16395889	rs143059626	4.01e-06	missing
2	50393852	rs12470857	4.013e-06	NRXN1
2	50393803	rs12478889	4.013e-06	NRXN1
8	122224085	chr8:122224085:I	4.121e-06	missing
17	15102214	rs72811147	4.492e-06	missing
5	116977050	rs382137	4.496e-06	missing
9	101805757	rs10988660	4.602e-06	COL15A1
4	93214779	chr4:93214779:D	4.614e-06	missing
5	116975426	rs371223	4.616e-06	missing
4	156299591	rs75214307	4.778e-06	missing
4	64322072	rs17628760	4.846e-06	missing
1	34660298	rs899379	4.977e-06	C1orf94
3	77842138	rs9824656	5.073e-06	missing
1	34663812	rs1478929	5.102e-06	C1orf94
1	34663147	rs1382603	5.102e-06	C1orf94
1	34662000	rs1612291	5.102e-06	C1orf94
1	34661652	rs1771362	5.102e-06	C1orf94
5	146675133	rs13170855	5.202e-06	STK32A
5	117026872	rs1526882	5.395e-06	missing

8	122224306	rs7814273	5.408e-06	missing
4	93214812	rs17019289	5.465e-06	missing
10	709840	rs45473196	5.512e-06	DIP2C,PRR26
5	122060452	rs149197958	5.637e-06	LOC101927379
16	85104129	rs56888354	5.659e-06	KIAA0513
16	85104172	rs59376162	5.659e-06	KIAA0513
16	85103953	rs61158014	6.035e-06	KIAA0513
5	116978639	rs437696	6.274e-06	missing
5	116978949	rs402589	6.274e-06	missing
5	116977767	rs387029	6.569e-06	missing
2	50386966	rs76559474	6.642e-06	NRXN1
7	83228793	rs10246409	6.645e-06	SEMA3E
4	64315456	rs66775783	6.708e-06	missing
5	135909159	rs7448234	6.748e-06	missing
1	34657223	rs2783983	6.968e-06	C1orf94
2	20436975	rs62111437	6.974e-06	missing
4	61183979	rs6820377	7.069e-06	missing
6	145419949	rs113367288	7.069e-06	missing
16	85104226	rs57882905	7.079e-06	KIAA0513
6	145414905	rs75515233	7.108e-06	missing
2	50392150	rs57947630	7.127e-06	NRXN1
6	145416004	rs7758944	7.192e-06	missing
5	116970618	rs388878	7.29e-06	missing
5	116970213	rs372523	7.29e-06	missing
1	89592133	rs17130736	7.352e-06	missing
2	50392938	rs72881281	7.358e-06	NRXN1
5	116971784	rs373580	7.424e-06	missing
6	145417117	rs75678308	7.438e-06	missing
5	116967460	rs449687	7.641e-06	missing
5	146670908	rs34094800	7.695e-06	STK32A
1	89586518	rs10922572	7.765e-06	GBP2
5	116975184	rs447179	7.777e-06	missing
14	41965940	rs10131690	7.962e-06	missing
16	27897892	rs75850286	8.011e-06	GSG1L
1	89588521	rs7532145	8.046e-06	GBP2
1	89588300	rs7537582	8.046e-06	GBP2
2	50377832	rs10490237	8.132e-06	NRXN1
2	50375871	rs17505611	8.132e-06	NRXN1
2	113828425	rs9308682	8.231e-06	IL1F10
1	89584252	rs7531349	8.266e-06	GBP2
1	89578097	rs12068052	8.266e-06	GBP2
5	116966319	rs637744	8.341e-06	missing
5	116965479	rs386554	8.341e-06	missing
4	75197036	rs28423375	8.479e-06	missing
9	32410850	rs4442231	8.571e-06	ACO1
20	8299102	rs2745771	8.594e-06	PLCB1
6	10261644	rs11963800	8.687e-06	missing

2	50419759	rs113757726	8.858e-06	NRXN1
2	50414759	rs115267333	8.858e-06	NRXN1
2	50416322	rs114171136	8.858e-06	NRXN1
2	50414171	rs17447848	8.858e-06	NRXN1
2	50421383	rs79967285	8.858e-06	NRXN1
2	50413526	chr2:50413526:D	8.858e-06	NRXN1
2	50421580	rs77407791	8.858e-06	NRXN1
2	50411902	rs74737038	8.858e-06	NRXN1
2	50424877	rs10495982	8.858e-06	NRXN1
2	50411801	rs17447792	8.858e-06	NRXN1
2	113830626	rs4849151	9.137e-06	IL1F10
8	3333227	rs10106196	9.179e-06	CSMD1
2	113829522	rs4849148	9.216e-06	IL1F10
5	116965644	rs425650	9.225e-06	missing
14	46762470	rs111295476	9.284e-06	LINC00871
6	19353011	rs7773088	9.293e-06	missing
2	50418000	rs111273985	9.38e-06	NRXN1
2	50372726	rs17446158	9.422e-06	NRXN1
5	135880680	rs13176483	9.499e-06	missing
5	116974165	rs392457	9.672e-06	missing
9	14498096	rs58172969	9.741e-06	missing
12	104884438	rs10778336	9.756e-06	CHST11
14	46780110	rs4578561	9.868e-06	LINC00871

Manhattan Plot:

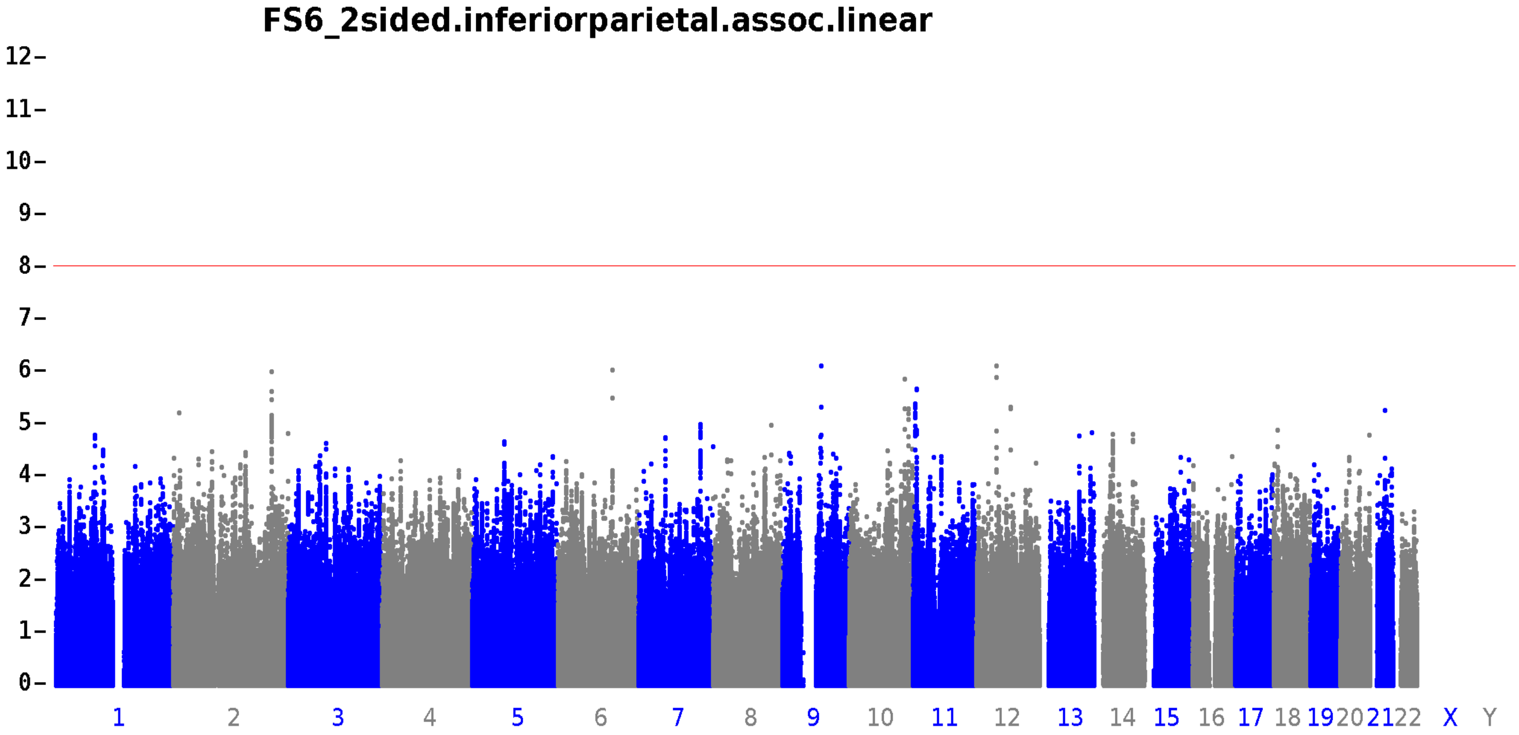

SNP mapped to gene:

CHR	BP	SNP	P	GENE
9	82622502	rs11138453	7.251e-07	LOC101927477
12	42973243	rs57566659	7.427e-07	PRICKLE1
6	116399822	rs6938248	8.726e-07	missing
2	209002493	rs77365020	9.462e-07	LOC100507443
12	42976838	chr12:42976838:D	1.202e-06	PRICKLE1
10	118696266	rs4751614	1.328e-06	KIAA1598
11	8782520	rs34599227	2.058e-06	ST5
11	8782570	chr11:8782570:I	2.109e-06	ST5
2	209006520	chr2:209006520:I	2.237e-06	LOC100507443
6	116426165	rs478187	3.002e-06	NT5DC1
2	208995109	rs2854719	3.226e-06	LOC100507443
2	209003563	rs13389772	3.227e-06	LOC100507443
11	5474838	rs2030094	3.929e-06	OR51I2
11	5473821	chr11:5473821:I	4.204e-06	missing
9	82666349	rs10867457	4.517e-06	missing
12	72260076	rs12310865	4.592e-06	TBC1D15
11	5472735	rs7120894	4.752e-06	missing
10	125727175	rs7074389	4.802e-06	missing
12	72259707	rs11834054	4.853e-06	TBC1D15
10	118678712	rs4752018	4.876e-06	KIAA1598
10	125729458	rs9422292	4.953e-06	missing
21	31466422	rs2832596	5.273e-06	missing
11	5472371	rs10742679	5.748e-06	missing
11	5473060	rs7942704	5.748e-06	missing

2	11691224	rs974163	5.779e-06	GREB1
11	5471746	rs7948471	5.83e-06	missing
11	5471832	rs7938426	6.032e-06	missing
10	125734851	rs4929811	6.064e-06	missing
2	208997125	chr2:208997125:I	6.409e-06	LOC100507443
11	5472768	rs6578646	7.085e-06	missing
11	5472767	rs6578645	7.237e-06	missing
2	208998341	rs75530197	7.613e-06	LOC100507443
10	125733523	rs7101053	7.924e-06	missing
2	209001699	rs2305428	8.478e-06	LOC100507443,LOC100533727
2	208998441	rs7425722	8.576e-06	LOC100507443
2	209002036	rs971493	8.892e-06	LOC100507443,LOC100533727
7	131547960	rs17133213	9.544e-06	missing

Manhattan Plot:

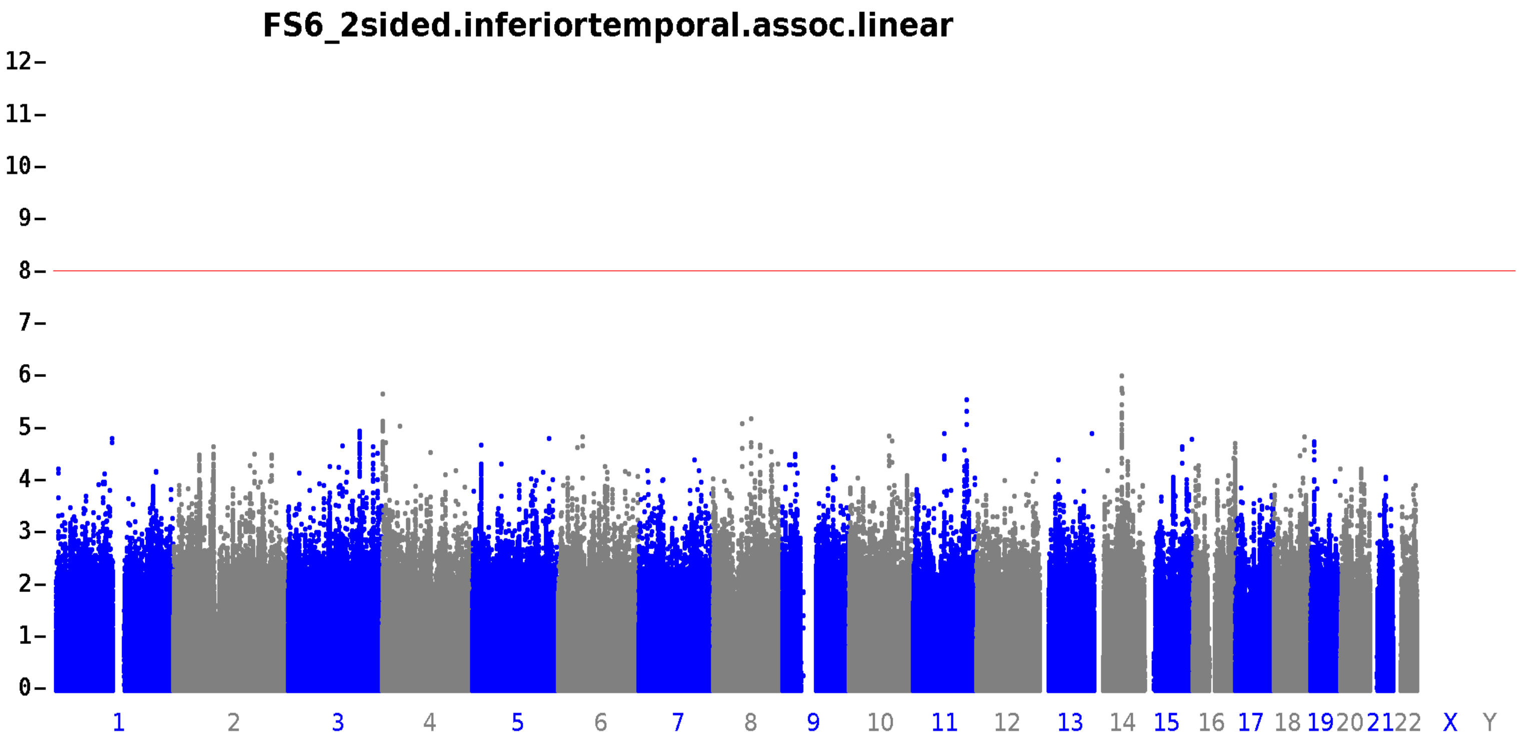

SNP mapped to gene:

CHR	BP	SNP	P	GENE
14	59491396	rs6573235	9.038e-07	missing
14	59491074	rs7145577	1.549e-06	missing
14	59492768	rs10135889	1.622e-06	missing
14	59504619	chr14:59504619:D	1.756e-06	missing
14	59815040	rs755011	1.991e-06	DAAM1
4	1749160	rs11731421	2.048e-06	missing
11	113345669	rs79092434	2.623e-06	DRD2
14	59451196	rs17255003	3.251e-06	missing
11	113338946	rs7102650	4.287e-06	DRD2
14	59468636	rs28661690	4.675e-06	missing
14	59470410	rs11623913	4.734e-06	missing
14	59470509	rs11623852	4.734e-06	missing
14	59460745	rs72724609	4.913e-06	missing
14	59471071	rs68136176	5.156e-06	missing
14	59452572	rs11628130	5.248e-06	missing
14	59494042	rs28524490	5.296e-06	missing
14	59463234	rs10047903	5.85e-06	missing
8	80002532	rs74940243	6.149e-06	missing
4	1743754	rs11248074	6.708e-06	TACC3
4	1742779	rs1530588	6.708e-06	TACC3
4	1744545	chr4:1744545:D	7.313e-06	TACC3
8	60580017	rs28709348	7.581e-06	missing
8	60580078	chr8:60580078:I	7.581e-06	missing
14	59469784	rs17833476	7.728e-06	missing

4	1746229	rs2290011	7.759e-06	TACC3
4	1746125	chr4:1746125:I	7.759e-06	TACC3
4	1744939	rs11736125	7.759e-06	TACC3
11	113337446	rs77655590	7.815e-06	DRD2
4	1737502	rs11248073	8.079e-06	TACC3
4	37065987	rs12507593	8.324e-06	missing
4	1737108	rs3752747	8.525e-06	TACC3
14	59450351	rs10129876	9.759e-06	missing
4	1715349	rs2854920	9.8e-06	missing
14	59449492	rs72722695	9.955e-06	missing

Manhattan Plot:

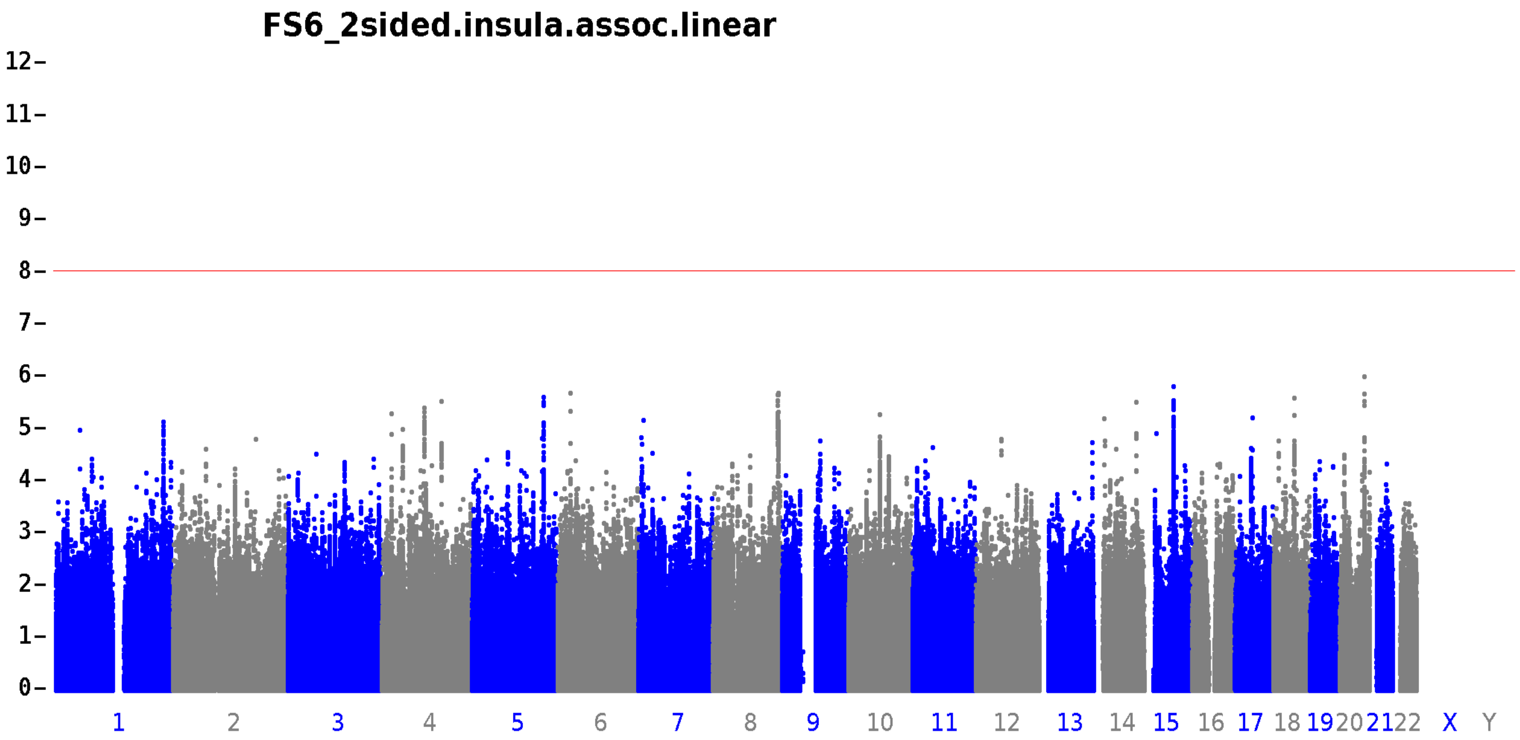

SNP mapped to gene:

CHR	BP	SNP	P	GENE
20	51278831	rs715433	9.501e-07	missing
15	62373113	rs4143842	1.465e-06	missing
6	27595044	rs6936999	1.944e-06	missing
8	137785396	chr8:137785396:D	1.985e-06	missing
20	51278108	rs6068294	2.059e-06	missing
8	137757449	rs7816164	2.111e-06	missing
8	137754996	chr8:137754996:D	2.111e-06	missing
8	137754467	rs3850495	2.111e-06	missing
8	137757301	rs7815994	2.111e-06	missing
8	137753761	rs7826349	2.111e-06	missing
8	137758854	rs78972200	2.111e-06	missing
8	137752666	chr8:137752666:I	2.111e-06	missing
8	137752084	rs112447175	2.111e-06	missing
8	137745755	rs78496734	2.111e-06	missing
8	137752221	rs56056072	2.111e-06	missing
8	137745746	rs80281427	2.111e-06	missing
5	151141895	rs2964585	2.328e-06	missing
5	151140844	rs1549920	2.334e-06	missing
18	44045347	rs112134119	2.414e-06	missing
18	44045966	rs76992290	2.414e-06	missing
8	137745657	rs17587409	2.672e-06	missing
15	62379449	rs34096515	2.76e-06	missing
15	62372827	rs17205407	2.773e-06	missing
15	62374487	rs1436964	2.773e-06	missing

15	62373940	rs10047986	2.773e-06	missing
15	62374593	rs1436963	2.773e-06	missing
15	62373459	rs7162536	2.773e-06	missing
15	62374915	rs4775457	2.773e-06	NPM1P47
15	62374942	rs12438803	2.773e-06	NPM1P47
15	62375036	rs4775458	2.773e-06	NPM1P47
20	51282552	rs6063858	2.824e-06	missing
4	126696422	rs4488951	2.854e-06	missing
8	137715389	rs112808998	2.87e-06	missing
15	62372592	rs12913951	2.898e-06	missing
5	151130901	rs2915822	2.962e-06	ATOX1
14	90052945	rs243176	2.976e-06	FOXN3
15	62375389	rs17271403	3.172e-06	NPM1P47
15	62381065	rs17271438	3.364e-06	missing
8	137721436	rs116197725	3.374e-06	missing
20	51286077	rs6068296	3.387e-06	missing
5	151126324	rs2016409	3.405e-06	ATOX1
4	89796711	rs150775800	3.745e-06	FAM13A
15	62377805	rs8026008	3.788e-06	missing
15	62377820	rs8025877	3.788e-06	missing
15	62378136	rs731820	3.824e-06	missing
15	62378608	rs893158	3.824e-06	missing
15	62189432	rs12901247	4.124e-06	VPS13C
6	27623511	rs9366694	4.378e-06	missing
4	89791075	rs17815912	4.48e-06	FAM13A
8	137798925	rs79630743	4.535e-06	missing
8	137698084	rs76657704	4.631e-06	missing
8	137699837	rs17646719	4.743e-06	missing
8	137687955	rs17646599	4.854e-06	missing
4	19988469	rs4411990	4.868e-06	missing
10	66842456	rs10996188	4.962e-06	missing
8	137700990	rs56937088	5.127e-06	missing
8	137701072	rs75941775	5.127e-06	missing
8	137700774	rs78788770	5.127e-06	missing
8	137701373	rs113623821	5.133e-06	missing
8	137704159	rs74781139	5.133e-06	missing
18	44043455	rs113826818	5.15e-06	missing
4	89855138	rs72665835	5.591e-06	FAM13A
4	89795863	rs142866806	5.641e-06	FAM13A
4	89864998	rs59489826	5.646e-06	FAM13A
15	62380390	rs12438690	5.647e-06	missing
17	37166993	rs113511226	5.832e-06	missing
8	137682669	rs79168090	5.845e-06	missing
15	62370484	rs11635977	5.851e-06	missing
8	137744118	chr8:137744118:I	5.929e-06	missing
8	137743491	rs113414227	5.929e-06	missing
8	137743543	rs10505663	5.929e-06	missing

14	23441543	chr14:23441543:I	6.124e-06	AJUBA
8	137710873	rs55665977	6.151e-06	missing
7	11330027	rs17164270	6.445e-06	missing
15	62365390	rs12900703	6.6e-06	missing
8	137734369	rs76261634	6.635e-06	missing
8	137706843	rs112911717	6.641e-06	missing
4	89792885	rs11732471	6.715e-06	FAM13A
15	62380595	rs12439356	6.769e-06	missing
8	137758044	rs77721199	6.83e-06	missing
15	62359350	rs8039651	6.897e-06	C2CD4A
8	137794069	rs75484363	6.926e-06	missing
1	227819369	rs12080321	6.951e-06	ZNF678
8	137742641	rs78418909	7.07e-06	missing
8	137749921	rs7822714	7.206e-06	missing
8	137750168	rs7824092	7.206e-06	missing
8	137751689	rs75320527	7.206e-06	missing
8	137749983	rs7822847	7.206e-06	missing
8	137753352	rs56087125	7.206e-06	missing
8	137749207	rs112687646	7.206e-06	missing
8	137753398	rs55997640	7.206e-06	missing
8	137749137	rs113910390	7.206e-06	missing
8	137753448	rs7822396	7.206e-06	missing
8	137748918	rs80109840	7.206e-06	missing
8	137754601	rs3850496	7.206e-06	missing
8	137747288	rs75317421	7.206e-06	missing
8	137756206	rs79275478	7.206e-06	missing
8	137745571	rs17587374	7.206e-06	missing
8	137757057	rs7815557	7.206e-06	missing
8	137745536	rs17647650	7.206e-06	missing
8	137757796	rs77852240	7.206e-06	missing
8	137745152	rs17647614	7.206e-06	missing
8	137758887	rs78297157	7.206e-06	missing
8	137743439	rs77965990	7.206e-06	missing
8	137762202	rs111533073	7.206e-06	missing
8	137743331	rs76338317	7.206e-06	missing
8	137762402	rs75085741	7.206e-06	missing
8	137743048	rs79705404	7.206e-06	missing
8	137766592	rs17594095	7.206e-06	missing
8	137742548	rs75448392	7.206e-06	missing
8	137767546	rs7821442	7.206e-06	missing
8	137741563	rs56358005	7.206e-06	missing
8	137767616	rs7839295	7.206e-06	missing
8	137735613	rs17647473	7.206e-06	missing
8	137768473	rs7813155	7.241e-06	missing
5	151147198	rs2964578	7.265e-06	missing
8	137739989	rs56034455	7.318e-06	missing
8	137729204	rs17647250	7.318e-06	missing

8	137727453	rs79303926	7.318e-06	missing
8	137723617	rs76694267	7.318e-06	missing
8	137723215	rs17647089	7.318e-06	missing
8	137724128	rs55915103	7.318e-06	missing
8	137722871	rs17586653	7.318e-06	missing
8	137725175	rs77810730	7.318e-06	missing
8	137722826	rs79544863	7.318e-06	missing
8	137725629	rs75583953	7.318e-06	missing
8	137722425	rs78439910	7.318e-06	missing
8	137722423	rs77862711	7.318e-06	missing
8	137725764	rs113688638	7.318e-06	missing
8	137722364	rs74420931	7.318e-06	missing
8	137726332	rs79971898	7.318e-06	missing
8	137722357	rs75759378	7.318e-06	missing
8	137730991	rs79945587	7.318e-06	missing
8	137721989	rs6989696	7.318e-06	missing
8	137731182	rs75123023	7.318e-06	missing
8	137721743	rs6989264	7.318e-06	missing
8	137732468	rs112982444	7.318e-06	missing
8	137720894	rs75855177	7.318e-06	missing
8	137732719	rs17587015	7.318e-06	missing
8	137719203	rs80308726	7.318e-06	missing
8	137733343	rs76699138	7.318e-06	missing
8	137718995	chr8:137718995:D	7.318e-06	missing
8	137733497	rs80114875	7.318e-06	missing
8	137718923	rs113537054	7.318e-06	missing
8	137734805	rs17587101	7.318e-06	missing
8	137735286	rs17647461	7.318e-06	missing
8	137735860	rs77229948	7.318e-06	missing
8	137734842	rs56262276	7.318e-06	missing
8	137737265	rs114815340	7.318e-06	missing
8	137717920	rs75895679	7.318e-06	missing
8	137739697	rs55658615	7.318e-06	missing
8	137717440	rs17646994	7.318e-06	missing
8	137740479	rs79946217	7.318e-06	missing
8	137717340	rs17646958	7.318e-06	missing
8	137718298	rs76613468	7.324e-06	missing
8	137715861	rs77527004	7.324e-06	missing
8	137715776	rs75157763	7.324e-06	missing
8	137715541	chr8:137715541:I	7.324e-06	missing
8	137718224	rs79333688	7.356e-06	missing
8	137762987	chr8:137762987:D	7.38e-06	missing
8	137725713	rs75289790	7.407e-06	missing
8	137737835	rs55881368	7.414e-06	missing
4	89814047	chr4:89814047:D	7.565e-06	FAM13A
8	137729314	rs17647274	7.62e-06	missing
15	62365107	rs4564516	7.728e-06	missing

8	137778780	chr8:137778780:D	7.878e-06	missing
8	137773900	rs111402489	7.878e-06	missing
15	62379971	rs7177711	7.903e-06	missing
8	137732661	rs75184246	7.966e-06	missing
8	137721889	rs7002910	8.214e-06	missing
1	227819490	rs12070002	8.334e-06	ZNF678
5	151149001	rs62377931	8.344e-06	missing
5	151115517	rs2915817	8.365e-06	missing
5	151115883	rs2915815	8.365e-06	missing
4	89836536	rs56837788	8.377e-06	FAM13A
4	89833082	rs72665823	8.377e-06	FAM13A
15	62365732	chr15:62365732:D	8.559e-06	missing
4	89844697	rs144165668	8.567e-06	FAM13A
15	62353458	rs8029942	8.832e-06	LOC101928907
8	137723820	rs17647106	8.984e-06	missing
8	137769867	rs75245195	9.095e-06	missing
8	137768063	rs17594232	9.095e-06	missing
8	137775833	rs111823623	9.195e-06	missing
8	137787861	rs79965553	9.195e-06	missing
8	137782504	rs112827815	9.195e-06	missing
8	137788174	rs77266899	9.195e-06	missing
8	137782268	rs112968651	9.195e-06	missing
8	137789180	rs75472914	9.195e-06	missing
8	137782116	rs112776225	9.195e-06	missing
8	137792032	rs55955815	9.195e-06	missing
8	137781331	rs56314248	9.195e-06	missing
8	137792858	rs79678119	9.195e-06	missing
8	137779834	rs75633892	9.195e-06	missing
8	137794790	rs78538375	9.195e-06	missing
8	137775306	rs147984323	9.195e-06	missing
8	137799345	rs74363428	9.413e-06	missing
4	89861309	rs2904256	9.575e-06	FAM13A
8	137803849	rs2875701	9.6e-06	missing
8	137804140	rs3850502	9.6e-06	missing
4	44535145	rs2118937	9.741e-06	missing
1	227812941	rs12079123	9.897e-06	ZNF678

Manhattan Plot:

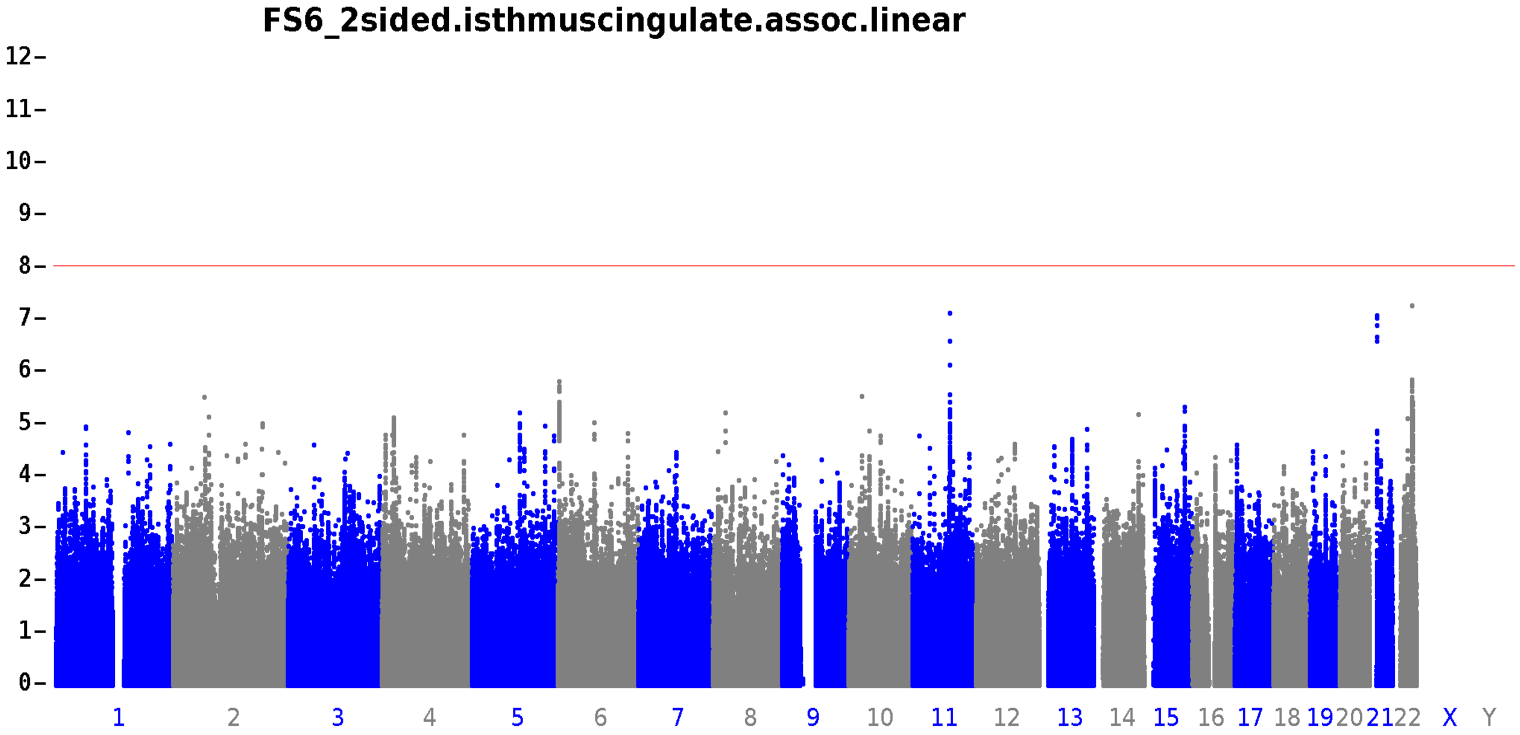

SNP mapped to gene:

CHR	BP	SNP	P	GENE
22	42398849	rs133310	5.172e-08	WBP2NL
11	79631597	rs596896	7.192e-08	missing
21	16171020	rs9982123	7.976e-08	missing
21	16168717	rs1534839	8.971e-08	missing
21	16165965	rs1997579	1.252e-07	missing
21	16162541	rs9975483	2.025e-07	missing
21	16164920	rs8132693	2.456e-07	missing
11	79631868	rs624164	2.511e-07	missing
11	79631786	rs624285	6.989e-07	missing
22	42416987	rs133339	1.378e-06	WBP2NL
22	42416056	rs133335	1.412e-06	WBP2NL
6	3769745	rs11242880	1.486e-06	missing
22	42540551	rs2743461	1.542e-06	CYP2D7P1
22	42407689	rs133327	1.736e-06	WBP2NL
6	3764880	rs73356955	1.814e-06	missing
22	42405322	rs133321	1.83e-06	WBP2NL
6	3762433	rs12215659	1.97e-06	missing
6	3762342	rs74810911	1.97e-06	missing
6	3763025	rs7756322	2.059e-06	missing
22	42385967	rs6519301	2.198e-06	SEPT3
6	3765691	rs10458078	2.214e-06	missing
22	42390093	rs133301	2.279e-06	SEPT3
6	3762685	rs7738442	2.303e-06	missing
11	79688804	rs642770	2.604e-06	missing

10	27707512	rs12783768	2.851e-06	missing
22	42405922	rs133323	2.871e-06	WBP2NL
22	42401749	rs133313	2.878e-06	WBP2NL
22	42401841	rs133314	2.878e-06	WBP2NL
2	65939468	rs113269336	2.892e-06	LOC100996770
22	42380001	rs5751195	3.355e-06	SEPT3
22	42397042	rs5758537	3.493e-06	WBP2NL,SLC25A5P1
22	42396568	rs133307	3.493e-06	WBP2NL
22	42396150	rs133306	3.493e-06	WBP2NL
22	42395960	rs133305	3.493e-06	WBP2NL
22	42609148	rs5758651	3.599e-06	TCF20
11	79622779	rs681822	3.63e-06	missing
6	3770293	rs10458079	3.643e-06	missing
22	42488163	rs5758576	3.679e-06	NDUFA6-AS1
22	42491479	rs5758579	3.713e-06	NDUFA6-AS1
22	42399396	rs129856	3.745e-06	WBP2NL
22	42395242	rs133303	3.818e-06	WBP2NL
6	3768270	rs78810036	4.117e-06	missing
22	42645202	rs5758682	4.236e-06	TCF20
22	42528976	rs28360521	4.244e-06	missing
22	42406371	rs133324	4.396e-06	WBP2NL
22	42409456	rs133330	4.396e-06	WBP2NL
15	85702753	rs6496767	4.475e-06	missing
6	3762208	rs78754035	4.548e-06	missing
22	42399325	rs129855	4.576e-06	WBP2NL
22	42399686	rs129857	4.576e-06	WBP2NL
11	79685121	rs625168	5.018e-06	missing
6	3763181	rs10458161	5.037e-06	missing
22	42612408	rs5758652	5.237e-06	TCF20
22	42375761	rs5758527	5.255e-06	SEPT3
22	42365073	rs10154700	5.32e-06	missing
22	42538071	rs2267444	5.369e-06	CYP2D7P1
15	85705121	rs6496770	5.488e-06	missing
22	42486056	rs5751211	5.532e-06	NDUFA6
22	42578596	rs9607885	5.659e-06	TCF20
6	3772896	rs12206306	5.677e-06	missing
22	42534956	rs2743453	5.683e-06	missing
22	42614823	rs34979382	5.753e-06	TCF20
8	26869785	rs9693865	5.817e-06	LOC100132229
11	79691401	rs600385	5.82e-06	missing
5	101385881	rs12054761	5.895e-06	missing
22	42619308	rs1033460	5.947e-06	TCF20
6	3773654	rs56122805	5.967e-06	missing
6	3773398	rs56128599	5.967e-06	missing
22	42493875	rs5758580	6.235e-06	NDUFA6-AS1
22	42540863	rs3021079	6.26e-06	missing
22	42663871	rs5751258	6.281e-06	TCF20

14	96029868	rs35436559	6.285e-06	missing
11	79688850	rs642405	6.349e-06	missing
22	42618669	rs5758657	6.539e-06	TCF20
22	42406885	rs133326	6.554e-06	WBP2NL
22	42614825	chr22:42614825:I	6.6e-06	TCF20
22	42403853	rs133320	6.718e-06	WBP2NL
22	42400416	rs6002571	6.785e-06	WBP2NL
22	42534601	rs2743450	6.813e-06	missing
6	3759207	rs4431478	6.986e-06	missing
11	79689054	rs641527	6.994e-06	missing
22	42667962	rs66977013	7.047e-06	LOC388906
2	76251131	rs72907431	7.105e-06	missing
22	42664972	rs5758689	7.19e-06	missing
4	25033280	rs9998234	7.281e-06	missing
22	42541328	rs4467371	7.537e-06	missing
22	32178117	chr22:32178117:I	7.543e-06	DEPDC5
4	25035399	chr4:25035399:I	7.601e-06	missing
22	42382797	rs133293	7.682e-06	SEPT3
22	42668295	rs5758690	7.752e-06	LOC388906
6	3773768	rs73356977	7.87e-06	missing
22	42633204	rs5758667	7.898e-06	TCF20
22	42536313	rs2743456	8.139e-06	CYP2D7P1
22	42492985	rs57117731	8.143e-06	NDUFA6-AS1
22	42538084	rs2267446	8.379e-06	CYP2D7P1
4	25033008	rs736831	8.758e-06	missing
22	42642576	rs58654759	8.782e-06	TCF20
22	42670111	rs1001587	8.829e-06	LOC388906
22	42670293	rs1001586	8.829e-06	LOC388906
22	42671066	rs739147	8.829e-06	missing
22	42668504	rs5758691	8.829e-06	LOC388906
22	42667594	rs1107554	8.995e-06	LOC388906
6	3769191	rs12210209	9.081e-06	missing
6	77789421	chr6:77789421:D	9.104e-06	missing
22	42667473	rs1107553	9.172e-06	LOC388906
5	101104880	rs6877543	9.205e-06	missing
22	42567451	rs9607882	9.286e-06	TCF20
2	188871632	chr2:188871632:D	9.317e-06	missing
11	79688912	rs645370	9.355e-06	missing
5	101348691	rs72774363	9.376e-06	missing
22	42382748	rs133292	9.491e-06	SEPT3
22	42561365	rs9611746	9.503e-06	TCF20
22	42625509	rs17002902	9.514e-06	TCF20
22	42628088	rs5758662	9.514e-06	TCF20
22	42623258	rs55867855	9.514e-06	TCF20
22	42655377	rs5758686	9.518e-06	TCF20
22	42662501	rs5758688	9.583e-06	TCF20
22	42541285	rs2856957	9.679e-06	missing

22	42541394	rs5751226	9.679e-06	missing
22	42542153	rs5751227	9.679e-06	missing
22	42684818	rs5758698	9.692e-06	missing
6	3767777	rs7741887	9.72e-06	missing
4	25034175	rs10001303	9.939e-06	missing

Manhattan Plot:

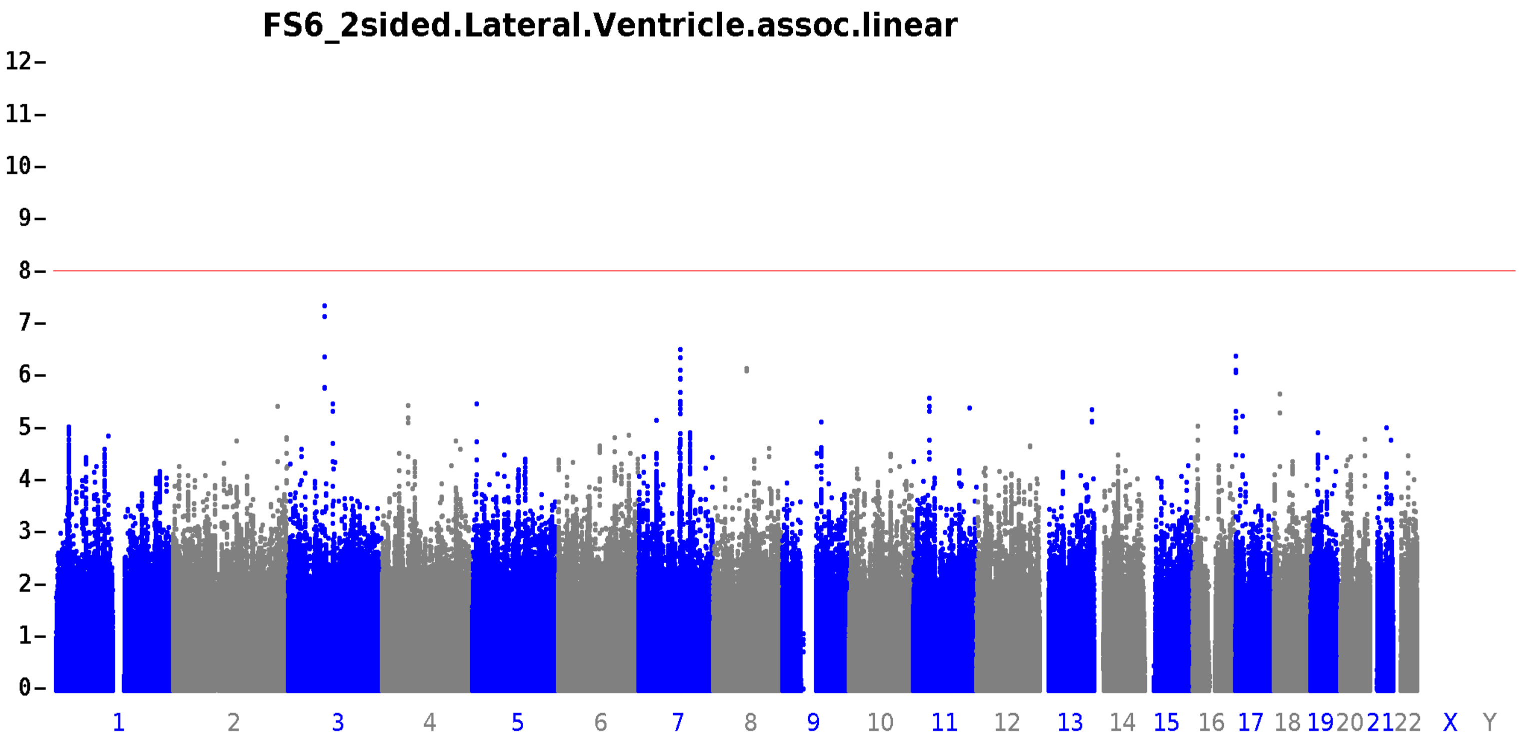

SNP mapped to gene:

CHR	BP	SNP	P	GENE
3	77856623	rs3852018	4.217e-08	missing
3	77842138	rs9824656	6.592e-08	missing
7	88546344	rs12666963	2.902e-07	ZNF804B
17	289269	rs9889815	3.792e-07	missing
3	77896273	rs9812488	4e-07	missing
7	88541704	rs12671549	4.117e-07	ZNF804B
8	70665563	rs10101039	6.657e-07	SLCO5A1
7	88519331	rs1609374	6.963e-07	ZNF804B
8	70661555	rs10112770	7.048e-07	SLCO5A1
17	288148	rs8076098	7.081e-07	missing
8	70651455	rs16919172	7.214e-07	SLCO5A1
8	70654071	rs10216572	7.214e-07	SLCO5A1
17	289708	rs9908779	7.452e-07	missing
17	288943	rs9894984	7.823e-07	missing
7	88513041	rs2214632	1.022e-06	ZNF804B
7	88518174	rs11981162	1.048e-06	ZNF804B
7	88518146	rs11974437	1.048e-06	ZNF804B
3	77873563	rs11923610	1.505e-06	missing
3	77888401	chr3:77888401:I	1.572e-06	missing
7	88474666	rs17164709	1.882e-06	ZNF804B
18	12969888	chr18:12969888:I	2.035e-06	SEH1L
11	34413865	rs61880787	2.438e-06	missing
7	88522762	rs62464064	2.779e-06	ZNF804B
3	95448245	chr3:95448245:I	3.187e-06	missing

5	9130780	rs1479647	3.195e-06	SEMA5A
7	88527360	rs35688571	3.26e-06	ZNF804B
7	88504112	rs34054603	3.281e-06	ZNF804B
4	54628995	rs62297703	3.422e-06	missing
2	220898718	rs77175201	3.453e-06	missing
11	34396539	rs7927163	3.495e-06	missing
11	120426508	rs2248558	3.779e-06	GRIK4
7	88479121	rs7797423	3.919e-06	ZNF804B
13	110918660	rs11619113	4.057e-06	COL4A1
13	110918874	rs11616893	4.057e-06	COL4A1
3	95450052	chr3:95450052:D	4.342e-06	missing
17	285990	rs9893525	4.363e-06	missing
11	34380706	rs58512167	4.393e-06	missing
18	12970526	rs57692810	4.648e-06	SEH1L
7	88513561	rs6948962	4.94e-06	ZNF804B
17	15322064	rs8078304	5.517e-06	missing
17	283566	rs7502706	5.772e-06	missing
4	54629549	rs62297705	5.837e-06	missing
7	37986332	rs2167267	6.48e-06	EPDR1
13	110920852	rs12873154	6.691e-06	COL4A1
13	110923799	rs11619038	6.874e-06	COL4A1
9	82945953	rs72742702	6.892e-06	missing
4	54631772	rs4457010	7.152e-06	missing
16	11250413	rs7189563	8.292e-06	CLEC16A
1	27473349	rs6691248	8.706e-06	SLC9A1
1	27455505	rs752454	8.909e-06	SLC9A1
21	34392683	rs35474228	8.989e-06	missing
17	278302	rs11652688	9.018e-06	missing
1	27474064	rs12120926	9.251e-06	SLC9A1

Manhattan Plot:

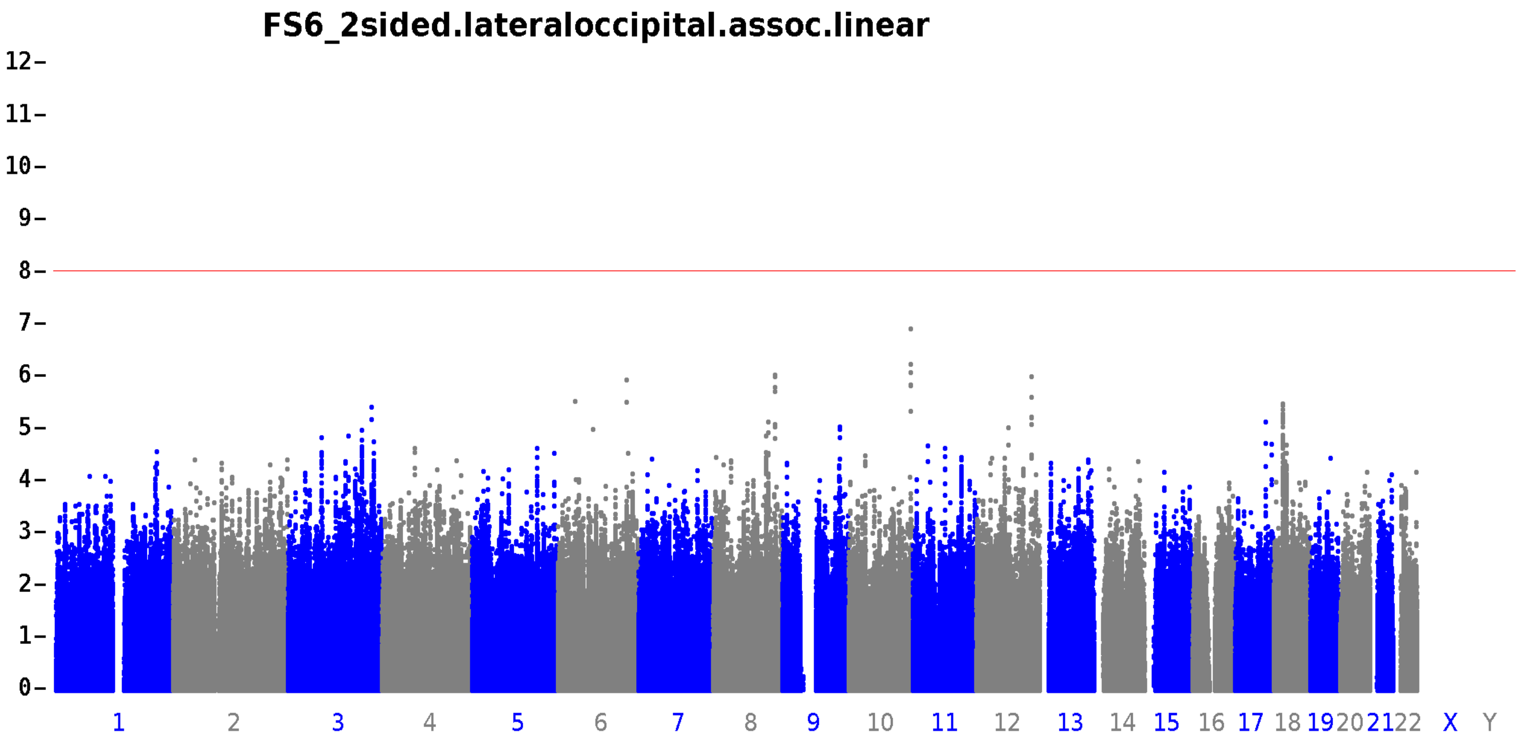

SNP mapped to gene:

CHR	BP	SNP	P	GENE
10	131246957	rs1973698	1.161e-07	missing
10	131253796	rs1771449	5.521e-07	missing
10	131256585	rs11016798	8e-07	missing
8	130483338	rs12543365	8.831e-07	missing
8	130500331	rs1835844	9.343e-07	missing
12	116263755	rs7960215	9.437e-07	missing
6	145614252	rs476358	1.113e-06	missing
10	131247389	rs1554225	1.357e-06	missing
10	131247521	rs1556656	1.407e-06	missing
8	130491752	rs891835	1.496e-06	missing
8	130491452	rs891834	1.831e-06	missing
12	116258388	rs111607789	2.389e-06	missing
6	36695223	rs12201069	2.844e-06	RAB44
6	145634441	rs79556505	2.954e-06	missing
18	19636742	rs7234041	3.187e-06	missing
18	19636739	rs7235214	3.375e-06	missing
3	176635691	rs76034233	3.696e-06	missing
18	19613151	rs35687829	3.989e-06	missing
10	131250070	rs11016797	4.405e-06	missing
18	19630281	rs12961736	4.793e-06	missing
18	19638310	rs8086768	5.471e-06	missing
12	116260661	rs17776952	5.556e-06	missing
12	116257664	rs11611588	5.76e-06	missing
18	19632380	rs12958584	6.19e-06	missing

3	176650567	rs77938864	6.298e-06	missing
8	116952845	rs7827717	6.923e-06	missing
17	64730629	rs9899787	7.104e-06	PRKCA
18	19616025	rs9952434	7.294e-06	missing
8	130480641	rs10956481	7.702e-06	missing
12	116250734	rs2080652	7.862e-06	missing
18	19637466	chr18:19637466:D	8.671e-06	missing
8	130473200	rs977308	8.684e-06	missing
18	19613152	chr18:19613152:I	8.74e-06	missing
9	122500129	rs17478568	8.782e-06	missing
18	19632479	rs12959282	8.828e-06	missing
12	68440296	chr12:68440296:D	8.903e-06	missing
9	122527946	rs4837668	9.585e-06	missing
6	74432636	rs9442951	9.659e-06	CD109
3	157179834	rs4680369	9.881e-06	VEPH1

Manhattan Plot:

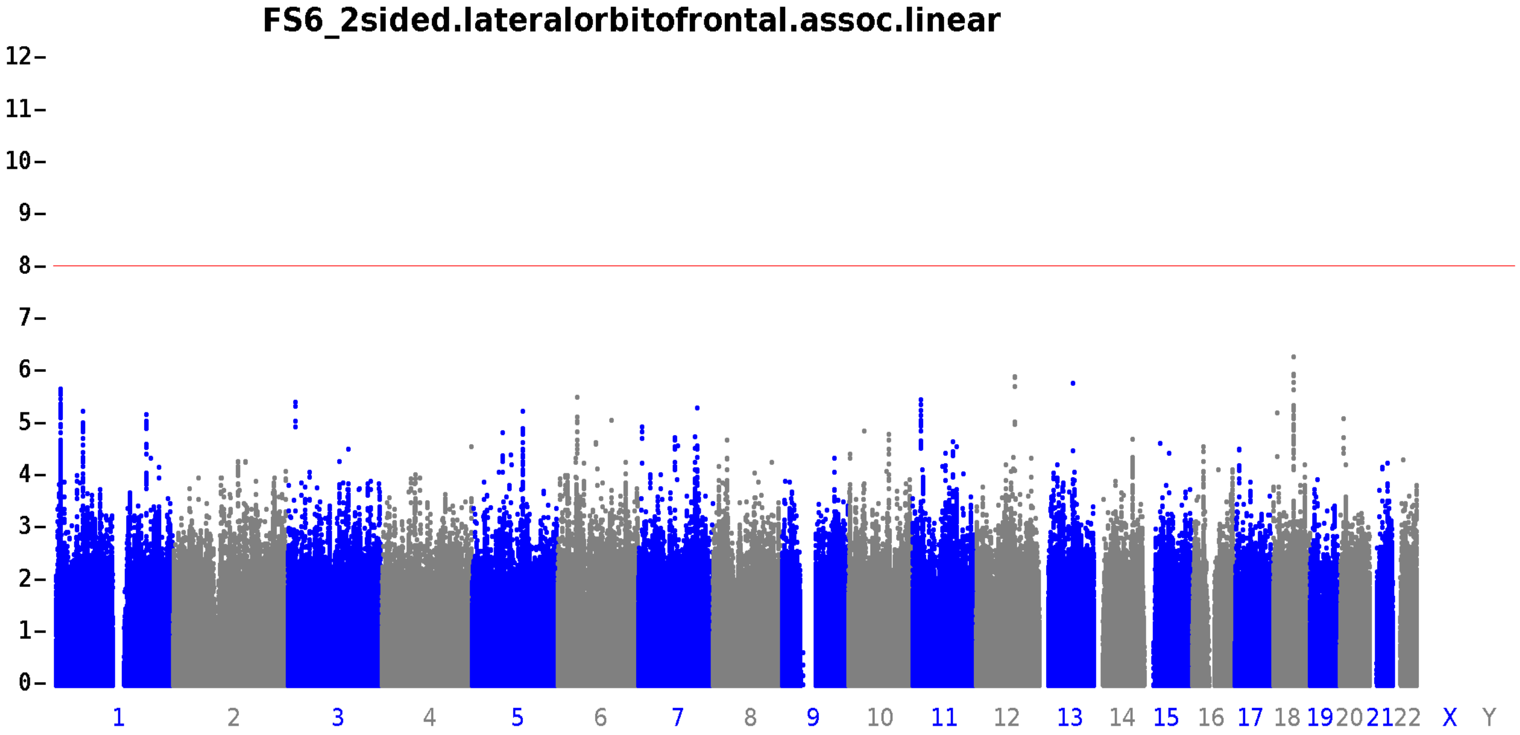

SNP mapped to gene:

CHR	BP	SNP	P	GENE
18	43013572	rs2362840	4.834e-07	SLC14A2,LOC101927980
18	43014121	rs1345491	4.834e-07	SLC14A2,LOC101927980
18	43013567	rs4358031	4.834e-07	SLC14A2,LOC101927980
18	43013294	rs57793355	4.834e-07	SLC14A2,LOC101927980
18	43015532	rs2287434	1.068e-06	SLC14A2,LOC101927980,
18	43013668	rs2216416	1.143e-06	SLC14A2,LOC101927980
12	81595700	rs11114787	1.191e-06	ACSS3
12	81599705	rs9888380	1.232e-06	ACSS3
18	43014434	chr18:43014434:D	1.523e-06	SLC14A2,LOC101927980
13	71646254	rs1467627	1.573e-06	missing
12	81598584	rs7136590	1.807e-06	ACSS3
1	10435816	rs61778406	2.026e-06	KIF1B
18	43024398	rs2362841	2.144e-06	SLC14A2,LOC101927980,
1	10443590	chr1:10443590:I	2.243e-06	missing
1	10443867	rs11585794	2.663e-06	missing
6	41286008	rs67592608	2.899e-06	missing
6	41286012	rs67128026	2.899e-06	missing
6	41285965	rs67124164	2.899e-06	missing
1	10447622	rs11587854	3.165e-06	missing
11	17684732	rs6486386	3.23e-06	missing
3	16388390	rs7622858	3.635e-06	RFTN1
1	10457540	rs12409754	3.926e-06	missing
1	10454714	rs12141201	3.936e-06	missing
11	17683525	rs4757558	4.012e-06	missing

11	17683264	rs4757557	4.012e-06	missing
18	43014134	rs1345492	4.209e-06	SLC14A2,LOC101927980
1	10459779	rs3737155	4.306e-06	PGD
3	16391851	chr3:16391851:D	4.356e-06	RFTN1
7	124783849	rs2293346	4.732e-06	missing
18	43011077	rs1465371	4.809e-06	SLC14A2,LOC101927980
1	10453374	rs7530167	4.846e-06	missing
1	10438892	rs1002076	4.881e-06	KIF1B
1	10448999	rs11587495	5.165e-06	missing
11	17688529	chr11:17688529:I	5.228e-06	missing
18	43013157	rs3909261	5.235e-06	SLC14A2,LOC101927980
11	17695874	rs3858491	5.268e-06	missing
11	17690789	rs4757561	5.282e-06	missing
5	107925765	rs9326734	5.399e-06	missing
1	58362123	rs1202835	5.407e-06	DAB1
1	10461107	rs11121560	5.547e-06	PGD
18	8840838	rs4798694	5.912e-06	missing
1	192774477	rs77609503	6.218e-06	missing
18	43021140	rs1421194	6.393e-06	SLC14A2,LOC101927980,
11	17697503	rs4757562	6.457e-06	missing
1	10442926	rs60743860	6.469e-06	missing
6	41279788	rs13211886	7.004e-06	missing
6	41281870	chr6:41281870:D	7.004e-06	missing
1	10475120	rs11576384	7.356e-06	PGD
20	6897593	rs4592924	7.393e-06	missing
18	43012312	rs11082446	7.448e-06	SLC14A2,LOC101927980
18	43012472	rs11873459	7.448e-06	SLC14A2,LOC101927980
18	43012435	rs11082448	7.448e-06	SLC14A2,LOC101927980
18	43012623	rs3850523	7.448e-06	SLC14A2,LOC101927980
18	43012431	rs11082447	7.448e-06	SLC14A2,LOC101927980
18	43013041	rs3844038	7.448e-06	SLC14A2,LOC101927980
18	43012358	rs11660576	7.448e-06	SLC14A2,LOC101927980
18	43012344	rs11664067	7.448e-06	SLC14A2,LOC101927980
20	6897700	rs4603854	7.556e-06	missing
11	17675080	rs7101815	8.165e-06	missing
6	114251867	chr6:114251867:D	8.214e-06	missing
11	17699704	rs7935216	8.264e-06	missing
3	16396272	rs34888646	8.383e-06	RFTN1
6	41283287	rs34830209	8.463e-06	missing
1	192770519	rs74687876	8.541e-06	missing
6	41283456	rs13205497	8.545e-06	missing
6	41283399	rs13205790	8.545e-06	missing
12	81604161	rs7968007	8.618e-06	ACSS3
11	17685462	rs4757559	8.736e-06	missing
6	41284030	rs56171453	8.754e-06	missing
6	41283983	rs36091686	8.754e-06	missing
6	41284152	rs34689624	8.754e-06	missing

6	41283931	rs17608590	8.754e-06	missing
6	41284150	rs35548358	8.869e-06	missing
1	58367209	rs1202839	9.065e-06	DAB1
11	17698692	rs4757563	9.084e-06	missing
1	192772273	rs77280497	9.442e-06	missing
18	43002176	rs9950296	9.676e-06	SLC14A2
1	10427459	rs34636442	9.767e-06	KIF1B
12	81604773	rs7954225	9.823e-06	ACSS3
18	43005963	chr18:43005963:D	9.93e-06	SLC14A2,LOC101927980

Manhattan Plot:

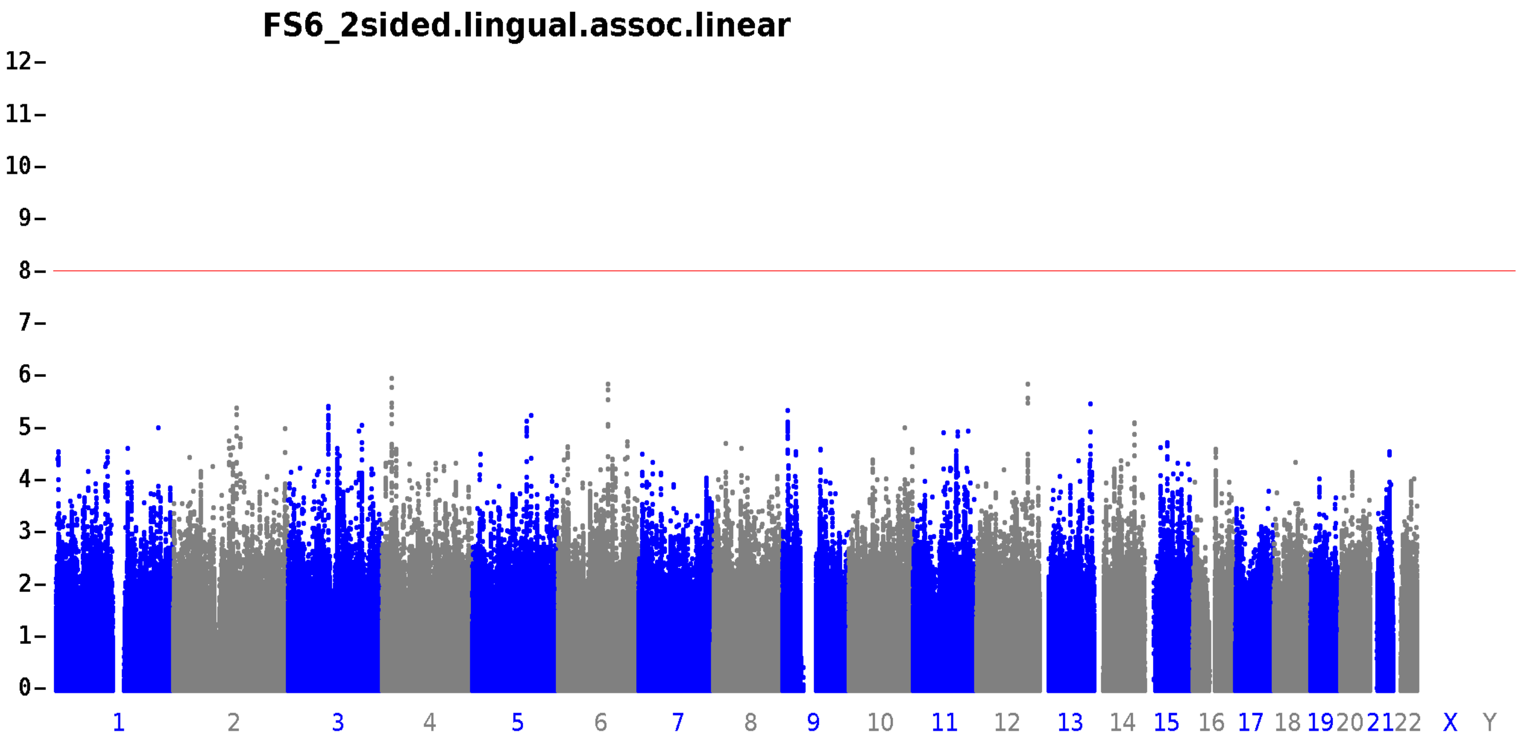

SNP mapped to gene:

CHR	BP	SNP	P	GENE
4	20945826	rs1347385	1.018e-06	KCNIP4
12	108474631	rs11608785	1.322e-06	missing
6	106800394	chr6:106800394:D	1.331e-06	missing
4	20956632	rs1865444	1.495e-06	KCNIP4
6	106799970	rs62420764	1.681e-06	missing
12	108403913	rs71454717	2.458e-06	missing
6	106797087	rs9399982	2.62e-06	missing
4	20954962	rs6448004	2.989e-06	KCNIP4
12	108397985	rs7295909	2.995e-06	missing
13	107091074	rs116831470	3.148e-06	RPL35P7
3	85716560	rs78532577	3.562e-06	CADM2
4	20946495	chr4:20946495:I	3.671e-06	KCNIP4
3	85716227	rs74400531	3.745e-06	CADM2
2	134261295	rs1432284	3.811e-06	NCKAP5
9	10859621	rs76258469	4.248e-06	missing
9	10859843	rs79644758	4.248e-06	missing
4	20938619	rs7690016	4.998e-06	KCNIP4
2	134261816	rs6736284	5.06e-06	NCKAP5
3	85700890	rs6785555	5.158e-06	CADM2
5	124688713	rs72782907	5.298e-06	missing
3	85711039	rs75027492	5.791e-06	CADM2
3	85685387	rs75699990	6.025e-06	CADM2
3	85720888	rs7644873	6.206e-06	CADM2
5	114913930	rs11740401	6.675e-06	missing

9	10872109	rs77487902	6.882e-06	missing
14	85577359	rs17120757	7.301e-06	missing
4	20953796	rs6448003	7.413e-06	KCNIP4
9	10871852	rs7048354	7.485e-06	missing
14	85576853	chr14:85576853:I	7.496e-06	missing
3	85687128	rs75739001	7.743e-06	CADM2
6	106791204	rs9386527	7.784e-06	missing
3	85714440	rs57885949	7.882e-06	CADM2
6	106801481	rs11758295	7.947e-06	missing
3	157186678	rs4679796	8.236e-06	VEPH1
6	106788486	rs9384603	8.278e-06	missing
3	85697381	rs77434537	8.656e-06	CADM2
9	10859828	rs76999355	8.846e-06	missing
2	134260359	rs59817257	8.948e-06	NCKAP5
5	114902614	rs256979	8.985e-06	missing
3	85696295	rs58879228	9.018e-06	CADM2
1	217388033	rs6694391	9.047e-06	missing
9	10860666	rs77806833	9.15e-06	missing
10	118041682	chr10:118041682:D	9.161e-06	missing
2	237252454	rs72978647	9.393e-06	IQCA1

Manhattan Plot:

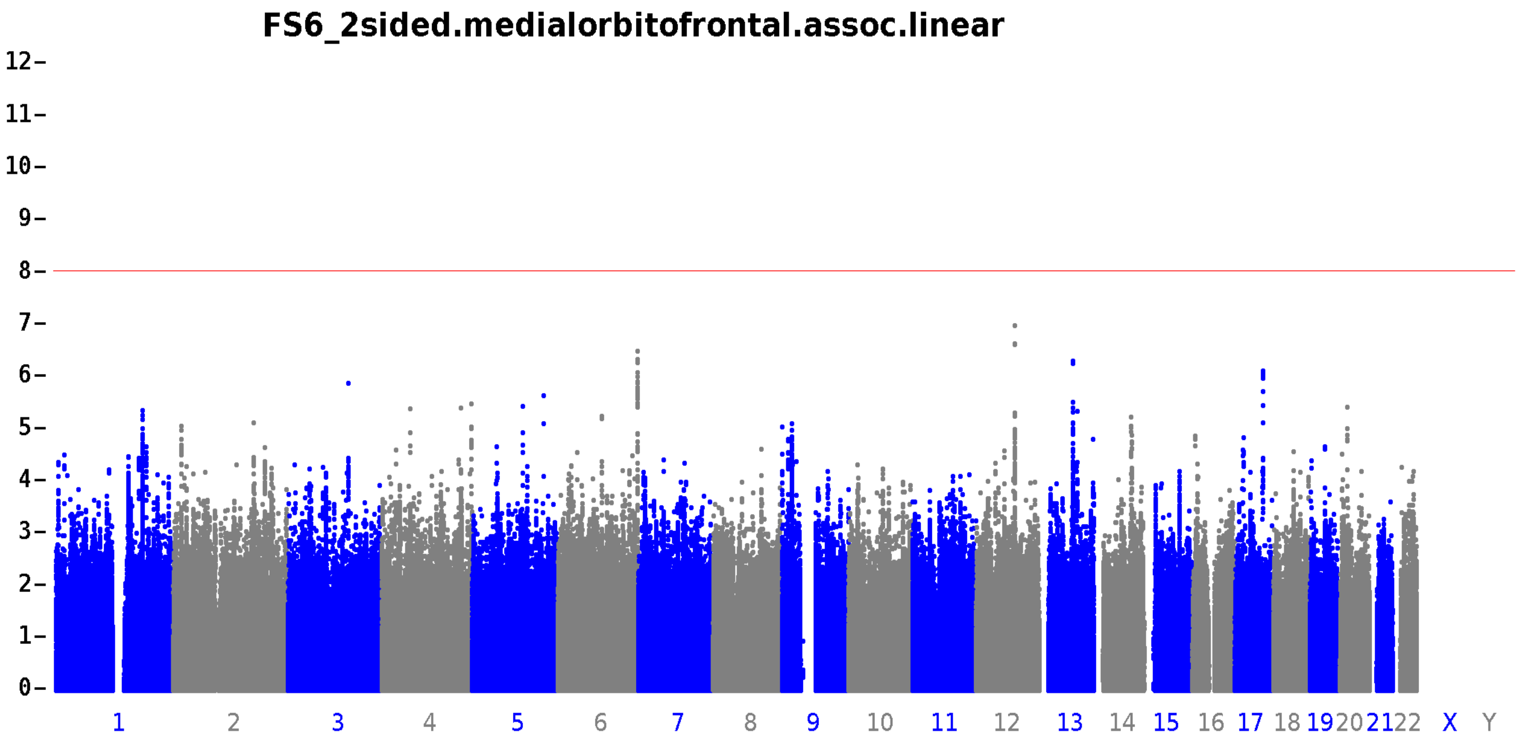

SNP mapped to gene:

CHR	BP	SNP	P	GENE
12	81598584	rs7136590	9.968e-08	ACSS3
12	81599705	rs9888380	2.205e-07	ACSS3
12	81595700	rs11114787	2.297e-07	ACSS3
6	169421443	rs11755456	3.12e-07	missing
6	169413068	rs12215761	4.403e-07	missing
13	71646254	rs1467627	4.723e-07	missing
6	169416743	rs12196251	5.078e-07	missing
13	71642840	rs36017588	5.297e-07	missing
17	59083292	rs150014520	7.258e-07	BCAS3
17	59090930	rs79194109	7.727e-07	BCAS3
6	169420733	rs1322636	7.92e-07	missing
17	59077242	rs77067556	8.277e-07	BCAS3
17	59077516	chr17:59077516:D	8.277e-07	BCAS3
17	59076932	chr17:59076932:D	8.277e-07	BCAS3
17	59081951	rs6504013	8.277e-07	BCAS3
17	59085037	rs76648989	8.277e-07	BCAS3
17	59085393	rs113403510	8.277e-07	BCAS3
17	59084295	rs7223260	8.277e-07	BCAS3
17	59090228	rs79521803	8.277e-07	BCAS3
17	59084278	rs7222834	8.277e-07	BCAS3
17	59090241	rs113671229	8.277e-07	BCAS3
17	59090854	rs80081946	8.277e-07	BCAS3
6	169401021	rs4708569	9.453e-07	missing
6	169400965	rs4708568	9.453e-07	missing

17	59108262	rs16944758	9.613e-07	BCAS3
17	59092076	rs79929685	9.934e-07	BCAS3
17	59096466	rs79277883	1.01e-06	BCAS3
17	59096980	rs113185766	1.01e-06	BCAS3
17	59096535	rs115676780	1.01e-06	BCAS3
6	169429003	rs910690	1.162e-06	missing
3	128650296	chr3:128650296:I	1.244e-06	LOC100132731
6	169399815	rs6938498	1.265e-06	missing
6	169425881	rs12211695	1.519e-06	missing
6	169409603	rs7770399	1.693e-06	missing
6	169429492	chr6:169429492:I	1.703e-06	missing
17	59106971	rs75227573	1.825e-06	BCAS3
17	59107721	rs16944752	1.825e-06	BCAS3
17	59104266	rs16944747	1.825e-06	BCAS3
17	59109065	rs77659861	1.825e-06	BCAS3
17	59098340	rs10491184	1.825e-06	BCAS3
6	169416679	rs12194918	1.87e-06	missing
6	169413804	rs10945358	1.87e-06	missing
6	169411057	rs10945357	1.968e-06	missing
6	169403564	rs2207965	2.138e-06	missing
5	150977057	rs4958275	2.217e-06	missing
6	169425778	rs12203323	2.258e-06	missing
6	169412768	rs11753069	2.36e-06	missing
6	169401047	rs4708570	2.385e-06	missing
6	169421120	rs11752531	2.462e-06	missing
6	169403323	rs2207966	2.49e-06	missing
13	71621978	rs1506585	2.904e-06	missing
6	169418823	rs1322638	3.042e-06	missing
6	169422640	rs13204563	3.106e-06	missing
4	189811357	rs7674523	3.192e-06	missing
17	59114554	rs4335826	3.346e-06	BCAS3
5	107005665	rs77199804	3.507e-06	EFNA5
20	15435203	rs10485526	3.646e-06	MACROD2
6	169422415	rs11757721	3.661e-06	missing
13	71622842	rs9572629	3.764e-06	missing
13	71622496	rs59348011	3.764e-06	missing
4	168080928	rs10024236	3.782e-06	SPOCK3
4	59512591	rs4865342	3.9e-06	missing
1	184508272	rs4651217	4.203e-06	C1orf21
1	184507803	rs7544240	4.203e-06	C1orf21
1	184507549	rs7533466	4.203e-06	C1orf21
13	81190353	rs9574700	4.29e-06	missing
13	71621989	rs1506584	4.505e-06	missing
12	81668675	rs56358071	4.686e-06	PPFIA2
1	184506303	rs12125349	5.173e-06	C1orf21
12	81604773	rs7954225	5.323e-06	ACSS3
12	81604161	rs7968007	5.461e-06	ACSS3

6	93869814	rs9353994	5.512e-06	missing
14	79785699	rs2218786	5.618e-06	NRXN3
6	93881538	rs4288174	6.055e-06	missing
1	184505012	rs12127674	6.295e-06	C1orf21
13	71614837	rs1911141	7.154e-06	missing
17	59103561	rs16944743	7.238e-06	BCAS3
2	170676227	rs1541776	7.287e-06	METTL5
5	150975694	rs4958463	7.43e-06	missing
9	20747070	rs7043296	7.505e-06	FOCAD
13	71615178	rs1911140	7.553e-06	missing
14	79709510	rs4899737	8.265e-06	NRXN3
2	17197349	rs10165361	8.407e-06	missing
14	79727238	rs7153957	8.695e-06	NRXN3
9	871235	rs60085143	8.77e-06	DMRT1
4	189812475	rs12505757	8.77e-06	missing
14	79723290	rs12888606	8.871e-06	NRXN3
20	15442466	rs6079840	9.232e-06	MACROD2
20	15442520	rs6079841	9.232e-06	MACROD2
4	189812524	rs12498968	9.312e-06	missing
1	184514081	rs2144294	9.445e-06	C1orf21
13	71615269	rs2875598	9.498e-06	missing
13	71617775	rs2135488	9.586e-06	missing
12	81610088	rs10862266	9.667e-06	ACSS3
2	17194313	rs4641937	9.948e-06	missing
9	20794591	rs4333699	9.968e-06	FOCAD

Manhattan Plot:

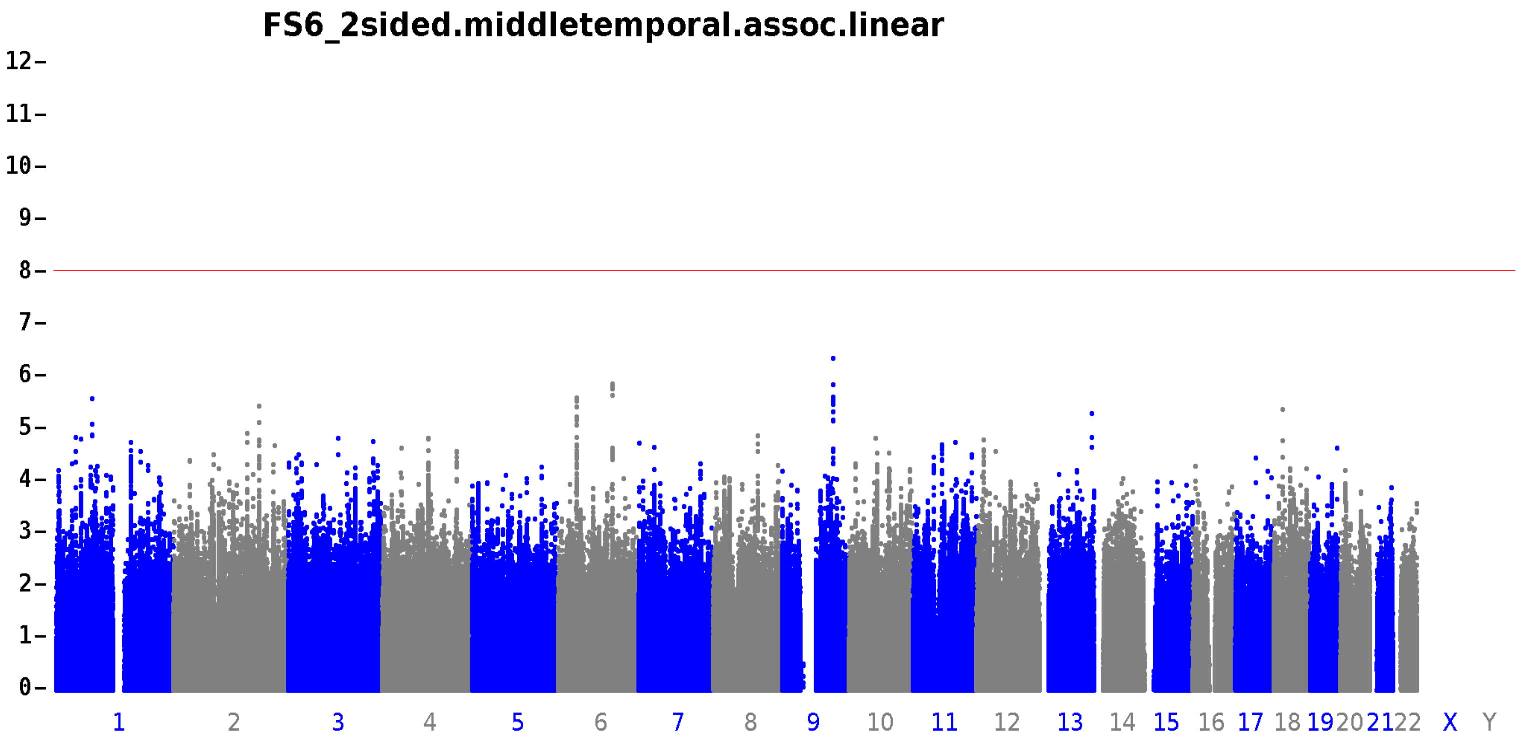

SNP mapped to gene:

CHR	BP	SNP	P	GENE
9	107290451	rs1403810	4.321e-07	missing
6	116775633	rs2640882	1.31e-06	missing
9	107290115	rs10820664	1.381e-06	missing
9	107290126	rs10820665	1.381e-06	missing
6	116773400	rs2213561	1.534e-06	missing
6	116769633	rs578150	1.627e-06	missing
6	116776499	rs2858830	2.199e-06	missing
9	107283708	rs10991289	2.386e-06	missing
6	40217930	rs10947864	2.41e-06	missing
1	77016278	rs315060	2.541e-06	ST6GALNAC3
9	107280763	rs999167	2.658e-06	missing
9	107281102	rs7045936	2.658e-06	missing
9	107275816	rs9775098	2.703e-06	missing
6	40167498	rs9380935	2.786e-06	missing
9	107273553	rs7025484	2.812e-06	missing
9	107278619	chr9:107278619:D	3.279e-06	missing
2	181024868	rs4893897	3.549e-06	missing
6	40169739	rs6904685	3.591e-06	missing
18	19630281	rs12961736	4.118e-06	missing
9	107284126	rs7036108	4.502e-06	missing
13	111123112	rs9555705	4.828e-06	COL4A2
6	40172340	rs9394662	5.676e-06	missing
6	40174390	rs9349139	6.389e-06	missing
9	107274245	rs10820662	6.463e-06	missing

9	107285984	rs1523673	6.791e-06	missing
2	181024612	rs4894162	7.145e-06	missing
1	77015633	rs315061	7.697e-06	ST6GALNAC3
1	77015622	rs402112	7.697e-06	ST6GALNAC3
6	40157228	rs1325543	8.226e-06	missing

Manhattan Plot:

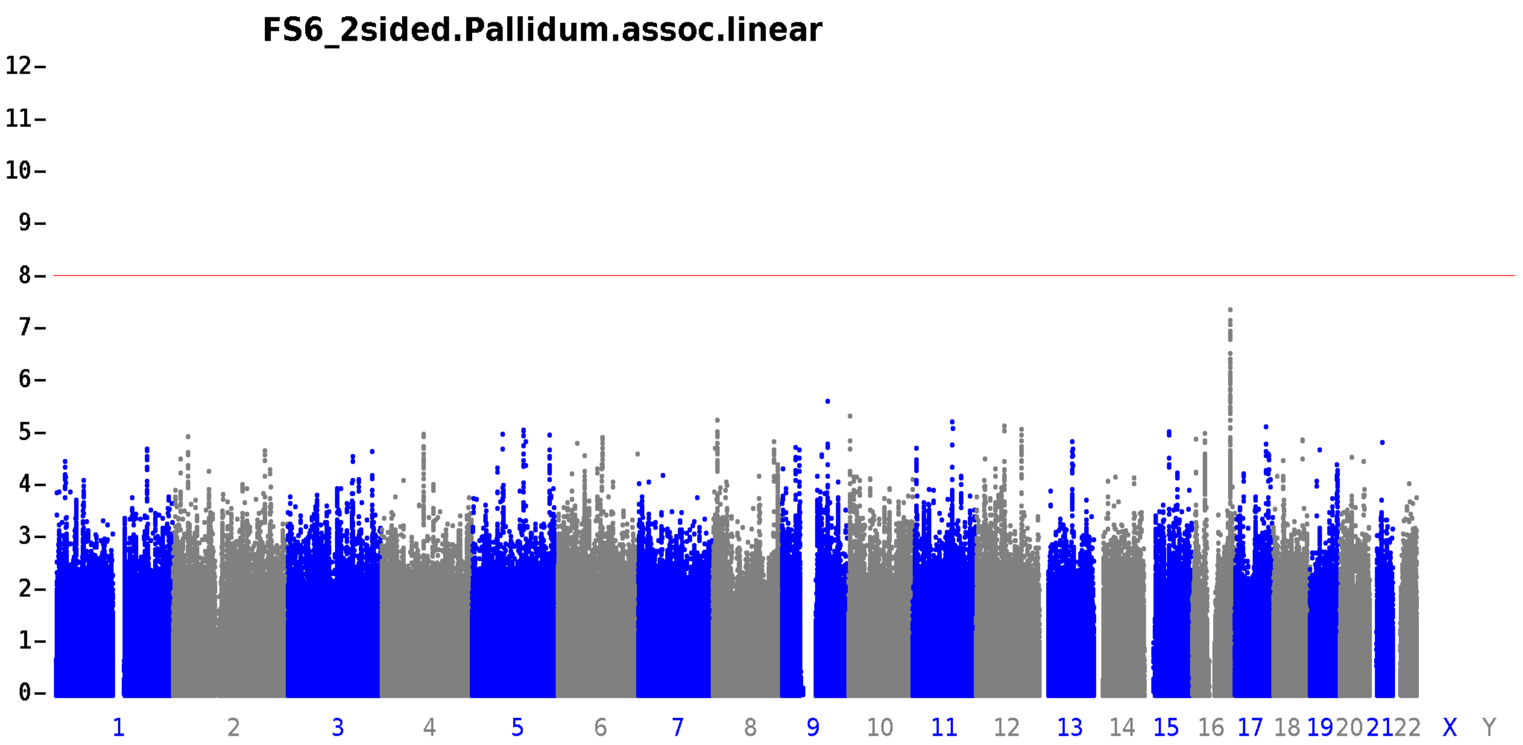

SNP mapped to gene:

CHR	BP	SNP	P	GENE
16	80215561	rs9935652	4.076e-08	missing
16	80213498	rs7201276	6.451e-08	missing
16	80219024	rs9940589	7.592e-08	missing
16	80214690	rs11640831	7.762e-08	missing
16	80209443	rs2169806	1.029e-07	missing
16	80219097	rs9930130	1.153e-07	missing
16	80218291	rs2169804	1.179e-07	missing
16	80217927	rs1904186	1.232e-07	missing
16	80212969	rs1382389	1.252e-07	missing
16	80217126	rs9939067	1.339e-07	missing
16	80222686	rs8061813	1.504e-07	missing
16	80223533	rs8047391	1.504e-07	missing
16	80192458	rs8045952	2.774e-07	missing
16	80191607	rs12102804	3.527e-07	missing
16	80217906	rs1904185	3.717e-07	missing
16	80212781	rs8060188	3.797e-07	missing
16	80193726	rs7193651	3.827e-07	missing
16	80227255	rs7186149	4.286e-07	missing
16	80192347	rs8045772	5.103e-07	missing
16	80233498	rs9319550	6.331e-07	missing
16	80193250	rs7205467	7.37e-07	missing
16	80194358	rs4889112	7.764e-07	missing
16	80229337	rs9944373	7.886e-07	missing
16	80213480	rs12597962	9.073e-07	missing

16	80201444	rs9673176	9.382e-07	missing
16	80211844	rs9935498	1.045e-06	missing
16	80196025	rs1871209	1.307e-06	missing
16	80211981	rs11646831	1.403e-06	missing
16	80201595	rs9673185	1.735e-06	missing
16	80231733	rs13380441	1.914e-06	missing
16	80230474	rs12930060	2.161e-06	missing
9	96186490	rs12555047	2.233e-06	missing
16	80232682	rs9934453	2.334e-06	missing
16	80240329	rs1564219	2.882e-06	missing
16	80236034	rs1904188	3.187e-06	missing
16	80188197	rs9926605	3.406e-06	missing
16	80237726	rs1564220	3.653e-06	missing
16	80197159	rs4889115	3.938e-06	missing
16	80237041	rs28615887	3.942e-06	missing
10	2357484	rs5020023	4.3e-06	missing
8	9286975	rs11997533	5.152e-06	missing
16	80246910	rs11648758	5.163e-06	missing
16	80242310	rs1904187	5.18e-06	missing
11	84825044	rs540153	5.649e-06	DLG2
12	60242836	chr12:60242836:D	6.763e-06	missing
17	66402256	rs11870010	6.926e-06	ARSG
16	80244411	rs8043986	7.135e-06	missing
16	80239382	rs9938515	7.318e-06	missing
11	85495195	rs7925465	7.426e-06	SYTL2
16	80247432	rs9889013	7.586e-06	missing
16	80244404	rs2169807	7.627e-06	missing
12	96384122	rs60881496	7.745e-06	HAL
5	108587146	rs72793038	7.991e-06	LOC285638
12	60253082	rs4540857	8.257e-06	missing
5	108624482	rs2112315	8.4e-06	LOC285638
15	53527494	rs59373856	8.547e-06	missing
8	9286018	rs2085639	8.79e-06	missing
16	26103397	rs11861027	9.254e-06	HS3ST4
8	9276945	rs10107959	9.264e-06	missing
5	65190626	rs251292	9.534e-06	missing
8	9276870	rs10107932	9.548e-06	missing
4	88360229	rs7665115	9.721e-06	NUDT9
5	164642051	rs17392297	9.921e-06	missing
12	96384139	rs57822148	9.971e-06	HAL
15	53533456	chr15:53533456:D	9.993e-06	missing

Manhattan Plot:

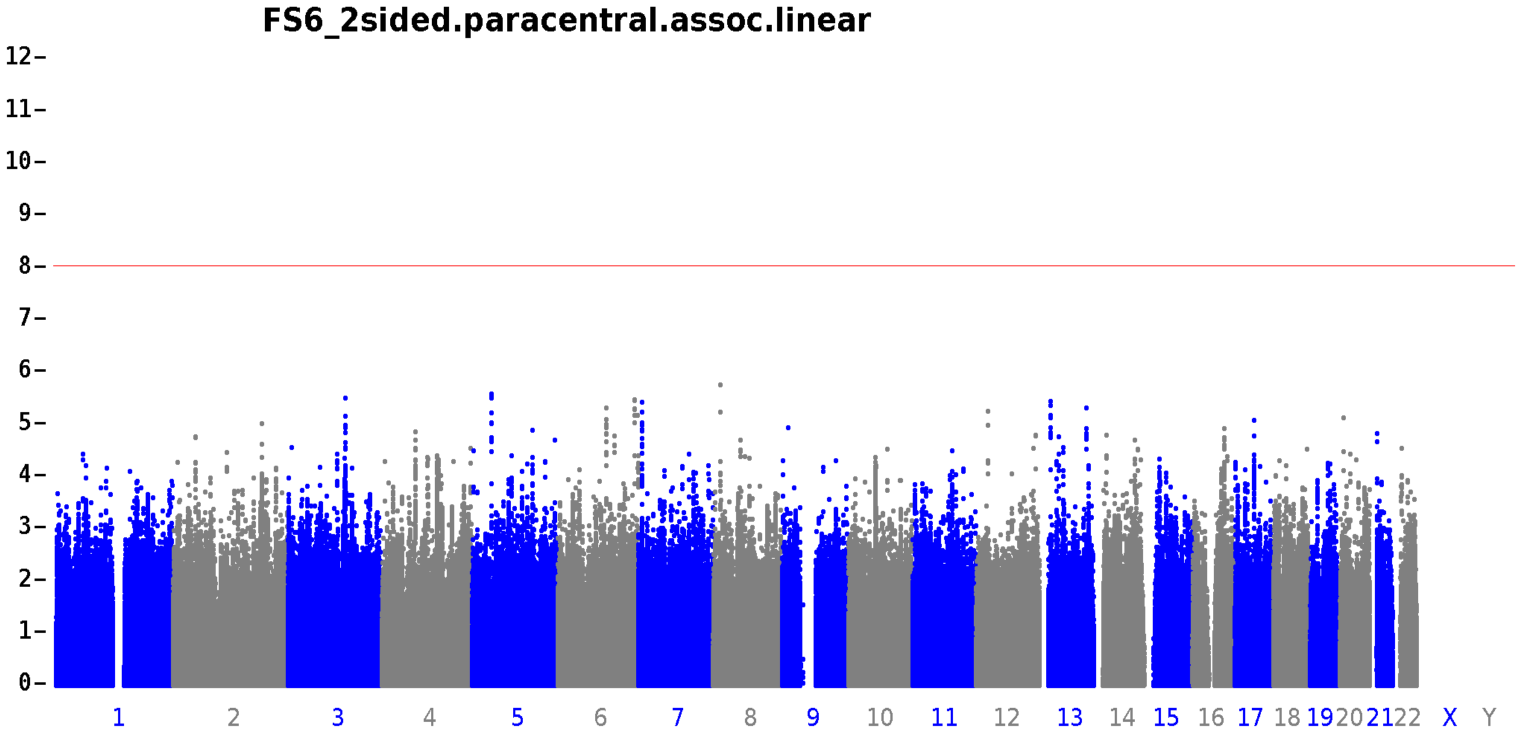

SNP mapped to gene:

CHR	BP	SNP	P	GENE
8	15843195	rs75000616	1.715e-06	missing
5	40870337	rs62357613	2.552e-06	missing
5	40869727	rs75353980	2.552e-06	missing
5	40866559	rs34153616	2.552e-06	missing
5	40870505	rs62357614	2.552e-06	missing
5	40875611	rs62357615	2.653e-06	missing
5	40861790	rs62357609	2.976e-06	missing
5	40860275	rs79220182	3.033e-06	missing
3	121100414	rs17740072	3.038e-06	STXBP5L
6	163530356	rs75846880	3.278e-06	PACRG
6	163524816	rs17381249	3.322e-06	PACRG
6	163527859	rs11964879	3.322e-06	PACRG
6	163525355	rs6917102	3.322e-06	PACRG
13	23614655	rs9578535	3.461e-06	missing
7	8939505	rs11770269	3.663e-06	missing
13	23614385	rs9507003	4.217e-06	missing
6	103469369	rs9404300	4.729e-06	missing
13	99426429	rs9557052	4.774e-06	missing
6	163534162	rs78432026	4.863e-06	PACRG
6	163522910	rs75245740	4.97e-06	PACRG
12	25422717	rs7313581	5.336e-06	missing
7	8944009	rs4449704	5.561e-06	missing
8	15841656	rs11203744	5.707e-06	missing
5	40850073	rs62357602	5.759e-06	CARD6

6	170654894	rs4710716	6.399e-06	FAM120B
13	23613264	rs9578530	6.479e-06	missing
13	23613298	rs9578532	6.479e-06	missing
6	163531407	rs16894498	6.509e-06	PACRG
3	121136623	rs2169300	6.688e-06	STXBP5L
20	6950918	rs142943889	7.19e-06	missing
13	23612546	rs9317418	7.283e-06	missing
6	103483902	rs9404302	7.768e-06	missing
6	103481466	rs6900275	7.918e-06	missing
17	40414762	chr17:40414762:I	8.219e-06	STAT5B
6	163524132	rs74970617	8.998e-06	PACRG
7	8948182	rs12702795	9.041e-06	missing
7	8948804	rs1159394	9.041e-06	missing
5	40907222	rs62357647	9.087e-06	missing
2	187391701	rs13001360	9.322e-06	missing
5	40922660	rs78602479	9.325e-06	C7
5	40915039	rs11741864	9.325e-06	C7
5	40908194	rs12153063	9.325e-06	missing
6	103479836	chr6:103479836:D	9.637e-06	missing

Manhattan Plot:

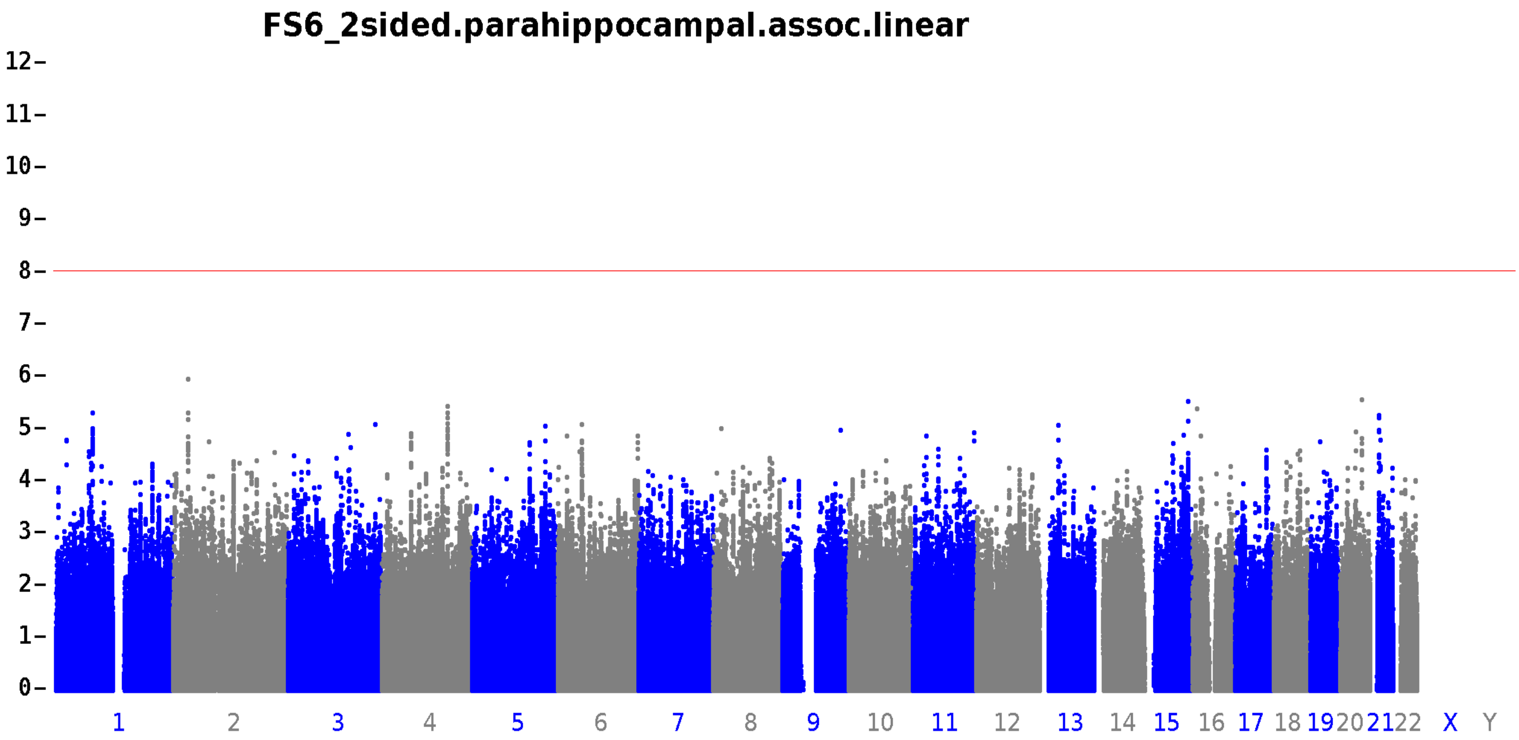

SNP mapped to gene:

CHR	BP	SNP	P	GENE
2	31587511	rs994727	1.066e-06	XDH
20	45884385	rs13037018	2.663e-06	ZMYND8
15	92824927	rs11074058	2.862e-06	DUXAP6
4	139687927	rs10030317	3.502e-06	missing
16	9416637	rs61666746	3.85e-06	missing
2	31587235	rs1018505	4.634e-06	XDH
1	78772009	rs2104621	4.659e-06	MGC27382
4	139693668	rs28726321	4.66e-06	missing
4	139681324	rs28731133	4.694e-06	missing
21	19352736	rs1108858	5.188e-06	missing
21	19368612	rs2824593	5.736e-06	missing
21	19360456	rs67487312	5.863e-06	missing
4	139682312	rs10006215	5.873e-06	missing
2	31574334	rs7571426	6.37e-06	XDH
15	92829452	rs28566962	6.758e-06	NPM1P5
4	139657611	rs28397758	7.517e-06	missing
4	139659423	rs10026190	7.517e-06	missing
4	139656345	rs28706832	7.517e-06	missing
4	139655744	rs1505866	7.517e-06	missing
6	50734755	rs12174225	7.69e-06	TFAP2D
3	184412702	rs7621895	7.836e-06	missing
13	39469339	rs4146173	8.112e-06	missing
5	154780954	rs139648861	8.252e-06	missing
1	78786208	rs7521129	9.199e-06	MGC27382

4	139689421	rs4463111	9.317e-06	missing
4	139689626	rs10026304	9.317e-06	missing
8	17222900	rs10107226	9.498e-06	MTMR7
4	139690884	rs72941644	9.734e-06	missing
4	139691338	rs28420916	9.734e-06	missing
4	139691336	rs28637574	9.734e-06	missing

Manhattan Plot:

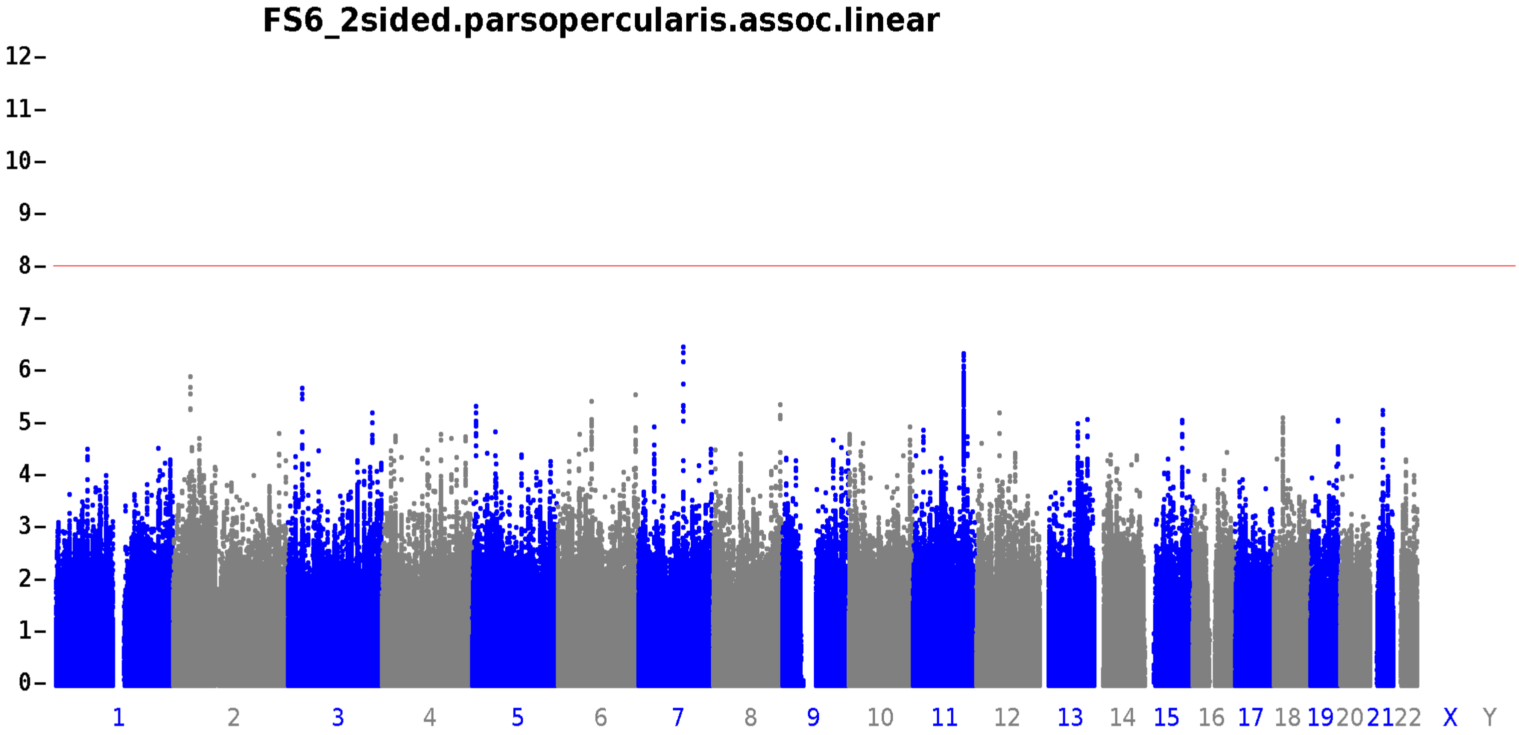

SNP mapped to gene:

CHR	BP	SNP	P	GENE
7	95266519	rs73393947	3.138e-07	missing
7	95268056	rs55850130	4.035e-07	missing
11	107117691	rs2216998	4.258e-07	missing
11	107197235	rs1046095	4.707e-07	CWF19L2
11	107155911	rs7929345	5.626e-07	missing
7	95269172	rs10264647	6.162e-07	missing
11	107155909	rs7929343	7.329e-07	missing
11	107262510	rs11212197	7.513e-07	CWF19L2
11	107196729	rs11212159	7.637e-07	missing
11	107197256	rs1046094	7.637e-07	CWF19L2
11	107197640	rs3758911	7.637e-07	CWF19L2
11	107196918	rs11212160	7.637e-07	missing
11	107159302	rs11212132	7.865e-07	missing
11	107179942	rs10890701	9.881e-07	missing
11	107181061	rs10789600	1.022e-06	missing
11	107230895	rs11212181	1.084e-06	CWF19L2
11	107178010	rs10749893	1.112e-06	missing
11	107178969	rs10890700	1.153e-06	missing
11	107180312	rs10749894	1.153e-06	missing
11	107179871	rs10789599	1.153e-06	missing
2	36124322	rs2949055	1.171e-06	missing
2	36124316	rs2949054	1.171e-06	missing
11	107196191	rs1490945	1.176e-06	missing
11	107198839	rs11212164	1.176e-06	CWF19L2

11	107196471	rs11212158	1.176e-06	missing
11	107181440	chr11:107181440:I	1.253e-06	missing
11	107181581	rs10749895	1.253e-06	missing
11	107180885	rs10890702	1.319e-06	missing
11	107180781	chr11:107180781:D	1.323e-06	missing
11	107276281	chr11:107276281:D	1.383e-06	CWF19L2
11	107177213	rs10890696	1.384e-06	missing
11	107173720	rs11212142	1.384e-06	missing
11	107175334	rs12285607	1.384e-06	missing
11	107181776	rs10789603	1.393e-06	missing
11	107278683	rs17106875	1.406e-06	CWF19L2
11	107175040	rs10789597	1.412e-06	missing
11	107181419	rs10789601	1.416e-06	missing
11	107165649	rs10890695	1.455e-06	missing
11	107181129	rs10890703	1.463e-06	missing
11	107201451	rs6588964	1.503e-06	CWF19L2
11	107141847	rs12287772	1.517e-06	missing
11	107149083	rs10749890	1.548e-06	missing
11	107147779	rs10890692	1.548e-06	missing
11	107149932	rs10454489	1.548e-06	missing
11	107147483	rs11212117	1.548e-06	missing
11	107147347	rs11212116	1.548e-06	missing
11	107146352	rs10890689	1.548e-06	missing
11	107156880	rs10789593	1.561e-06	missing
11	107201689	rs6588965	1.562e-06	CWF19L2
11	107161117	rs10789594	1.593e-06	missing
11	107157977	rs2355840	1.597e-06	missing
11	107154315	rs10789592	1.638e-06	missing
11	107178053	chr11:107178053:I	1.652e-06	missing
7	95265377	rs10275635	1.655e-06	missing
11	107178890	rs10890699	1.661e-06	missing
11	107178641	rs10890697	1.661e-06	missing
11	107164499	rs7131522	1.773e-06	missing
11	107168926	rs11212141	1.773e-06	missing
2	36123824	rs2949052	1.858e-06	missing
11	107275251	rs11212206	1.877e-06	CWF19L2,SMARCE1P1
3	30571935	chr3:30571935:I	1.935e-06	missing
11	107179280	rs11212143	2e-06	missing
11	107229356	rs11212180	2.073e-06	CWF19L2
11	107227980	rs11212179	2.073e-06	CWF19L2
11	107196983	rs10890718	2.103e-06	missing
11	107146900	rs10890690	2.163e-06	missing
11	107232725	rs655448	2.226e-06	CWF19L2
11	107153581	rs112617433	2.239e-06	missing
11	107178604	rs10789598	2.248e-06	missing
11	107224718	rs4343000	2.403e-06	CWF19L2
11	107181455	rs10789602	2.466e-06	missing

11	107178806	rs10890698	2.469e-06	missing
2	36127010	rs76305760	2.487e-06	missing
2	36126822	rs36174987	2.487e-06	missing
11	107214607	rs11212173	2.507e-06	CWF19L2
11	107219307	rs11212175	2.507e-06	CWF19L2
3	30570719	rs11716372	2.559e-06	missing
6	165326879	rs12176440	2.604e-06	missing
11	107165279	rs11212138	2.645e-06	missing
11	107145666	rs11212114	2.657e-06	missing
11	107143393	rs11212112	2.657e-06	missing
11	107143137	rs1388171	2.657e-06	missing
11	107143502	rs11212113	2.657e-06	missing
11	107142856	rs1388170	2.657e-06	missing
11	107142746	rs10890688	2.657e-06	missing
11	107151059	rs10749891	3.062e-06	missing
11	107180591	rs112051111	3.116e-06	missing
3	30571886	rs6783800	3.165e-06	missing
11	107180522	rs11604771	3.2e-06	missing
11	107231605	rs11212182	3.203e-06	CWF19L2
11	107137051	rs10890684	3.266e-06	missing
11	107232596	rs11607304	3.4e-06	CWF19L2
11	107138365	rs11212111	3.404e-06	missing
6	71453655	chr6:71453655:I	3.482e-06	SMAP1
11	107207602	rs10890721	3.549e-06	CWF19L2
11	107180453	chr11:107180453:D	3.579e-06	missing
11	107195502	rs10749901	3.607e-06	missing
11	107214328	chr11:107214328:I	3.767e-06	CWF19L2
11	107222776	rs72990443	3.875e-06	CWF19L2
8	141128357	rs4736138	4.097e-06	TRAPPC9
11	107259071	rs10789621	4.14e-06	CWF19L2
7	95266858	rs7800814	4.207e-06	missing
7	95265935	chr7:95265935:D	4.207e-06	missing
5	7573966	rs34930574	4.316e-06	ADCY2
7	95266161	rs1468146	4.335e-06	missing
7	95266209	rs1468147	4.335e-06	missing
2	36126942	rs146568685	4.819e-06	missing
2	36128741	rs2949062	5.02e-06	missing
11	107203904	rs7358308	5.171e-06	CWF19L2
11	107198610	rs11212162	5.175e-06	CWF19L2
11	107306812	rs671493	5.187e-06	CWF19L2
21	26210484	rs73147817	5.274e-06	missing
11	107208661	chr11:107208661:I	5.396e-06	CWF19L2
11	107208764	rs12223855	5.396e-06	CWF19L2
7	95267882	rs73393959	5.434e-06	missing
7	95267719	rs73393957	5.434e-06	missing
7	95267967	chr7:95267967:I	5.434e-06	missing
7	95267710	rs73708090	5.434e-06	missing

11	107202604	rs11212166	5.646e-06	CWF19L2
3	179305654	chr3:179305654:I	5.745e-06	ACTL6A
11	107211045	rs12285491	5.804e-06	CWF19L2
5	7583156	rs12519539	5.805e-06	ADCY2
5	7565152	rs4530734	5.825e-06	ADCY2
12	49156220	rs28889716	5.832e-06	LINC00935
11	107297952	chr11:107297952:I	5.852e-06	CWF19L2
11	107275522	rs11212207	5.876e-06	CWF19L2
11	107192257	rs999985	5.948e-06	missing
21	26209226	rs9981221	6.306e-06	missing
11	107273938	rs7358296	6.423e-06	CWF19L2
8	141130066	rs6994441	6.535e-06	TRAPPC9
11	107184478	rs10789604	6.98e-06	missing
18	19659465	rs34157981	7.236e-06	missing
11	107189929	chr11:107189929:I	7.292e-06	missing
8	141121745	rs10101006	7.49e-06	TRAPPC9
6	71485345	rs12202909	7.689e-06	SMAP1
13	101333859	rs9518135	7.93e-06	missing
11	107205426	rs10789620	7.996e-06	CWF19L2
11	107318279	rs590699	8.08e-06	CWF19L2
15	80141806	rs36060920	8.08e-06	MTHFS,ST20-MTHFS
15	80143118	rs8033289	8.08e-06	MTHFS,ST20-MTHFS
19	58017362	rs2005684	8.129e-06	ZNF773
7	95269426	rs10249748	8.271e-06	missing
7	95268916	rs10249116	8.271e-06	missing
7	95268728	chr7:95268728:D	8.271e-06	missing
5	7573787	rs113664928	8.428e-06	ADCY2
19	58027460	rs4801498	8.429e-06	ZNF773
11	107213236	rs10890723	8.6e-06	CWF19L2
11	107211334	rs12286915	8.6e-06	CWF19L2
11	107213628	rs56393657	8.6e-06	CWF19L2
11	107213279	rs7350479	8.6e-06	CWF19L2
11	107276280	chr11:107276280:D	8.602e-06	CWF19L2
11	107198731	rs11212163	8.753e-06	CWF19L2
6	71423441	rs1577108	8.858e-06	SMAP1
15	80151536	rs12912711	8.9e-06	MTHFS,ST20-MTHFS
5	7587233	chr5:7587233:D	8.916e-06	ADCY2
3	179336625	rs13325172	8.948e-06	NDUFB5
18	19641628	rs9960339	9.006e-06	missing
13	81161098	rs17072871	9.31e-06	missing
11	107186294	rs12286342	9.479e-06	missing
11	107252208	rs10502087	9.536e-06	CWF19L2
5	7574367	rs12517386	9.78e-06	ADCY2
11	107183505	rs10890704	9.816e-06	missing
6	71512875	rs139847311	9.916e-06	SMAP1

Manhattan Plot:

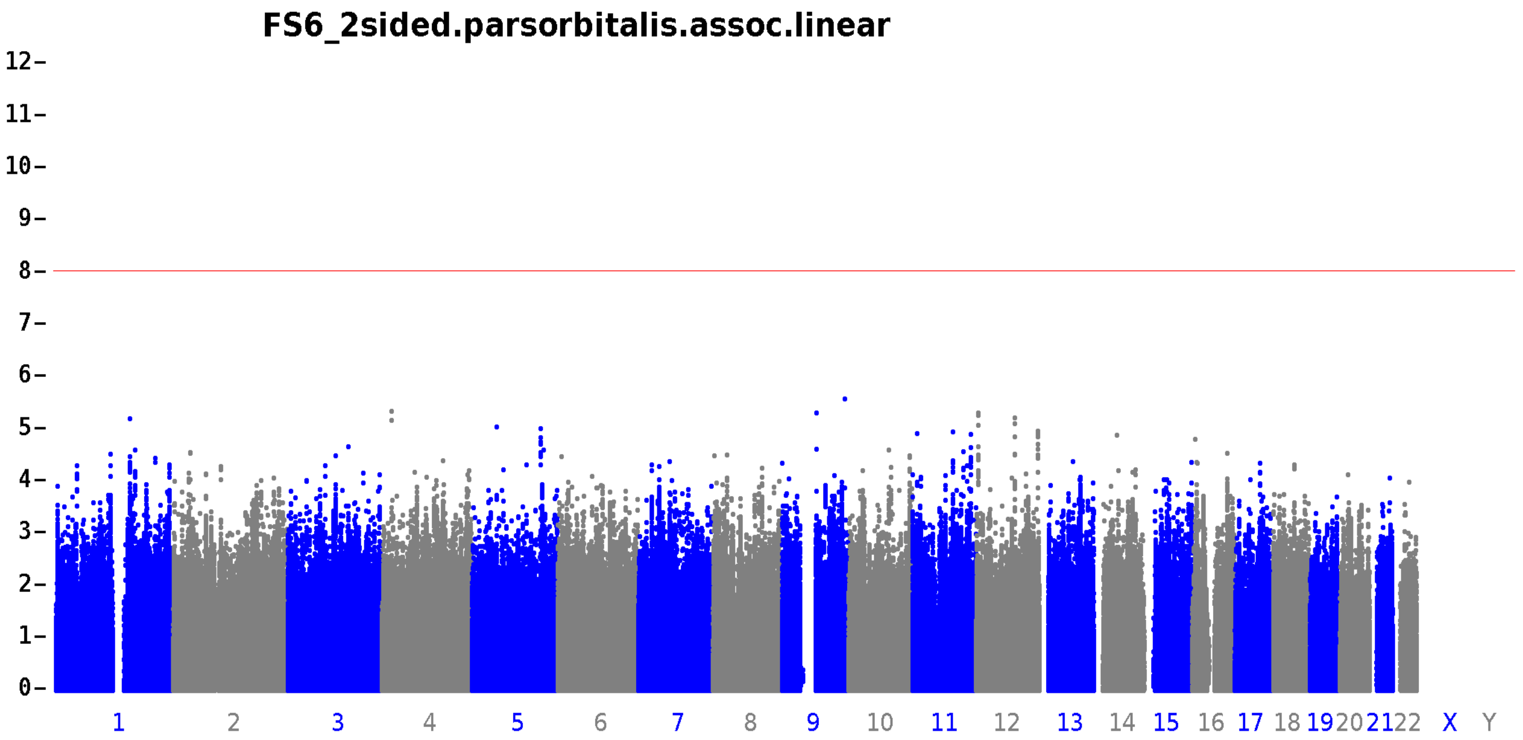

SNP mapped to gene:

CHR	BP	SNP	P	GENE
9	132515081	rs2241271	2.49e-06	PTGES
4	20527864	rs7690660	4.306e-06	SLIT2
12	3877127	rs35021047	4.666e-06	missing
9	72605579	rs62572599	4.72e-06	missing
12	3876761	rs12813576	4.833e-06	missing
12	3876721	rs7980884	5.312e-06	missing
12	81595700	rs11114787	5.784e-06	ACSS3
1	157276797	rs79271885	5.945e-06	missing
1	157276132	rs7527191	5.945e-06	missing
4	20515441	rs60029443	6.532e-06	SLIT2
12	81598584	rs7136590	7.433e-06	ACSS3
12	3876394	chr12:3876394:D	8.139e-06	missing
5	52022713	rs830886	8.743e-06	missing
5	145749362	rs28559912	9.294e-06	missing

Manhattan Plot:

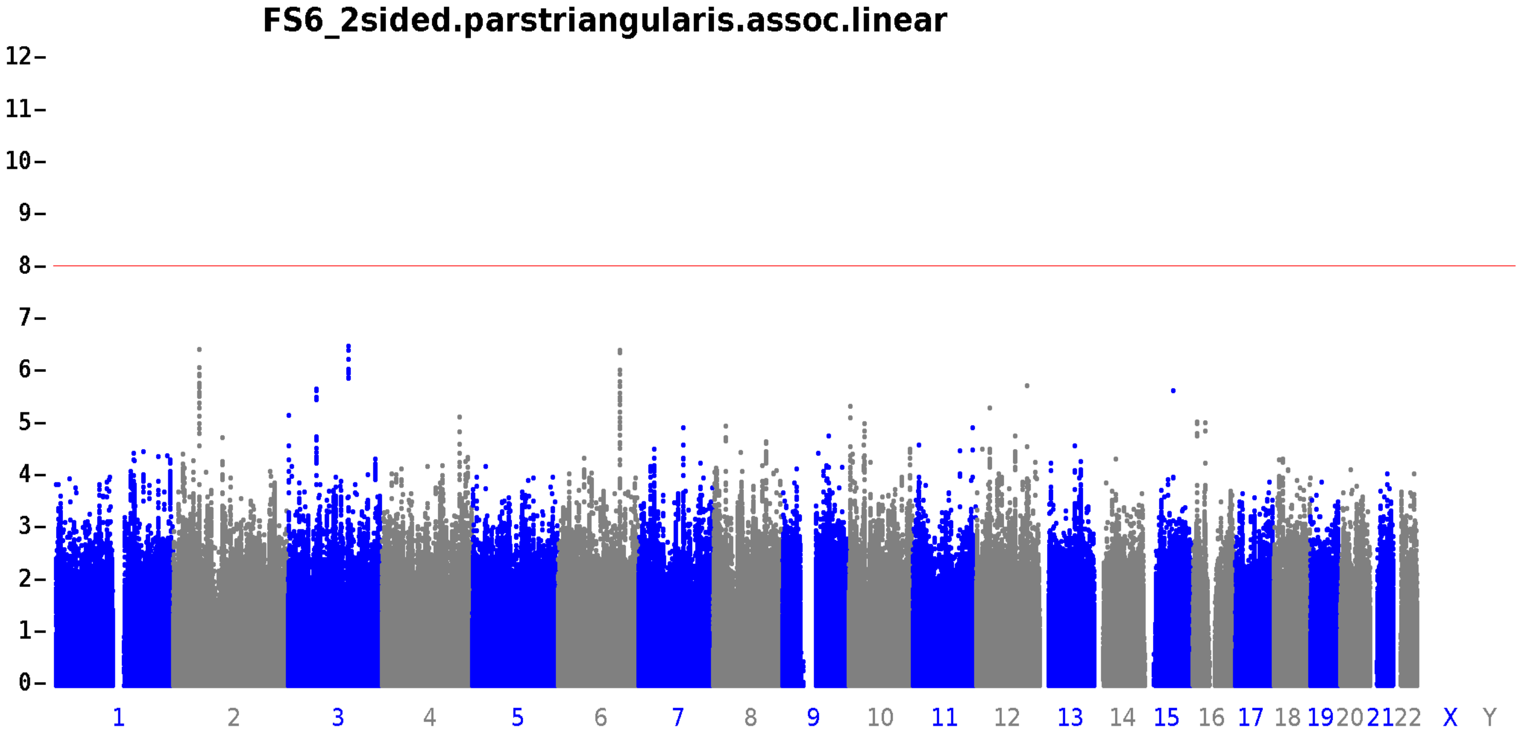

SNP mapped to gene:

CHR	BP	SNP	P	GENE
3	127440619	rs11711251	3.071e-07	MGLL
2	54717713	rs17045917	3.531e-07	SPTBN1
6	131298491	chr6:131298491:D	3.628e-07	EPB41L2
3	127439381	rs526862	3.678e-07	MGLL
3	127439043	rs523925	3.678e-07	MGLL
6	131293112	rs6910656	4.079e-07	EPB41L2
6	131284260	rs2095961	4.094e-07	EPB41L2
3	127437631	rs600884	5.441e-07	MGLL
3	127440142	rs555183	5.481e-07	MGLL
2	54714553	rs17039554	7.957e-07	SPTBN1
3	127451533	rs11717519	8.595e-07	MGLL
6	131291390	rs6921396	8.96e-07	EPB41L2
3	127446639	rs9877209	9.253e-07	MGLL
3	127448047	rs10512693	1.013e-06	MGLL
2	54718805	rs4671943	1.057e-06	SPTBN1
6	131298481	rs6922177	1.062e-06	EPB41L2
6	131298913	rs9321263	1.062e-06	EPB41L2
2	54717168	rs4561702	1.119e-06	SPTBN1
2	54711568	rs10205013	1.138e-06	SPTBN1
3	127447302	rs10934819	1.203e-06	MGLL
3	127447884	rs11713989	1.283e-06	MGLL
6	131288074	rs7741148	1.49e-06	EPB41L2
2	54703114	rs72618661	1.583e-06	SPTBN1
2	54707690	rs72618662	1.639e-06	SPTBN1

2	54707562	rs6754429	1.639e-06	SPTBN1
2	54707020	rs7599380	1.639e-06	SPTBN1
12	107776693	rs11113296	1.729e-06	BTBD11
12	107776829	rs12307408	1.729e-06	BTBD11
6	131289917	rs7769678	1.731e-06	EPB41L2
6	131299179	rs1811949	1.731e-06	EPB41L2
6	131291697	rs9385568	1.731e-06	EPB41L2
6	131290474	rs9372985	1.838e-06	EPB41L2
2	54722297	chr2:54722297:D	1.891e-06	SPTBN1
2	54710181	rs4671218	1.899e-06	SPTBN1
3	60921746	rs78693890	2.008e-06	FHIT
15	60439578	rs12443021	2.146e-06	missing
3	60932376	rs61439091	2.199e-06	FHIT
3	60932115	rs67072589	2.199e-06	FHIT
2	54709683	rs6545409	2.329e-06	SPTBN1
6	131298531	rs6922045	2.405e-06	EPB41L2
6	131166004	rs2005012	2.409e-06	EPB41L2
2	54722301	chr2:54722301:D	2.688e-06	SPTBN1
2	54705007	rs74351288	2.858e-06	SPTBN1
6	131295959	rs6908980	2.873e-06	EPB41L2
3	60891684	rs73097839	2.939e-06	FHIT
6	131161504	rs1043596	2.983e-06	EPB41L2
3	60901712	rs60414361	3.26e-06	FHIT
6	131178099	rs928592	3.322e-06	EPB41L2
2	54700653	chr2:54700653:D	3.838e-06	SPTBN1
6	131276473	chr6:131276473	4.039e-06	EPB41L2
10	3684211	rs59679352	4.432e-06	missing
12	28772395	rs73091543	4.692e-06	missing
2	54704203	rs12477221	4.743e-06	SPTBN1
6	131285267	rs9402303	5.559e-06	EPB41L2
3	1670301	rs4143127	6.503e-06	missing
2	54688767	rs72618660	6.742e-06	SPTBN1
4	164455255	rs17472994	7.083e-06	MARCH1
10	3689910	chr10:3689910:D	7.168e-06	missing
6	131183715	rs933063	7.325e-06	EPB41L2
16	8302778	rs12919950	8.57e-06	missing
16	8302797	rs12923884	8.57e-06	missing
6	131276945	rs2297853	8.684e-06	EPB41L2
6	131163660	rs12194612	8.768e-06	EPB41L2
16	26763226	rs11862783	9.107e-06	missing
16	8308802	rs77441842	9.195e-06	missing
10	32264033	rs1775725	9.463e-06	missing
2	54708408	rs10171331	9.496e-06	SPTBN1

Manhattan Plot:

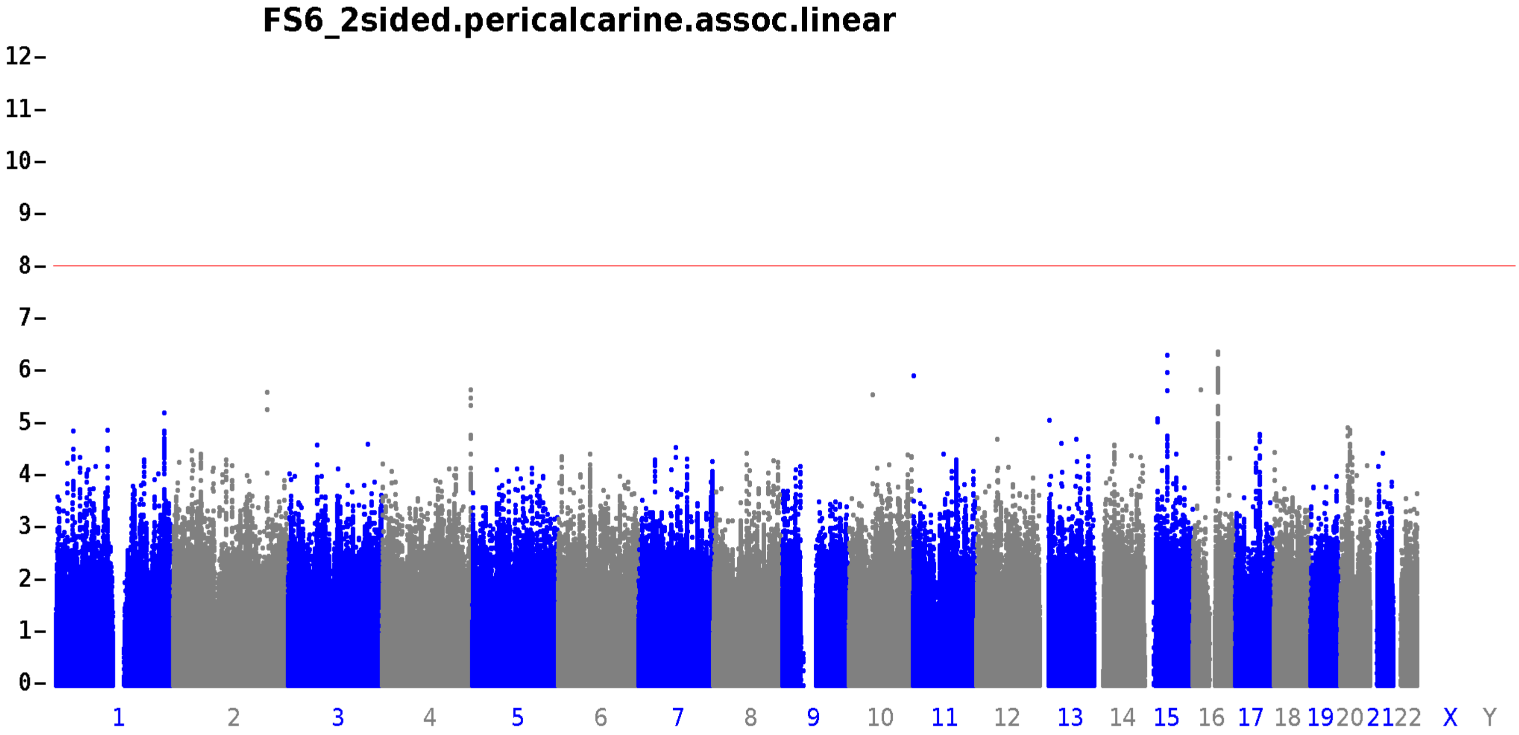

SNP mapped to gene:

CHR	BP	SNP	P	GENE
16	52638503	rs3104788	3.911e-07	CASC16
16	52622419	rs3112629	4.482e-07	CASC16
15	47901737	rs10152500	4.524e-07	SEMA6D
16	52620577	rs3112633	8.173e-07	CASC16
16	52631717	rs3112617	9.606e-07	CASC16
16	52631964	rs11075551	9.606e-07	CASC16
16	52631747	rs3112616	9.606e-07	CASC16
16	52632350	rs3112615	9.606e-07	CASC16
16	52631492	chr16:52631492:I	9.606e-07	CASC16
16	52631462	rs3104776	9.606e-07	CASC16
15	47903174	rs74014029	9.909e-07	SEMA6D
16	52631186	rs3112618	1.045e-06	CASC16
16	52633652	rs3104778	1.047e-06	CASC16
16	52635164	rs3112612	1.047e-06	CASC16
16	52633657	rs3104779	1.047e-06	CASC16
16	52629702	rs3104774	1.077e-06	CASC16
16	52628537	rs11860769	1.077e-06	CASC16
16	52628564	rs11861036	1.077e-06	CASC16
16	52628418	rs11860998	1.077e-06	CASC16
16	52629326	rs3104772	1.077e-06	CASC16
16	52629503	rs3112622	1.077e-06	CASC16
16	52629827	rs3104775	1.077e-06	CASC16
16	52630145	rs7203671	1.077e-06	CASC16
11	2324347	rs55648810	1.139e-06	TSPAN32

16	52625200	rs3112626	1.198e-06	CASC16
16	52626396	rs3104768	1.315e-06	CASC16
16	52608263	rs3112562	1.324e-06	CASC16
16	52626551	rs3112624	1.369e-06	CASC16
16	52627526	rs3112623	1.369e-06	CASC16
16	52627231	rs3104769	1.406e-06	CASC16
16	52629436	rs3104773	1.421e-06	CASC16
16	52603028	rs3104751	1.523e-06	CASC16
16	52603911	rs3104753	1.532e-06	CASC16
16	52637363	rs3104786	1.679e-06	CASC16
16	52637543	rs3104787	1.679e-06	CASC16
16	52612619	rs3112638	1.699e-06	CASC16
16	52599394	rs3104744	1.908e-06	CASC16
16	52638663	rs3104790	1.954e-06	CASC16
16	52638962	rs3104791	2.044e-06	CASC16
4	188125013	rs9790438	2.116e-06	missing
16	16985541	rs74009651	2.144e-06	missing
16	52638548	rs3104789	2.178e-06	CASC16
15	47902502	rs28431305	2.208e-06	SEMA6D
16	52632631	rs3104777	2.259e-06	CASC16
16	52632897	rs45500599	2.259e-06	CASC16
16	52605096	rs3481	2.259e-06	CASC16
16	52606237	rs3104755	2.281e-06	CASC16
2	199050088	rs75402199	2.363e-06	missing
16	52633914	rs79346458	2.37e-06	CASC16
10	50312845	chr10:50312845:D	2.615e-06	VSTM4
4	188128286	rs55912730	3.018e-06	missing
4	188128188	rs59005743	4.135e-06	missing
4	188132177	rs73874309	4.162e-06	missing
16	52612552	chr16:52612552:I	4.311e-06	CASC16
16	52616366	chr16:52616366:I	4.692e-06	CASC16
2	199033012	rs77075612	4.983e-06	missing
16	52613581	rs3104763	5.269e-06	CASC16
16	52612550	chr16:52612550:I	5.459e-06	CASC16
1	229701489	rs35421764	5.762e-06	missing
16	52612044	rs1109951	6.147e-06	CASC16
15	26804131	rs61996546	7.447e-06	GABRB3
13	20728090	rs12428977	8.179e-06	GJA3
15	26801317	rs10444887	8.715e-06	GABRB3
16	52636365	rs3104784	9.307e-06	CASC16

Manhattan Plot:

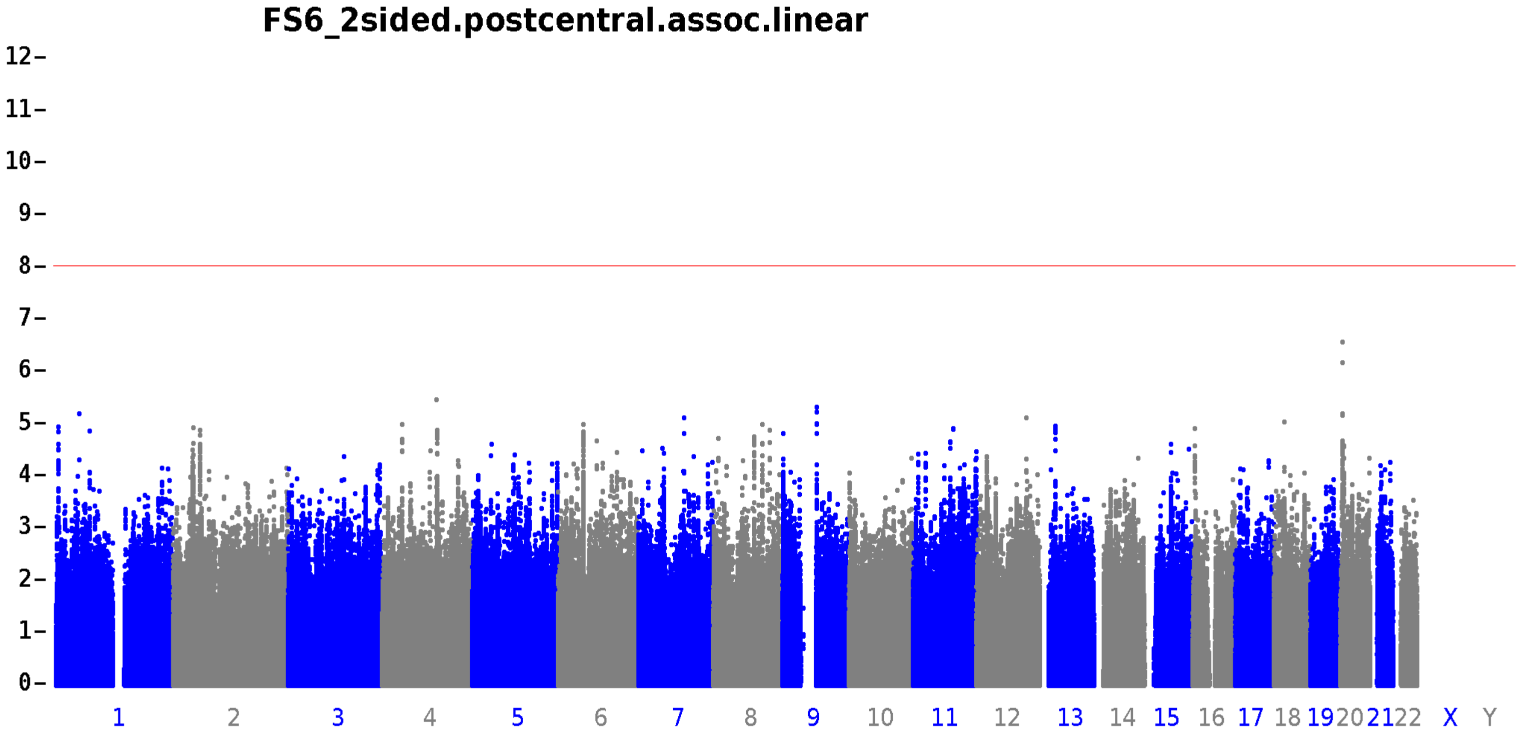

SNP mapped to gene:

CHR	BP	SNP	P	GENE
20	3618313	rs2595588	2.603e-07	ATRN
20	3630681	rs12930	6.459e-07	ATRN
4	115881827	rs1971146	3.23e-06	NDST4
9	72547631	rs11788737	4.525e-06	missing
9	72537085	rs10780702	5.621e-06	missing
9	72537290	rs10780703	5.621e-06	missing
1	49037701	rs1539526	6.01e-06	AGBL4
20	3628312	rs1064833	6.073e-06	ATRN
20	3635182	rs2670307	6.394e-06	missing
12	105782676	rs6539209	7.165e-06	missing
7	96719089	rs62497458	7.386e-06	missing
18	22492582	rs1786152	8.655e-06	missing
9	72563698	rs10868308	9.22e-06	missing
9	72532478	rs12684384	9.563e-06	missing
4	42329287	rs2719930	9.601e-06	missing
6	54966582	rs9357840	9.841e-06	missing
6	54961364	rs3857606	9.841e-06	missing
8	103612553	rs919957	9.843e-06	missing
8	103612849	rs56791675	9.843e-06	missing

Manhattan Plot:

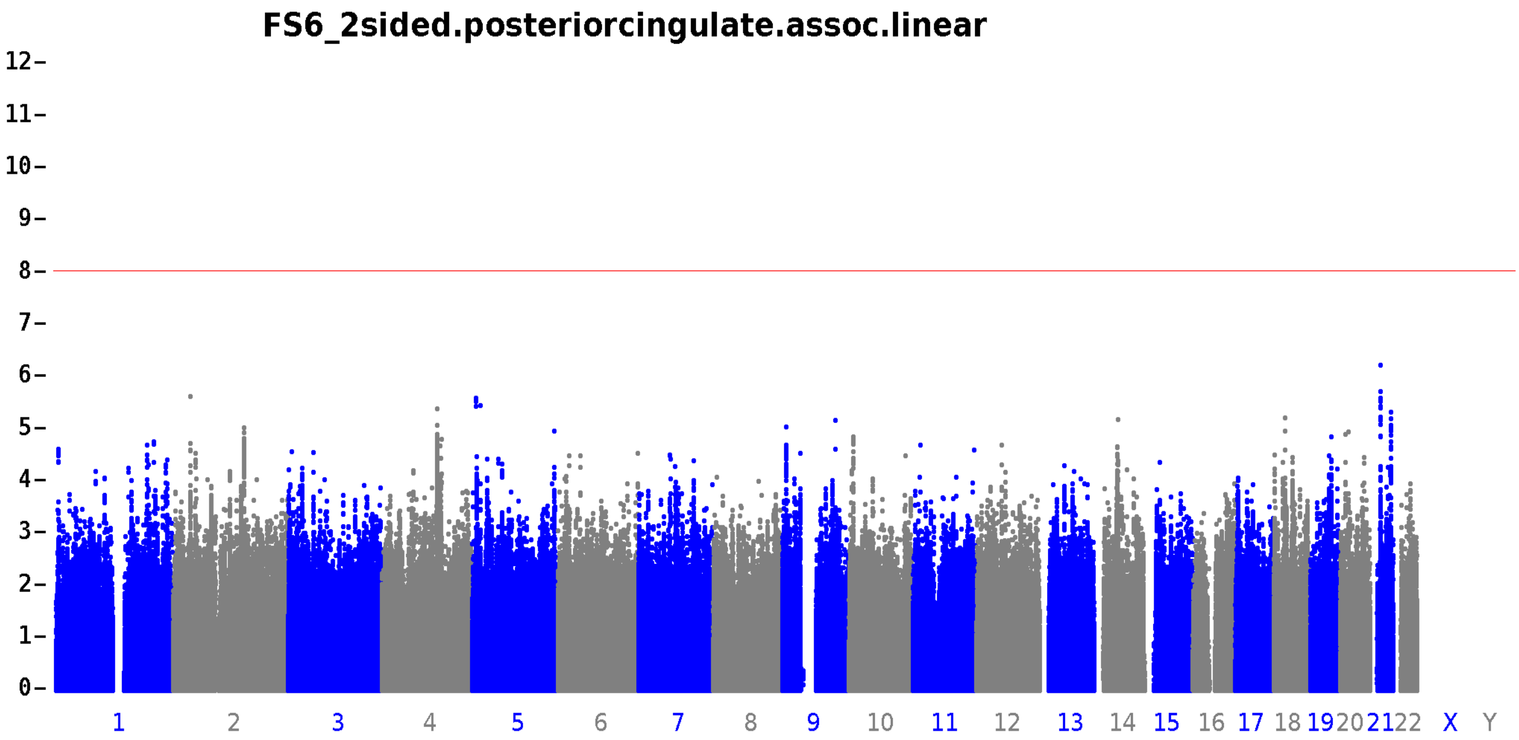

SNP mapped to gene:

CHR	BP	SNP	P	GENE
21	22323268	chr21:22323268:I	5.679e-07	missing
21	22332323	rs139810663	1.817e-06	missing
2	35638202	rs4459713	2.248e-06	missing
5	7528825	rs12654951	2.43e-06	ADCY2
21	22320719	chr21:22320719:D	2.479e-06	missing
21	22335217	rs76652899	2.583e-06	missing
21	22315343	rs13052854	2.769e-06	missing
5	7538237	rs1392481	2.814e-06	ADCY2
5	17784779	rs7708009	3.383e-06	missing
21	22340108	rs77491998	3.469e-06	missing
5	7531623	rs61141857	3.545e-06	ADCY2
21	22339271	chr21:22339271:D	3.83e-06	missing
4	116169689	rs11731910	3.876e-06	missing
21	44001795	rs400625	4.524e-06	missing
21	22330950	rs142230080	5.566e-06	missing
18	24488490	rs163045	5.776e-06	AQP4-AS1
21	44000063	rs380161	6.06e-06	SLC37A1
21	44000078	rs453647	6.06e-06	SLC37A1
14	51313590	rs11627172	6.372e-06	missing
21	22321081	rs9981637	6.554e-06	missing
9	112927881	rs10980231	6.618e-06	PALM2-AKAP2,AKAP2
21	22322923	rs10482914	7.769e-06	missing
21	22323440	rs17794311	7.769e-06	missing
21	44001199	rs389157	8.033e-06	SLC37A1

4	116053779	rs552791	8.19e-06	missing
4	116050207	rs510499	8.19e-06	missing
21	44001218	rs1046783	8.776e-06	SLC37A1
9	7318036	rs73643019	8.857e-06	missing
2	150526826	rs12989809	8.919e-06	LOC101929231
21	43989950	rs2839556	9.489e-06	SLC37A1

Manhattan Plot:

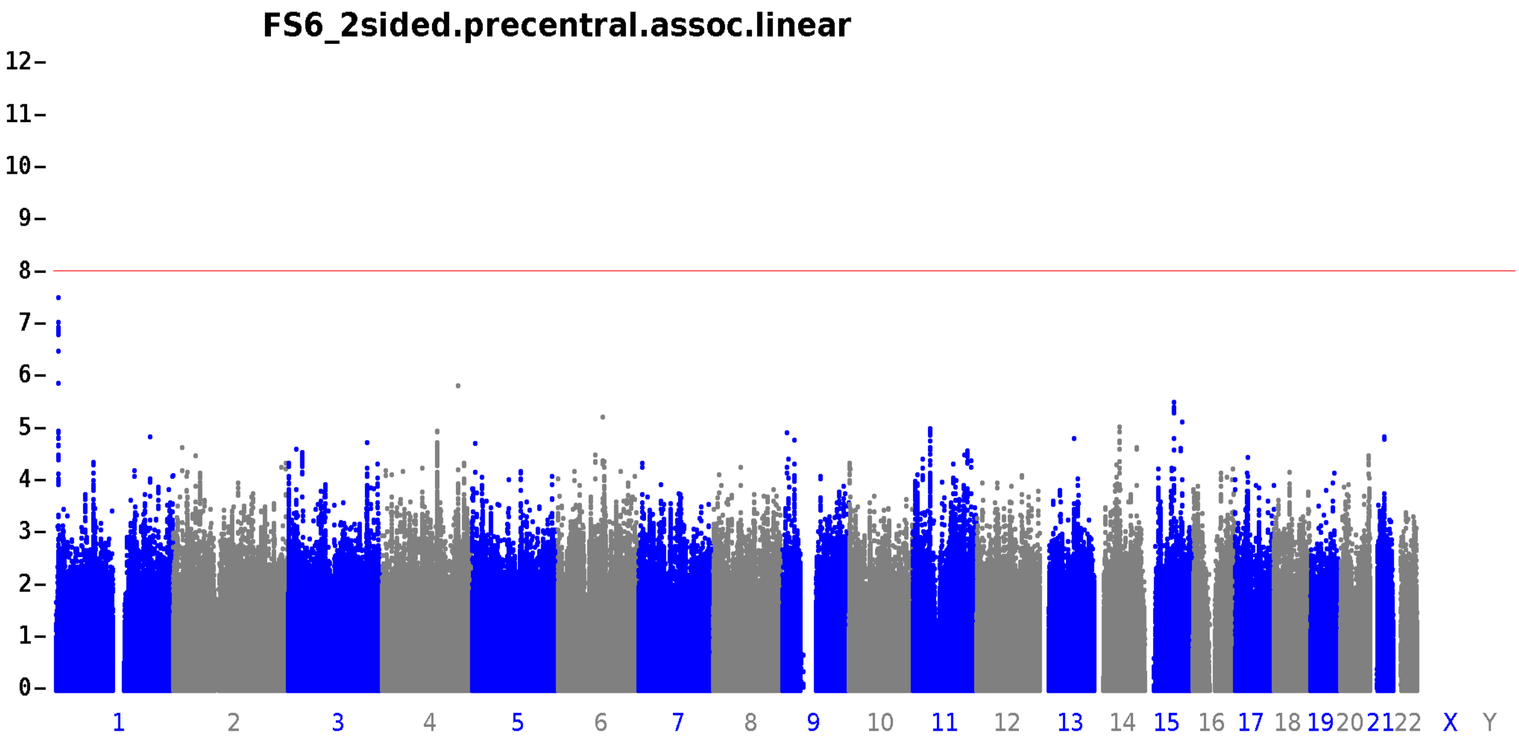

SNP mapped to gene:

CHR	BP	SNP	P	GENE
1	5337271	rs61759358	2.91e-08	missing
1	5341784	rs17423575	8.468e-08	missing
1	5341689	rs57925978	8.468e-08	missing
1	5341289	rs58812660	8.607e-08	missing
1	5341248	rs17457420	8.607e-08	missing
1	5341296	rs61558245	8.607e-08	missing
1	5340869	rs56278800	8.607e-08	missing
1	5341819	chr1:5341819:D	1.052e-07	missing
1	5341464	rs17423547	1.134e-07	missing
1	5339531	rs66495077	1.264e-07	missing
1	5339744	rs56056508	1.482e-07	missing
1	5340385	rs12143953	3.047e-07	missing
1	5338212	rs17457350	1.258e-06	missing
4	161470474	rs9790464	1.389e-06	missing
15	61913054	rs7166435	2.882e-06	missing
15	61914858	rs12915907	3.601e-06	missing
15	61913759	chr15:61913759:I	3.999e-06	missing
15	61915299	rs2414714	4.722e-06	missing
6	95089252	chr6:95089252:I	5.652e-06	missing
15	79719161	rs769761	6.963e-06	missing
14	53914026	rs9323243	8.855e-06	missing
11	37160582	rs1599421	9.335e-06	missing

Manhattan Plot:

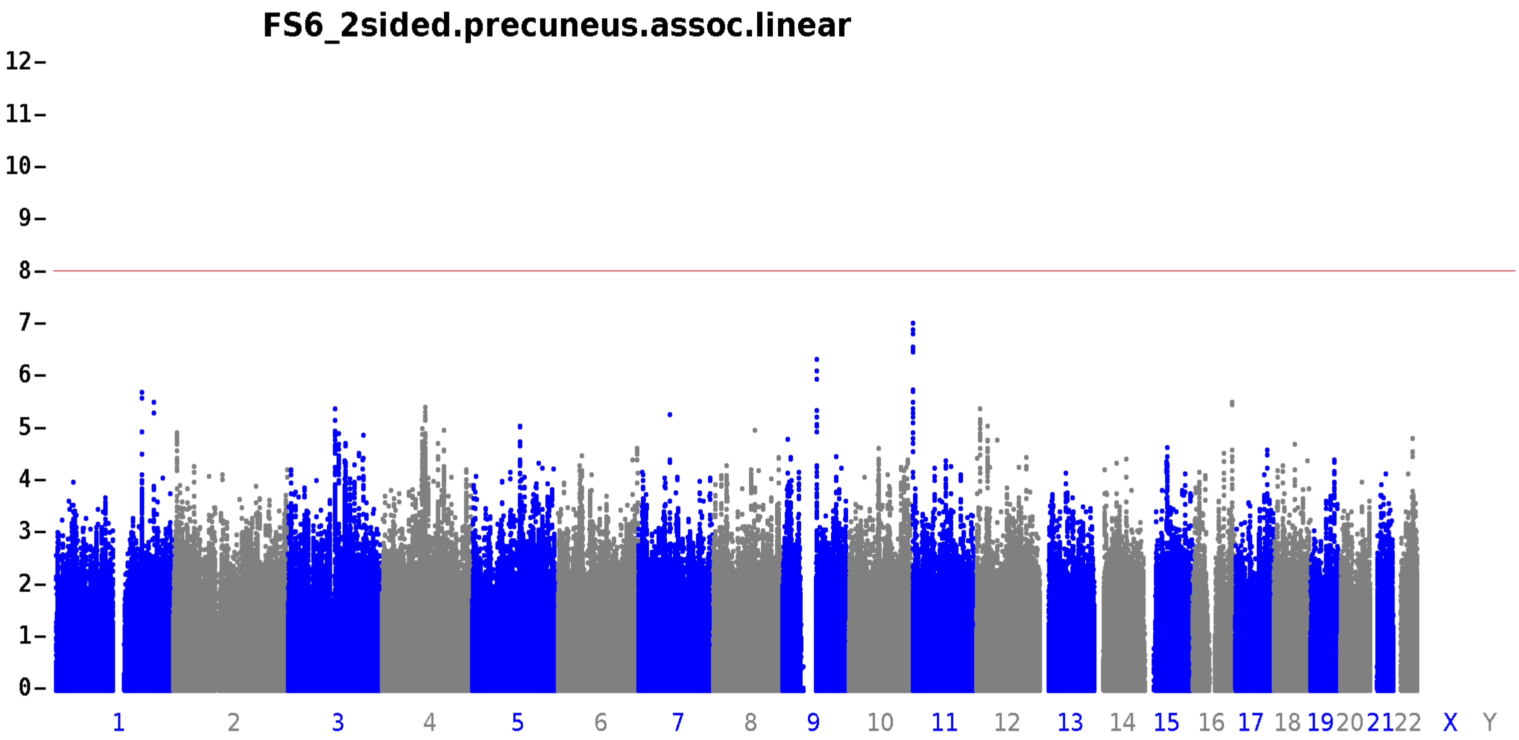

SNP mapped to gene:

CHR	BP	SNP	P	GENE
11	783126	rs78797428	8.793e-08	LOC100506518
11	780689	rs117060799	1.196e-07	LOC100506518,NS3BP
11	782419	rs118129301	1.428e-07	LOC100506518
11	786452	rs117237696	2.563e-07	missing
11	786376	rs76300016	2.856e-07	missing
11	789328	rs77579534	3.135e-07	CEND1
9	72537290	rs10780703	4.407e-07	missing
9	72537085	rs10780702	4.407e-07	missing
9	72532478	rs12684384	7.416e-07	missing
9	72531600	rs10780701	1.065e-06	missing
11	791009	rs118051043	1.695e-06	SLC25A22
11	790418	rs74573437	1.729e-06	missing
11	793502	rs79478359	1.798e-06	SLC25A22
1	182151236	rs1689796	1.857e-06	FLJ46134,LOC100130996
1	182151909	rs1689791	2.469e-06	FLJ46134,LOC100130996
1	207118228	rs4844369	2.878e-06	PIGR
16	83451961	rs9674268	2.891e-06	CDH13
11	817509	rs117857810	2.925e-06	RPLP2
16	83451578	rs9674250	3.306e-06	CDH13
4	90941048	rs1838223	3.587e-06	missing
4	90943750	rs7662081	3.673e-06	missing
12	8047594	rs12423358	3.875e-06	NANOGP1
3	99117309	rs2700603	3.905e-06	missing
11	721877	rs74916186	3.962e-06	EPS8L2

9	72547631	rs11788737	4.15e-06	missing
4	90955516	rs6532211	4.514e-06	missing
4	90989980	rs13119778	4.527e-06	missing
4	90942824	rs6812321	4.573e-06	missing
11	804027	rs77173309	4.622e-06	PIDD
1	207117558	rs1683225	4.705e-06	PIGR
7	67428948	chr7:67428948:D	5.063e-06	missing
4	90957655	rs6842029	5.36e-06	missing
11	793588	rs80335370	5.523e-06	SLC25A22
4	90941051	rs1838222	5.621e-06	missing
4	90940990	rs7667181	5.621e-06	missing
9	72539240	rs10780705	5.66e-06	missing
4	90946548	rs7671006	5.833e-06	missing
12	8050367	rs76442356	6.173e-06	NANOGP1
4	90948523	rs12648916	6.357e-06	missing
4	90941822	rs12641504	6.408e-06	missing
3	99116207	rs1383852	6.598e-06	missing
12	8049599	rs67710609	6.945e-06	NANOGP1
12	8049836	rs145527981	6.945e-06	NANOGP1
11	755659	rs3895063	7.263e-06	TALDO1
12	8050120	rs66776854	7.568e-06	NANOGP1
9	72539262	rs10746756	7.831e-06	missing
9	72540327	rs10868271	8.259e-06	missing
12	22793049	rs73080476	8.297e-06	ETNK1
5	101441031	rs73177261	8.503e-06	missing
5	101453333	rs73177270	8.807e-06	missing
4	85453347	chr4:85453347:D	9.192e-06	missing
12	8046979	rs7967681	9.341e-06	NANOGP1

Manhattan Plot:

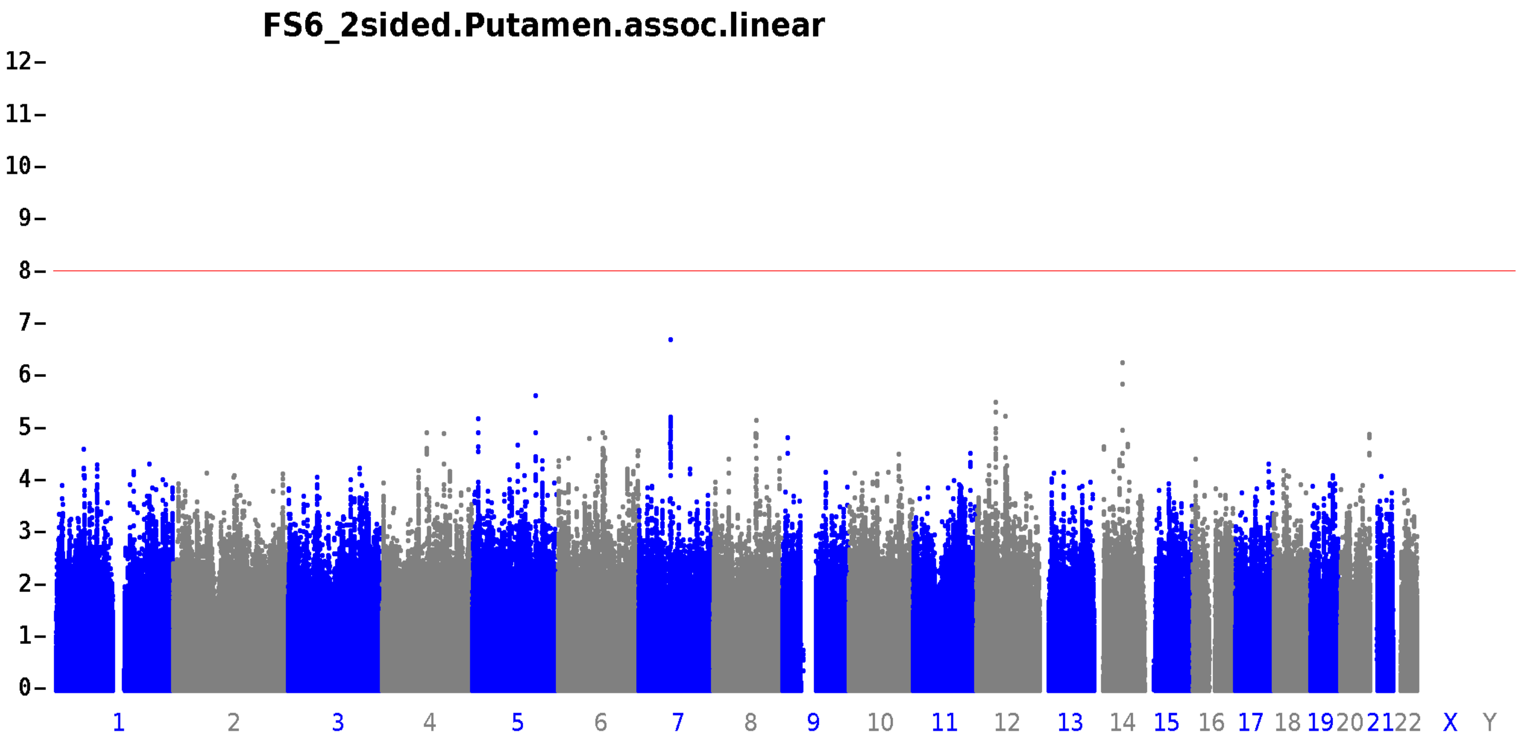

SNP mapped to gene:

CHR	BP	SNP	P	GENE
7	68540921	rs28435928	1.819e-07	missing
14	60112115	rs2349697	5.122e-07	RTN1
14	60112112	rs2349696	1.303e-06	RTN1
5	133777572	rs328836	2.214e-06	missing
12	40341502	rs28370599	2.884e-06	SLC2A13
12	40339112	rs35985096	4.53e-06	SLC2A13
12	61703936	rs12816622	5.338e-06	missing
7	68494409	rs73411304	5.527e-06	missing
7	68496680	rs73411314	5.589e-06	missing
7	68494987	rs73411307	5.589e-06	missing
7	68495130	rs78756159	5.589e-06	missing
5	12102037	rs4601043	5.94e-06	missing
7	68489391	rs7789368	6.399e-06	missing
8	90350244	rs1947835	6.475e-06	missing
7	68487034	rs112856102	7.253e-06	missing
7	68490692	rs28394379	7.351e-06	missing
7	68538411	rs12112294	8.169e-06	missing
7	68526836	rs923136	8.638e-06	missing
7	68526961	rs923134	8.638e-06	missing
7	68535886	rs10252979	8.638e-06	missing
7	68526742	chr7:68526742:I	8.638e-06	missing
7	68525689	rs4541798	8.638e-06	missing
12	40337799	chr12:40337799:D	9.392e-06	SLC2A13

Manhattan Plot:

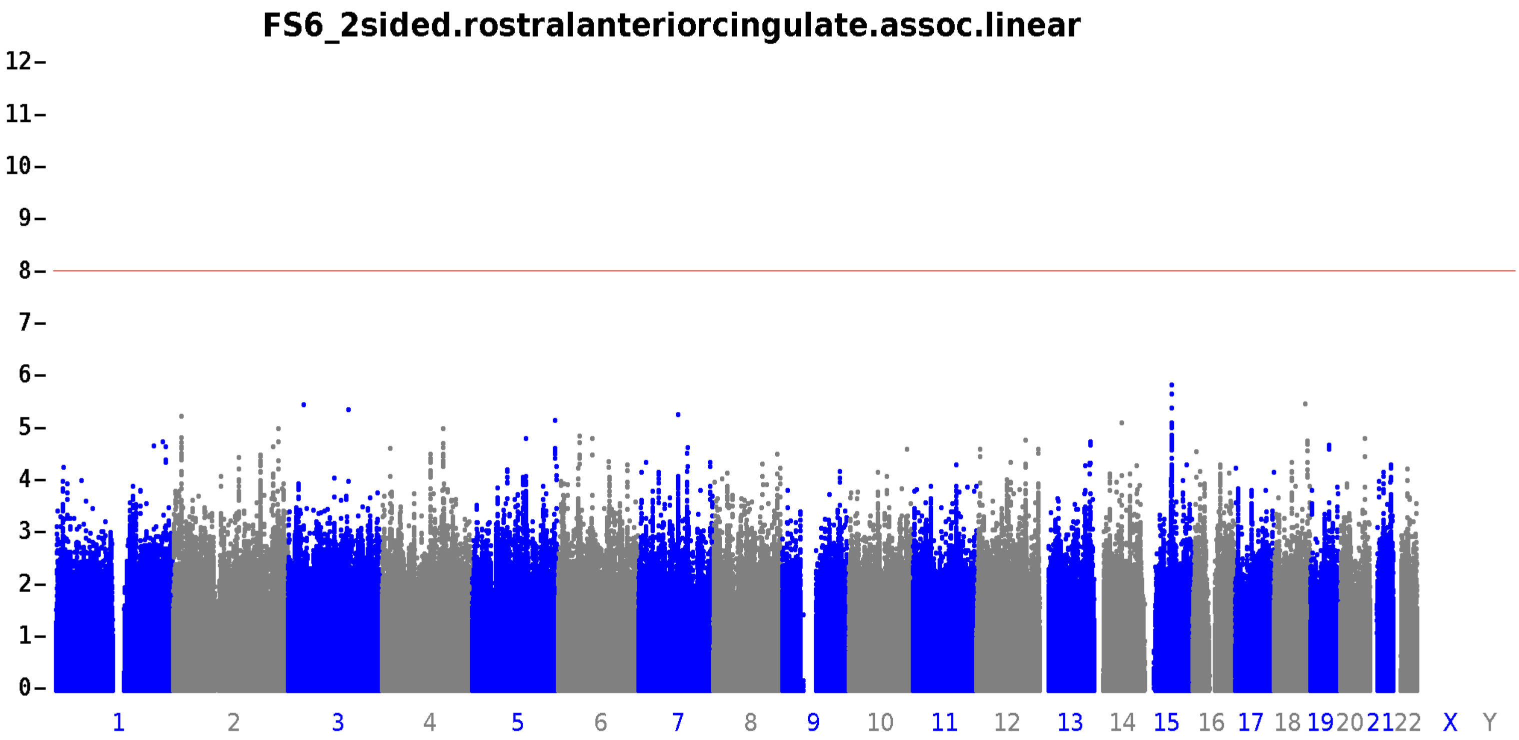

SNP mapped to gene:

CHR	BP	SNP	P	GENE
15	57876788	rs1908195	1.387e-06	missing
15	57876962	rs1908196	2.001e-06	missing
18	66575528	rs73462092	3.155e-06	CCDC102B
3	33424753	rs13078580	3.264e-06	FBXL2
15	57882297	rs1908202	3.798e-06	missing
3	128665169	chr3:128665169:D	4.033e-06	missing
7	84506283	chr7:84506283:I	5.079e-06	HMGN2P11
2	16540639	rs1429403	5.346e-06	missing
2	16540055	rs74173263	5.346e-06	missing
5	175028900	rs2001690	6.613e-06	missing
15	57884219	rs2453089	7.221e-06	MYZAP,GCOM1
14	59471071	rs68136176	7.238e-06	missing
15	57876086	rs2451187	8.292e-06	missing
15	57878365	rs1908197	9.098e-06	missing
2	222314415	rs60138526	9.377e-06	EPHA4
4	130177345	rs113000049	9.469e-06	missing
4	130176011	rs7659117	9.469e-06	missing

Manhattan Plot:

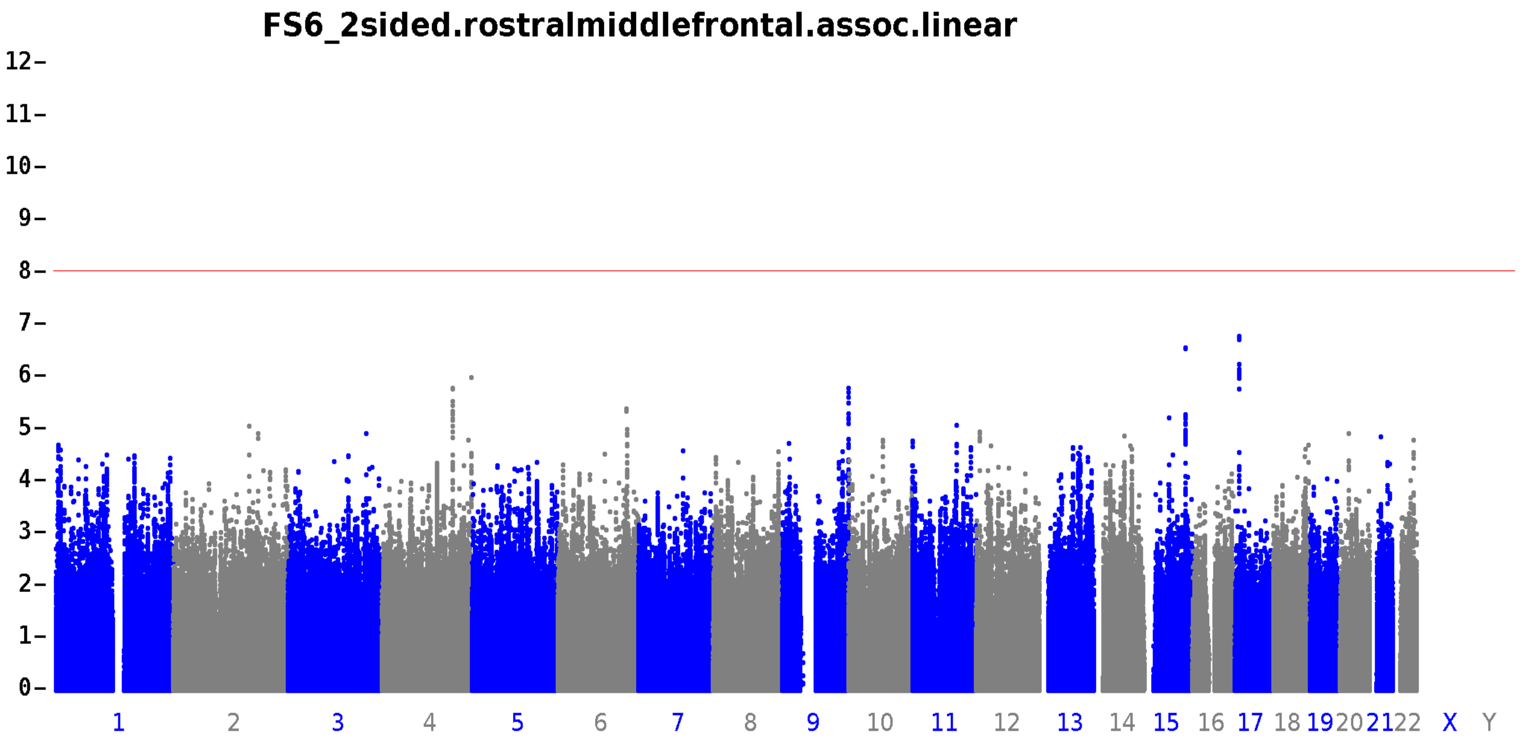

SNP mapped to gene:

CHR	BP	SNP	P	GENE
17	8968500	rs9910696	1.584e-07	NTN1
17	8970198	rs72660225	1.869e-07	NTN1
15	87193018	rs2469185	2.632e-07	AGBL1
15	87215267	rs8036577	2.716e-07	AGBL1
17	8967380	rs7222847	5.525e-07	NTN1
17	8969667	rs6503176	6.729e-07	NTN1
17	8968276	rs8065801	7.382e-07	NTN1
17	8968695	rs9905180	8.052e-07	NTN1
17	8967920	rs8078106	8.052e-07	NTN1
17	8969573	chr17:8969573:I	9.218e-07	NTN1
17	8969672	rs8066040	9.218e-07	NTN1
17	8969817	rs7215014	9.218e-07	NTN1
4	189811357	rs7674523	9.76e-07	missing
17	8968513	rs9910706	1.031e-06	NTN1
4	149989412	rs11730050	1.589e-06	missing
9	140494249	rs9777268	1.604e-06	missing
4	149986818	rs9985850	1.618e-06	missing
17	8973969	rs6503177	1.62e-06	NTN1
9	140493271	rs7020339	1.876e-06	missing
9	140518784	rs2501553	2.329e-06	EHMT1
4	149955408	rs4259039	2.827e-06	missing
9	140497072	rs1776776	3.037e-06	missing
4	149949326	rs6851434	3.362e-06	missing
6	146939392	rs9403796	3.97e-06	ADGB

4	149956375	rs6842098	4.327e-06	missing
6	146938317	rs6570770	4.366e-06	ADGB
4	149950136	chr4:149950136:D	4.506e-06	missing
4	149987640	rs4302443	4.646e-06	missing
4	149979341	rs7673586	4.674e-06	missing
4	149979309	rs7668770	4.674e-06	missing
9	140506058	rs9410111	4.913e-06	ARRDC1
9	140505790	rs35374891	4.913e-06	ARRDC1
15	87213424	rs6496351	4.965e-06	AGBL1
4	149955558	rs4635800	5.022e-06	missing
15	87217613	rs8028043	5.146e-06	AGBL1
15	87211579	rs12898875	5.619e-06	AGBL1
9	140497212	rs11137148	5.772e-06	missing
15	52371745	rs72732980	5.93e-06	missing
4	149982625	rs10213414	6.015e-06	missing
15	87212854	rs4322631	6.019e-06	AGBL1
9	140526439	rs2987621	6.35e-06	EHMT1
4	149967290	rs13124217	6.351e-06	missing
4	149960317	rs4640651	6.351e-06	missing
4	149973339	rs12645768	6.439e-06	missing
4	149970995	rs4371599	6.503e-06	missing
15	87179871	rs2447282	7.158e-06	AGBL1
15	87221126	rs6496352	7.5e-06	AGBL1
9	140506209	rs821313	7.599e-06	ARRDC1
15	87192942	chr15:87192942:D	7.681e-06	AGBL1
11	93162908	rs11820548	8.112e-06	CCDC67
2	160950112	rs13432522	8.368e-06	missing
4	149984123	rs13435788	8.371e-06	missing
6	146957941	rs957547	9.798e-06	ADGB

Manhattan Plot:

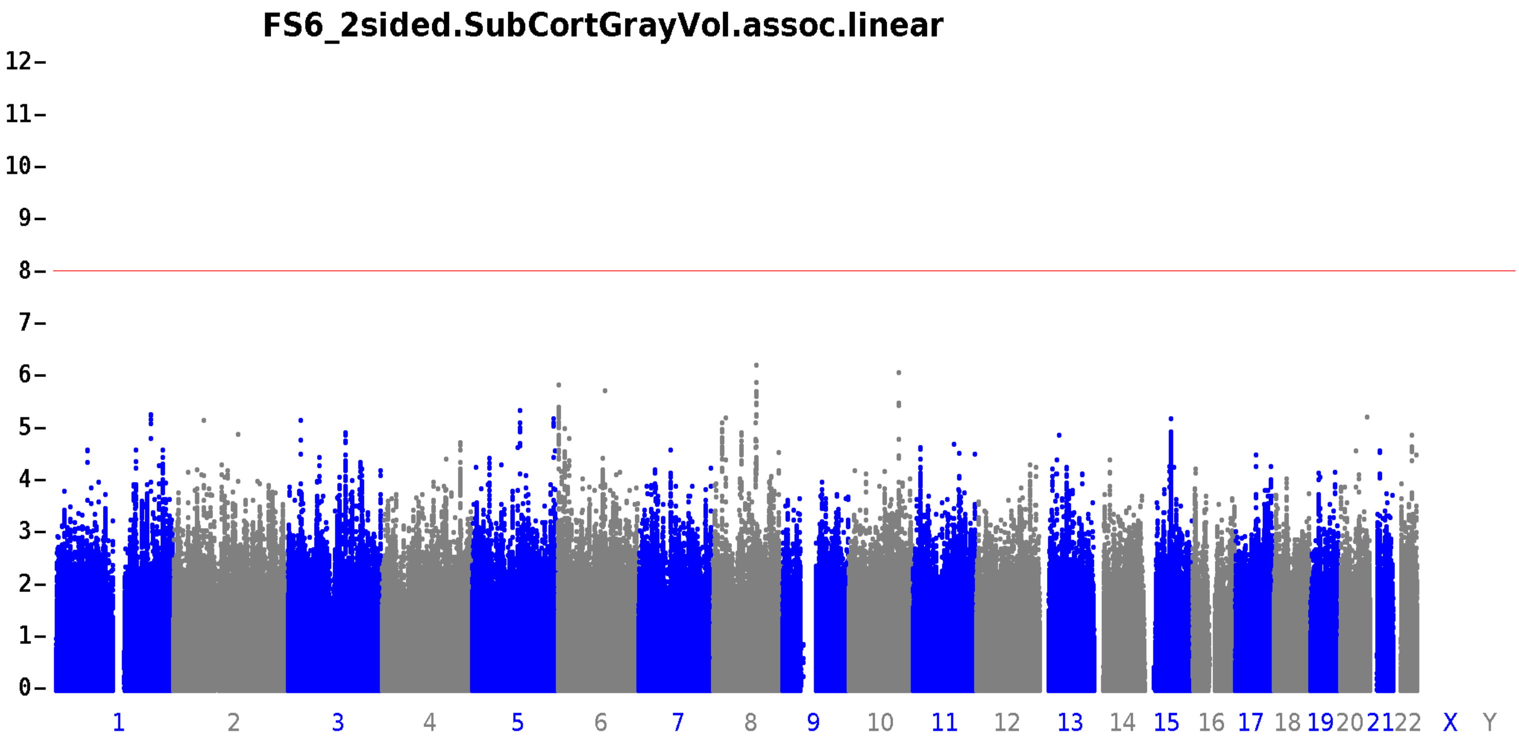

SNP mapped to gene:

CHR	BP	SNP	P	GENE
8	90350244	rs1947835	5.603e-07	missing
10	105664459	rs2475213	7.821e-07	OBFC1
8	90361235	rs4263817	1.216e-06	missing
8	90343201	rs899638	1.238e-06	missing
6	1722293	rs4959598	1.373e-06	GMDS
6	100957344	rs240768	1.769e-06	ASCC3
8	90364065	rs1904521	1.814e-06	missing
8	90363204	rs7018458	1.905e-06	missing
8	90363808	rs6981493	1.905e-06	missing
8	90346979	rs12549775	1.988e-06	missing
8	90363889	chr8:90363889:I	2.299e-06	missing
10	105666745	rs9420904	3.066e-06	OBFC1
8	90363990	rs6985497	3.08e-06	missing
8	90341715	rs1479905	3.211e-06	missing
10	105660651	rs2475216	3.325e-06	OBFC1
6	1725885	rs9378309	3.618e-06	GMDS
6	1725261	rs7754428	4.011e-06	GMDS
6	1725288	rs9378308	4.011e-06	GMDS
6	1725491	rs9405148	4.011e-06	GMDS
6	1725301	rs7754564	4.011e-06	GMDS
5	101395391	rs1477616	4.252e-06	missing
5	101394874	rs11748160	4.252e-06	missing
5	101394058	rs28530672	4.252e-06	missing
5	101393615	rs56256687	4.252e-06	missing

6	1732441	rs1986205	4.57e-06	GMDS
6	1724081	rs2318107	4.776e-06	GMDS
6	1723961	rs9392327	4.776e-06	GMDS
6	1724111	rs764518	4.776e-06	GMDS
1	201154032	rs78196549	5.061e-06	missing
8	90362569	rs10098050	5.105e-06	missing
6	1731692	rs1013064	5.11e-06	GMDS
1	201152188	rs12119866	5.286e-06	missing
8	90358646	rs1842299	5.464e-06	missing
20	56053766	rs11700223	5.571e-06	missing
8	26857693	rs7842266	5.825e-06	missing
6	1725792	rs9392328	5.927e-06	GMDS
5	172723981	rs13436374	6.135e-06	missing
15	56517816	rs11636035	6.152e-06	RFX7
1	201150162	rs12117787	6.295e-06	missing
3	26397294	rs4973780	6.389e-06	missing
2	64563171	rs1426703	6.407e-06	missing
6	1729442	rs6937688	7.106e-06	GMDS
6	1729986	rs9328068	7.106e-06	GMDS
6	1729475	rs6942353	7.106e-06	GMDS
8	90300401	rs13274960	7.209e-06	missing
8	18856363	rs1365600	7.222e-06	PSD3
5	172723961	chr5:172723961:I	7.271e-06	missing
5	101396165	rs28757584	7.374e-06	missing
6	1723854	rs9405507	7.468e-06	GMDS
6	1723356	rs9378652	7.468e-06	GMDS
1	201140614	rs78455761	7.498e-06	TMEM9
1	201140516	rs79721915	7.498e-06	TMEM9
5	172723955	rs13436373	7.628e-06	missing
5	172723954	chr5:172723954:D	7.628e-06	missing
6	1731609	rs7754761	8.1e-06	GMDS
5	172723961	rs13436370	8.262e-06	missing
6	1722203	rs4959150	8.876e-06	GMDS
5	101395497	rs1477615	9.148e-06	missing
6	15234787	rs112575696	9.376e-06	missing
8	18859248	rs12677249	9.725e-06	PSD3

Manhattan Plot:

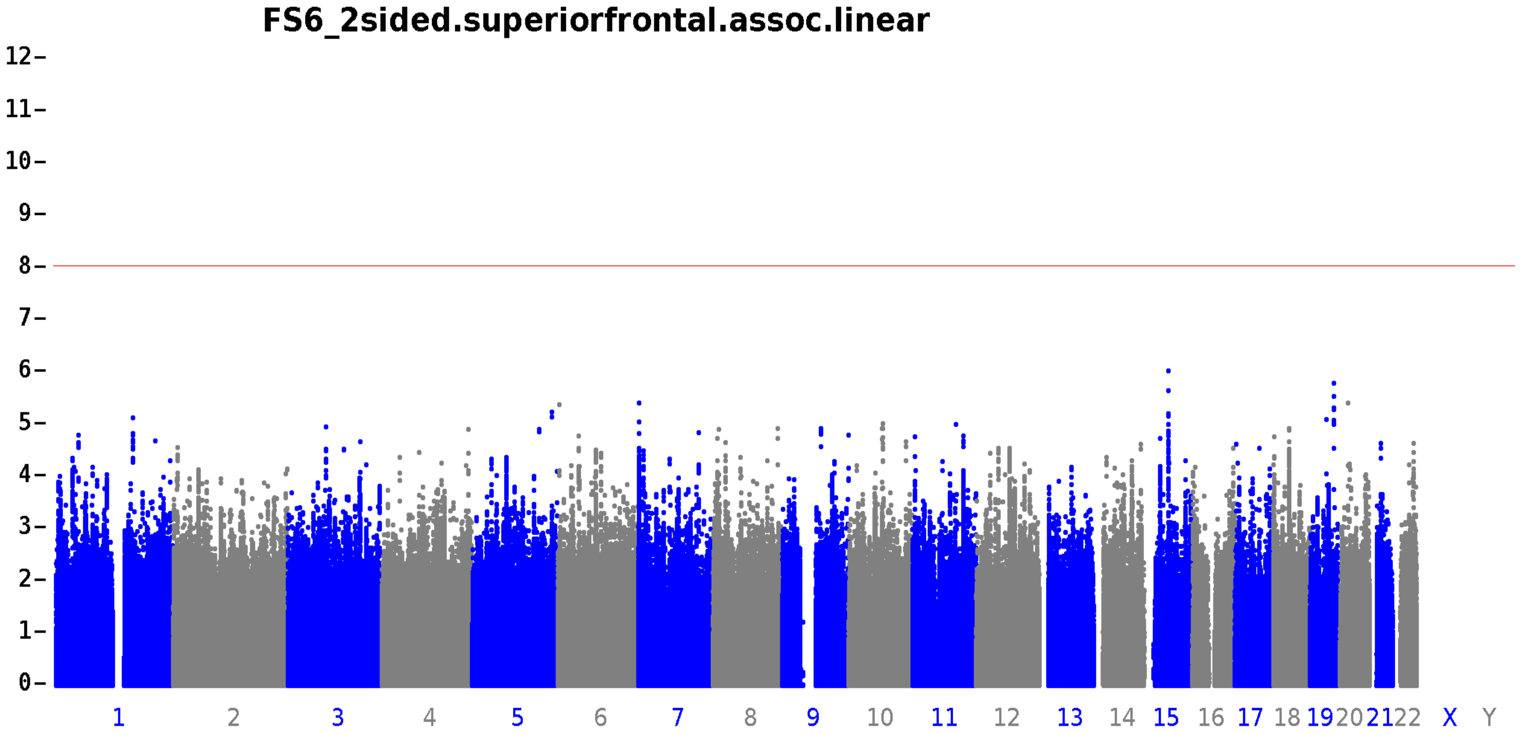

SNP mapped to gene:

CHR	BP	SNP	P	GENE
15	50636018	rs11630063	9.241e-07	GABPB1
19	51136844	rs8104662	1.582e-06	SYT3
15	50645617	rs526952	2.19e-06	GABPB1,FLJ10038
19	51109930	rs1895374	2.859e-06	missing
7	2036460	rs59574136	3.734e-06	MAD1L1
20	16650695	rs6044208	3.79e-06	missing
6	4186722	rs664369	4.081e-06	missing
19	51111061	rs7258762	4.713e-06	missing
19	51137292	rs7359910	5.134e-06	SYT3
5	169538008	rs7724629	5.675e-06	missing
15	50604559	rs4775873	5.997e-06	GABPB1
15	50602837	rs11637975	5.997e-06	GABPB1
15	50596070	rs7164798	5.997e-06	GABPB1
15	50609591	rs34312018	6.099e-06	GABPB1
15	50579197	rs28453939	6.236e-06	GABPB1
15	50628715	rs35013067	6.799e-06	GABPB1
5	169529903	rs17738959	7.027e-06	missing
1	164237192	rs7512727	7.202e-06	missing
1	164236390	rs79958639	7.202e-06	missing
19	35472213	rs295769	7.699e-06	missing
19	51109793	rs1895373	8.027e-06	missing
7	2029867	rs58120505	8.582e-06	MAD1L1
10	72269484	rs4746045	9.219e-06	PALD1
19	51134973	rs7258140	9.362e-06	SYT3

15	50608855	rs11631197	9.712e-06	GABPB1
15	50618102	rs7183422	9.712e-06	GABPB1
15	50602067	rs35446819	9.712e-06	GABPB1
11	92132457	rs659041	9.764e-06	FAT3
19	51138089	rs6509493	9.843e-06	SYT3

Manhattan Plot:

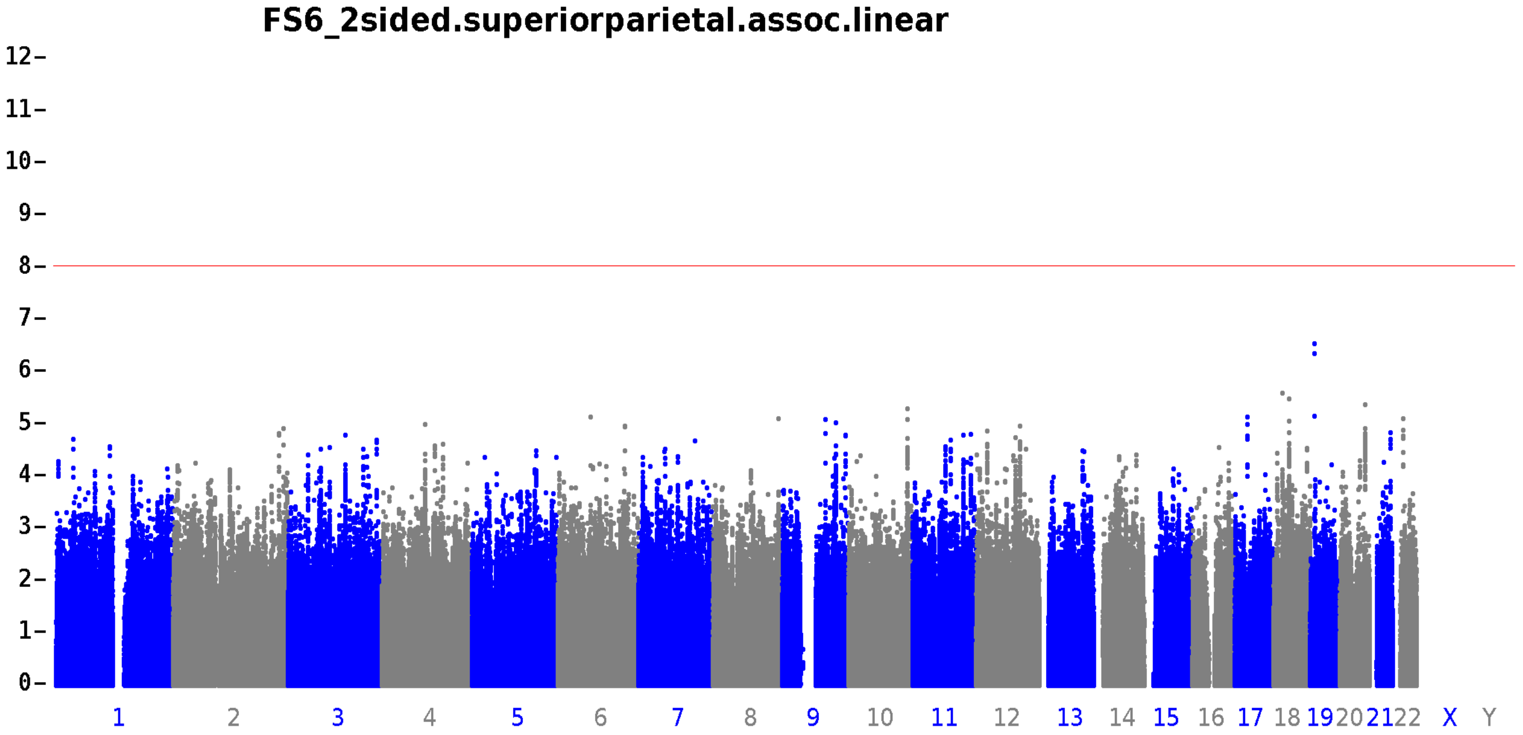

SNP mapped to gene:

CHR	BP	SNP	P	GENE
19	10178233	rs11880218	2.791e-07	C3P1
19	10176663	rs4804480	4.266e-07	C3P1
18	20188356	rs4530258	2.443e-06	missing
18	33169134	rs72958837	3.182e-06	missing
20	52429950	rs6022773	4.059e-06	missing
20	52429826	rs6022772	4.063e-06	missing
10	124086757	rs6585812	4.844e-06	BTBD16
19	10181347	rs12610576	6.82e-06	C3P1
6	69551838	rs1589733	6.932e-06	BAI3
17	25563430	rs62057205	7.05e-06	missing
8	138060119	rs67017865	7.511e-06	missing
22	22118857	rs59312009	7.557e-06	MAPK1
10	124081852	rs9633735	7.774e-06	BTBD16
9	91677507	rs17054710	7.839e-06	SHC3
18	33284519	rs7230728	8.321e-06	GALNT1
9	114647373	rs76769539	9.145e-06	missing
9	114646524	rs72756216	9.145e-06	missing
9	114645249	rs58326846	9.145e-06	missing
17	25557996	rs16965175	9.76e-06	missing
17	25557550	rs16965173	9.76e-06	missing
4	90989980	rs13119778	9.823e-06	missing

Manhattan Plot:

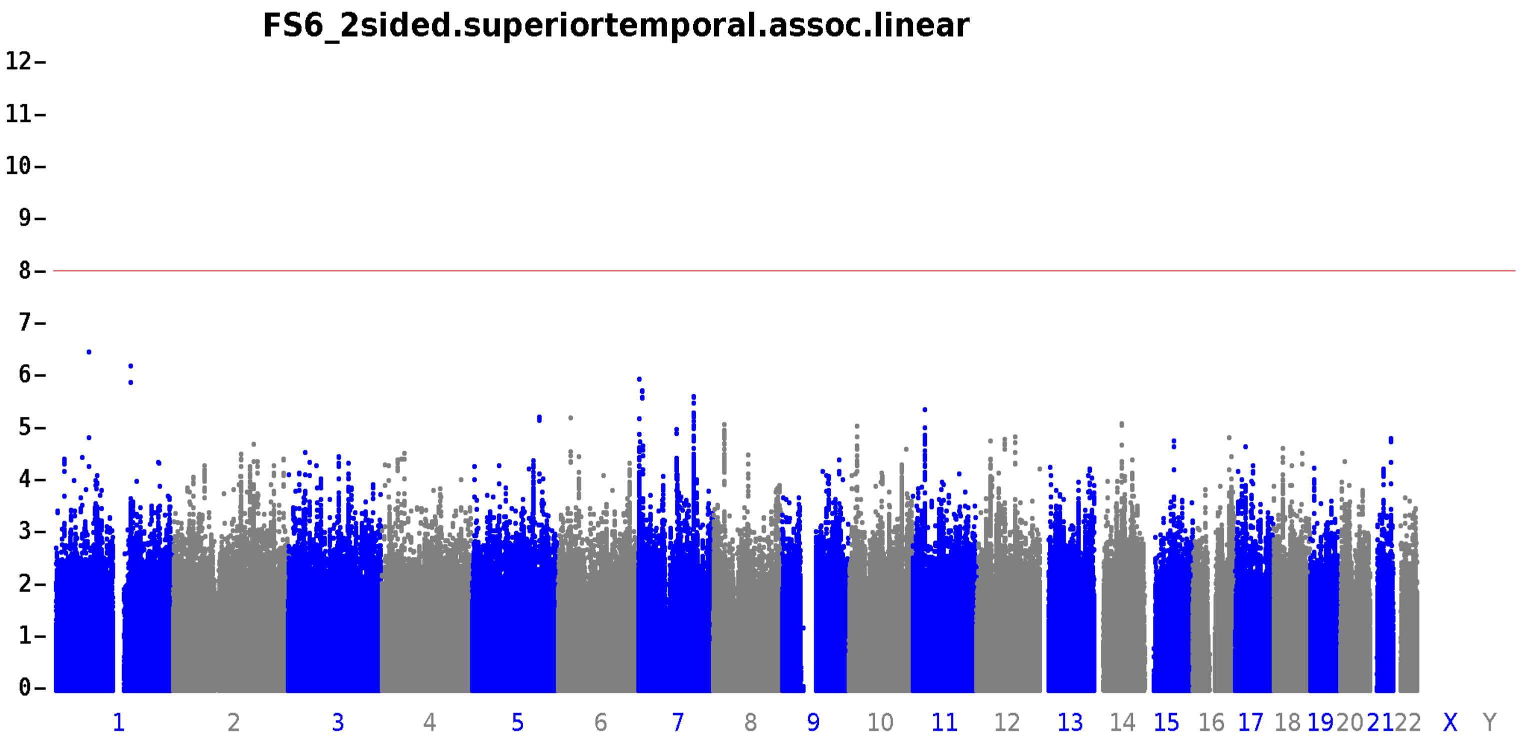

SNP mapped to gene:

CHR	BP	SNP	P	GENE
1	71088780	rs17131365	3.232e-07	missing
1	158493055	rs11265005	5.911e-07	missing
7	2140312	rs4719431	1.051e-06	MAD1L1
1	158475894	rs12041363	1.218e-06	missing
7	8795456	rs10239719	1.764e-06	missing
7	8794010	rs10238890	1.832e-06	missing
7	117779712	rs112560678	2.26e-06	missing
7	117857351	rs6953131	2.316e-06	missing
7	117882854	rs62468166	2.316e-06	missing
7	8796378	rs10486254	2.385e-06	missing
7	8797698	rs10248396	2.417e-06	missing
7	117813571	rs74302507	3.028e-06	missing
11	24873665	chr11:24873665:D	4.016e-06	LUZP2
7	117810969	rs4141030	4.728e-06	missing
7	117816390	rs62468315	5.518e-06	missing
5	141369465	rs252090	5.619e-06	RNF14
7	117822747	rs6945543	5.649e-06	missing
6	27614951	chr6:27614951:I	5.859e-06	missing
7	2036460	rs59574136	6.146e-06	MAD1L1
5	141364752	rs252101	6.595e-06	RNF14
7	117818015	rs62466462	6.722e-06	missing
7	117818264	rs6957412	6.722e-06	missing
7	117818210	rs6957623	6.722e-06	missing
7	117817497	rs62466461	6.722e-06	missing

14	59504086	rs7149404	7.421e-06	missing
8	22538879	rs4872007	7.757e-06	missing
14	59504847	rs12385904	7.98e-06	missing
7	117802844	rs62468300	8.218e-06	missing
7	117801733	rs62468299	8.218e-06	missing
7	117802944	rs62468301	8.218e-06	missing
7	117801556	rs62468298	8.218e-06	missing
7	117803805	rs62468302	8.218e-06	missing
7	117800213	rs62468294	8.218e-06	missing
7	117796109	rs77955826	8.218e-06	missing
10	17011716	rs78858833	8.256e-06	CUBN
7	117810171	chr7:117810171:D	8.364e-06	missing
7	117811358	rs62468308	8.364e-06	missing
7	117810876	rs4141029	8.364e-06	missing
7	117811487	rs62468309	8.364e-06	missing
7	117810384	rs62468306	8.364e-06	missing
7	117814508	chr7:117814508:D	8.364e-06	missing
7	117809603	rs62468305	8.364e-06	missing
7	117807420	rs78572985	8.364e-06	missing
7	117807002	rs78160092	8.364e-06	missing
7	117815304	rs62468314	8.364e-06	missing
7	117806290	rs62468303	8.364e-06	missing
7	117799882	chr7:117799882:I	8.56e-06	missing
11	24879621	chr11:24879621:I	9.022e-06	LUZP2
7	117815099	rs62468312	9.349e-06	missing
7	117815116	rs62468313	9.349e-06	missing
7	117918040	rs74954828	9.421e-06	missing
7	81363334	rs11975846	9.698e-06	HGF

Manhattan Plot:

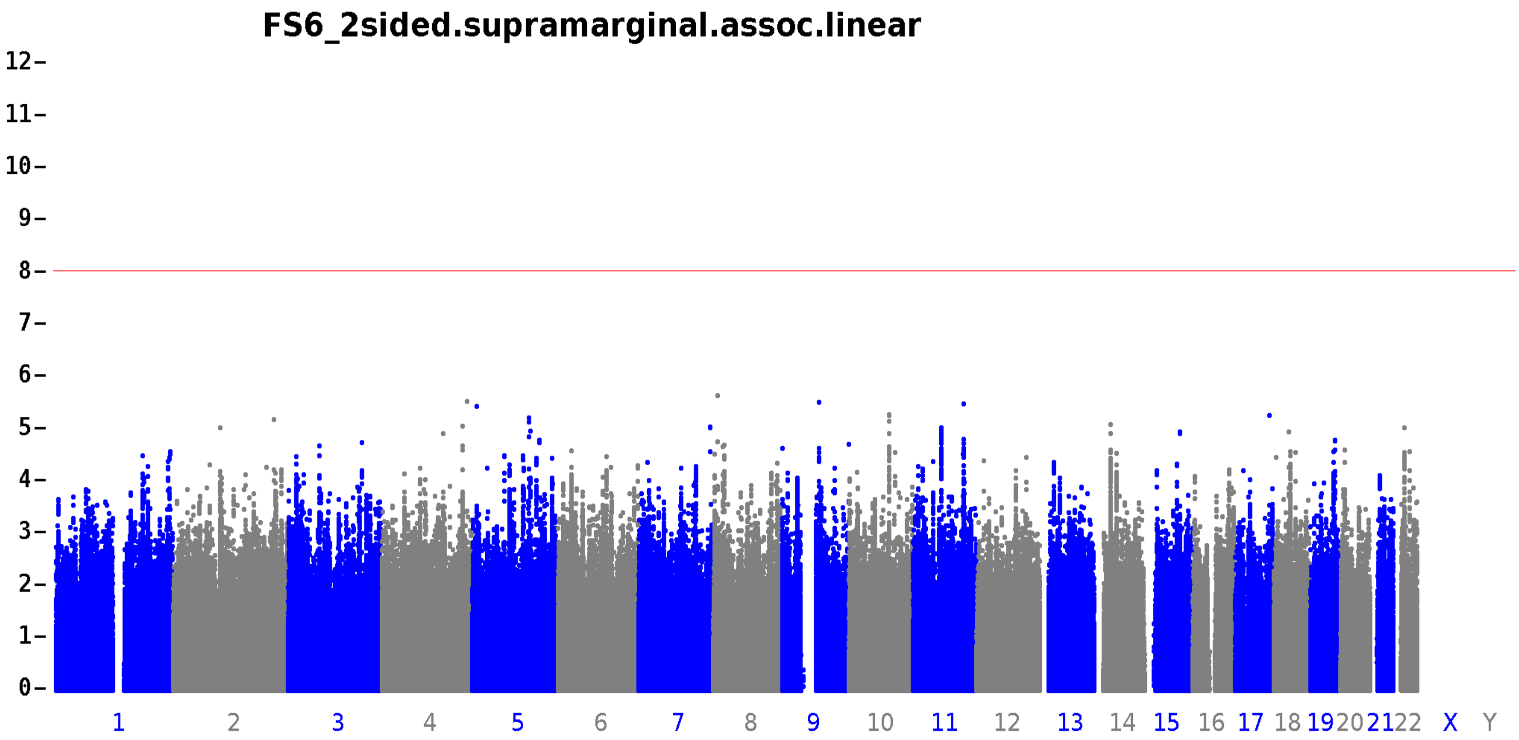

SNP mapped to gene:

CHR	BP	SNP	P	GENE
8	8573004	rs544377	2.179e-06	missing
4	179676626	rs17066724	2.87e-06	missing
9	76841028	rs76083613	2.923e-06	LOC101927329
11	107188149	rs10890710	3.155e-06	missing
5	8647119	rs17221177	3.512e-06	missing
10	85522191	rs66834924	5.06e-06	missing
17	72031724	rs2198989	5.204e-06	missing
10	85519215	rs11200446	5.251e-06	missing
5	120649567	rs62379951	5.798e-06	missing
2	213602048	rs6725004	6.293e-06	missing
10	85519116	rs11200444	6.858e-06	missing
5	120649523	rs62379950	7.101e-06	missing
14	35605876	rs4981267	7.779e-06	KIAA0391
4	170374260	rs138504420	8.373e-06	NEK1
7	151602696	rs12703179	8.812e-06	missing
11	60266798	rs4939378	8.875e-06	MS4A12
2	99500630	rs1581249	8.894e-06	KIAA1211L
7	151606466	rs17490405	8.999e-06	missing
22	24886387	rs4822504	9.079e-06	ADORA2A-AS1,UPB1

Manhattan Plot:

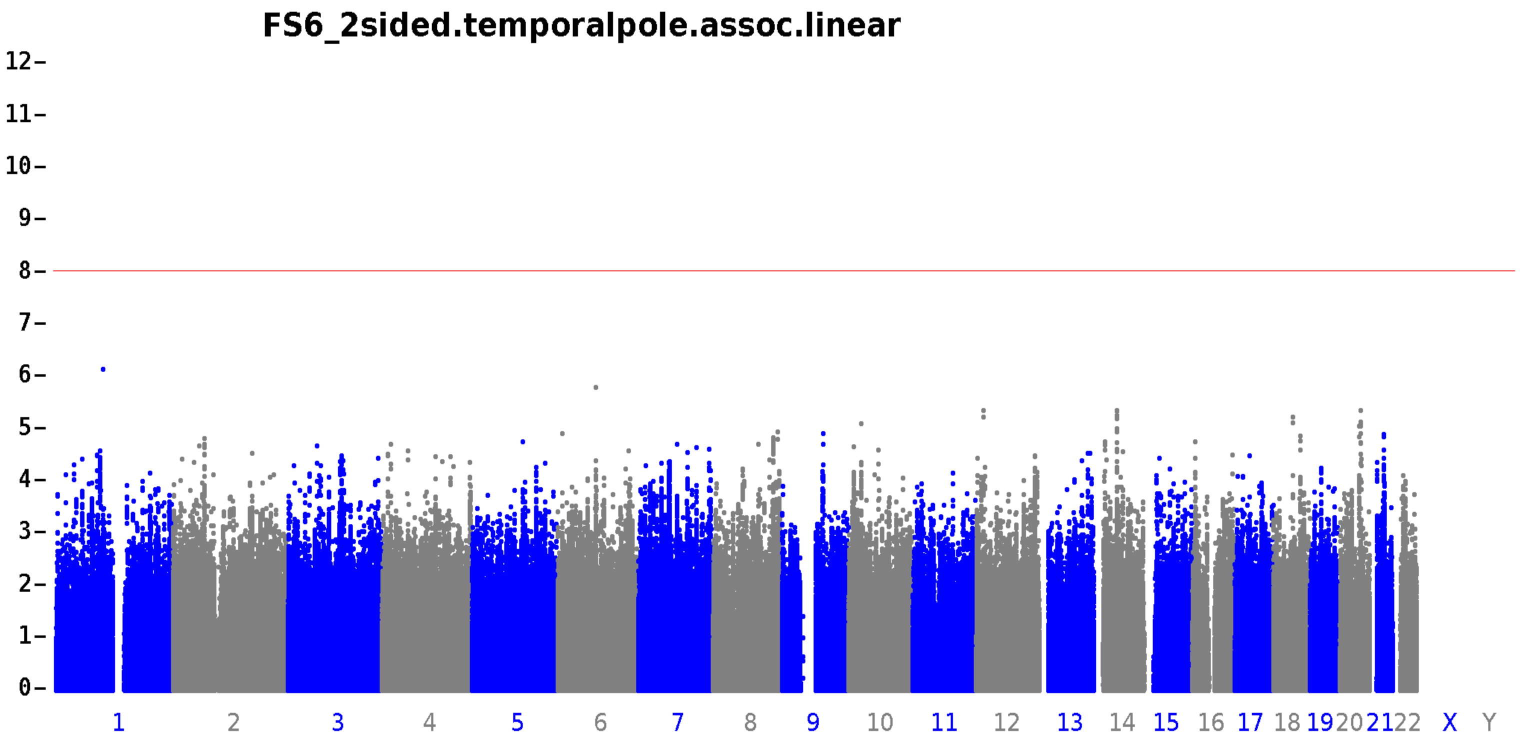

SNP mapped to gene:

CHR	BP	SNP	P	GENE
1	100638801	rs7528137	6.899e-07	LRRC39
6	81671407	rs73750943	1.529e-06	missing
12	15579166	rs7972505	4.213e-06	PTPRO
14	48963630	rs1385594	4.218e-06	missing
20	44429383	rs186635489	4.257e-06	DNTTIP1
14	48964310	rs4898599	4.315e-06	missing
14	48975890	rs77755473	5.155e-06	missing
18	41486749	rs12326432	5.699e-06	missing
12	15574082	rs11056471	5.726e-06	PTPRO
14	48962101	rs7150842	5.767e-06	missing
14	48967924	rs7140187	6.099e-06	missing
20	44399570	rs73122716	6.986e-06	missing
20	44407391	rs73122729	6.986e-06	WFDC3
20	44405060	rs111504208	6.986e-06	WFDC3
20	44407431	rs73122731	6.986e-06	WFDC3
20	44399538	rs73120855	6.986e-06	missing
20	44425747	rs73122768	6.986e-06	DNTTIP1
18	41511671	rs11876994	7.361e-06	missing
20	44391255	rs112356554	7.426e-06	missing
20	44393915	rs74573863	7.426e-06	missing
10	25936301	rs11014652	7.515e-06	GPN3P1
10	25926872	rs11014648	7.515e-06	missing
20	44410877	rs73122735	8.324e-06	WFDC3
20	41194912	chr20:41194912:I	8.453e-06	PTPRT

14	48974473	rs9788475	9.524e-06	missing

Manhattan Plot:

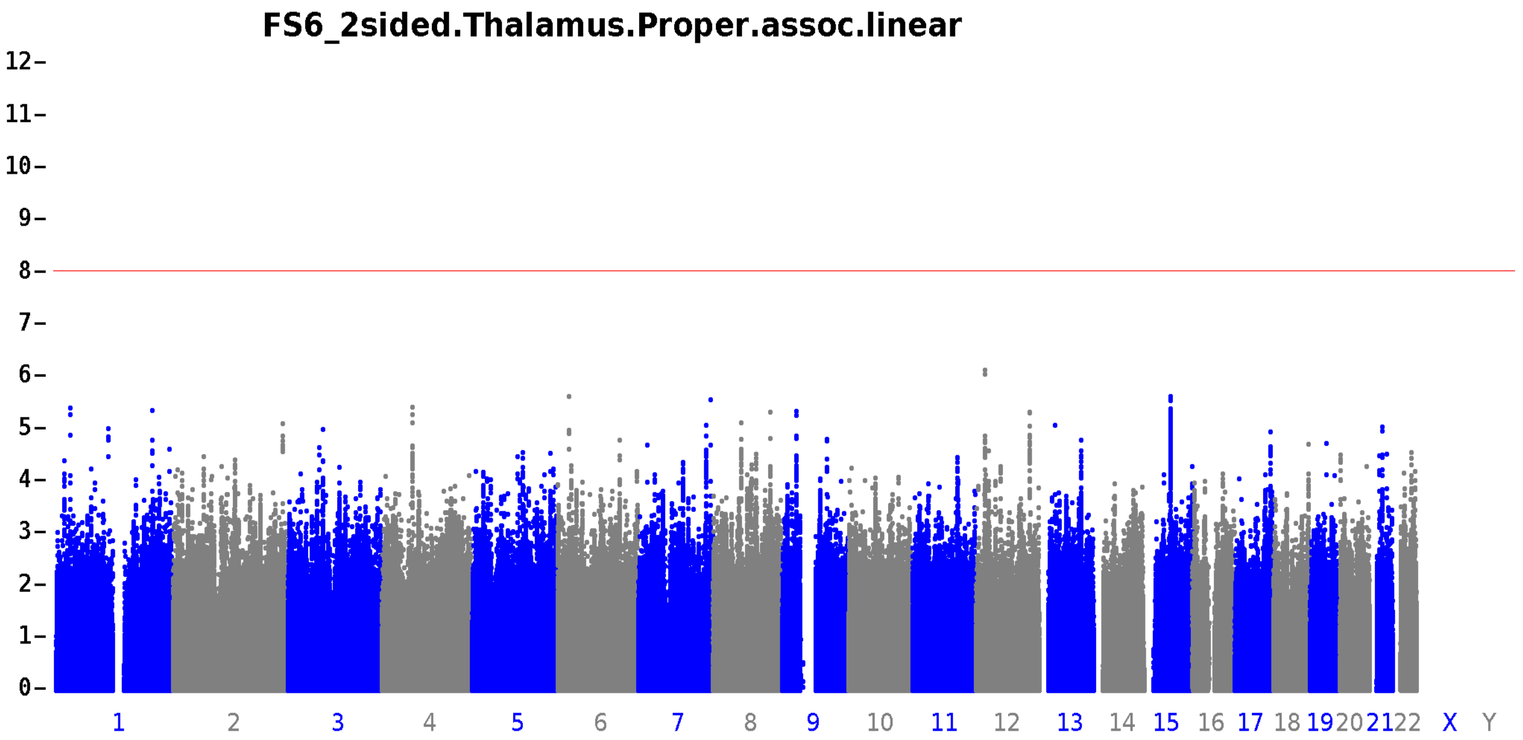

SNP mapped to gene:

CHR	BP	SNP	P	GENE
12	19420064	rs17343287	7.011e-07	PLEKHA5
12	19450236	rs17271690	8.447e-07	PLEKHA5
15	56517816	rs11636035	2.272e-06	RFX7
6	24990085	rs12205717	2.287e-06	missing
15	56761558	rs72742676	2.548e-06	missing
7	154209858	rs12668377	2.63e-06	DPP6
15	56759941	rs72742674	2.653e-06	missing
15	56465429	chr15:56465429:D	2.672e-06	RFX7
4	65008382	rs4860568	3.646e-06	missing
1	31330844	rs3935266	3.794e-06	missing
15	56510287	rs12591093	3.89e-06	RFX7
15	56418771	rs12595072	4.009e-06	RFX7
1	204728542	rs12070501	4.133e-06	missing
9	29542660	chr9:29542660:D	4.327e-06	missing
8	121645514	rs7840986	4.46e-06	SNTB1
15	56416620	chr15:56416620:I	4.505e-06	RFX7
12	113443222	rs2013593	4.561e-06	OAS2
12	113444418	rs929291	4.678e-06	OAS2
15	56772174	rs72742680	4.711e-06	missing
15	56494889	rs72738651	4.758e-06	RFX7
15	56781255	rs16976932	4.905e-06	missing
15	56425189	rs72738604	4.92e-06	RFX7
4	65025545	rs80133642	4.981e-06	missing
1	31330732	rs2506968	5.06e-06	missing

15	56765899	rs72742677	5.127e-06	missing
9	29539557	rs471561	5.203e-06	missing
15	56767730	rs77699201	5.373e-06	missing
15	56410911	rs72736495	5.703e-06	RFX7
15	56413388	rs11634404	5.703e-06	RFX7
15	56404221	rs76347629	6.227e-06	RFX7
15	56370951	rs1553738	6.275e-06	missing
15	56370492	rs1553737	6.275e-06	missing
15	56521525	rs76680454	6.307e-06	RFX7
15	56406027	rs12443118	6.309e-06	RFX7
15	56400338	rs139161093	6.309e-06	RFX7
15	56398952	rs72736479	6.309e-06	RFX7
15	56423732	chr15:56423732:D	6.31e-06	RFX7
15	56557715	rs72740524	6.343e-06	TEX9
15	56420710	rs72736498	6.363e-06	RFX7
15	56524136	rs11631915	6.713e-06	RFX7
15	56465885	rs12592130	6.772e-06	RFX7
15	56487811	rs16976801	6.921e-06	RFX7
15	56487683	rs17841131	6.921e-06	RFX7
15	56422623	chr15:56422623:D	6.969e-06	RFX7
15	56444381	rs150235055	7.036e-06	RFX7
15	56444901	rs79941117	7.036e-06	RFX7
15	56372846	rs72736465	7.171e-06	missing
15	56425746	rs72738608	7.198e-06	RFX7
4	65014121	rs114706214	7.307e-06	missing
8	59382092	rs59985577	7.362e-06	missing
15	56441360	rs11631518	7.368e-06	RFX7
15	56454223	rs764014	7.368e-06	RFX7
15	56466615	rs72738631	7.368e-06	RFX7
15	56451415	rs72738618	7.368e-06	RFX7
15	56439202	rs11636040	7.368e-06	RFX7
15	56436240	rs1867089	7.368e-06	RFX7
15	56451242	rs72738616	7.368e-06	RFX7
15	56434956	rs1550586	7.368e-06	RFX7
2	231438559	rs112324089	7.453e-06	TPM3P8
15	56492774	rs12594860	7.611e-06	RFX7
15	56471123	rs11071253	7.758e-06	RFX7
15	56485061	rs1879349	7.758e-06	RFX7,LOC390586
15	56485411	rs56156930	7.758e-06	RFX7,LOC390586
15	56484363	rs10518839	7.758e-06	RFX7,LOC390586
15	56485967	rs12593752	7.758e-06	RFX7
15	56510439	rs78768773	7.857e-06	RFX7
15	56423061	rs72736501	7.906e-06	RFX7
13	32979682	chr13:32979682:D	8.132e-06	N4BP2L1
15	56457442	rs11632023	8.158e-06	RFX7
15	56459914	rs12595366	8.158e-06	RFX7
15	56460951	rs12593569	8.158e-06	RFX7

15	56449456	rs72738615	8.192e-06	RFX7
7	143797346	rs12703572	8.204e-06	missing
15	56496525	rs10518843	8.205e-06	RFX7
15	56501019	rs72738666	8.205e-06	RFX7
15	56500907	rs72738664	8.205e-06	RFX7
15	56501415	rs72738668	8.205e-06	RFX7
15	56500064	rs72738662	8.205e-06	RFX7
15	56494711	rs60210425	8.205e-06	RFX7
15	56495136	rs10518842	8.205e-06	RFX7
15	56493724	rs11638709	8.205e-06	RFX7
15	56495555	rs16976821	8.205e-06	RFX7
15	56493287	rs12594908	8.205e-06	RFX7
15	56495784	rs118135259	8.205e-06	RFX7
15	56491847	rs72738648	8.205e-06	RFX7
15	56497133	rs72738658	8.205e-06	RFX7
15	56496952	rs72738656	8.205e-06	RFX7
15	56497140	rs72738659	8.205e-06	RFX7
15	56491665	rs16976810	8.205e-06	RFX7
15	56498700	rs72738660	8.205e-06	RFX7
15	56490848	rs72738646	8.205e-06	RFX7
15	56499326	rs12591980	8.205e-06	RFX7
15	56490549	rs11634455	8.205e-06	RFX7
15	56499380	rs12594245	8.205e-06	RFX7
15	56489159	rs1879347	8.205e-06	RFX7
15	56499986	rs12591236	8.205e-06	RFX7
15	56488934	rs1879345	8.205e-06	RFX7
15	56496290	rs72738654	8.31e-06	RFX7
12	113392182	rs7310667	8.364e-06	OAS3
15	56426852	rs181139921	8.441e-06	RFX7
15	56446120	rs58084889	8.499e-06	RFX7
15	56494368	rs12595649	8.575e-06	RFX7
15	56474085	rs72738638	8.593e-06	RFX7
15	56473321	rs72738636	8.593e-06	RFX7
15	56478983	rs61270078	8.593e-06	RFX7
15	56473040	rs75251003	8.593e-06	RFX7
15	56525216	rs12592881	8.605e-06	RFX7
15	56405467	rs11633911	8.829e-06	RFX7
15	56523152	rs12594121	8.836e-06	RFX7
15	56775597	rs11636802	8.836e-06	missing
15	56775521	rs11636793	8.836e-06	missing
21	25851193	rs57936699	8.852e-06	missing
15	56477485	rs16976792	8.867e-06	RFX7
15	56531755	rs12594038	8.898e-06	RFX7
15	56531056	rs72738701	8.898e-06	RFX7
15	56531365	rs12593439	8.898e-06	RFX7
15	56532219	rs72740503	8.898e-06	RFX7
15	56410918	rs116140504	8.978e-06	RFX7

15	56408168	rs72736484	8.978e-06	RFX7
15	56518254	rs72738694	8.983e-06	RFX7
15	56524787	chr15:56524787:D	8.983e-06	RFX7
15	56524307	rs11632087	8.983e-06	RFX7
15	56525311	rs12592899	8.983e-06	RFX7
15	56524268	rs11632081	8.983e-06	RFX7
15	56526217	rs16976832	8.983e-06	RFX7
15	56523659	rs140751507	8.983e-06	RFX7
15	56526620	rs55957636	8.983e-06	RFX7
15	56523255	rs11071255	8.983e-06	RFX7
15	56526648	rs56215018	8.983e-06	RFX7
15	56522988	rs11630394	8.983e-06	RFX7
15	56527163	rs2414467	8.983e-06	RFX7
15	56522460	rs16976831	8.983e-06	RFX7
15	56527277	rs2414468	8.983e-06	RFX7
15	56521898	rs12595557	8.983e-06	RFX7
15	56528764	rs12595490	8.983e-06	RFX7
15	56521137	rs72738697	8.983e-06	RFX7
15	56528806	rs12595496	8.983e-06	RFX7
15	56520860	rs76740259	8.983e-06	RFX7
15	56533177	rs72740506	8.983e-06	RFX7
15	56520352	rs72738696	8.983e-06	RFX7
15	56533340	rs72740508	8.983e-06	RFX7
15	56519917	rs74427244	8.983e-06	RFX7
15	56533519	rs72740509	8.983e-06	RFX7
15	56518208	rs35607436	8.983e-06	RFX7
15	56506298	rs12593096	9.013e-06	RFX7
15	56396132	rs17238607	9.034e-06	RFX7
15	56456230	rs72738620	9.079e-06	RFX7
15	56409172	rs59025920	9.226e-06	RFX7
15	56507616	rs79096665	9.393e-06	RFX7
15	56508995	rs11632619	9.393e-06	RFX7
15	56509389	rs72738680	9.393e-06	RFX7
15	56508104	rs75833539	9.393e-06	RFX7
15	56510059	rs10851604	9.393e-06	RFX7
15	56507635	rs72738676	9.393e-06	RFX7
15	56510796	rs72738682	9.393e-06	RFX7
15	56507438	rs72738674	9.393e-06	RFX7
15	56510989	rs72738683	9.393e-06	RFX7
15	56507015	rs11629809	9.393e-06	RFX7
15	56511122	rs72738684	9.393e-06	RFX7
15	56506243	rs12595323	9.393e-06	RFX7
15	56511363	rs72738685	9.393e-06	RFX7
15	56505714	rs2414465	9.393e-06	RFX7
15	56511561	rs72738686	9.393e-06	RFX7
15	56504598	rs8030605	9.393e-06	RFX7
15	56511683	rs11637515	9.393e-06	RFX7

15	56502930	rs2899597	9.393e-06	RFX7
15	56511949	rs11632394	9.393e-06	RFX7
15	56502586	rs2899596	9.393e-06	RFX7
15	56512949	rs56083605	9.393e-06	RFX7
15	56512961	rs55942364	9.393e-06	RFX7
15	56513500	rs12593713	9.393e-06	RFX7
15	56516381	rs58934221	9.393e-06	RFX7
1	111014608	chr1:111014608:D	9.483e-06	missing
3	75125657	chr3:75125657:I	9.615e-06	missing
15	56469583	rs11629674	9.866e-06	RFX7
15	56469488	rs34401800	9.866e-06	RFX7

Manhattan Plot:

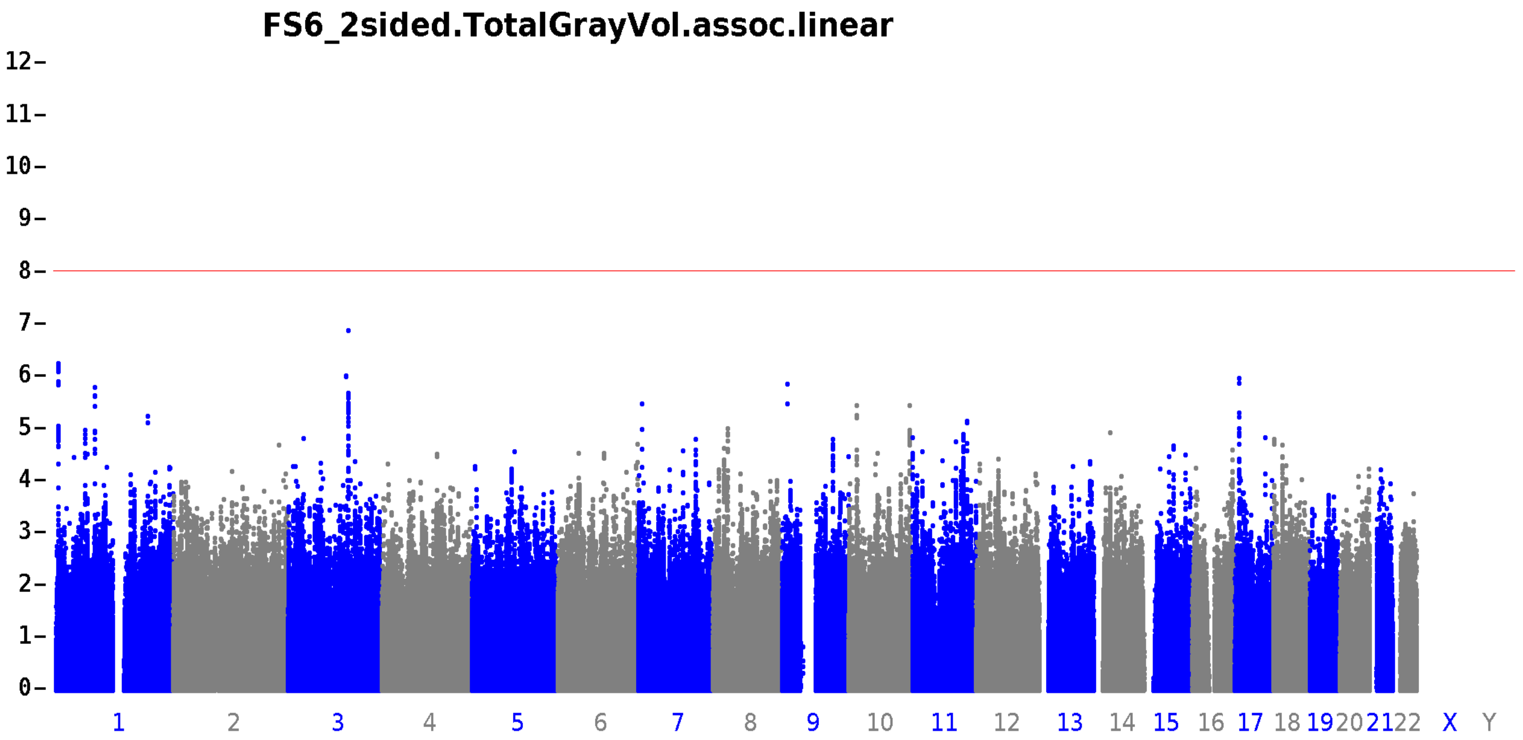

SNP mapped to gene:

CHR	BP	SNP	P	GENE
3	128650296	chr3:128650296:I	1.248e-07	LOC100132731
1	5337271	rs61759358	5.367e-07	missing
1	5341689	rs57925978	5.796e-07	missing
1	5341784	rs17423575	5.796e-07	missing
1	5341296	rs61558245	6.321e-07	missing
1	5341289	rs58812660	6.321e-07	missing
1	5341248	rs17457420	6.321e-07	missing
1	5340869	rs56278800	6.321e-07	missing
1	5341819	chr1:5341819:D	6.744e-07	missing
1	5339531	rs66495077	6.785e-07	missing
1	5339744	rs56056508	7.707e-07	missing
3	122683207	rs12635724	9.081e-07	SEMA5B
3	122683171	rs12630339	9.462e-07	SEMA5B
17	8968500	rs9910696	1.003e-06	NTN1
1	5341464	rs17423547	1.194e-06	missing
17	8970198	rs72660225	1.271e-06	NTN1
9	10865386	rs78322468	1.317e-06	missing
1	5340385	rs12143953	1.356e-06	missing
1	82230850	rs61775263	1.492e-06	missing
1	82230758	rs77706956	1.492e-06	missing
3	128595795	rs9825854	1.951e-06	missing
3	128570263	rs16851607	1.994e-06	missing
3	128593259	rs6796031	2.011e-06	missing
3	128594373	rs9847759	2.011e-06	missing

3	128592669	rs6795293	2.011e-06	missing
3	128595514	rs9825119	2.011e-06	missing
3	128596734	chr3:128596734:I	2.011e-06	missing
3	128592457	rs6768014	2.112e-06	missing
1	82226762	rs61775262	2.148e-06	missing
1	82225303	rs61775260	2.287e-06	missing
3	128652553	rs9810890	2.44e-06	LOC100132731
3	128591267	rs6789094	3.074e-06	missing
3	128647823	rs6778057	3.101e-06	LOC100132731
3	128641995	chr3:128641995:D	3.101e-06	LOC100132731
9	10868024	rs79172070	3.185e-06	missing
9	10868300	rs74615352	3.185e-06	missing
7	8796378	rs10486254	3.191e-06	missing
3	128609255	rs13434201	3.314e-06	ACAD9
10	17032281	rs2291521	3.327e-06	CUBN
10	129384543	rs12774813	3.439e-06	missing
1	82235239	rs61775265	3.529e-06	missing
1	82234965	rs61775264	3.529e-06	missing
3	128571974	rs13323780	3.579e-06	missing
3	128662213	chr3:128662213:I	3.609e-06	LOC100132731
3	128590405	rs9826700	3.722e-06	missing
3	128587629	rs6808675	3.722e-06	LOC653712
3	128587818	rs6796590	3.722e-06	LOC653712
3	128585471	rs60876670	3.722e-06	LOC653712
3	128583514	rs68127468	3.787e-06	LOC653712
3	128583073	rs11923686	3.787e-06	LOC653712
3	128583226	rs78115835	3.787e-06	LOC653712
3	128581001	rs11921456	3.787e-06	LOC653712
3	128575353	rs28675843	3.787e-06	missing
3	128575365	rs28694035	3.787e-06	missing
3	128572314	rs9862461	3.787e-06	missing
3	128571735	rs13322981	3.787e-06	MARK2P6
3	128649081	rs9869254	3.919e-06	LOC100132731
3	128585715	rs56063711	4.018e-06	LOC653712
3	128623785	rs61096019	4.388e-06	ACAD9
3	128602135	rs13323787	4.481e-06	ACAD9
3	128588632	rs4927923	4.654e-06	LOC653712,LOC101927197
17	8967380	rs7222847	4.689e-06	NTN1
10	17030775	rs10904861	5.324e-06	CUBN
1	194665178	rs16837328	5.396e-06	missing
17	8969573	chr17:8969573:I	5.596e-06	NTN1
17	8969817	rs7215014	5.596e-06	NTN1
17	8969672	rs8066040	5.596e-06	NTN1
17	8968513	rs9910706	5.597e-06	NTN1
17	8968276	rs8065801	5.662e-06	NTN1
17	8967920	rs8078106	5.696e-06	NTN1
17	8968695	rs9905180	5.696e-06	NTN1

10	17032834	rs3816870	5.756e-06	CUBN
3	128595566	chr3:128595566:I	5.849e-06	missing
11	115482160	rs2446878	6.798e-06	missing
3	128572219	rs9862012	6.99e-06	missing
11	115482738	rs2446879	7.124e-06	missing
1	194626713	rs72739070	7.386e-06	missing
1	5338212	rs17457350	8.429e-06	missing
1	5341840	rs12738436	8.446e-06	missing
3	128647141	rs56000731	8.496e-06	LOC100132731
1	5341078	rs12733390	9.079e-06	missing
1	5340818	rs35263239	9.079e-06	missing
8	30316952	rs16876993	9.328e-06	RBPMS
17	8969667	rs6503176	9.489e-06	NTN1
7	8794010	rs10238890	9.578e-06	missing

Manhattan Plot:

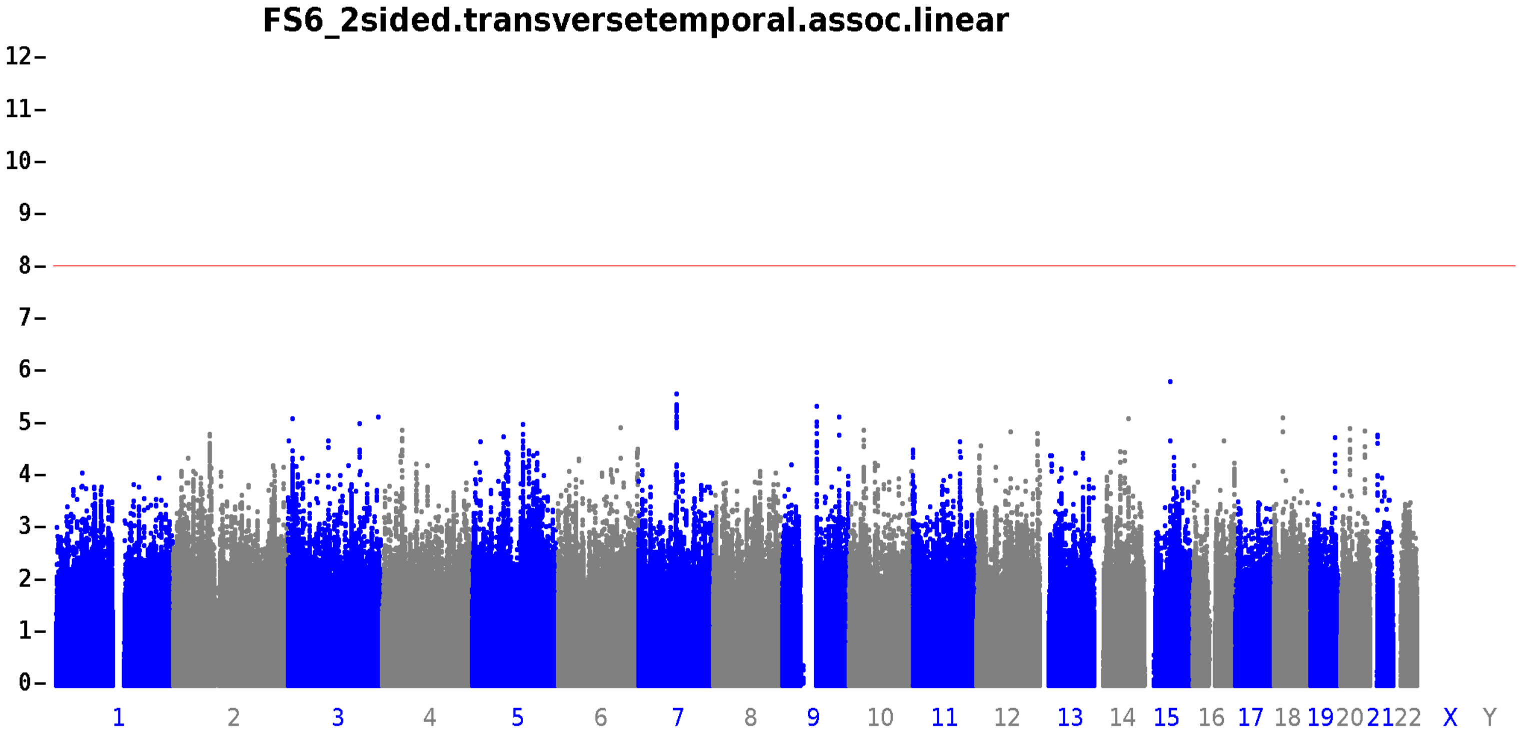

SNP mapped to gene:

CHR	BP	SNP	P	GENE
15	54806904	rs7176858	1.456e-06	UNC13C
7	81365789	rs12671260	2.499e-06	HGF
7	81368427	rs6945750	2.548e-06	HGF
7	81362506	rs757831	4.029e-06	HGF
7	81370264	rs9642131	4.22e-06	HGF
9	72483647	rs11792259	4.318e-06	C9orf135
9	72484431	rs11140741	4.318e-06	C9orf135
7	81359503	rs5745684	4.636e-06	HGF
7	81363334	rs11975846	4.693e-06	HGF
7	81362190	rs2040968	5.469e-06	HGF
7	81359950	chr7:81359950:D	6.794e-06	HGF
3	191308489	chr3:191308489:I	6.93e-06	missing
9	119635810	chr9:119635810:I	6.999e-06	ASTN2
18	19627576	rs9948417	7.156e-06	missing
7	81364187	rs12540393	7.16e-06	HGF
7	81367396	rs10268100	7.165e-06	HGF
14	72834181	rs7158564	7.502e-06	RGS6
3	8971787	rs524898	7.582e-06	RAD18
9	72443953	rs9792493	8.646e-06	C9orf135
7	81373514	rs4732405	8.782e-06	HGF
3	152638047	rs142660115	9.397e-06	missing
5	107427637	rs41131	9.591e-06	FBXL17
7	81357055	rs7810969	9.884e-06	HGF

Manhattan Plot:

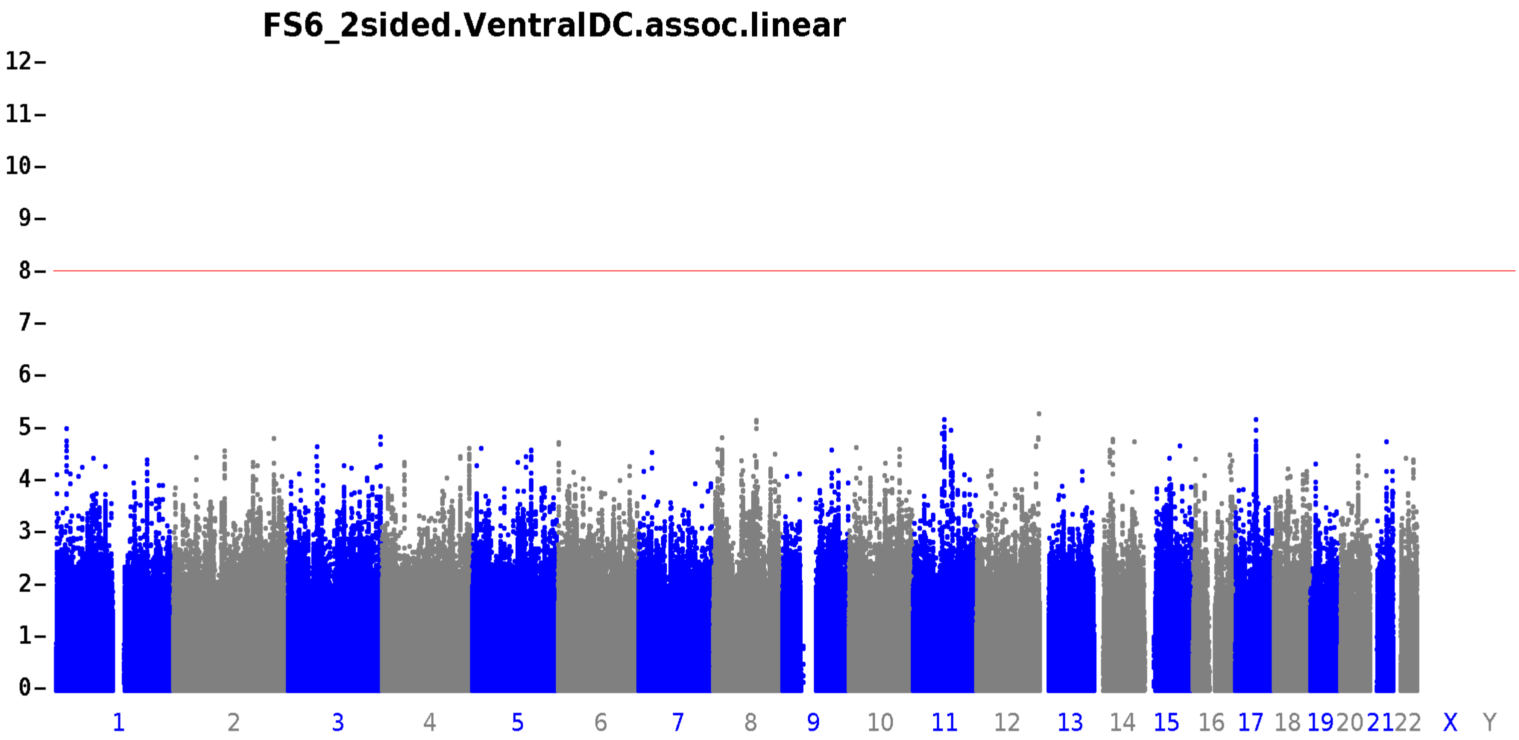

SNP mapped to gene:

CHR	BP	SNP	P	GENE
12	133061081	rs61951158	4.799e-06	missing
11	67204342	rs12787021	6.306e-06	PTPRCAP
17	44228169	rs114974956	6.338e-06	KANSL1
8	90363808	rs6981493	6.528e-06	missing
8	90363204	rs7018458	6.528e-06	missing
8	90363889	chr8:90363889:I	7.085e-06	missing
11	67245831	rs61889868	8.581e-06	missing
8	90364065	rs1904521	9.387e-06	missing
1	23122549	rs7517616	9.51e-06	EPHB2

Manhattan Plot:

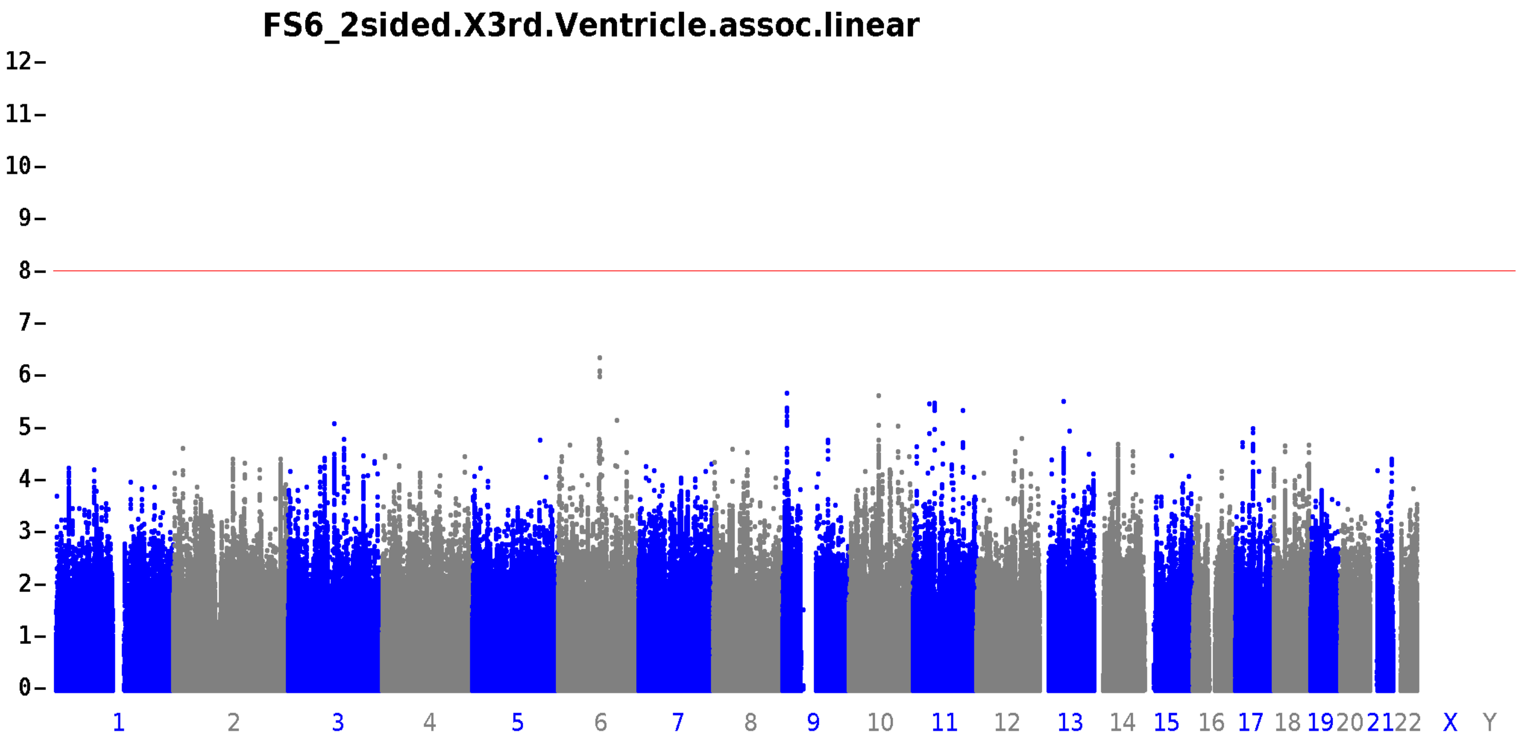

SNP mapped to gene:

CHR	BP	SNP	P	GENE
6	89983196	rs62416351	4.035e-07	GABRR2
6	89985622	rs41530450	7.293e-07	GABRR2
6	89988307	rs11759189	7.547e-07	GABRR2
6	89982382	rs62416350	9.408e-07	GABRR2
9	9428410	rs148326517	1.945e-06	PTPRD
10	62343371	rs7924180	2.194e-06	ANK3
13	50609321	chr13:50609321:D	2.771e-06	DLEU2
11	45391351	rs12365508	3.081e-06	missing
11	34396539	rs7927163	3.193e-06	missing
11	45390519	rs7130052	3.295e-06	missing
11	45389225	rs11601710	3.587e-06	missing
9	9411763	rs72702530	3.772e-06	PTPRD
9	9411251	rs2184080	3.796e-06	PTPRD
9	9410922	rs72702528	3.796e-06	PTPRD
9	9409860	rs72702527	3.796e-06	PTPRD
9	9409698	rs72702526	3.796e-06	PTPRD
9	9406190	rs72702522	3.796e-06	PTPRD
11	106969071	rs117259798	4.218e-06	missing
11	45400537	rs1989169	4.241e-06	FLJ41423
9	9432088	rs11794871	4.345e-06	PTPRD
9	9412457	chr9:9412457:D	5.473e-06	PTPRD
6	124824802	rs626967	6.415e-06	NKAIN2
9	9414162	rs1412869	6.815e-06	PTPRD
9	9417430	rs16929315	7.138e-06	PTPRD

9	9414893	rs11790368	7.407e-06	PTPRD
9	9416914	chr9:9416914:I	7.407e-06	PTPRD
3	98394871	rs1584335	7.65e-06	missing
10	62346398	rs7922717	7.95e-06	ANK3
9	9419392	rs11791073	8.199e-06	PTPRD
10	103831598	rs11191205	8.447e-06	missing
17	38116339	chr17:38116339:I	9.207e-06	missing
11	45401413	rs896334	9.629e-06	FLJ41423

Manhattan Plot:

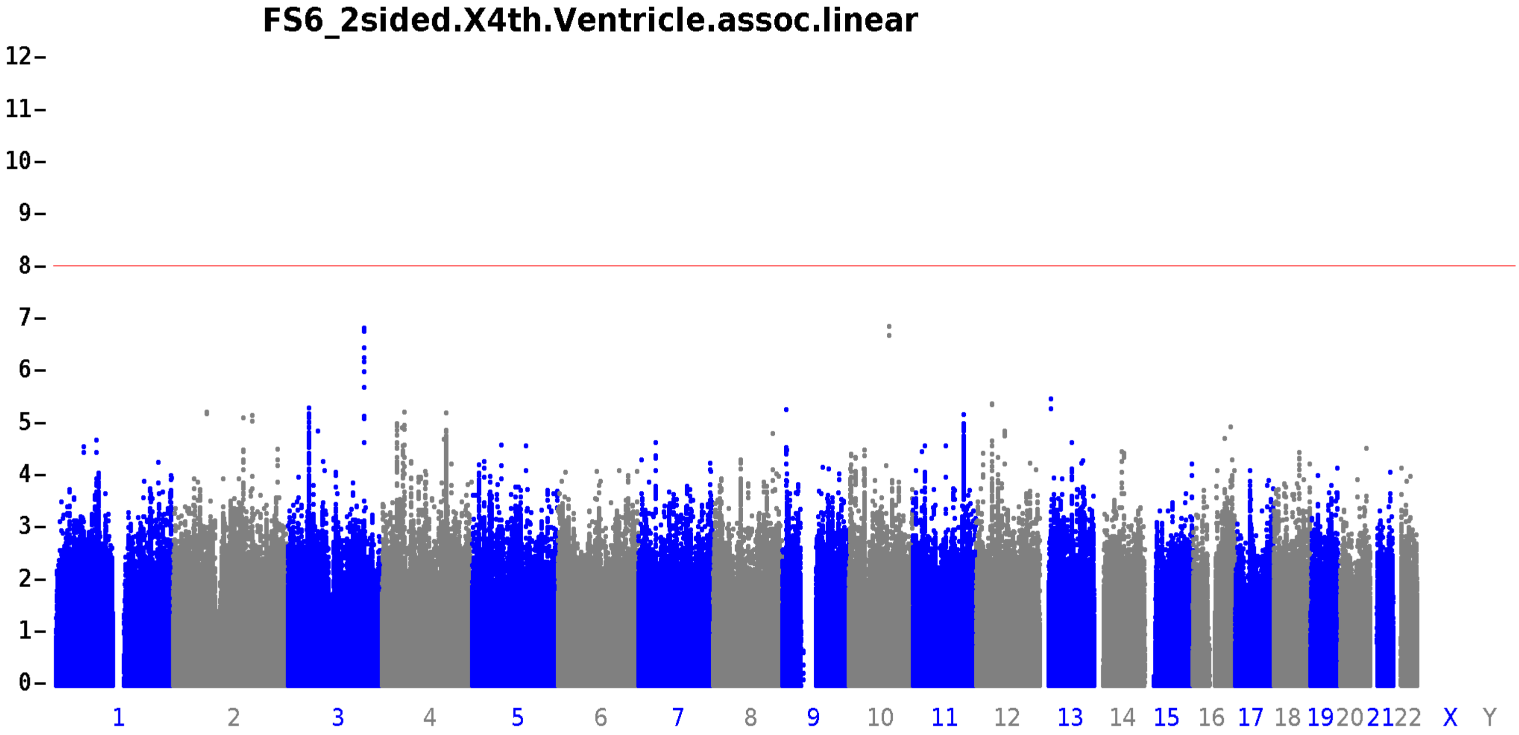

SNP mapped to gene:

CHR	BP	SNP	P	GENE
10	84980080	rs11197818	1.272e-07	missing
3	161587166	rs2220684	1.375e-07	missing
3	161581996	rs6441409	1.586e-07	missing
10	84979839	rs59151049	1.934e-07	missing
3	161575182	chr3:161575182:I	3.333e-07	missing
3	161582755	rs4856730	3.337e-07	missing
3	161560079	rs4856729	5.142e-07	missing
3	161564813	rs7426852	6.025e-07	missing
3	161602310	rs13095765	6.227e-07	missing
3	161566739	rs6771433	9.554e-07	missing
3	161564798	chr3:161564798:D	9.554e-07	missing
3	161561379	rs9811500	9.627e-07	missing
3	161571447	rs7431241	1.914e-06	missing
13	24430875	rs7325694	3.108e-06	MIPEP
12	32418251	rs161981	3.941e-06	BICD1
12	32416346	rs2632364	4.106e-06	BICD1
3	44639615	chr3:44639615:D	4.697e-06	ZNF660
13	24429097	rs9507174	4.898e-06	MIPEP
9	7874018	rs112156520	4.969e-06	missing
2	70867256	rs61490593	5.533e-06	missing
4	47512364	rs73237070	5.673e-06	ATP10D
4	135285067	chr4:135285067:I	5.881e-06	missing
2	70862912	rs7565278	5.997e-06	missing
2	70861189	rs59502136	5.997e-06	missing

2	70860250	rs73936984	5.997e-06	missing
3	44657661	rs6800701	6.072e-06	missing
11	107180453	chr11:107180453:D	6.289e-06	missing
2	167674236	rs6724120	6.437e-06	missing
3	44655286	rs7643602	6.56e-06	missing
3	161568993	rs7433024	6.817e-06	missing
3	161563675	rs6441404	6.879e-06	missing
3	44748516	rs6795322	7.073e-06	missing
3	44746931	rs9814722	7.209e-06	missing
2	149054431	chr2:149054431:D	7.372e-06	MBD5
3	161563986	rs6441405	7.402e-06	missing
3	161567025	rs6796264	7.402e-06	missing
2	167675894	rs12994437	8.454e-06	missing
3	44732013	rs13058913	8.559e-06	missing
3	44731730	rs13058822	8.559e-06	missing
3	44673430	rs28699350	8.753e-06	ZNF197
3	44660856	rs2009644	9.018e-06	missing
4	31333616	rs10025805	9.299e-06	missing
11	107320644	rs687991	9.445e-06	CWF19L2
4	47512763	rs4423850	9.945e-06	ATP10D

Manhattan Plot:

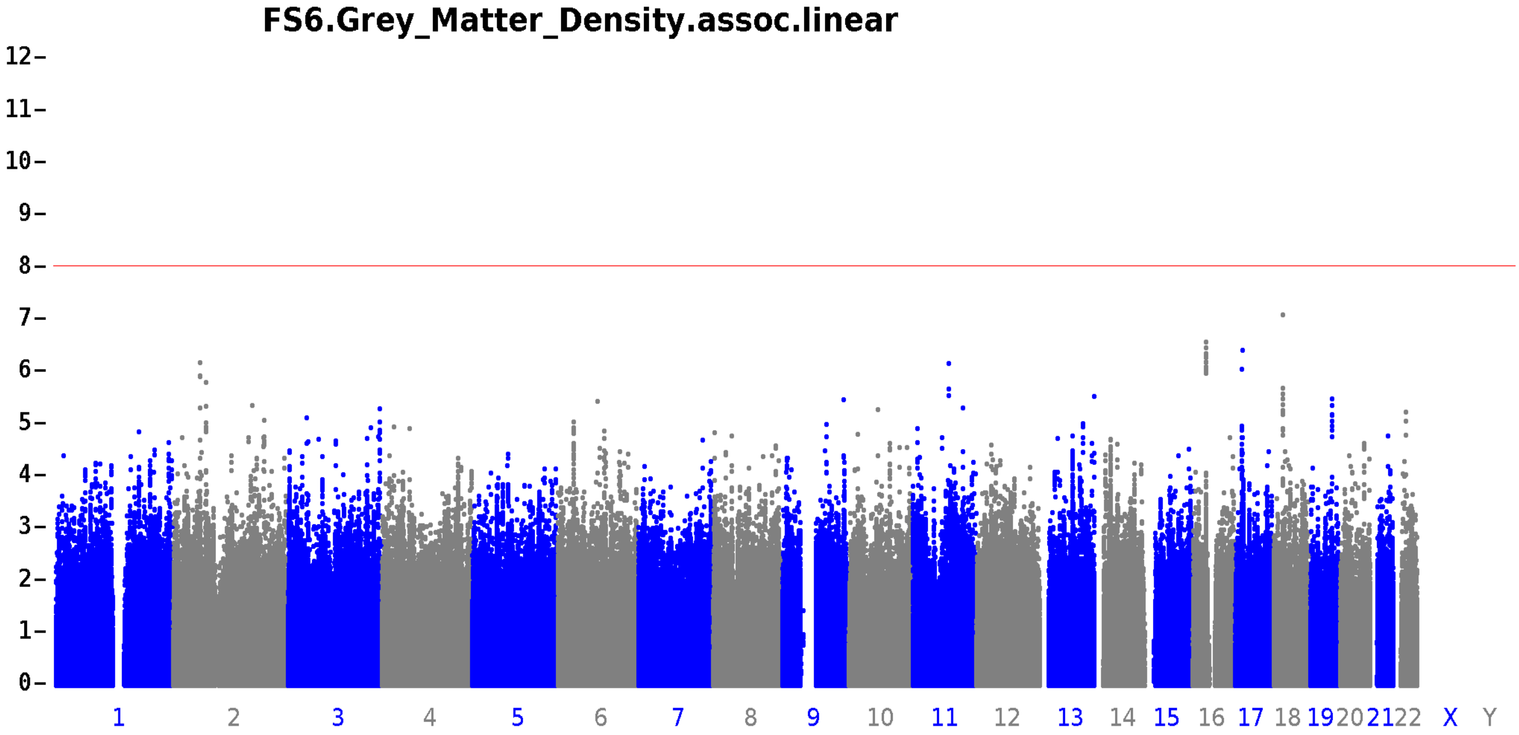

SNP mapped to gene:

CHR	BP	SNP	P	GENE
18	19647942	rs16962746	7.646e-08	missing
18	19649765	rs80046192	7.735e-08	missing
16	27712553	rs964002	2.586e-07	KIAA0556
16	27656641	rs233466	3.348e-07	KIAA0556
17	15833593	rs113133607	3.656e-07	missing
16	27756096	rs12924607	4.32e-07	KIAA0556
16	27599139	rs1018885	4.43e-07	KIAA0556
16	27690604	rs12445661	4.656e-07	KIAA0556
16	27680989	rs12920717	4.656e-07	KIAA0556
16	27738012	rs34342175	5.176e-07	KIAA0556
16	27725864	rs12924688	6.124e-07	KIAA0556,LOC100128079
2	57096095	rs13386863	6.35e-07	missing
2	57095273	rs10165631	6.35e-07	missing
2	57095507	rs13383501	6.35e-07	missing
2	57093249	rs7559064	6.35e-07	missing
16	27670114	rs233472	6.357e-07	KIAA0556
16	27672093	rs233474	6.357e-07	KIAA0556
16	27670235	rs233473	6.357e-07	KIAA0556
11	75890907	rs689196	6.661e-07	missing
16	27753737	rs12928569	7.59e-07	KIAA0556
16	27733715	rs12102783	8.012e-07	KIAA0556
16	27651529	rs233459	8.278e-07	KIAA0556
16	27720201	rs12930355	8.538e-07	KIAA0556,LOC100128079
17	13081057	rs78685864	8.629e-07	missing

16	27559815	rs4787968	8.829e-07	GTF3C1
16	27642249	rs233453	8.945e-07	KIAA0556
16	27473860	rs12932325	9.105e-07	GTF3C1
16	27512760	rs232072	9.153e-07	GTF3C1
16	27479229	rs35913539	9.23e-07	GTF3C1
16	27479767	rs12933047	9.23e-07	GTF3C1
16	27474910	rs12919017	9.23e-07	GTF3C1
16	27472772	rs2228248	9.315e-07	GTF3C1
16	27491989	rs232059	9.821e-07	GTF3C1
16	27521415	rs232078	1.003e-06	GTF3C1
2	57085437	rs10197201	1.154e-06	missing
2	57091362	rs10170053	1.18e-06	missing
2	70104313	rs6752271	1.529e-06	GMCL1
18	19654059	rs78326335	1.945e-06	missing
18	19649927	rs16962752	1.945e-06	missing
18	19646225	rs79645390	1.983e-06	missing
18	19646665	rs79840590	1.983e-06	missing
11	75890695	rs582132	2.043e-06	missing
18	19641812	rs9807576	2.542e-06	missing
18	19641743	rs9807574	2.542e-06	missing
11	75892741	rs647159	2.74e-06	missing
13	114867087	rs61971965	2.862e-06	RASA3
19	46538822	rs2005893	3.145e-06	missing
19	46538837	chr19:46538837:D	3.145e-06	missing
18	19639611	rs142445227	3.192e-06	missing
18	19640888	rs78229063	3.192e-06	missing
18	19640965	rs74710777	3.192e-06	missing
18	19639898	rs115940844	3.192e-06	missing
18	19641221	rs147622478	3.192e-06	missing
18	19641384	rs114271232	3.192e-06	missing
9	130421581	chr9:130421581:I	3.262e-06	STXBP1
6	84772469	rs2480192	3.554e-06	MRAP2
18	19638425	rs4308039	4.015e-06	missing
18	19635182	rs114005506	4.015e-06	missing
18	19637907	rs78290752	4.015e-06	missing
19	46534913	rs57454787	4.253e-06	missing
19	46535246	rs11879766	4.253e-06	missing
19	46535401	rs73572708	4.253e-06	missing
19	46535928	rs8112441	4.253e-06	missing
2	166920814	rs57074029	4.256e-06	SCN1A
2	70073013	rs12328891	4.412e-06	GMCL1
11	106700051	rs75051536	4.685e-06	GUCY1A2
2	57077144	rs13387720	4.753e-06	missing
3	194438053	rs1471333	4.801e-06	LOC100507391
10	61688546	rs10994073	5.035e-06	missing
18	19626806	rs9989575	5.233e-06	missing
22	27100912	rs12166700	5.569e-06	missing

22	27104440	rs11090453	5.569e-06	missing
18	19644274	rs2891768	6.095e-06	missing
19	46537400	chr19:46537400:I	6.183e-06	missing
19	46537292	rs10407053	6.183e-06	missing
19	46537971	rs114722205	6.183e-06	missing
19	46538289	rs73572721	6.183e-06	missing
19	46537764	rs10407636	6.183e-06	missing
19	46538313	rs73572722	6.183e-06	missing
18	19532325	rs58358010	6.203e-06	missing
19	46537713	rs10407979	6.499e-06	missing
3	40277153	rs17076962	7.283e-06	MYRIP,EIF1B-AS1
3	40275461	rs34800524	7.283e-06	MYRIP,EIF1B-AS1
3	40279743	rs13080600	7.283e-06	MYRIP,EIF1B-AS1
2	192418945	rs4595916	8.108e-06	missing
19	46536552	rs111349128	8.344e-06	missing
19	46536647	rs73572710	8.344e-06	missing
22	27095998	rs7291023	8.423e-06	missing
3	194439908	rs1461774	8.543e-06	LOC100507391
6	33714781	rs530614	8.694e-06	missing
2	69561808	rs57318013	8.983e-06	GFPT1
13	91734983	rs10492554	9.485e-06	missing
9	93633487	rs11791054	9.597e-06	SYK

Manhattan Plot:

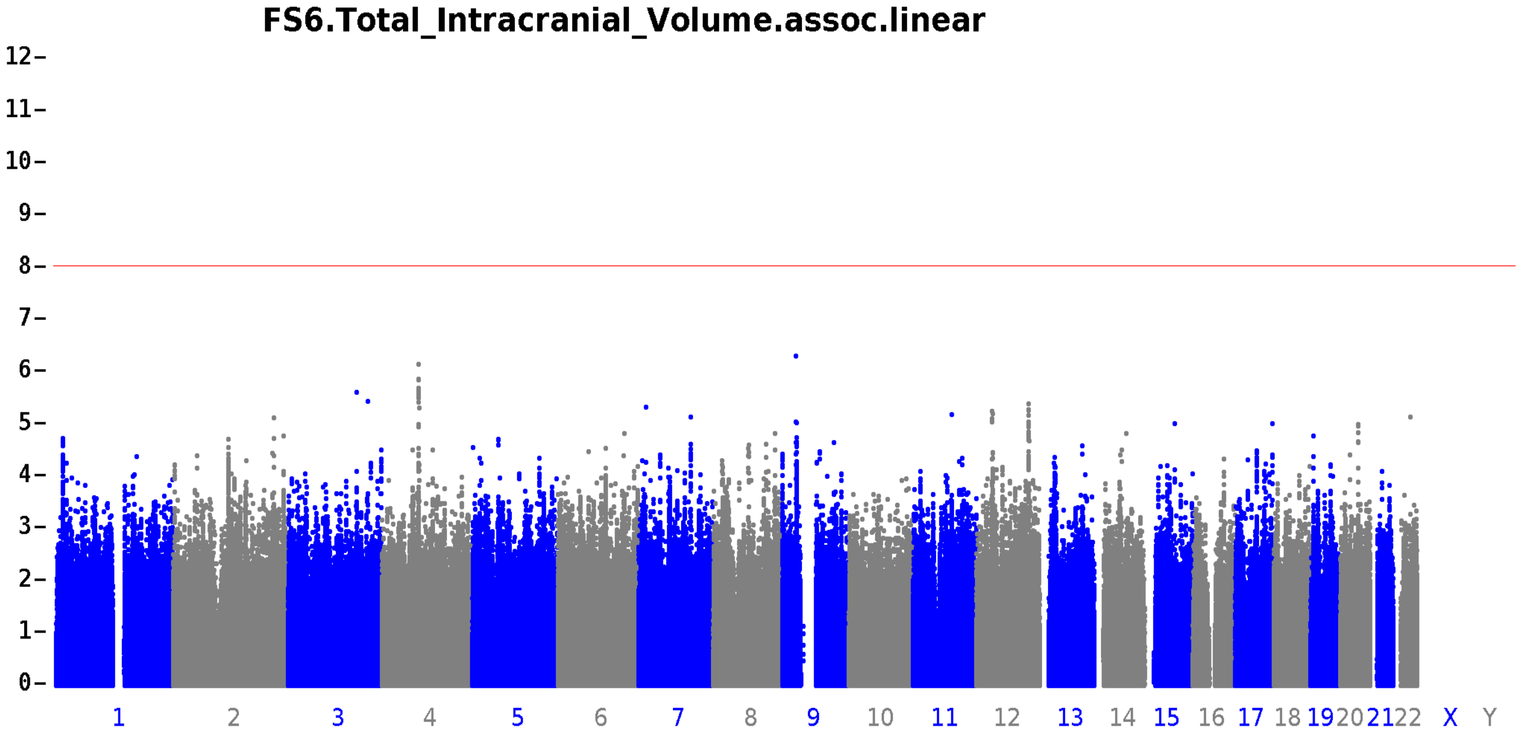

SNP mapped to gene:

CHR	BP	SNP	P	GENE
9	27977787	chr9:27977787:D	4.713e-07	LINGO2
4	76899968	chr4:76899968:I	6.928e-07	SDAD1
4	76910813	rs58909712	1.322e-06	SDAD1,LOC101928809
4	76909616	rs66477189	1.373e-06	SDAD1,LOC101928809
4	76923998	rs3733236	1.377e-06	LOC101928809,CXCL9
4	76885871	rs1910907	1.378e-06	SDAD1
4	76882771	rs17001284	1.977e-06	SDAD1
4	76901738	chr4:76901738:D	1.993e-06	SDAD1
4	76860935	rs67297326	2.127e-06	NAAA
4	76936098	rs12511861	2.264e-06	ART3
3	145749280	chr3:145749280:I	2.387e-06	missing
4	76864138	chr4:76864138:I	2.534e-06	missing
4	76861038	rs72651387	2.534e-06	NAAA
4	76886473	rs61279846	2.625e-06	SDAD1
4	76874996	rs17001275	2.906e-06	SDAD1
4	76866362	rs6828744	2.906e-06	missing
4	76875547	rs7696286	3.009e-06	SDAD1
3	169332833	rs7632977	3.545e-06	MECOM
4	76929593	rs6532084	3.627e-06	missing
12	110815922	rs28609096	3.948e-06	ANAPC7
7	15719407	rs7781366	4.544e-06	MEOX2
4	78136251	rs6817538	4.662e-06	missing
12	110602275	rs7975364	5.025e-06	IFT81
12	110705800	rs1986123	5.276e-06	missing

12	33642464	rs7297506	5.384e-06	missing
12	33752808	rs1873011	6.028e-06	missing
11	81964683	rs582124	6.284e-06	LOC101928989
12	110832027	rs34840178	6.421e-06	ANAPC7
22	36541725	rs1807672	7.013e-06	APOL3
7	111385671	rs4727760	7.041e-06	DOCK4
2	212912472	rs12616270	7.148e-06	ERBB4
12	33637975	rs12368022	7.56e-06	missing
12	110714855	chr12:110714855:I	8.552e-06	missing
9	27971144	rs4969136	8.61e-06	LINGO2
12	110587637	rs7309141	8.775e-06	IFT81
12	33565065	rs10844587	8.796e-06	SYT10
12	33563116	rs10844586	8.796e-06	SYT10
9	30284143	rs7854037	9.054e-06	missing
15	63312208	rs16946284	9.319e-06	LOC100287243
17	78407859	rs7218055	9.437e-06	ENDOV
20	37995172	rs6101487	9.667e-06	missing
4	76993951	rs12511359	9.697e-06	ART3
12	110571845	rs11065129	9.987e-06	IFT81

7. References

1. Desikan RS, Segonne F, Fischl B, Quinn BT, Dickerson BC, Blacker D, et al. An automated labeling system for subdividing the human cerebral cortex on MRI scans into gyral based regions of interest. Neuroimage. 2006;31(3):968-80.
2. Destrieux C, Fischl B, Dale A, Halgren E. Automatic parcellation of human cortical gyri and sulci using standard anatomical nomenclature. Neuroimage. 2010;53(1):1-15.
3. Fischl B, Salat DH, Busa E, Albert M, Dieterich M, Haselgrove C, et al. Whole brain segmentation: automated labeling of neuroanatomical structures in the human brain. Neuron. 2002;33(3):341-55.
4. Ashburner J, Friston KJ. Voxel-based morphometry--the methods. Neuroimage. 2000;11(6 Pt 1):805-21.
5. Ivleva EI, Clementz BA, Dutcher AM, Arnold SJM, Jeon-Slaughter H, Aslan S, et al. Brain Structure Biomarkers in the Psychosis Biotypes: Findings From the Bipolar-Schizophrenia Network for Intermediate Phenotypes. Biol Psychiatry. 2017;82(1):26-39.
6. Gauderman WJ. Sample size requirements for matched case-control studies of gene-environment interaction. Stat Med. 2002;21(1):35-50.
7. Gauderman WJM, J. M. QUANTO. version 1.2 ed. University of Southern California2007.
8. Watanabe K, Taskesen E, van Bochoven A, Posthuma D. Functional mapping and annotation of genetic associations with FUMA. Nat Commun. 2017;8(1):1826.
9. Miller JA, Ding SL, Sunkin SM, Smith KA, Ng L, Szafer A, et al. Transcriptional landscape of the prenatal human brain. Nature. 2014;508(7495):199-206.
